# An activator of tailless’ repressor function stimulates anti-neurodegenerative gene expression

**DOI:** 10.1021/acs.jmedchem.6c00734

**Published:** 2026-06-03

**Authors:** Emily C. Hank, Romy Busch, Úrsula López-García, Till Kasch, Loris Knümann, Vasily Morozov, Georg Höfner, Julian A. Marschner, Daniel Merk

**Affiliations:** 1 Ludwig-Maximilians-Universität München, Department of Pharmacy, 81377 Munich, Germany

## Abstract

The transcription factor tailless (TLX, NR2E1) is expressed in neuronal stem cells and retinal progenitor cells and thought to maintain their self-renewal by balancing proliferation and differentiation. It is considered as a master regulator of neurogenesis and as promising target in neurodegeneration. Here, we developed highly potent and selective activators of TLX’ transcriptional repressor activity by systematic extension of previously optimized fragment TLX ligands and tuning of the substitution pattern. An optimized TLX activator with nanomolar potency and pronounced selectivity was studied for its transcriptomic effects by RNAseq which demonstrated upregulation of genes associated with ATP production, lysosomal function, and vesicular transport. Moreover, the mitochondrial quality control associated PTEN-induced kinase 1 (PINK1) was induced and the amyloidogenic β-secretase (BACE) was downregulated supporting anti-neurodegenerative effects of TLX modulation on transcriptional level.

## Table of Contents Graphic

**Figure:**
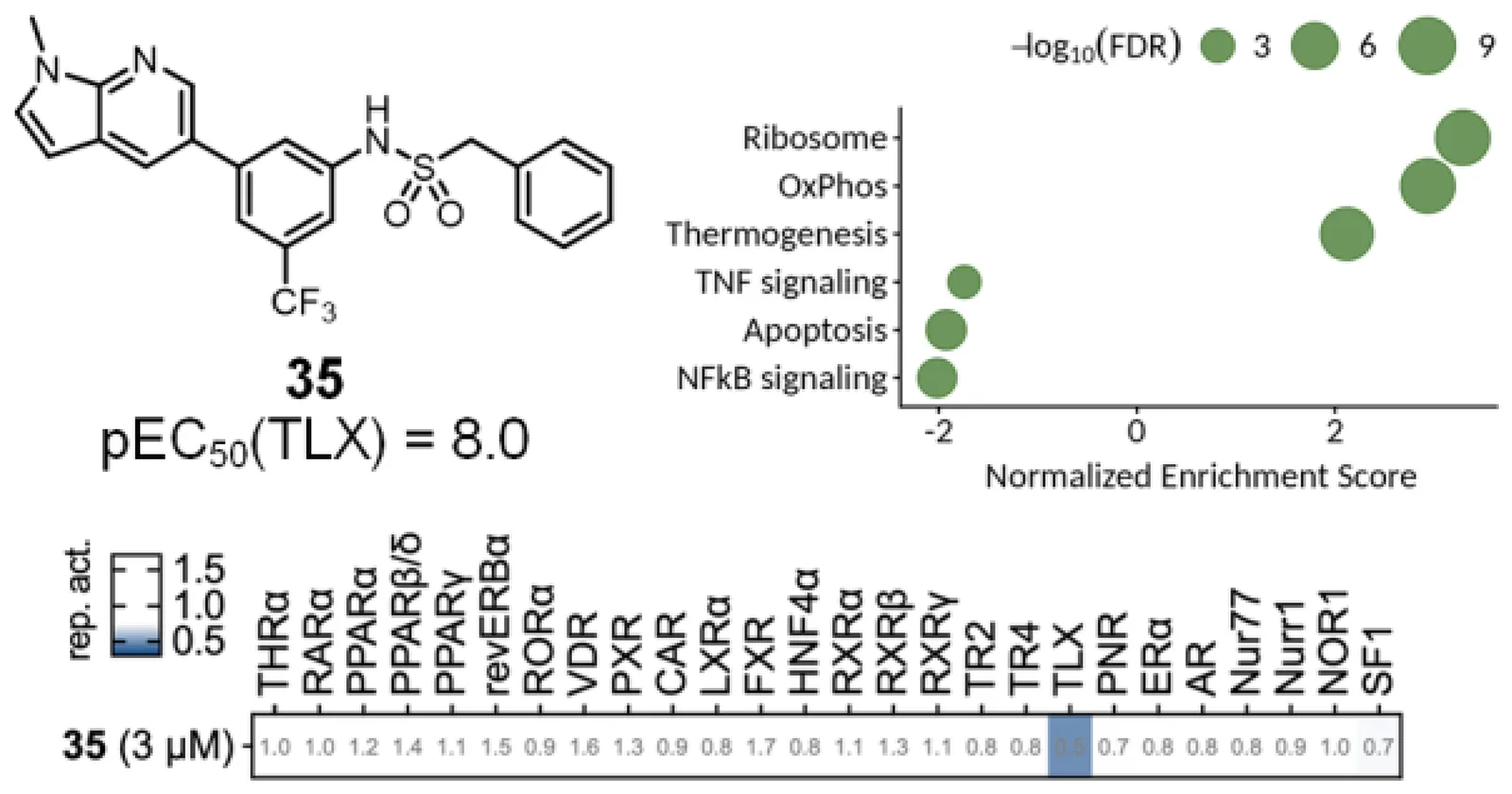

## Introduction

The transcription factor tailless homologue (TLX, NR2E1) is an orphan nuclear receptor (NR) found in neuronal stem cells (NSC) and retinal progenitor cells (RPC).^1,2^ It mainly acts as a transcriptional repressor and controls expression of cell cycle regulators, such as p21 and the phosphatase and tensin homologue (PTEN), to maintain NSC in an undifferentiated, self-renewing state.^1–7^ TLX is essential for normal brain development^8^ and has been linked to behavior^9–11^, memory function^11–13^, and learning capacity^7^ in vitro and in vivo. The stimulation of TLX activity seems to promote neurogenesis and protect from neuroinflammation.^7,14–17^ Moreover, TLX is important for vision and retinal health suggesting its activation as beneficial in retinal dystrophies^5,18–20^. Several lines of evidence thus highlight TLX as a promising target in neurodegenerative diseases which has stimulated the search for TLX modulators^21,22^.

**Figure 1:**
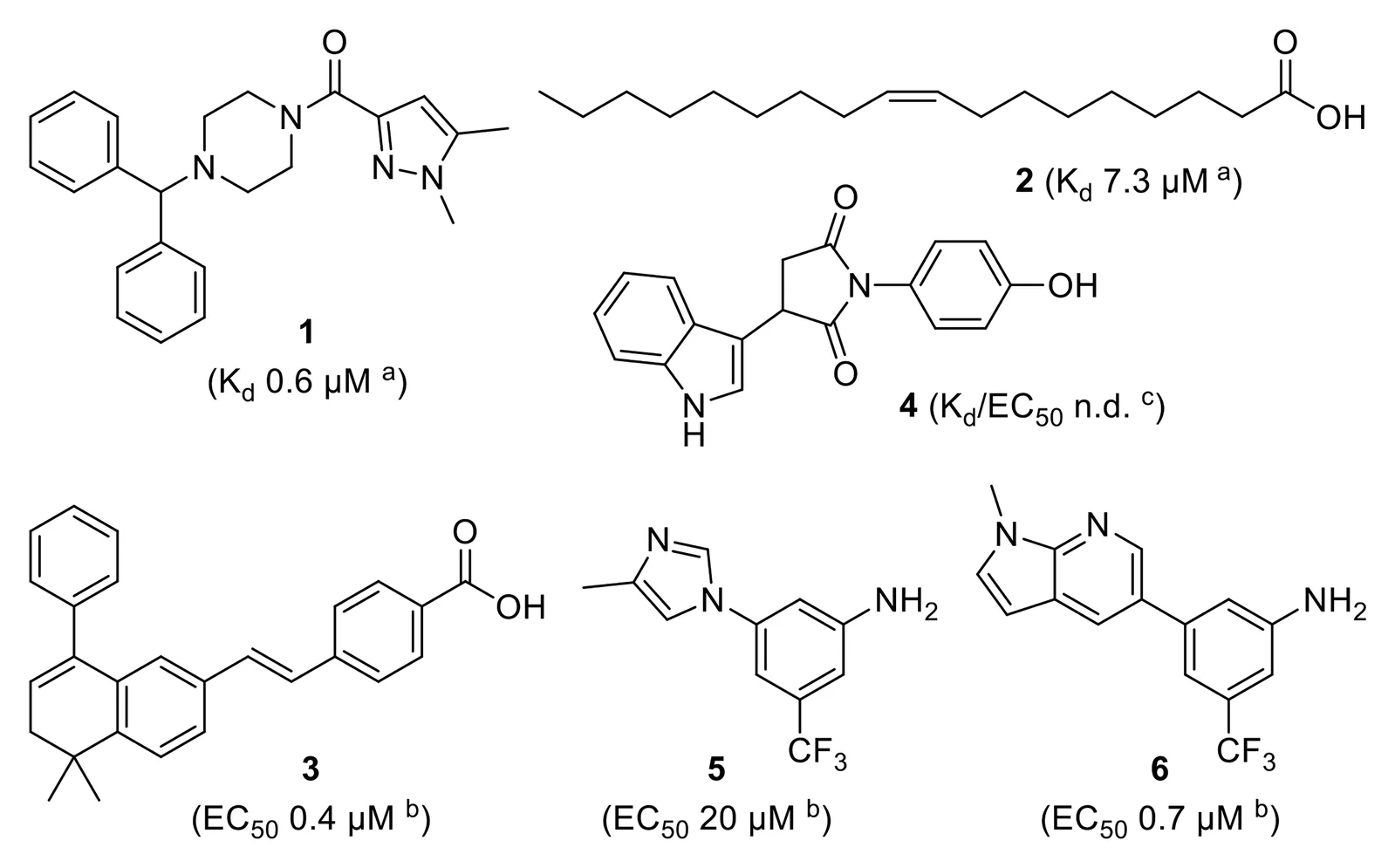
Published TLX agonists.^23–28^ ^a^ K_d_ determined by biolayer interferometry (BLI)^23,26, b^ EC_50_ determined in a Gal4-TLX hybrid reporter gene assay^24,25,27, c^ Compound **4** showed 50% inhibition of a 3xTAE-Luc reporter in a full-length TLX reporter gene assay at 35 µM^28^ (K_d_ or EC_50_ was not determined).

Early druggability evaluation by library screening in a thermal shift assay yielded the first three TLX ligands including ccrp2 (**1**, Figure 1) as the highest affinity entity (K_d_ 0.6 µM)^23^. Kandel et al.^26^ reported unsaturated fatty acids including oleic acid (**2**; K_d_ 7.3 µM) as relevant endogenous TLX ligands with oleic acid stimulating neurogenesis in vivo. Additionally, Griffett et al.^27^ discovered TLX modulation by natural and synthetic retinoids with all-(*E*)-retinal acting as inverse agonist and BMS453 (**3**) as activator of TLX’s repressor activity. Dueva et al.^28^ identified a number of chemically diverse TLX modulators (e.g., **4**) with weaker potency by virtual screening and we employed a focused drug fragment screening to identify both TLX agonists (e.g. **5**) and inverse agonists^24,29,30^. Recently, systematic SAR evaluation of the fragment screening hit **5** enabled marked optimization of potency and affinity up to sub-micromolar EC_50_ values in **6**^25^.

Here, we report the development of selective high-affinity TLX agonists from the most optimized fragment derivatives. Comparative evaluation of scaffold extension and systematic optimization of the substitution pattern resulted in TLX activators with double-digit nanomolar potency. A highly potent and selective representative was used to study the transcriptomic impact of the TLX repressor activation which revealed induction of respiratory chain components and ATP production genes as most prominent effect. Additionally, we observed altered expression of genes associated with neurodegeneration such as downregulation of β-secretase (BACE), which is linked to the amyloidogenic pathway in Alzheimer’s disease.

## Results & Discussion

Systematic optimization of the screening hit **5** recently enabled the development of four potent fragment TLX agonists (**6**-**9**, Table 1) with low micromolar to sub-micromolar TLX agonist potency.^25^ To refine this collection for further optimization, we scanned the suitability of **6**-**9** for structural extension by introducing *N*-benzyl (**10**-**13**) and phenylacetamide (**14**-**17**) substituents. The benzyl motif (**10**-**13**) consistently disrupted TLX agonism of all lead fragments while the phenylacetamide (**14**-**17**) consistently enhanced potency. However, the impact of this extension varied markedly with the smallest gain in potency observed for the most active lead fragment **6** and the strongest increase for **9**. Nevertheless, all phenylacetamide derivatives **14**-**17** displayed favorable TLX agonist potency with similar sub-micromolar EC_50_ values between 0.19 and 0.5 µM and hence appeared suitable as leads for further evaluation. We based our choice on the drug-like properties and prioritized **14** (logP 4.35^31^) and **16** (logP 5.08) for their lower lipophilicity compared to **15** (logP 5.16) and **17** (logP 5.41).

With the scaffolds of **6**/**14** and **8**/**16**, we next explored structural extension from the aniline in more detail (Table 2). Shorter benzamide substituents (**18, 19**), reduction of the phenylacetamides (**14, 16**) to the corresponding secondary amines (**20, 21**), and phenethyl ethers (**22, 23**) were detrimental for TLX activation. Inversion of the amide group (**24, 25**) was tolerated but slightly diminished potency. Urea (**26, 27**) and urethane (**28, 29**) linkers also retained slightly reduced TLX agonism, suggesting that rigid groups were favored, but incorporation of a cyclopropyl motif (**30, 31**) for rigidification of the phenylacetamide was only tolerated in the dimethylpyridine-based scaffold (**30**). *N*-Methylation of the acetamide (**32, 33**) was tolerated but accompanied by a loss of TLX activation efficacy. Only a phenylmethanesulfonamide motif (**34, 35**) could enhance activity on TLX when combined with the pyrrolo[2,3-*b*]pyridine-based fragment **6**. The resulting TLX agonist **35** displayed a remarkable EC_50_ value of 10 nM and emerged as first TLX ligand with low nanomolar potency. The inverted sulfonamides (**36, 37**) and the shorter benzenesulfonamides (**38, 39**) were less potent.

Within this strict SAR, the evaluation of structural extension from the aniline thus highlighted the phenylacetamide **16** and particularly the phenylmethanesulfonamide **35** as the most potent TLX agonist scaffolds for fine-grained optimization. We next continued structural tuning of **16** and studied the introduction of substituents on the phenylacetamide (Table 3). As the incorporation of a cyclopropane in the acetamide linker (**30**) was detrimental, we focused on substituents on the phenyl motif.

Scanning of methyl groups in all positions of the phenyl substituent (**40**-**42**) revealed no major activity difference and no improvement over **16** but a slight preference for 2-substitution (**40**). Fluorine (**43**), chlorine (**44**) and a methoxy group (**45**) were tolerated as alternative residues in 2-position, with fluorine (**43**) being favored in terms of efficacy but no modification provided an improvement in TLX agonist activity. Based on the preference for fluoro substitution in 2-position we tested the 2,6-difluoro analogue (**46**, Table 4) which indeed enhanced TLX agonist potency to a double-digit nanomolar EC_50_ value. The isomeric 2,3- (**47**), 2,4- (**48**) and 2,5- (**49**) difluoro derivatives were less favored. Evaluation of alternative, asymmetric 2,6-disubstitution patterns (**50**-**53**) revealed a distinctive SAR. Combined fluoro and chloro substitution (**50**) diminished potency compared to the difluoro analogue **46** while the 2-fluoro-6-trifluoromethyl (**51**) and 2-fluoro-6-methoxy (**52**) derivatives were equipotent to the 2,6-difluoro analogue **46**. Combining these observations in a 2-fluoro-6-difluoromethoxy substitution pattern (**53**) was strongly favored and displayed an EC_50_ of 15 nM for TLX activation. With a 100-fold increase in activity over the fragment lead **8, 53** emerged as highly potent TLX agonist and appeared suitable for in-depth evaluation as chemical tool.

Using the SAR insights from **40**-**53**, we moved our attention to the phenylmethanesulfonamide **35** and evaluated transfer of the favored substitution patterns (Table 5). As observed for **43**, installation of a 2-fluoro substituent on the phenyl motif (**54**) was tolerated, but double fluorine substitution (**55**), which was favored in the phenylacetamide series (**46**), markedly decreased TLX agonism. Introduction of a bulkier trifluoromethyl motif in the 2-position (**56**) was detrimental, overall highlighting the unsubstituted phenylmethanesulfonamide **35** as favored. SAR transfer between the scaffolds of **16** and **35** was hence not productive which underscored the chemical diversity of both TLX agonists as valuable feature for their application as tools.

Our previous efforts in optimization of the fragment screening hit **5**^25^ had also yielded the moderately potent inverse agonist **57** (Table 6). Based on the remarkable improvement in TLX agonist potency that could be achieved via extension from the aniline motif (e.g., in **35** and **53**), we explored whether this strategy could also boost inverse agonism (Table 6). Introduction of the 2-fluoro-6-difluoromethoxyphenylbenzamide motif of **53** to the inverse agonist scaffold of **57** was not tolerated (**58**), however, and even a small acetamide motif (**59**) disrupted inverse agonism and switched the profile to weak agonist activity. These results suggested that inverse TLX agonists did not follow a similar SAR pattern in this region and required individual systematic optimization.

**Figure 2:**
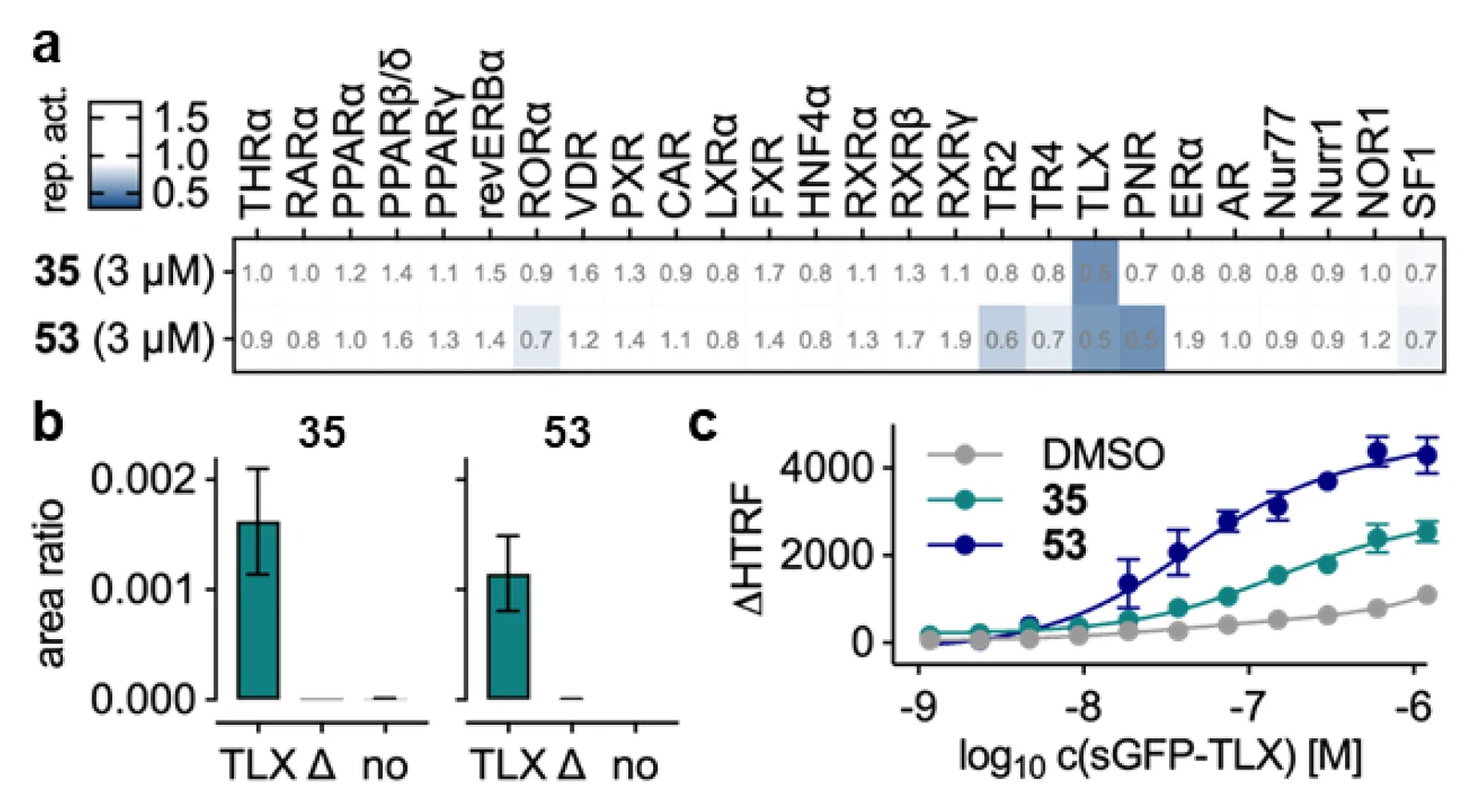
In vitro characterization of the TLX agonists **35** and **53**. (a) Selectivity profiling in uniform Gal4-hybrid reporter gene assays for a representative panel of nuclear receptors. **35** was selective for TLX modulation at 3 µM (selectivity ≥ 30-fold), while **53** also modulated the activity of the TLX-related receptors TR2, TR4 and PNR in the NR2 family. The heatmap shows the mean reporter activity relative to DMSO treated cells; n≥3. (b) Binding of **35** and **53** to the TLX LBD was confirmed in affinity-selection-mass-spectrometry (ASMS) experiments. Both compounds eluted with the recombinant TLX LBD in size-exclusion chromatography but were hardly detectable with heat-inactivated TLX LBD (Δ) and in absence of TLX (no). Data are the mean±SD; n=7 (TLX), n=3 (Δ, no). (c) **35** and **53** promoted dimerization of the TLX LBD in homogenous time-resolved fluorescence resonance energy transfer (HTRF) based assays. A fixed concentration of Tb^3+^-cryptate labeled TLX LBD was treated with increasing concentrations of sGFP-labeled TLX LBD in absence (DMSO ctrl) or presence of the TLX ligands (10 µM). The total sGFP content was kept constant by addition of free sGFP. Data are mean±SD ΔHTRF, n=3.

**35** and **53** emerged from the fragment extension approach with double digit nanomolar TLX agonist potency for further exploration and in-depth characterization. Both compounds displayed favorable selectivity for TLX in the nuclear receptor family (Figure 2a) with no relevant activity at 3 µM in a representative panel of NR1, NR3, NR4 and NR5 receptors. **35** retained selectivity also within the NR2 family, while **53** affected the activity of TR2, TR4 and PNR closely related to TLX. A slight off-target liability on RORα, which we have detected in previous studies on this TLX ligand chemotype^32^, was not relevant for **35** and **53** with >30-fold selectivity for TLX over RORα (Table 7, Figure S1). Additionally, in vitro metabolic stability evaluation using human liver microsomes (HLM) revealed a half-life >100 min for both **35** and **53** indicating potential suitability for in vivo evaluation (Figure S2, Table 7).

Affinity-selection-mass-spectrometry (ASMS) experiments confirmed binding of **35** and **53** to the recombinant TLX LBD (Figure 2b) validating TLX mediated effects. For preliminary mechanistic insights, we studied the compounds’ effects on TLX dimer formation, which has been found responsive to ligand binding^29^. In a homogenous time-resolved fluorescence resonance energy transfer (HTRF) based setting using Tb^3+^-cryptate labeled TLX LBD and sGFP-labeled TLX LBD, **35** and **53** promoted TLX dimerization (Figure 2c). In line with the mechanism of the nuclear receptor revERB (NR1D), which forms homodimers binding to the RORE to act as a repressor^33,34^, these results suggest that activation of TLX’ repressor activity by ligands involves stabilization of a TLX dimer.

**Figure 3:**
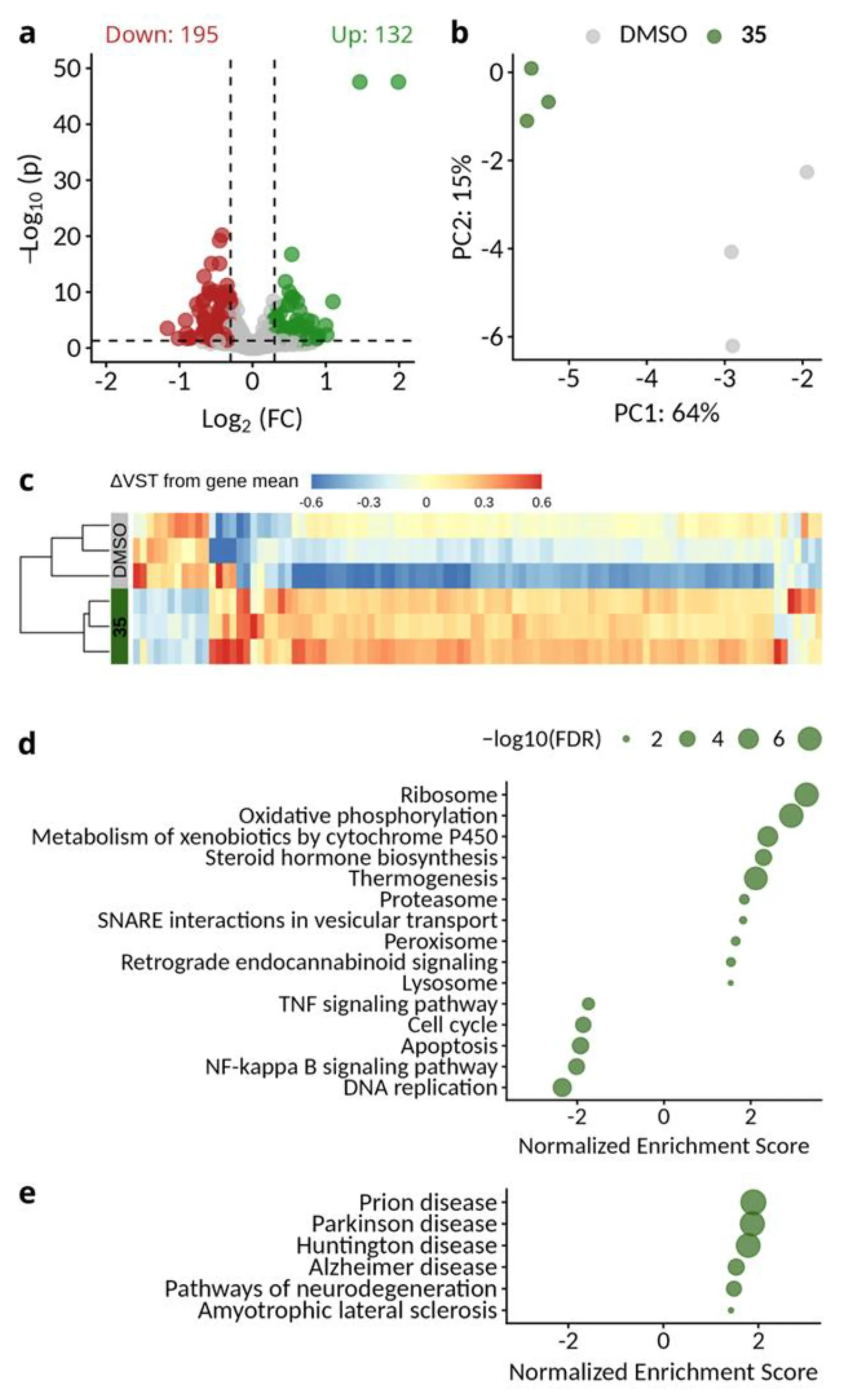
Transcriptomic effects of **35** in retinal pigment epithelial cells (ARPE-19). Cells were treated for 16 h with 1 µM **35**; n=3. (a) Volcano plot of genes regulated by **35**. (b) Principal component analysis (PCA) of the transcriptomic profiles of cells treated with **35** or DMSO ctrl. (c) Correlation analysis of gene expression profiles in cells treated with **35** or DMSO ctrl. The heatmap shows the top-100 regulated genes. (d) Gene set enrichment analysis (GSEA) on KEGG pathways. (e) GSEA on pathology-related KEGG pathways. Selected pathways are shown in Figures S3-4.

With nanomolar TLX agonist potency, >30-fold selectivity, and validated binding, **35** appeared suitable as tool to study the transcriptomic impact of activating TLX’ repressor function. Therefore, we performed RNA sequencing (RNAseq) in TLX expressing retinal pigment epithelial cells (ARPE-19) after treatment with **35** (1 µM) for 16 h to detect direct effects. **35** modulated the expression of 327 genes (padj ≤ 0.05, log2foldchange ≥ |0.3|; Figure 3a) and caused uniform effects over the samples according to principal component (PCA, Figure 3b) and correlation analysis (Figure 3c).

Gene Set Enrichment Analysis (GSEA; Figure 3d) highlighted upregulation of cellular respiration, oxidative phosphorylation and ATP production as prominent transcriptomic outcomes. These effects centered mainly on components of the respiratory chain complexes I (NADH:ubiquinone oxidoreductase) and V (ATP synthase). Additionally, **35** induced genes related to SNAREs and vesicular transport (e.g., synaptosome-associated protein of 23 kDa (SNAP23)) as well as lysosomal components (e.g., vacuolar-type H^+^-adenosine triphosphatase (V-ATPase), biogenesis of lysosome-related organelles complex-1 (BLOC1), acid ceramidase (ASAH1)). Overall, however, **35** mainly caused suppression of gene expression in line with its repressor-activating mode-of-action. Inflammatory signaling pathways (TNF, NFκB), apoptosis signaling, as well as cell cycle and DNA replication genes were downregulated. These transcriptomic effects of **35** pointed to a protective, energy-storing state upon activation of TLX’ repressor activity.

GSEA on pathology-related pathways linked the transcriptomic effects of **35** to several neurodegenerative diseases. Therein, the favorable upregulation of respiratory chain components, was accompanied by direct transcriptomic effects on prominent neurodegeneration-associated genes: For example, β-secretase (BACE), an amyloid precursor protein processing enzyme producing amyloid-β, was downregulated, while the PTEN-induced kinase 1 (PINK1), a key mitochondrial quality control factor, and LC3 genes, which are related to autophagy, were induced. Altogether, the transcriptomic effects of **35** add another clue for therapeutic potential of TLX modulation in neurodegeneration.

## Conclusion

Several lines of evidence from preclinical studies designate the peculiar nuclear receptor TLX as master regulator of neuronal stem cell homeostasis and as promising target in degenerative diseases. However, the effects of pharmacological TLX modulation remain elusive due to the lack of high-quality ligands. Here, we developed a potent and selective activator of TLX’ repressor function (**35**) as chemical tool and explored its transcriptomic impact in RNAseq. **35** stimulated genes associated with ATP production, lysosome function, and vesicular transport while suppressing inflammatory and apoptotic pathways. Additionally, **35** affected the expression of prominent genes linked to neurodegenerative pathologies such as BACE and PINK1. These transcriptomic effects of activation of TLX’ repressor function further support the receptor’s potential as a target in degenerative diseases.

### Chemistry

**Scheme 1:**
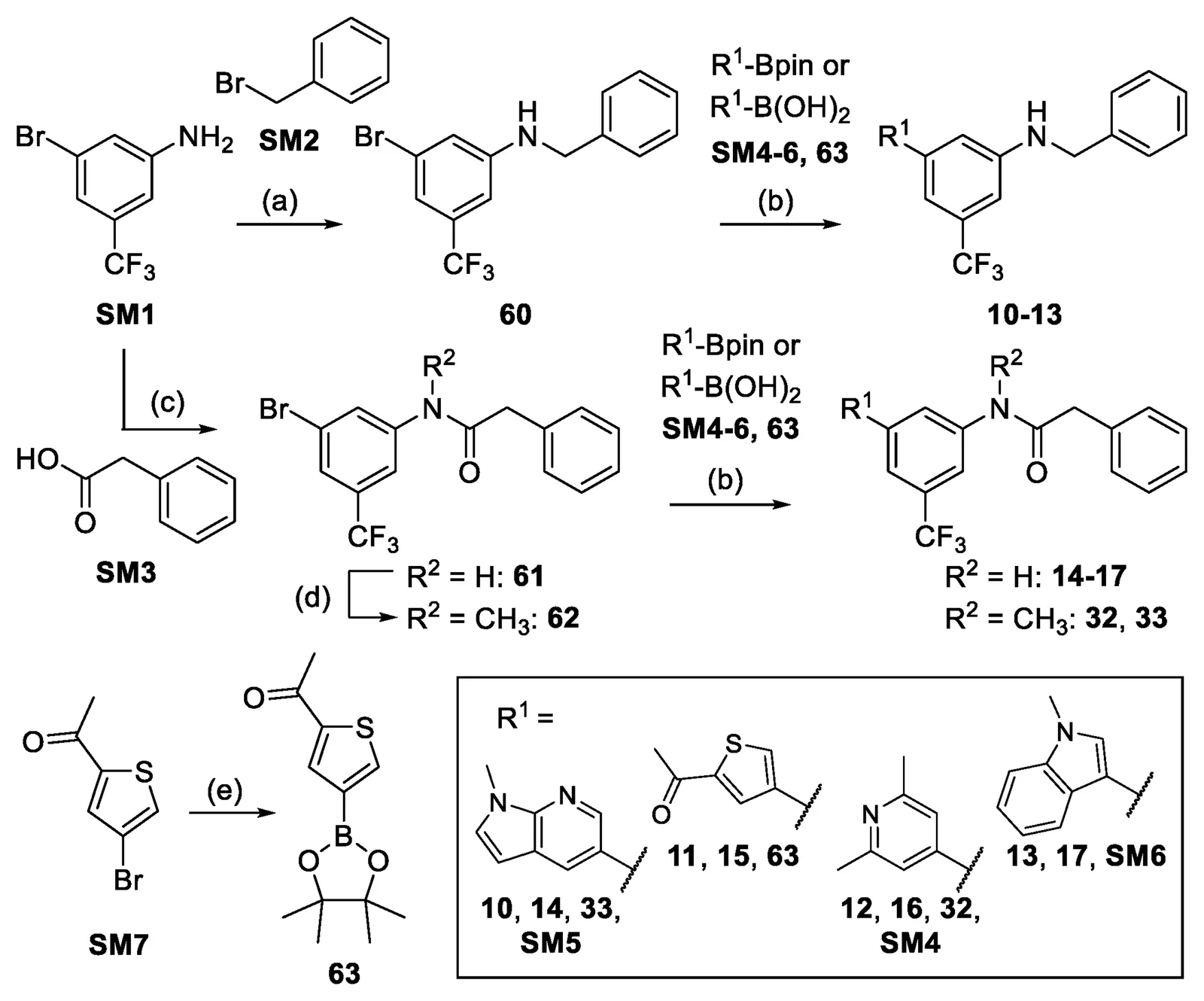
Synthesis o 10-17, 32 and 33 ^a^. ^a^ Reagents & Conditions: (a) K_2_CO_3_ (1.5 eq.), DMF, 90 °C, 20 h, 52%; (b) XPhosPdG3 (10 mol%), K_3_PO_4_, dioxane/H_2_O, 100 °C, overnight, 46-87%; (c) TCFH, NMI, DMF, 80 °C, overnight, 69%; (d) MeI (2 eq.), NaH (1.5 eq.), DMF, 0°C to rt, 6 h, 27%; (e) B_2_Pin_2_ (1.1 eq.), Pd(dppf)Cl_2_ (2.5 mol%), KOAc (3 eq.), dry dioxane, N_2_, 90 °C, overnight, 41%. Starting materials labeled with SM numbers were commercially available.

Compounds **10-17** (Scheme 1) were prepared by Suzuki-Miyaura reaction of the 3-bromo-5-(trifluoromethyl)aniline derivatives **60**-**62**. The benzyl amine **60** was generated by reaction of **SM1** with benzyl bromide (**SM2**), the phenylacetamide intermediate **61** was obtained from TCFH/NMI-mediated coupling^35^ of **SM1** with phenylacetic acid (**SM3**), and the precursor **62** of the *N*-methyl derivatives **32** and **33** was prepared by methylation of **61** with MeI. Suzuki-Miyaura couplings were performed with commercially available boronic acids (**SM4**) or Bpin esters (**SM5, SM6**) and with the Bpin ester **63**, which was prepared by borylation of 2-acetyl-4-bromothiophene (**SM7**).

**Scheme 2:**
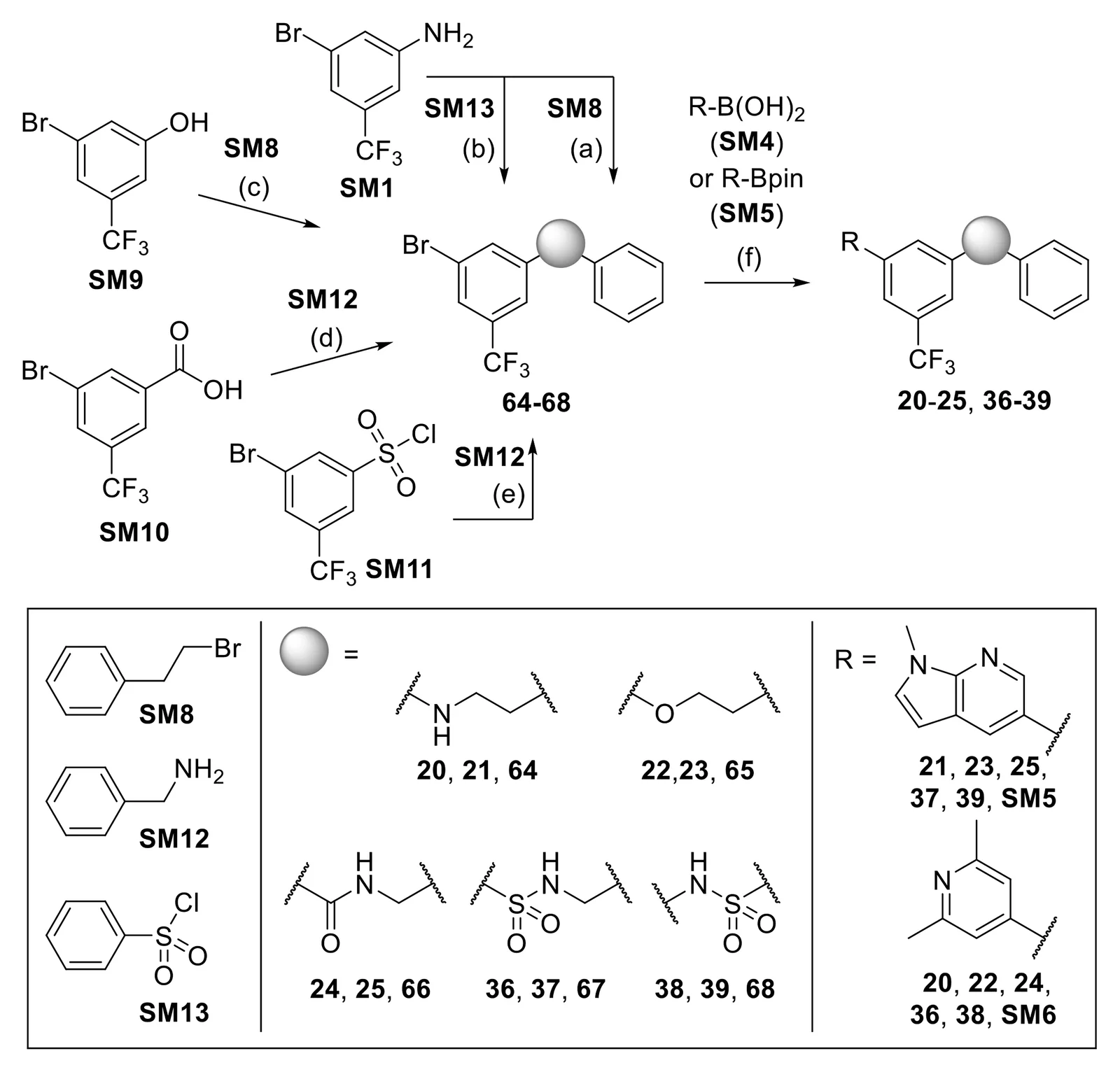
Synthesis o20-25 and 36-39. ^a^ Reagents & Conditions: (a) K_2_CO_3_ (1.5 eq.), DMF, 90°C, 20 h, 15%; (b) pyridine (3 eq.), CH_2_Cl_2_, 0 °C to rt, 3 h, 24%; (c) K_2_CO_3_ (2 eq.), ACN, 80 °C, 16 h, 40%; (d) TCFH, NMI, DMF, 80 °C, overnight, 70%; (e) DIPEA (3 eq.), CH_2_Cl_2_, 0 °C to rt, overnight, 58%. (f) XPhosPdG3 (10 mol%), K_3_PO_4_, dioxane/H_2_O, 100 °C, overnight, 25-89%. Starting materials labeled with SM numbers were commercially available.

Similarly, compounds **20-25** and **36-39** were prepared from the respective aryl bromides **64-68** by Suzuki-Miyaura coupling with (2,6-dimethylpyridin-4-yl)boronic acid (**SM4**) or (1-methyl-1*H*-pyrrolo[2,3-*b*]pyridin-5-yl)boronic acid pinacol ester (**SM5**, Scheme 2). The required precursors **64** and **65** were generated by nucleophilic substitution of 2-phenylethylbromid (**SM8**) with **SM1** or 3-bromo-5-trifluoromethylphenol (**SM9**). **66** and **67** were obtained by reaction of 3-bromo-5- (trifluoromethyl)benzoic acid (**SM10**) or 3-bromo-5- (trifluoromethyl)benzenesulphonyl chloride (**SM11**) with benzylamine (**SM12**). **68** was available from the reaction of **SM1** with benzenesulfonyl chloride (**SM13**).

**Scheme 3:**
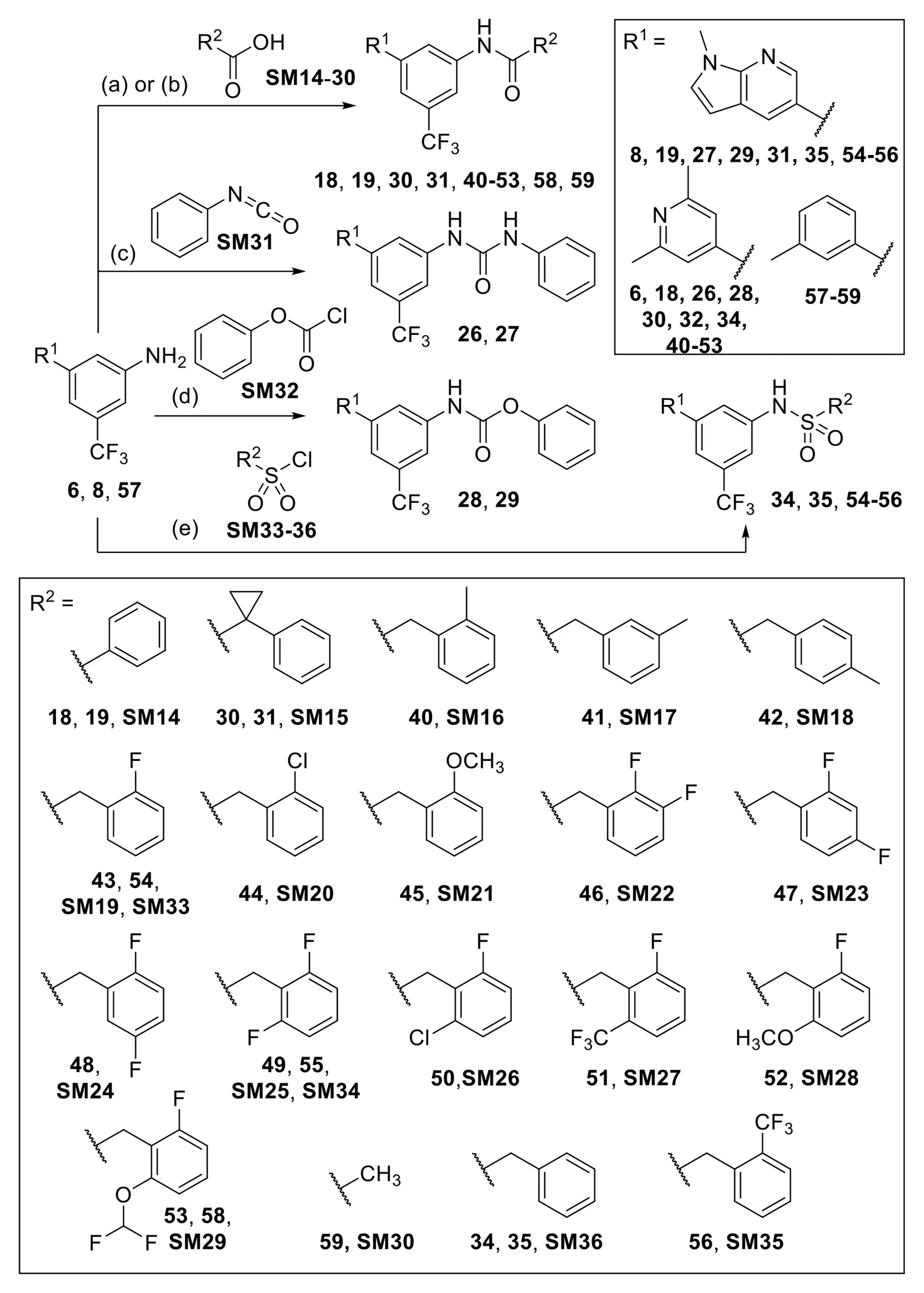
Synthesis of 18, 19, 26-31, 34, 35, 40-56, 58, 59. ^a^ ^a^ Reagents & Conditions: (a) SOCl_2_, reflux, 2 h, then addition of the aniline, CHCl_3_, 0 °C to rt, overnight, 51-72%; (b) TCFH, NMI, DMF, 80 °C, overnight, 28-67%; (c) TEA (2.3 eq.), CH_2_Cl_2_, 0 °C to rt, overnight, 9-36%; (d) TEA (2 eq.), CH_2_Cl_2_, 0 °C to rt, overnight, 3-45%; (e) DIPEA (3 eq.), CH_2_Cl_2_, 0 °C to rt, overnight, 14-60%. Preparation of **6, 8** and **57** has been described previously^25^. Starting materials labeled with SM numbers were commercially available.

Compounds **18, 19, 26**-**31, 34, 35, 40**-**56, 58** and **59** were synthesized from the respective aniline fragments **6, 8** and **57** (Scheme 3), whose preparation was described previously^25^. To obtain the amides **30, 31, 40-53** and **58**, the respective carboxylic acids (**SM15-SM29**) were used for coupling with **6, 8** or **57** using SOCl_2_ or TCFH/NMI. **18, 19** and **60** were prepared by reacting **6** or **8** with benzoyl chloride (**SM14**) and **57** with acetyl chloride (**SM30**). The urea derivatives **26** and **27** were obtained by treating aniline **6** or **8** with phenyl isocyanate (**SM31**), while the carbamates **28** and **29** were formed by reaction of **6** or **8** with phenyl chloroformate (**SM32**). The sulfonamides **34, 35** and **54-56** were generated by coupling **6** or **8** with the sulfonyl chlorides **SM33-SM36**.

### Experimental Procedures

#### Chemistry

##### General

All chemicals were of reagent grade, purchased from commercial sources (e.g., Sigma-Aldrich, TCI, BLDpharm) and used without further purification unless otherwise specified. All reactions were conducted under nitrogen (N_2_) or argon atmosphere and in absolute solvents purchased from Sigma-Aldrich. Other solvents, especially for work-up procedures, were of reagent grade or purified by distillation (iso-hexane, cyclohexane, EtOAc, EtOH). Reactions were monitored by thin layer chromatography (TLC) on TLC Silica gel 60 F254 coated aluminum sheets by Merck and visualized under ultraviolet light (254 nm). Purification by column chromatography was performed on a puriFlash® XS520Plus system (Advion, Ithaca, NY, USA) using high performance spherical silica columns (SIHP, 30 or 50 µm) by Interchim and a gradient of iso-hexane or cyclohexane to EtOAc, reversed-phase (RP) column chromatography was performed on a puriFlash® 5.250 system (Advion) using C18HP columns (SIHP, 15 µm) by Interchim and a gradient of H_2_O with 10% MeCN to 100% MeCN (HPLC gradient grade). Melting points (mp) were determined with a Krüss M3000 melting point meter (A. Krüss Optronic GmbH, Hamburg, Germany). Mass spectra were obtained on a puriFlash®-CMS system (Advion) using atmospheric pressure chemical ionization (APCI). High-resolution mass spectra (HRMS) were obtained with a Thermo Finnigan LTQ FT instrument for electron impact ionization (EI) or electrospray ionization (ESI). NMR spectra were recorded on Bruker Avance III HD 400 MHz or 500 MHz spectrometers equipped with a CryoProbeTM Prodigy broadband probe (Bruker). Chemical shifts are reported in δ values (ppm) relative to residual protium signals in the NMR solvent (^1^H-NMR: acetone-*d*_*6*_: δ = 2.05 ppm; DMSO-*d*_*6*_: δ = 2.50 ppm; methanol-*d*_*4*_: δ = 3.31 ppm; CD_2_Cl_2_: δ = 5.32 ppm, ^13^C-NMR: acetone-*d*_*6*_: δ = 206.26, 29.84 ppm; DMSO-*d*_*6*_: δ = 39.52 ppm; methanol-*d*_*4*_:δ = 49.0 ppm; CD_2_Cl_2_: δ = 53.84 ppm) and coupling constants (*J*) in hertz (Hz). The splitting of signals is indicated by s for singlet, d for doublet, t for triplet, q for quartet, dd for doublet of a doublet and m for multiplet. The purity of the compounds was determined by ^1^H NMR (qHNMR) according to the method described by Pauli et al.^36^ with internal calibration. To ensure accurate determination of peak area ratio, the qHNMR measurements were conducted under conditions allowing for complete relaxation. Maleic acid (LOT#BCCH2407, purity 99.85%) was used as internal standard in methanol-*d*_*4*_ or DMSO-*d*_*6*_. All compounds for biological testing had a purity >95% according to quantitative ^1^H NMR (qHNMR).

The synthesis of compounds **6-9** and **57** has been reported previously^25^.

##### General procedure A (Suzuki-coupling)

A 50-100 mL Schlenk flask was charged with a stir bar, the boronic acid/pinacol ester (1 eq.) and K_3_PO_4_ (3 eq.). The flask was evacuated and backfilled with nitrogen three times. The previously de-gassed (3 freeze thaw-cycles) solvent mixture (dioxane/water = 85:15, ~0.05M), together with the Pd catalyst (10 mol%) and the heteroaryl bromide (1.5-2 eq.) was added and the reaction mixture was stirred at 100°C under reflux and nitrogen atmosphere. After TLC indicated completion of the reaction, the solution was cooled down to room temperature (rt) and the solvents were evaporated. The residue was taken up in EtOAc and washed with water and brine. The organic layer was dried over Na_2_SO_4_, concentrated *in vacuo*, and the product was purified by flash column chromatography.

##### General procedure B (amide coupling with SOCl_2_)

The carboxylic acid (1 eq.) was dissolved in SOCl_2_ (1-3 mL) and heated to reflux for 2-2.5 h. The mixture was cooled down to rt and the solvent was evaporated. To the prepared acyl chloride was added CHCl_3_ (~0.1 M), and the amine (1 – 2 eq.) at 0 °C. The reaction mixture was flushed with N_2_, allowed to warm up to rt and stirred for 12-72 h. After TLC indicated completion of the reaction, the solvent was evaporated and the residue was acidified with 5% aq. HCl. The mixture was extracted with EtOAc (3x), the combined organic layers were washed with 1 M NaOH, water and brine, dried over Na_2_SO_4_ and concentrated *in vacuo*. The crude product was purified by flash column chromatography and if necessary, by RP column chromatography to yield the desired amides.

##### General procedure C (amide coupling with TCFH)

The carboxylic acid (1-1.5 eq.), the amine (1-2 eq.) and *N*-methylimidazole (NMI) (2 eq.) were dissolved in ACN or DMF (~0.03M) and TCFH (1.2-1.5 eq.) was added under stirring. The mixture was heated to 80 °C for 16-24 h. After TLC indicated completion of the reaction, the solvent was evaporated, and the residue was acidified with 5% aq. HCl. The mixture was extracted with EtOAc (3x), the combined organic layers were washed with 1 M NaOH, water and brine, dried over Na_2_SO_4_ and concentrated *in vacuo*. The crude product was purified by flash column chromatography and if necessary, by RP column chromatography to yield the desired amides.

##### General procedure D (Sulfonamide coupling)

The amine (1-2 eq.) was dissolved in dry CH_2_Cl_2_ (~0.1M) and stirred at 0 °C. The sulfonyl chloride (1-2 eq.) and DIPEA (3 eq.) were added, the mixture was allowed to warm up to rt, stirred overnight, and then concentrated *in vacuo*. The residue was taken up in EtOAc and washed with 5% aq. HCl and brine, dried over Na_2_SO_4_ and concentrated *in vacuo*. The crude product was purified by flash column and if necessary, by RP column chromatography to yield the desired sulfonamides.

##### N-Benzyl-3-(1-methyl-1H-pyrrolo[2,3-b]pyridin-5-yl)-5-(trifluoromethyl)aniline (10)

Preparation according to general procedure A from **60** (133 mg, 0.40 mmol, 1.0 eq.) and 1-methyl-5-(4,4,5,5-tetramethyl-1,3,2-dioxaborolan-2-yl)-1*H*-pyrrolo[2,3-b]pyridine (**SM5**, 127 mg, 0.47 mmol, 1.2 eq.). Further purification by RP column chromatography yielded a colorless oil (134 mg, 87%). ^1^H NMR (400 MHz, methanol-*d*_*4*_) δ 8.33 (d, *J* = 2.1 Hz, 1H), 8.04 (d, *J* = 2.2 Hz, 1H), 7.43 – 7.35 (m, 2H), 7.34 – 7.28 (m, 3H), 7.26 – 7.17 (m, 1H), 7.06 – 7.02 (m, 1H), 7.01 – 6.96 (m, 1H), 6.86 – 6.80 (m, 1H), 6.48 (d, *J* = 3.5 Hz, 1H), 4.38 (s, 2H), 3.83 (s, 3H). ^13^C NMR (101 MHz, methanol-*d*_*4*_) δ 151.03, 148.35, 142.24, 142.08, 140.70, 132.97 (q, *J* = 31.4 Hz), 131.93, 129.92, 129.57, 128.66, 128.34, 128.07, 125.93 (q, *J* = 271.8 Hz), 122.57, 115.42, 112.44 (q, *J* = 4.3 Hz), 108.66 (q, *J* = 4.2 Hz), 100.88, 48.32, 31.61. HRMS (GC/EI+): *m/z* calculated 381.1447 for C_22_H_18_F_3_N_3_, found 381.1458 ([M]^·+^). qHNMR (400 MHz, DMSO-*d*_6_) purity = 96.8%.

##### 1-(4-(3-(Benzylamino)-5-(trifluoromethyl)phenyl)thiophen-2-yl)ethan-1-one (11)

Preparation according to general procedure A from **60** (97 mg, 0.29 mmol, 1.0 eq.) and **63** (74 mg, 0.29 mmol, 1.0 eq.). Further purification by RP column chromatography yielded a pale brown solid (95 mg, 86%). Mp 118 °C. ^1^H NMR (400 MHz, DMSO -*d*_*6*_) δ 8.34 (d, *J* = 1.5 Hz, 1H), 8.30 (d, *J* = 1.5 Hz, 1H), 7.44 – 7.38 (m, 2H), 7.38 – 7.31 (m, 2H), 7.28 – 7.18 (m, 3H), 6.88 – 6.85 (m, 1H), 6.80 (t, *J* = 5.9 Hz, 1H), 4.41 (d, *J* = 5.9 Hz, 2H), 2.60 (s, 3H). ^13^C NMR (101 MHz, DMSO-*d*_*6*_) δ 191.08, 149.65, 144.77, 141.81, 139.43, 135.83, 132.41, 130.53 (q, *J* = 31.3 Hz), 130.03, 128.42, 127.43, 126.91, 123.07 (q, *J* = 273.1 Hz), 112.45, 109.69 (q, *J* = 3.3 Hz), 108.00 (q, *J* = 4.2 Hz), 46.24, 26.70. HRMS (GC/EI+): *m/z* calculated 375.0899 for C_20_H_16_F_3_NOS, found 375.0899 ([M]^·+^). qHNMR (400 MHz, DMSO-*d*_6_) purity = 96.3%.

##### N-Benzyl-3-(2,6-dimethylpyridin-4-yl)-5-(trifluoromethyl)aniline (12)

Preparation according to general procedure A from **60** (111 mg, 0.34 mmol, 1.0 eq.) and (2,6-dimethylpyridin-4-yl)boronic acid (**SM4**; 68 mg, 0.44 mmol, 1.3 eq.). Further purification by RP column chromatography yielded a pale-yellow solid (95 mg, 79%). Mp 133 °C. ^1^H NMR (400 MHz, DMSO-*d*_*6*_) δ 7.42 – 7.31 (m, 4H), 7.29 – 7.22 (m, 3H), 7.16 – 7.13 (m, 1H), 7.12 – 7.10 (m, 1H), 6.95 – 6.92 (m, 1H), 6.89 (t, *J* = 5.9 Hz, 1H), 4.41 (d, *J* = 5.9 Hz, 2H), 2.46 (s, 6H). ^13^C NMR (101 MHz, DMSO-*d*_*6*_) δ 157.91, 149.74, 147.19, 139.63, 139.34, 130.61 (q, *J* = 31.0 Hz), 128.46, 127.39, 126.95, 124.45 (q, *J* = 273.4 Hz), 117.74, 113.51, 110.07 (q, *J* = 3.6 Hz), 108.80 (q, *J* = 3.1 Hz), 46.24, 24.02. HRMS (DEP/EI+): *m/z* calculated 356.1495 for C_21_H_19_F_3_N_2_, found 356.1494 ([M]^·+^). qHNMR (400 MHz, DMSO-*d*_6_) purity = 99.2%.

##### N-Benzyl-3-(1-methyl-1H-indol-3-yl)-5-(trifluoromethyl)aniline (13)

Preparation according to general procedure A from **60** (102 mg, 0.31 mmol, 1.0 eq.) and 1-methyl-3-(4,4,5,5-tetramethyl-1,3,2-dioxaborolan-2-yl)-1*H*-indole (**SM6**, 93 mg, 0.35 mmol, 1.1 eq.). Further purification by RP column chromatography yielded a pale-yellow oil (96 mg, 82%). ^1^H NMR (400 MHz, methanol-*d*_*4*_) δ 7.51 – 7.44 (m, 1H), 7.40 – 7.21 (m, 7H), 7.20 – 7.13 (m, 1H), 7.06 (s, 1H), 7.04 – 6.97 (m, 2H), 6.77 – 6.72 (m, 1H), 4.36 (s, 2H), 3.72 (s, 3H). ^13^C NMR (101 MHz, methanol-*d*_*4*_) δ 150.64, 140.94, 139.02, 138.72, 132.58 (q, *J* = 31.1 Hz), 129.60, 128.19, 128.17, 127.95, 127.13, 126.13 (q, *J* = 271.6 Hz), 122.85, 120.93, 120.43, 116.77, 114.94, 112.39 (q, *J* = 3.6 Hz), 110.61, 107.58 (q, *J* = 3.4 Hz), 32.85, 32.82. HRMS (GC/EI+): *m/z* calculated 380.1495 for C_23_H_19_F_3_N_2_, found 380.1494 ([M]^·+^). qHNMR (400 MHz, DMSO-*d*_6_) purity = 96.8%.

##### N-(3-(1-Methyl-1H-pyrrolo[2,3-b]pyridin-5-yl)-5-(trifluoromethyl)phenyl)-2-phenylacetamide (14)

Preparation according to general procedure A from **61** (144 mg, 0.40 mmol, 1.0 eq.) and 1-methyl-5-(4,4,5,5-tetramethyl-1,3,2-dioxaborolan-2-yl)-1*H*-pyrrolo[2,3-b]pyridine (**SM5**, 120 mg, 0.44 mmol, 1.1 eq.). Further purification by RP column chromatography yielded a colorless solid (125 mg, 76%). Mp 155 °C. ^1^H NMR (400 MHz, DMSO-*d*_6_) δ 10.62 (s, 1H), 8.56 (d, *J* = 2.2 Hz, 1H), 8.26 (d, *J* = 2.2 Hz, 1H), 8.12 (s, 1H), 8.07 (s, 1H), 7.70 (s, 1H), 7.59 (d, *J* = 3.4 Hz, 1H), 7.42 – 7.30 (m, 4H), 7.29 – 7.22 (m, 1H), 6.55 (d, *J* = 3.4 Hz, 1H), 3.86 (s, 3H), 3.71 (s, 2H). ^13^C NMR (101 MHz, DMSO-*d*_6_) δ 169.89, 147.34, 141.21, 140.87, 140.56, 135.52, 131.30, 130.30 (q, *J* = 31.2 Hz), 129.22, 128.36, 126.86, 126.68, 126.66, 124.08 (q, *J* = 272.2 Hz), 120.82, 120.01, 118.14 – 117.73 (m), 113.76 – 113.40 (m), 99.43, 43.38, 30.99. HRMS (DEP/EI+): *m/z* calculated 409.1396 for C_23_H_18_F_3_N_3_O, found 409.1376 ([M]^·+^). qHNMR (400 MHz, DMSO-*d*_6_) purity = 95.6%.

##### N-(3-(5-Acetylthiophen-3-yl)-5-(trifluoromethyl)phenyl)-2-phenylacetamide (15)

Preparation according to general procedure A from **61** (154 mg, 0.43 mmol, 1.0 eq.) and **63** (107 mg, 0.42 mmol, 1.0 eq.). Further purification by RP column chromatography yielded a colorless solid (148 mg, 85%). Mp 182 °C. ^1^H NMR (400 MHz, DMSO-*d*_6_) δ 10.60 (s, 1H), 8.41 (d, *J* = 1.5 Hz, 1H), 8.37 (d, *J* = 1.5 Hz, 1H), 8.14 (s, 1H), 8.04 (s, 1H), 7.83 (s, 1H), 7.40 – 7.31 (m, 4H), 7.30 – 7.21 (m, 1H), 3.70 (s, 2H), 2.62 (s, 3H). ^13^C NMR (101 MHz, DMSO-*d*_6_) δ 191.09, 169.88, 145.13, 140.72, 140.54, 135.94, 135.48, 132.26, 130.75, 130.36 (q, *J* = 31.8 Hz), 129.22, 128.37, 126.70, 124.00 (d, *J* = 272.9 Hz), 119.93, 117.47 (q, *J* = 4.0 Hz), 114.41 (q, *J* = 4.1 Hz), 43.34, 26.72. HRMS (DEP/EI+): *m/z* calculated 403.0848 for C_21_H_16_F_3_NO_2_S, found 403.0841 ([M]^·+^). qHNMR (400 MHz, DMSO-*d*_6_) purity = 95.0%.

##### N-(3-(2,6-Dimethylpyridin-4-yl)-5-(trifluoromethyl)phenyl)-2-phenylacetamide (16)

Preparation according to general procedure A from **61** (108 mg, 0.30 mmol, 1.0 eq.) and (2,6-dimethylpyridin-4-yl)boronic acid (**SM4**, 61 mg, 0.39 mmol, 1.3 eq.). Further purification by RP column chromatography yielded a colorless solid (93 mg, 80%). Mp 101 °C. ^1^H NMR (400 MHz, DMSO-*d*_*6*_) δ 10.65 (s, 1H), 8.21 – 8.16 (m, 1H), 8.14 – 8.09 (m, 1H), 7.76 – 7.71 (m, 1H), 7.40 – 7.30 (m, 6H), 7.30 – 7.23 (m, 1H), 3.70 (s, 2H), 2.49 (s, 6H). ^13^C NMR (101 MHz, DMSO-*d*_*6*_) δ 169.93, 158.16, 146.11, 140.68, 139.66, 135.42, 130.40 (q, *J* = 31.6 Hz), 129.22, 128.36, 126.69, 123.88 (q, *J* = 272.4 Hz), 120.70, 117.87 (q, *J* = 3.8 Hz), 117.73, 115.36 (q, *J* = 4.2 Hz), 43.35, 24.00. HRMS (GC/EI+): *m/z* calculated 384.1444 for C_22_H_19_F_3_N_2_O, found 384.1443 ([M]^·+^). qHNMR (400 MHz, DMSO-*d*_6_) purity = 96.0%.

##### N-(3-(1-Methyl-1H-indol-3-yl)-5-(trifluoromethyl)phenyl)-2-phenylacetamide (17)

Preparation according to general procedure A from **61** (110 mg, 0.31 mmol, 1.0 eq.) and 1-methyl-3-(4,4,5,5-tetramethyl-1,3,2-dioxaborolan-2-yl)-1*H*-indole (**SM6**, 92 mg, 0.34 mmol, 1.1 eq.). Further purification by RP column chromatography yielded a colorless solid (87 mg, 69%). Mp 156 °C. ^1^H NMR (400 MHz, DMSO-*d*_6_) δ 10.58 (s, 1H), 8.25 (s, 1H), 7.97 – 7.89 (m, 3H), 7.61 (s, 1H), 7.53 (d, *J* = 8.1 Hz, 1H), 7.41 – 7.31 (m, 4H), 7.30 – 7.16 (m, 3H), 3.85 (s, 3H), 3.72 (s, 2H). ^13^C NMR (101 MHz, DMSO-*d*_6_) δ 169.78, 140.49, 137.37, 137.22, 135.60, 130.07 (q, *J* = 31.4 Hz), 129.21, 128.92, 128.36, 126.64, 125.57 (q, *J* = 272.4 Hz), 124.88, 121.88, 120.28, 119.54, 118.91, 116.89 (q, *J* = 4.1 Hz), 112.96, 111.59 (q, *J* = 4.1 Hz), 110.56, 43.41, 32.67. HRMS (DEP/EI+): *m/z* calculated 408.1444 for C_24_H_19_F_3_N_2_O, found 408.1445 ([M]^·+^). qHNMR (400 MHz, DMSO-*d*_6_) purity = 97.4%.

##### N-(3-(2,6-Dimethylpyridin-4-yl)-5-(trifluoromethyl)phenyl)benzamide (18)

**8** (96 mg, 0.36 mmol, 1.0 eq.) was added to a stirring solution of benzoyl chloride (**SM14**, 50 µL, 0.43 mmol, 1.2 eq.) in CHCl_3_ (dry, 6 mL) at 0°C. The mixture was flushed with N_2_, allowed to warm up to rt and stirred overnight. The solvent was evaporated and the residue was taken up in aq. NaOH. The suspension was extracted with EtOAc and the combined organic layers were washed with brine, dried over Na_2_SO_4_ and concentrated *in vacuo*. The crude product was purified by RP column chromatography to yield a colorless solid (80 mg, 60%). Mp 206 °C. ^1^H NMR (400 MHz, DMSO-*d*_*6*_) δ 10.65 (s, 1H), 8.44 (s, 1H), 8.35 (s, 1H), 8.05 – 7.98 (m, 2H), 7.81 (s, 1H), 7.67 – 7.54 (m, 3H), 7.42 (s, 2H), 2.51 (s, 6H). ^13^C NMR (101 MHz, DMSO-*d*_*6*_) δ 165.97, 158.17, 146.15, 140.72, 139.47, 134.19, 132.08, 130.41 (q, *J* = 9.0 Hz), 128.54, 127.71, 124.00 (q, *J* = 272.5 Hz), 121.94, 118.22 (q, *J* = 3.5 Hz), 117.73, 116.54 (q, *J* = 4.0 Hz), 24.03. HRMS (GC/EI+): *m/z* calculated 370.1287 for C_21_H_17_F_3_N_2_O, found 370.1285 ([M]^·+^). qHNMR (400 MHz, DMSO-*d*_6_) purity = 95.8%.

##### N-(3-(1-Methyl-1H-pyrrolo[2,3-b]pyridin-5-yl)-5-(trifluoromethyl)phenyl)benzamide (19)

**6** (102 mg, 0.35 mmol, 1.0 eq.) was added to a stirring solution of benzoyl chloride (**SM14**, 50 µL, 0.43 mmol, 1.2 eq.) in CHCl_3_ (dry, 6 mL) at 0°C, The mixture was flushed with N_2_, allowed to warm up to rt and stirred overnight. The solvent was removed *in vacuo* and the residue was taken up in aq. NaOH. The suspension was extracted with EtOAc and the combined organic layers were washed with brine, dried over Na_2_SO_4_ and concentrated. The crude product was purified by RP column chromatography to yield a colorless solid (100 mg, 72%). Mp 185 °C. ^1^H NMR (400 MHz, DMSO-*d*_6_) δ 10.63 (s, 1H), 8.61 (d, *J* = 2.2 Hz, 1H), 8.43 – 8.37 (m, 1H), 8.31 (d, *J* = 2.2 Hz, 1H), 8.30 – 8.27 (m, 1H), 8.07 – 7.99 (m, 2H), 7.77 (s, 1H), 7.67 – 7.54 (m, 4H), 6.57 (d, *J* = 3.5 Hz, 1H), 3.87 (s, 3H). ^13^C NMR (101 MHz, DMSO-*d*_6_) δ 165.98, 147.35, 141.25, 140.69, 140.58, 134.35, 131.99, 131.30, 130.19 (q, *J* = 31.7 Hz), 128.52, 127.73, 126.83, 126.68, 124.16 (q, *J* = 272.3 Hz), 122.03, 120.01, 118.27 (q, *J* = 3.9 Hz), 114.71 (q, *J* = 3.9 Hz), 99.42, 31.01. HRMS (GC/EI+): *m/z* calculated 395.1240 for C_22_H_16_F_3_N_3_O, found 395.1245 ([M]^·+^). qHNMR (400 MHz, DMSO-*d*_6_) purity = 96.6%.

##### 3-(2,6-Dimethylpyridin-4-yl)-N-phenethyl-5-(trifluoromethyl)aniline (20)

Preparation according to general procedure A from **64** (100 mg, 0.29 mmol, 1.0 eq.) and (2,6-dimethylpyridin-4-yl)boronic acid (**SM4**, 63 mg, 0.41 mmol, 1.4 eq.). Further purification by RP column chromatography yielded a pale-yellow oil (54 mg, 50%). ^1^H NMR (400 MHz, DMSO-*d*_*6*_) δ 7.36 – 7.27 (m, 6H), 7.26 – 7.18 (m, 1H), 7.13 – 7.08 (m, 2H), 6.95 – 6.90 (m, 1H), 6.35 (t, *J* = 5.4 Hz, 1H), 3.42 – 3.37 (m, 2H), 2.88 (t, *J* = 7.3 Hz, 2H), 2.48 (s, 6H). ^13^C NMR (101 MHz, DMSO-*d*_*6*_) δ 157.89, 149.85, 147.24, 139.75, 139.62, 130.63 (q, *J* = 31.0 Hz), 128.80, 128.35, 126.15, 124.42 (q, *J* = 272.5 Hz), 117.78, 112.98, 110.03 – 109.62 (m), 108.99 – 108.20 (m), 44.28, 34.76, 24.01. HRMS (DEP/EI+): *m/z* calculated 370.1651 for C_22_H_21_F_3_N_2_, found 370.1648 ([M]^·+^). qHNMR (400 MHz, DMSO-*d*_6_) purity = 98.4%.

##### 3-(1-Methyl-1H-pyrrolo[2,3-b]pyridin-5-yl)-N-phenethyl-5-(trifluoromethyl)aniline (21)

Preparation according to general procedure A from **64** (100 mg, 0.29 mmol, 1.0 eq.) and 1-methyl-5-(4,4,5,5-tetramethyl-1,3,2-dioxaborolan-2-yl)-1*H*-pyrrolo[2,3-b]pyridine (**SM5**, 96 mg, 0.35 mmol, 1.2 eq.). Further purification by RP column chromatography yielded a colorless oil (55 mg, 48%). ^1^H NMR (400 MHz, DMSO-*d*_6_) δ 8.54 (d, *J* = 2.2 Hz, 1H), 8.21 (d, *J* = 2.2 Hz, 1H), 7.57 (d, *J* = 3.5 Hz, 1H), 7.31 (d, *J* = 4.4 Hz, 4H), 7.22 (h, *J* = 4.0 Hz, 1H), 7.10 (d, *J* = 9.1 Hz, 2H), 6.90 – 6.84 (m, 1H), 6.52 (d, *J* = 3.4 Hz, 1H), 6.31 (t, *J* = 5.5 Hz, 1H), 3.85 (s, 3H), 3.46 – 3.36 (m, 2H), 2.89 (t, *J* = 7.4 Hz, 2H). ^13^C NMR (101 MHz, DMSO-*d*_6_) δ 149.74, 147.21, 141.29, 140.87, 139.65, 130.99, 130.54 (q, *J* = 30.8 Hz), 128.75, 128.31, 127.69, 126.67, 126.09, 124.55 (q, *J* = 272.9 Hz), 119.89, 113.35, 110.10 (q, *J* = 4.2 Hz), 106.76 (q, *J* = 3.9 Hz), 99.28, 44.29, 34.77, 30.94. HRMS (GC/EI+): *m/z* calculated 396.1682 for C_23_H_20_F_3_N_3_, found 396.1641 ([M+H]^+^). qHNMR (400 MHz, DMSO-*d*_6_) purity = 95.6%.

##### 2,6-Dimethyl-4-(3-phenethoxy-5-(trifluoromethyl)phenyl)pyridine (22)

Preparation according to general procedure A from **65** (139 mg, 0.40 mmol, 1.0 eq.) and (2,6-dimethylpyridin-4-yl)boronic acid (**SM4**, 102 mg, 0.65 mmol, 1.6 eq.). Further purification by RP column chromatography yielded a colorless solid (116 mg, 77%). Mp 54 °C. ^1^H NMR (400 MHz, DMSO-*d*_*6*_) δ 7.62 (s, 1H), 7.59 – 7.55 (m, 1H), 7.46 (s, 2H), 7.39 – 7.32 (m, 4H), 7.31 – 7.29 (m, 1H), 7.26 – 7.20 (m, 1H), 4.39 (t, *J* = 6.8 Hz, 2H), 3.09 (t, *J* = 6.8 Hz, 2H), 2.48 (s, 6H). ^13^C NMR (126 MHz, DMSO-*d*_*6*_) δ 159.41, 158.01, 145.89, 140.50, 138.10, 131.10 (q, *J* = 31.9 Hz), 129.02, 128.33, 126.35, 123.85 (q, *J* = 272.7 Hz), 117.88, 116.72, 115.42 (q, *J* = 3.7 Hz), 111.64 (q, *J* = 4.0 Hz), 68.88, 34.85, 23.98. HRMS (DEP/EI+): *m/z* calculated 371.1492 for C_22_H_20_F_3_NO, found 371.1492 ([M]^·+^). qHNMR (400 MHz, DMSO-*d*_6_) purity = 96.1%.

##### Methyl-5-(3-phenethoxy-5-(trifluoromethyl)phenyl)-1H-pyrrolo[2,3-b]pyridine (23)

Preparation according to general procedure A from **65** (143 mg, 0.41 mmol, 1.0 eq.) and 1-methyl-5-(4,4,5,5-tetramethyl-1,3,2-dioxaborolan-2-yl)-1*H*-pyrrolo[2,3-b]pyridine (**SM5**, 137 mg, 0.50 mmol, 1.2 eq.). Further purification by RP column chromatography yielded a colorless oil (44 mg, 27%). ^1^H NMR (400 MHz, DMSO-*d*_6_) δ 8.63 (d, *J* = 2.2 Hz, 1H), 8.33 (d, *J* = 2.2 Hz, 1H), 7.61 – 7.54 (m, 3H), 7.41 – 7.30 (m, 4H), 7.28 – 7.19 (m, 2H), 6.53 (d, *J* = 3.4 Hz, 1H), 4.40 (t, *J* = 6.8 Hz, 2H), 3.85 (s, 3H), 3.09 (t, *J* = 6.8 Hz, 2H). ^13^C NMR (126 MHz, DMSO-*d*_6_) δ 159.36, 147.34, 141.79, 141.51, 138.17, 131.17, 130.99 (q, *J* = 31.6 Hz), 129.03, 128.34, 127.06, 126.54, 126.34, 124.01 (q, *J* = 272.7 Hz), 119.90, 116.80, 115.50 (q, *J* = 4.2 Hz), 109.69 (q, *J* = 4.4 Hz), 99.40, 68.78, 34.88, 30.96. HRMS (GC/EI+): *m/z* calculated 396.1444 for C_23_H_19_F_3_N_2_O, found 396.1448 ([M]^·+^). qHNMR (400 MHz, DMSO-*d*_6_) purity = 96.5%.

##### N-Benzyl-3-(2,6-dimethylpyridin-4-yl)-5-(trifluoromethyl)benzamide (24)

Preparation according to general procedure A from **66** (146 mg, 0.41 mmol, 1.0 eq.) and (2,6-dimethylpyridin-4-yl)boronic acid (**SM4**, 76 mg, 0.49 mmol, 1.2 eq.). Further purification by RP column chromatography yielded a colorless solid (96 mg, 61%). Mp 156 °C. ^1^H NMR (400 MHz, DMSO-*d*_*6*_) δ 9.43 (t, *J* = 5.8 Hz, 1H), 8.57 – 8.52 (m, 1H), 8.31 – 8.28 (m, 1H), 8.28 – 8.25 (m, 1H), 7.54 (s, 2H), 7.39 – 7.31 (m, 4H), 7.29 – 7.22 (m, 1H), 4.56 (d, *J* = 5.6 Hz, 2H), 2.51 (s, 6H). ^13^C NMR (101 MHz, DMSO-*d*_*6*_) δ 164.37, 158.17, 145.48, 139.30, 139.16, 135.98, 130.21 (q, *J* = 32.1 Hz), 129.40, 128.36, 127.40, 126.92, 126.04 (q, *J* = 3.7 Hz), 124.35 (q, *J* = 4.0 Hz), 123.82 (q, *J* = 273.2 Hz), 117.94, 42.90, 24.04. HRMS (GC/EI+): *m/z* calculated 384.1444 for C_22_H_19_F_3_N_2_O, found 384.1443 ([M]^·+^). qHNMR (400 MHz, DMSO-*d*_6_) purity = 97.2%.

##### N-Benzyl-3-(1-methyl-1H-pyrrolo[2,3-b]pyridin-5-yl)-5-(trifluoromethyl)benzamide (25)

Preparation according to general procedure A from **66** (144 mg, 0.40 mmol, 1.0 eq.) and 1-methyl-5-(4,4,5,5-tetramethyl-1,3,2-dioxaborolan-2-yl)-1*H*-pyrrolo[2,3-b]pyridine (**SM5**, 137 mg, 0.50 mmol, 1.3 eq.). Further purification by RP column chromatography yielded a colorless solid (122 mg, 74%). Mp 168 °C. ^1^H NMR (400 MHz, DMSO-*d*_6_) δ 9.42 (t, *J* = 5.8 Hz, 1H), 8.72 (d, *J* = 2.2 Hz, 1H), 8.58 – 8.52 (m, 1H), 8.42 (d, *J* = 2.2 Hz, 1H), 8.25 – 8.21 (m, 1H), 8.21 – 8.17 (m, 1H), 7.61 (d, *J* = 3.4 Hz, 1H), 7.40 – 7.31 (m, 4H), 7.30 – 7.23 (m, 1H), 6.56 (d, *J* = 3.5 Hz, 1H), 4.56 (d, *J* = 5.7 Hz, 2H), 3.87 (s, 3H). ^13^C NMR (101 MHz, DMSO-*d*_6_) δ 164.61, 147.39, 141.57, 140.54, 139.23, 135.85, 131.34, 130.13 (q, *J* = 31.8 Hz), 129.37, 128.37, 127.39, 127.16, 126.90, 126.04, 125.82 (q, *J* = 3.6 Hz), 123.98 (q, *J* = 272.9 Hz), 122.40 (q, *J* = 4.1 Hz), 119.96, 99.44, 42.89, 30.98. HRMS (DEP/EI+): *m/z* calculated 409.1396 for C_23_H_18_F_3_N_3_O, found 409.1409 ([M]^·+^). qHNMR (400 MHz, DMSO-*d*_6_) purity = 95.7%.

##### 1-(3-(2,6-Dimethylpyridin-4-yl)-5-(trifluoromethyl)phenyl)-3-phenylurea (26)

To a stirred solution of phenyl iscocyanate (**SM31**, 54 µL, 0.50 mmol, 1.0 eq.) in dry CH_2_Cl_2_ (3 mL) at 0 °C was added **8** (146 mg, 0.55 mmol, 1.1 eq.) and TEA (156 µL, 1.12 mmol, 2.3 eq.). The mixture was allowed to warm up to rt and stirred overnight, then diluted with water and extracted with EtOAc. The combined organic layers were washed with brine and dried over Na_2_SO_4_. Purification by flash column and RP column chromatography yielded a colorless solid (69 mg, 36%). Mp 113 °C. ^1^H NMR (400 MHz, acetone-*d*_*6*_) δ 8.61 (s, 1H), 8.33 (s, 1H), 8.11 – 8.08 (m, 1H), 8.07 – 8.03 (m, 1H), 7.66 – 7.60 (m, 1H), 7.58 – 7.56 (m, 1H), 7.55 – 7.54 (m, 1H), 7.36 – 7.34 (m, 2H), 7.32 – 7.27 (m, 2H), 7.05 – 7.00 (m, 1H), 2.52 (s, 6H). ^13^C NMR (101 MHz, acetone-*d*_*6*_) δ 159.46, 153.46, 148.07, 142.63, 141.42, 140.51, 132.29 (q, *J* = 32.1 Hz), 129.74, 125.27 (q, *J* = 271.8 Hz), 123.56, 121.20, 119.90, 118.78, 117.81 (q, *J* = 3.8 Hz), 115.95 (q, *J* = 4.0 Hz), 24.64. HRMS (DEP/EI+): *m/z* calculated 385.1396 for C_21_H_18_F_3_N_3_O, found 385.1408 ([M]^·+^). qHNMR (400 MHz, DMSO-*d*_6_) purity = 96.3%.

##### 1-(3-(1-Methyl-1H-pyrrolo[2,3-b]pyridin-5-yl)-5-(trifluoromethyl)phenyl)-3-phenylurea (27)

To a stirred solution of phenyl iscocyanate (**SM31**, 58 µL, 0.53 mmol, 1.0 eq.) in dry CH_2_Cl_2_ (3 mL) at 0 °C was added **6** (171 mg, 0.59 mmol, 1.1 eq.) and TEA (168 µL, 1.20 mmol, 2.3 eq.). The mixture was allowed to warm up to rt and stirred overnight, then diluted with water and extracted with EtOAc. The combined organic layers were washed with brine and dried over Na_2_SO_4_. Purification by flash column and RP column chromatography yielded a colorless solid (19 mg, 9%). Mp 196 °C. ^1^H NMR (400 MHz, acetone-*d*_6_) δ 8.60 (d, *J* = 2.2 Hz, 1H), 8.56 (s, 1H), 8.31 (s, 1H), 8.24 (d, *J* = 2.2 Hz, 1H), 8.09 – 8.04 (m, 1H), 8.04 – 8.01 (m, 1H), 7.64 – 7.61 (m, 1H), 7.59 – 7.55 (m, 2H), 7.49 (d, *J* = 3.5 Hz, 1H), 7.34 – 7.26 (m, 2H), 7.06 – 6.98 (m, 1H), 6.56 (d, *J* = 3.5 Hz, 1H), 3.92 (s, 3H). ^13^C NMR (101 MHz, acetone-*d*_6_) δ 152.59, 147.32, 141.28, 140.79, 139.70, 139.34, 131.24, 130.33 (q, *J* = 31.4 Hz), 128.79 (d, *J* = 2.3 Hz), 128.61, 126.84, 124.20 (q, *J* = 272.6 Hz), 119.99, 118.60, 118.17, 116.85 – 116.25 (m), 113.84, 112.91 – 112.54 (m), 99.42, 31.00. HRMS (DEP/EI+): *m/z* calculated 410.1349 for C_22_H_17_F_3_N_4_O_2_, found 410.1353 ([M]^·+^). qHNMR (400 MHz, DMSO-*d*_6_) purity = 95.5%.

##### Phenyl N-(3-(2,6-dimethylpyridin-4-yl)-5- (trifluoromethyl)phenyl)carbamate (28)

To a stirred solution of **8** (215 mg, 0.81 mmol, 1.5 eq.) in dry CH_2_Cl_2_at 0 °C was added TEA (150 µL, 1.08 mmol, 2.0 eq.) and phenyl chloroformate (**SM32**, 68 µL, 0.54 mmol, 1.0 eq.). After 30 min, the mixture was allowed to warm up to rt and stirred overnight. The reaction was stopped by adding water and the mixture was extracted with EtOAc (3x). The combined organic layers were washed with brine, dried over Na_2_SO_4_, concentrated and purified by flash column and RP column chromatography to yield a colorless solid (7 mg, 3%). Mp 150 °C. ^1^H NMR (400 MHz, acetone-*d*_*6*_) δ 9.64 (s, 1H), 8.25 – 8.20 (m, 1H), 8.10 – 8.05 (m, 1H), 7.77 –7.72 (m, 1H), 7.48 – 7.41 (m, 2H), 7.38 (s, 2H), 7.33 – 7.23 (m,3H), 2.53 (s, 6H). ^13^C NMR (101 MHz, acetone-*d*_*6*_) δ 159.53, 152.87, 151.85, 147.79, 141.68, 141.52, 132.50 (q, *J* = 31.9 Hz), 130.32, 126.59, 125.12 (q, *J* = 271.9 Hz), 122.77, 121.30, 119.11 – 118.86 (m), 118.76, 116.20 – 115.71 (m), 24.60. HRMS (DEP/EI+): *m/z* calculated 386.1237 for C_21_H_17_F_3_N_2_O_2_, found 386.1241 ([M]^·+^). qHNMR (400 MHz, DMSO-*d*_6_) purity = 96.3%.

##### Phenyl N-(3-(1-methyl-1H-pyrrolo[2,3-b]pyridin-5-yl)-5- (trifluoromethyl)phenyl)carbamate (29)

To a stirred solution of **6** (91 mg, 0.31 mmol, 1.0 eq.) in dry CH_2_Cl_2_ at 0 °C was added TEA (88 µL, 0.63 mmol, 2.0 eq.) and phenyl chloroformate (**SM32**, 95 µL, 0.75 mmol, 2.4 eq.). After 30 min, the mixture was allowed to warm up to rt and stirred overnight. The reaction was stopped by adding water and the mixture was extracted with EtOAc (3x). The combined organic layers were washed with brine, dried over Na_2_SO_4_, concentrated and purified by flash column and RP column chromatography to yield a colorless solid (58 mg, 45%). Mp 79 °C. ^1^H NMR (400 MHz, acetone-*d*_6_) δ 9.61 (s, 1H), 8.62 (d, *J* = 2.2 Hz, 1H), 8.25 (d, *J* = 2.2 Hz, 1H), 8.19 – 8.17 (m, 1H), 8.07 – 8.03 (m, 1H), 7.75 – 7.70 (m, 1H), 7.49 (d, *J* = 3.5 Hz, 1H), 7.47 – 7.41 (m, 2H), 7.31 – 7.24 (m, 3H), 6.56 (d, *J* = 3.5 Hz, 1H), 2.87 (s, 3H). ^13^C NMR (101 MHz, acetone-*d*_6_) δ 151.61, 150.65, 147.70, 141.56, 141.32, 140.09, 131.14 (q, *J* = 31.9 Hz), 130.47, 129.05, 126.97, 126.52, 125.28, 124.06 (q, *J* = 271.8 Hz), 121.53, 120.18, 120.15, 117.85 (q, *J* = 3.9 Hz), 113.09 – 112.78 (m), 99.27, 30.15. HRMS (DEP/EI+): *m/z* calculated 411.1189 for C_22_H_16_F_3_N_3_O_2_, found 411.1182 ([M]^·+^). qHNMR (400 MHz, DMSO-*d*_6_) purity = 95.1%.

##### N-(3-(2,6-Dimethylpyridin-4-yl)-5-(trifluoromethyl)phenyl)-1-phenylcyclopropane-1-carboxamide (30)

Preparation according to general procedure B from 1-phenylcyclopropanecarboxylic acid (**SM15**, 157 mg, 0.97 mmol, 2.4 eq.) and **8** (112 mg, 0.42 mmol, 1.0 eq.). Further purification by RP column chromatography yielded a colorless solid (97 mg, 59%). Mp 155 °C. ^1^H NMR (400 MHz, DMSO-*d*_*6*_) δ 9.52 (s, 1H), 8.24 – 8.19 (m, 1H), 8.17 – 8.12 (m, 1H), 7.75 – 7.70 (m, 1H), 7.46 – 7.40 (m, 2H), 7.40 – 7.34 (m, 4H), 7.33 – 7.27 (m, 1H), 2.49 (s, 6H), 1.55 – 1.47 (m, 2H), 1.20 – 1.13 (m, 2H).. ^13^C NMR (126 MHz, DMSO-*d*_*6*_) δ 171.87, 158.10, 146.10, 140.50, 139.63, 139.24, 130.07 (q, *J* = 31.8 Hz), 129.18, 128.59, 127.16, 123.97 (q, *J* = 272.7 Hz), 121.95, 117.98 (q, *J* = 3.9 Hz), 117.68, 116.67 (q, *J* = 4.2 Hz), 31.73, 24.00, 15.01. HRMS (GC/EI+): *m/z* calculated 410.1600 for C_24_H_21_F_3_N_2_O, found 410.1600 ([M]^·+^). qHNMR (400 MHz, DMSO-*d*_6_) purity = 98.8%.

##### N-(3-(1-Methyl-1H-pyrrolo[2,3-b]pyridin-5-yl)-5-(trifluoromethyl)phenyl)-1-phenylcyclopropane-1-carboxamide (31)

Preparation according to general procedure B from 1-phenylcyclopropanecarboxylic acid (**SM15**, 79 mg, 0.48 mmol, 1.2 eq.) and **6** (119 mg, 0.41 mmol, 1.0 eq.). Further purification by RP column chromatography yielded a colorless solid (118 mg, 67%). Mp 161 °C. ^1^H NMR (400 MHz, DMSO-*d*_6_) δ 9.48 (s, 1H), 8.55 (d, *J* = 2.2 Hz, 1H), 8.24 (d, *J* = 2.2 Hz, 1H), 8.20 – 8.15 (m, 1H), 8.10 – 8.04 (m, 1H), 7.72 – 7.66 (m, 1H), 7.58 (d, *J* = 3.4 Hz, 1H), 7.46 – 7.41 (m, 2H), 7.41 – 7.34 (m, 2H), 7.33 – 7.27 (m, 1H), 6.54 (d, *J* = 3.4 Hz, 1H), 3.85 (s, 3H), 1.55 – 1.48 (m, 2H), 1.20 – 1.13 (m, 2H).^13^C NMR (126 MHz, DMSO-*d*_6_) δ 171.79, 147.31, 141.24, 140.47, 140.36, 139.72, 131.25, 129.97 (q, *J* = 31.4 Hz), 129.14, 128.59, 127.13, 126.79, 126.61, 124.13 (q, *J* = 272.6 Hz), 122.05, 119.95, 117.99 (q, *J* = 4.2 Hz), 114.78 (q, *J* = 4.3 Hz), 99.38, 31.76, 30.99, 15.02. HRMS (GC/EI+): *m/z* calculated 435.1553 for C_25_H_20_F_3_N_3_O, found 435.1560 ([M]^·+^). qHNMR (400 MHz, DMSO-*d*_6_) purity = 98.8%.

##### N-(3-(2,6-Dimethylpyridin-4-yl)-5-(trifluoromethyl)phenyl)-N-methyl-2-phenylacetamide (32)

Preparation according to general procedure A from **62** (53 mg, 0.14 mmol, 1.0 eq.) and (2,6-dimethylpyridin-4-yl)boronic acid (**SM4**, 30 mg, 0.19 mmol, 1.3 eq.). Further purification by RP column chromatography yielded a colorless solid (26 mg, 46%). Mp 41 °C. ^1^H NMR (400 MHz, DMSO-*d*_*6*_) δ 8.06 (s, 1H), 8.03 – 7.97 (m, 1H), 7.77 – 7.71 (m, 1H), 7.45 (s, 2H), 7.29 – 7.15 (m, 3H), 7.12 – 6.95 (m, 2H), 3.53 (s, 2H), 3.29 (s, 3H), 2.49 (s, 6H). ^13^C NMR (126 MHz, DMSO-*d*_*6*_) δ 169.77, 158.09, 145.20, 140.20, 135.46, 130.23, 129.00, 128.15, 128.10 – 127.65 (m), 126.85, 126.36, 123.60 (q, *J* = 273.2 Hz), 120.34, 118.14 – 117.49 (m), 117.83, 40.70, 37.08, 24.00. HRMS (GC/EI+): *m/z* calculated 398.1600 for C_23_H_21_F_3_N_2_O, found 398.1598 ([M]^·+^). qHNMR (400 MHz, DMSO-*d*_6_) purity = 95.3%.

##### N-Methyl-N-(3-(1-methyl-1H-pyrrolo[2,3-b]pyridin-5-yl)-5-(trifluoromethyl)phenyl)-2-phenylacetamide (33)

Preparation according to general procedure A from **62** (53 mg, 0.14 mmol, 1.0 eq.) and 1-methyl-5-(4,4,5,5-tetramethyl-1,3,2-dioxaborolan-2-yl)-1*H*-pyrrolo[2,3-*b*]pyridine (**SM5**, 53 mg, 0.20 mmol, 1.4 eq.). Further purification by RP column chromatography yielded a colorless oil (43 mg, 73%). ^1^H NMR (400 MHz, DMSO-*d*_6_) δ 8.63 (d, *J* = 2.2 Hz, 1H), 8.32 (d, *J* = 2.2 Hz, 1H), 8.01 (s, 1H), 7.98 – 7.96 (m, 1H), 7.66 – 7.63 (m, 1H), 7.60 (d, *J* = 3.4 Hz, 1H), 7.28 – 7.15 (m, 3H), 7.06 (s, 2H), 6.55 (d, *J* = 3.4 Hz, 1H), 3.87 (s, 3H), 3.57 (s, 2H), 3.30 (s, 3H). ^13^C NMR (126 MHz, DMSO-*d*_6_) δ 169.81, 147.37, 145.12, 141.50, 135.56, 131.31, 130.54 – 129.55 (m), 129.22, 129.05, 128.35, 128.13, 127.13, 127.03 – 126.54 (m), 126.33, 125.77, 123.75 (q, *J* = 273.2 Hz), 122.34 – 122.05 (m), 119.92, 99.43, 40.65, 37.10, 30.99. HRMS (GC/EI+): *m/z* calculated 423.1553 for C_24_H_20_F_3_N_3_O, found 423.1562 ([M]^·+^). qHNMR (400 MHz, DMSO-*d*_6_) purity = 96.9%.

##### N-(3-(2,6-Dimethylpyridin-4-yl)-5-(trifluoromethyl)phenyl)-1-phenylmethanesulfonamide (34)

Preparation according to general procedure D from benzylsulfonyl chloride (**SM36**, 161 mg, 0.85 mmol, 2.1 eq.) and **8** (108 mg, 0.41 mmol, 1.0 eq.). The crude product was purified by flash column and RP column chromatography to yield a colorless solid (25 mg, 14%). Mp 112 °C. ^1^H NMR (400 MHz, methanol-*d*_*4*_) δ 7.48 (d, *J* = 1.9 Hz, 1H), 7.47 – 7.43 (m, 1H), 7.38 – 7.34 (m, 1H), 7.34 – 7.29 (m, 2H), 7.28 – 7.21 (m, 5H), 4.43 (s, 2H), 2.57 (s, 6H). ^13^C NMR (101 MHz, methanol-*d*_*4*_) δ 159.46, 149.99, 145.52, 141.23, 133.02 (q, *J* = 32.0 Hz), 132.19, 131.48, 129.46, 129.38, 125.40 (q, *J* = 271.7 Hz), 122.43, 119.89, 117.83 – 117.42 (m), 59.54, 23.82, 23.80. HRMS (DEP/EI+): *m/z* calculated 420.1113 for C_21_H_19_F_3_N_2_O_2_S, found 420.1110 ([M]^·+^). qHNMR (400 MHz, DMSO-*d*_6_) purity = 95.3%.

##### N-(3-(1-Methyl-1H-pyrrolo[2,3-b]pyridin-5-yl)-5-(trifluoromethyl)phenyl)-1-phenylmethanesulfonamide (35)

Preparation according to general procedure D from benzylsulfonyl chloride (**SM36**, 161 mg, 0.85 mmol, 2.0 eq.) and **8** (122 mg, 0.42 mmol, 1.0 eq.). Further purification by RP column chromatography yielded a colorless solid (34 mg, 19%). Mp 102 °C. ^1^H NMR (400 MHz, methanol-*d*_*4*_) δ 8.42 (d, *J* = 2.1 Hz, 1H), 8.15 (d, *J* = 2.1 Hz, 1H), 7.53 – 7.49 (m, 1H), 7.50 – 7.47 (m, 1H), 7.42 (d, *J* = 3.5 Hz, 1H), 7.37 – 7.30 (m, 3H), 7.29 – 7.22 (m, 3H), 6.58 (d, *J* = 3.5 Hz, 1H), 4.47 (s, 2H), 3.90 (s, 3H). ^13^C NMR (101 MHz, methanol-*d*_*4*_) δ 148.61, 143.73, 142.68, 142.17, 133.01 (q, *J* = 32.0 Hz), 132.23, 132.20, 131.09, 129.52, 128.96, 128.84, 125.48 (q, *J* = 272.0 Hz), 122.64, 122.41, 118.54 (q, *J* = 3.5 Hz), 115.46 (q, *J* = 3.8 Hz), 101.03, 59.42, 31.65. HRMS (GC/EI+): *m/z* calculated 445.1066 for C_22_H_18_F_3_N_3_O_2_S, found 445.1071 ([M]^·+^). qHNMR (400 MHz, DMSO-*d*_6_) purity = 96.0%.

##### N-Benzyl-3-(2,6-dimethylpyridin-4-yl)-5-(trifluoromethyl)benzenesulfonamide (36)

Preparation according to general procedure A from **67** (120 mg, 0.30 mmol, 1.0 eq.) and (2,6-dimethylpyridin-4-yl)boronic acid (**SM4**, 62 mg, 0.40 mmol, 1.3 eq.). Further purification by RP column chromatography yielded a colorless solid (58 mg, 45%). Mp 143 °C. ^1^H NMR (400 MHz, DMSO-*d*_*6*_) δ 8.49 (s, 1H), 8.29 – 8.27 (m, 1H), 8.27 – 8.25 (m, 1H), 8.03 – 7.97 (m, 1H), 7.46 (s, 2H), 7.21 – 7.09 (m, 5H), 4.13 (s, 2H), 2.53 (s, 6H). ^13^C NMR (101 MHz, DMSO-*d*_*6*_) δ 158.30, 144.63, 143.27, 140.19, 136.80, 130.68 (q, *J* = 32.7 Hz), 128.43, 128.08, 127.71, 127.17, 127.12 – 126.95 (m), 123.25 (q, *J* = 273.3 Hz), 123.21 – 122.89 (m), 117.96, 46.25, 24.00. HRMS (DEP/EI+): *m/z* calculated 420.1113 for C_21_H_19_F_3_N_2_O_2_S, found 420.1132 ([M]^·+^). qHNMR (400 MHz, DMSO-*d*_6_) purity = 98.1%.

##### N-Benzyl-3-(1-methyl-1H-pyrrolo[2,3-b]pyridin-5-yl)-5-(trifluoromethyl)benzenesulfonamide (37)

Preparation according to general procedure A from **67** (117 mg, 0.30 mmol, 1.0 eq.) and 1-methyl-5-(4,4,5,5-tetramethyl-1,3,2-dioxaborolan-2-yl)-1*H*-pyrrolo[2,3-b]pyridine (**SM5**, 99 mg, 0.36 mmol, 1.2 eq.). Further purification by RP column chromatography yielded a colorless solid (98 mg, 74%). Mp 128 °C. ^1^H NMR (400 MHz, DMSO-*d*_6_) δ 8.63 (d, *J* = 2.2 Hz, 1H), 8.47 (s, 1H), 8.35 (d, *J* = 2.3 Hz, 1H), 8.29 – 8.22 (m, 2H), 7.93 (s, 1H), 7.63 (d, *J* = 3.5 Hz, 1H), 7.25 – 7.08 (m, 5H), 6.59 (d, *J* = 3.5 Hz, 1H), 4.14 (s, 2H), 3.88 (s, 3H). ^13^C NMR (101 MHz, DMSO-*d*_6_) δ 147.49, 143.05, 141.48, 136.92, 131.52, 130.56 (q, *J* = 32.6 Hz), 128.33, 128.09, 127.72, 127.32, 127.16, 126.87 (q, *J* = 3.4 Hz), 125.25, 123.41 (q, *J* = 273.1 Hz), 121.07 (q, *J* = 3.8 Hz), 119.98, 99.58, 46.28, 31.03. HRMS (FIA/ESI+): *m/z* calculated 445.1066 for C_22_H_18_F_3_N_3_O_2_S, found 446.1141 ([M+H]^+^). qHNMR (400 MHz, DMSO-*d*_6_) purity = 95.3%.

##### N-(3-(2,6-Dimethylpyridin-4-yl)-5- (trifluoromethyl)phenyl)benzenesulfonamide (38)

Preparation according to general procedure A from **68** (122 mg, 0.32 mmol, 1.0 eq.) and (2,6-dimethylpyridin-4-yl)boronic acid (**SM4**, 60 mg, 0.38 mmol, 1.2 eq.). Further purification by RP column chromatography yielded a colorless solid (32 mg, 25%). Mp 139 °C. ^1^H NMR (400 MHz, acetone-*d*_*6*_) δ 7.94 – 7.86 (m, 2H), 7.81 – 7.76 (m, 1H), 7.75 – 7.70 (m, 1H), 7.69 – 7.62 (m, 1H), 7.62 – 7.53 (m, 3H), 7.26 (s, 2H), 2.51 (s, 6H). ^13^C NMR (101 MHz, acetone-*d*_*6*_) δ 158.38, 146.13, 140.87, 139.48, 139.32, 133.08, 131.51 (q, *J* = 32.2 Hz), 129.12, 126.93, 123.62 (q, *J* = 272.0 Hz), 121.86, 119.07 (q, *J* = 3.7 Hz), 117.51, 116.39 (q, *J* = 3.9 Hz), 23.42. HRMS (DEP/EI+): *m/z* calculated 406.0957 for C_20_H_17_F_3_N_2_O_2_S, found 406.0953 ([M]^·+^). qHNMR (400 MHz, DMSO-*d*_6_) purity = 95.8%.

##### N-(3-(1-Methyl-1H-pyrrolo[2,3-b]pyridin-5-yl)-5-(trifluoromethyl)phenyl)benzenesulfonamide (39)

Preparation according to general procedure A from **68** (119 mg, 0.31 mmol, 1.0 eq.) and 1-methyl-5-(4,4,5,5-tetramethyl-1,3,2-dioxaborolan-2-yl)-1*H*-pyrrolo[2,3-*b*]pyridine (**SM5**, 102 mg, 0.38 mmol, 1.2 eq.). Further purification by RP column chromatography yielded a colorless solid (120 mg, 89%). Mp 130 °C. ^1^H NMR (400 MHz, DMSO-*d*_6_) δ 10.90 (s, 1H), 8.43 (d, *J* = 2.2 Hz, 1H), 8.17 (d, *J* = 2.2 Hz, 1H), 7.89 – 7.81 (m, 2H), 7.69 – 7.66 (m, 1H), 7.65 – 7.56 (m, 5H), 7.38 – 7.32 (m, 1H), 6.55 (d, *J* = 3.5 Hz, 1H), 3.85 (s, 3H). ^13^C NMR (101 MHz, DMSO-*d*_6_) δ 147.35, 141.37, 141.10, 139.56, 139.12, 133.29, 131.39, 130.66 (q, *J* = 31.9 Hz), 129.51, 126.84, 126.73, 126.19, 123.73 (q, *J* = 272.6 Hz), 121.29, 119.96, 118.80 – 118.58 (m), 114.26 – 113.57 (m), 99.48, 31.00. HRMS (GC/EI+): *m/z* calculated 431.0910 for C_21_H_16_F_3_N_3_O_2_S, found 431.0913 ([M]^·+^). qHNMR (400 MHz, DMSO-*d*_6_) purity = 95.6%.

##### N-(3-(2,6-Dimethylpyridin-4-yl)-5-(trifluoromethyl)phenyl)-2-(o-tolyl)acetamide (40)

Preparation according to general procedure C from **8** (78 mg, 0.29 mmol, 1.0 eq.) and *o*-tolylacetic acid (**SM16**, 59 mg, 0.39 mmol, 1.3 eq.). Further purification by RP column chromatography yielded a colorless solid (53 mg, 45%). Mp 76 °C. ^1^H NMR (400 MHz, DMSO-*d*_*6*_) δ 10.62 (s, 1H), 8.23 – 8.17 (m, 1H), 8.14 – 8.08 (m, 1H), 7.77 – 7.71 (m, 1H), 7.37 (s, 2H), 7.30 – 7.25 (m, 1H), 7.21 – 7.13 (m, 3H), 3.74 (s, 2H), 2.50 (s, 6H), 2.31 (s, 3H). ^13^C NMR (101 MHz, DMSO-*d*_*6*_) δ 169.86, 158.16, 146.12, 140.69, 139.66, 136.69, 134.10, 130.40 (q, *J* = 31.8 Hz), 130.13, 129.92, 126.85, 125.80, 123.91 (q, *J* = 272.7 Hz), 120.68, 117.97 – 117.76 (m), 117.72, 115.48 – 115.26 (m), 40.96, 23.99, 19.38. HRMS (DEP/EI+): *m/z* calculated 398.1600 for C_23_H_21_F_3_N_2_O, found 398.1615 ([M]^·+^). qHNMR (400 MHz, DMSO-*d*_6_) purity = 99.3%.

##### N-(3-(2,6-Dimethylpyridin-4-yl)-5-(trifluoromethyl)phenyl)-2- (m-tolyl)acetamide (41)

Preparation according to general procedure C from **8** (64 mg, 0.24 mmol, 1.0 eq.) and *m*-tolylacetic acid (**SM17**, 47 mg, 0.31 mmol, 1.3 eq.). Further purification by RP column chromatography yielded a colorless solid (52 mg, 54%). Mp 118 °C. ^1^H NMR (400 MHz, DMSO-*d*_*6*_) δ 10.69 (s, 1H), 8.25 – 8.18 (m, 1H), 8.16 – 8.11 (m, 1H), 7.79 – 7.74 (m, 1H), 7.45 (s, 2H), 7.23 (t, *J* = 7.5 Hz, 1H), 7.19 – 7.12 (m, 2H), 7.08 (d, *J* = 7.5 Hz, 1H), 3.67 (s, 2H), 2.53 (s, 6H), 2.30 (s, 3H). ^13^C NMR (126 MHz, DMSO-*d*_*6*_) δ 170.02, 157.71, 146.84, 140.76, 139.36, 137.42, 135.30, 130.43 (q, *J* = 31.7 Hz), 129.85, 128.27, 127.33, 126.28, 123.90 (q, *J* = 272.7 Hz), 120.77, 118.18, 117.96 (q, *J* = 3.9 Hz), 115.56 (q, *J* = 4.1n Hz), 43.33, 23.55, 20.98. HRMS (DEP/EI+): *m/z* calculated 398.1600 for C_23_H_21_F_3_N_2_O, found 398.1607 ([M]^·+^). qHNMR (400 MHz, DMSO-*d*_6_) purity = 96.2%.

##### N-(3-(2,6-Dimethylpyridin-4-yl)-5-(trifluoromethyl)phenyl)-2- (p-tolyl)acetamide (42)

Preparation according to general procedure C from **8** (65 mg, 0.24 mmol, 1.0 eq.) and *p*-tolylacetic acid (**SM18**, 53 mg, 0.35 mmol, 1.5 eq.). Further purification by RP column chromatography yielded a colorless solid (53 mg, 54%). Mp 160 °C. ^1^H NMR (400 MHz, DMSO-*d*_*6*_) δ 10.60 (s, 1H), 8.20 – 8.15 (m, 1H), 8.13 – 8.09 (m, 1H), 7.75 – 7.71 (m, 1H), 7.36 (s, 2H), 7.23 (d, *J* = 7.8 Hz, 2H), 7.14 (d, *J* = 7.8 Hz, 2H), 3.64 (s, 2H), 2.49 (s, 6H), 2.28 (s, 3H). ^13^C NMR (101 MHz, DMSO-*d*_*6*_) δ 170.10, 158.16, 146.12, 140.71, 139.64, 135.74, 132.34, 130.38 (q, *J* = 31.7 Hz), 129.05, 128.91, 123.91 (q, *J* = 272.6 Hz), 120.69, 117.83 (q, *J* = 3.9 Hz), 117.72, 115.34 (q, *J* = 3.9 Hz), 42.99, 24.00, 20.64. HRMS (DEP/EI+): *m/z* calculated 398.1600 for C_23_H_21_F_3_N_2_O, found 398.1608 ([M]^·+^). qHNMR (400 MHz, DMSO-*d*_6_) purity = 95.9%.

##### N-(3-(2,6-Dimethylpyridin-4-yl)-5-(trifluoromethyl)phenyl)-2- (2-fluorophenyl)acetamide (43)

Preparation according to general procedure C from **8** (81 mg, 0.30 mmol, 1.0 eq.) and 2-fluorophenylacetic acid (**SM19**, 69 mg, 0.45 mmol, 1.5 eq.). Further purification by RP column chromatography yielded a colorless solid (68 mg, 56%). Mp 161 °C. ^1^H NMR (400 MHz, DMSO-*d*_*6*_) δ 10.70 (s, 1H), 8.23 – 8.17 (m, 1H), 8.12 – 8.07 (m, 1H), 7.77 – 7.73 (m, 1H), 7.45 – 7.39 (m, 1H), 7.37 (s, 2H), 7.36 – 7.30 (m, 1H), 7.24 – 7.15 (m, 2H), 3.80 (s, 2H), 2.50 (s, 6H). ^13^C NMR (101 MHz, DMSO-*d*_*6*_) δ 168.90, 160.69 (d, *J* = 244.3 Hz), 158.17, 146.10, 140.60, 139.69, 132.10 (d, *J* = 4.4 Hz), 130.42 (q, *J* = 31.8 Hz), 128.98 (d, *J* = 8.2 Hz), 124.27 (d, *J* = 3.3 Hz), 123.90 (q, *J* = 272.9 Hz), 122.42 (d, *J* = 15.8 Hz), 120.69, 118.04 – 117.83 (m), 117.73, 115.52 – 115.28 (m), 115.05 (d, *J* = 21.5 Hz), 36.38, 23.98. HRMS (DEP/EI+): *m/z* calculated 402.1350 for C_22_H_18_F_4_N_2_O, found 402.1338 ([M]^·+^). qHNMR (400 MHz, DMSO-*d*_6_) purity = 95.7%.

##### 2-(2-Chlorophenyl)-N-(3-(2,6-dimethylpyridin-4-yl)-5-(trifluoromethyl)phenyl)acetamide (44)

Preparation according to general procedure B from **8** (115 mg, 0.43 mmol, 1.0 eq.) and chlorophenylacetic acid (**SM20**, 78 mg, 0.46 mmol, 1.1 eq.). Further purification by RP column chromatography yielded a colorless solid (110 mg, 63%). Mp 186 °C. ^1^H NMR (400 MHz, DMSO-*d*_*6*_) δ 10.72 (s, 1H), 8.21 (s, 1H), 8.10 (s, 1H), 7.75 (s, 1H), 7.50 – 7.41 (m, 2H), 7.39 – 7.28 (m, 4H), 3.91 (s, 2H), 2.50 (s, 6H). ^13^C NMR (101 MHz, DMSO-*d*_*6*_) δ 168.80, 158.17, 146.11, 140.66, 139.70, 133.72, 133.44, 132.35, 130.44 (q, *J* = 31.5 Hz), 129.02, 128.78, 127.11, 123.91 (q, *J* = 272.7 Hz), 120.62, 117.86 (q, *J* = 3.8 Hz), 117.72, 115.31 (q, *J* = 4.3 Hz), 40.84, 24.01. HRMS (DEP/EI+): *m/z* calculated 418.1054 for C_22_H_18_ClF_3_N_2_O, found 418.1049 ([M]^·+^). qHNMR (400 MHz, DMSO-*d*_6_) purity = 95.7%.

##### N-(3-(2,6-Dimethylpyridin-4-yl)-5-(trifluoromethyl)phenyl)-2- (2-methoxyphenyl)acetamide (45)

Preparation according to general procedure C from **8** (80 mg, 0.30 mmol, 1.0 eq.) and 2-methoxyphenylacetic acid (**SM21**, 63 mg, 0.37 mmol, 1.2 eq.). Further purification by RP column chromatography yielded a colorless solid (73 mg, 59%). Mp 184 °C. ^1^H NMR (400 MHz, DMSO-*d*_*6*_) δ 10.54 (s, 1H), 8.24 – 8.19 (m, 1H), 8.13 – 8.08 (m, 1H), 7.75 – 7.70 (m, 1H), 7.36 (s, 2H), 7.30 – 7.20 (m, 2H), 6.98 (d, *J* = 8.1 Hz, 1H), 6.91 (t, *J* = 7.4 Hz, 1H), 3.77 (s, 3H), 3.69 (s, 2H), 2.49 (s, 6H). ^13^C NMR (101 MHz, DMSO-*d*_*6*_) δ 169.98, 158.15, 157.26, 146.17, 140.86, 139.62, 131.01, 130.38 (q, *J* = 31.7 Hz), 128.23, 123.94 (q, *J* = 272.6 Hz), 123.62, 120.56, 120.17, 117.72, 117.67 – 117.55 (m), 115.49 – 114.99 (m), 110.69, 55.42, 37.78, 23.99. HRMS (DEP/EI+): *m/z* calculated 414.1550 for C_23_H_21_F_3_N_2_O_2_, found 414.1551 ([M]^·+^). qHNMR (400 MHz, DMSO-*d*_6_) purity = 96.9%.

##### 2-(2,6-Difluorophenyl)-N-(3-(2,6-dimethylpyridin-4-yl)-5-(trifluoromethyl)phenyl)acetamide (46)

Preparation according to general procedure C from **8** (65 mg, 0.24 mmol, 1.0 eq.) and 2,6-difluorophenylacetic acid (**SM22**, 57 mg, 0.32 mmol, 1.3 eq.). Further purification by RP column chromatography yielded a colorless solid (61 mg, 60%). Mp 201 °C. ^1^H NMR (400 MHz, DMSO-*d*_*6*_) δ 10.84 (s, 1H), 8.25 – 8.21 (m, 1H), 8.09 – 8.05 (m, 1H), 7.80 – 7.75 (m, 1H), 7.50 – 7.36 (m, 3H), 7.17 – 7.07 (m, 2H), 3.85 (s, 2H), 2.52 (s, 6H). ^13^C NMR (126 MHz, DMSO-*d*_*6*_) δ 167.84, 162.11 (d, *J* = 8.2 Hz), 160.15 (d, *J* = 8.4 Hz), 157.73, 146.78, 140.51, 139.44, 130.47 (q, *J* = 31.9 Hz), 129.44 (t, *J* = 10.3 Hz), 127.72 – 120.19 (m), 120.76, 118.15, 118.10 (d, *J* = 3.9 Hz), 115.67 – 115.50 (m), 111.42 – 111.35 (m), 111.25 –111.20 (m), 111.35 – 111.01 (m), 29.92 (t, *J* = 2.4 Hz), 23.54. HRMS (GC/EI+): *m/z* calculated 420.1256 for C_22_H_17_F_5_N_2_O, found 420.1252 ([M]^·+^). qHNMR (400 MHz, DMSO-*d*_6_) purity = 96.5%.

##### 2-(2,3-Difluorophenyl)-N-(3-(2,6-dimethylpyridin-4-yl)-5-(trifluoromethyl)phenyl)acetamide (47)

Preparation according to general procedure C from **8** (67 mg, 0.25 mmol, 1.0 eq.) and 2-(2,3-difluorophenyl)acetic acid (**SM23**, 55 mg, 0.31 mmol, 1.3 eq.). Further purification by RP column chromatography yielded a colorless solid (53 mg, 50%). Mp 110 °C. ^1^H NMR (400 MHz, DMSO-*d*_*6*_) δ 10.76 (s, 1H), 8.22 – 8.17 (m, 1H), 8.11 – 8.06 (m, 1H), 7.78 – 7.73 (m, 1H), 7.41 – 7.31 (m, 3H), 7.27 – 7.15 (m, 2H), 3.87 (d, *J* = 1.4 Hz, 2H), 2.49 (s, 6H). ^13^C NMR (101 MHz, DMSO-*d*_*6*_) δ 168.35, 158.17, 149.68 (dd, *J* = 244.3, 12.8 Hz), 148.44 (dd, *J* = 245.3, 12.6 Hz), 146.08, 140.51, 139.71, 130.44 (q, *J* = 32.0 Hz), 127.21 (t, *J* = 3.3 Hz), 125.06 (d, *J* = 12.5 Hz), 124.51 (dd, *J* = 6.6, 4.7 Hz), 123.89 (q, *J* = 272.5 Hz), 120.73, 118.00 (q, *J* = 3.7 Hz), 117.73, 116.08 (d, *J* = 16.8 Hz), 115.41 (q, *J* = 3.6 Hz), 36.16, 24.01. HRMS (DEP/EI+): *m/z* calculated 420.1256 for C_22_H_17_F_5_N_2_O, found 420.1259 ([M]^·+^). qHNMR (400 MHz, DMSO-*d*_6_) purity = 96.7%.

##### 2-(2,4-Difluorophenyl)-N-(3-(2,6-dimethylpyridin-4-yl)-5-(trifluoromethyl)phenyl)acetamide (48)

Preparation according to general procedure C from **8** (64 mg, 0.24 mmol, 1.0 eq.) and (2,4-difluorophenyl)acetic acid (**SM24**, 59 mg, 0.34 mmol, 1.4 eq.). Further purification by RP column chromatography yielded a colorless solid (43 mg, 42%). Mp 198 °C. ^1^H NMR (400 MHz, DMSO-*d*_*6*_) δ 10.73 (s, 1H), 8.22 – 8.17 (m, 1H), 8.11 – 8.06 (m, 1H), 7.77 – 7.73 (m, 1H), 7.52 – 7.41 (m, 1H), 7.37 (s, 2H), 7.28 – 7.18 (m, 1H), 7.11 – 7.03 (m, 1H), 3.79 (s, 2H), 2.49 (s, 6H). ^13^C NMR (101 MHz, DMSO-*d*_*6*_) δ 168.76, 161.44 (dd, *J* = 245.1, 12.0 Hz), 160.66 (dd, *J* = 234.9, 12.2 Hz), 158.18, 146.11, 140.58, 139.70, 133.10 (dd, *J* = 9.7, 6.0 Hz), 130.44 (q, *J* = 31.7 Hz), 123.91 (q, *J* = 272.8 Hz), 120.71, 118.80 (dd, *J* = 16.2, 3.6 Hz), 117.94 (q, *J* = 3.8 Hz), 117.74, 115.40 (q, *J* = 3.3 Hz), 111.23 (dd, *J* = 21.3, 3.4 Hz), 103.60 (t, *J* = 26.0 Hz), 35.76, 24.01 (d, *J* = 2.7 Hz). HRMS (DEP/EI+): *m/z* calculated 420.1256 for C_22_H_17_F_5_N_2_O, found 420.1267 ([M]^·+^). qHNMR (400 MHz, DMSO-*d*_6_) purity = 95.3%.

##### 2-(2,5-Difluorophenyl)-N-(3-(2,6-dimethylpyridin-4-yl)-5-(trifluoromethyl)phenyl)acetamide (49)

Preparation according to general procedure C from **8** (65 mg, 0.24 mmol, 1.0 eq.) and 2,5-difluorophenyl)acetic acid (**SM25**, 56 mg, 0.32 mmol, 1.3 eq.). Further purification by RP column chromatography yielded a colorless solid (31 mg, 31%). Mp 104 °C. ^1^H NMR (400 MHz, DMSO-*d*_*6*_) δ 10.74 (s, 1H), 8.22 – 8.17 (m, 1H), 8.11 – 8.06 (m, 1H), 7.78 – 7.73 (m, 1H), 7.37 (s, 2H), 7.34 – 7.21 (m, 2H), 7.21 – 7.14 (m, 1H), 3.82 (d, *J* = 1.5 Hz, 2H), 2.50 (s, 6H). ^13^C NMR (101 MHz, DMSO-*d*_*6*_) δ 168.36, 158.87, 157.87 (dd, *J* = 239.4, 2.2 Hz), 157.17 (dd, *J* = 240.8, 2.2 Hz), 146.09, 140.51, 139.71, 130.43 (q, *J* = 31.6 Hz), 124.37 (dd, *J* = 19.0, 8.8 Hz), 123.90 (q, *J* = 272.5 Hz), 120.73, 118.47 (dd, *J* = 24.4, 4.7 Hz), 118.05 – 117.91 (m), 117.73, 116.40 (dd, *J* = 24.9, 8.9 Hz), 115.52 – 115.25 (m), 115.17 (dd, *J* = 24.3, 8.8 Hz), 36.19, 24.00 (d, *J* = 2.3 Hz). HRMS (GC/EI+): *m/z* calculated 420.1256 for C_22_H_17_F_5_N_2_O, found 420.1253 ([M]^·+^). qHNMR (400 MHz, DMSO-*d*_6_) purity = 95.8%.

##### 2-(2-Chloro-6-fluorophenyl)-N-(3-(2,6-dimethylpyridin-4-yl)-5-(trifluoromethyl)phenyl)acetamide (50)

Preparation according to general procedure C from **8** (65 mg, 0.25 mmol, 1.0 eq.) and 2-chloro-6-fluorophenylacetic acid (**SM26**, 58 mg, 0.31 mmol, 1.3 eq.). Further purification by RP column chromatography yielded a colorless solid (46 mg, 43%). Mp 211 °C. ^1^H NMR (400 MHz, DMSO-*d*_*6*_) δ 10.81 (s, 1H), 8.23 – 8.17 (m, 1H), 8.09 – 8.03 (m, 1H), 7.78 – 7.73 (m, 1H), 7.43 – 7.34 (m, 4H), 7.29 – 7.23 (m, 1H), 3.95 (s, 2H), 2.49 (s, 6H). ^13^C NMR (101 MHz, DMSO-*d*_*6*_) δ 167.65, 161.30 (d, *J* = 247.0 Hz), 158.17, 146.07, 140.50, 139.73, 134.99 (d, *J* = 5.7 Hz), 130.45 (q, *J* = 31.9 Hz), 129.67 (d, *J* = 9.8 Hz), 125.13 (d, *J* = 3.2 Hz), 123.88 (q, *J* = 272.5 Hz), 121.64 (d, *J* = 18.9 Hz), 120.61, 118.12 – 117.87 (m), 117.72, 115.54 – 115.06 (m), 114.20 (d, *J* = 22.7 Hz), 33.92, 24.00. HRMS (GC/EI+): *m/z* calculated 436.0960 for C_22_H_17_ClF_4_N_2_O, found 436.0957 ([M]^·+^). qHNMR (400 MHz, DMSO-*d*_6_) purity = 95.9%.

##### N-(3-(2,6-Dimethylpyridin-4-yl)-5-(trifluoromethyl)phenyl)-2-(2-fluoro-6-(trifluoromethyl)phenyl)acetamide (51)

Preparation according to general procedure C from **8** (65 mg, 0.25 mmol, 1.0 eq.) and 2-chloro-6-fluorophenylacetic acid (**SM27**, 62 mg, 0.27 mmol, 1.1 eq.). Further purification by RP column chromatography yielded a colorless solid (62 mg, 54%). Mp 216 °C. ^1^H NMR (400 MHz, DMSO-*d*_*6*_) δ 10.80 (s, 1H), 8.20 – 8.14 (m, 1H), 8.07 – 8.01 (m, 1H), 7.78 – 7.73 (m, 1H), 7.65 – 7.55 (m, 3H), 7.37 (s, 2H), 4.00 (s, 2H), 2.49 (s, 6H). ^13^C NMR (101 MHz, DMSO-*d*_*6*_) δ 167.55, 161.60 (d, *J* = 246.1 Hz), 158.17, 146.05, 140.45, 139.78, 130.48 (q, *J* = 31.8 Hz), 130.05 – 129.65 (m), 129.80 – 129.39 (m), 123.87 (d, *J* = 272.5 Hz), 125.15 – 122.20 (m), 121.92 – 121.59 (m), 121.22 (d, *J* = 18.1 Hz), 120.55, 119.65 (d, *J* = 22.9 Hz), 117.97 (q, *J* = 3.8 Hz), 117.72, 115.52 – 115.00 (m), 32.87, 23.98. HRMS (GC/EI+): *m/z* calculated 470.1224 for C_23_H_17_F_7_N_2_O, found 470.1222 ([M]^·+^). qHNMR (400 MHz, DMSO-*d*_6_) purity = 95.9%.

##### 2-(2-Methoxy-6-fluorophenyl)-N-(3-(2,6-dimethylpyridin-4-yl)-5-(trifluoromethyl)phenyl)acetamide (52)

Preparation according to general procedure C from **8** (70 mg, 0.26 mmol, 1.0 eq.) and 2-fluoro-6-methoxyphenylacetic acid (**SM28**, 67 mg, 0.30 mmol, 1.3 eq.). Further purification by RP column chromatography yielded a colorless solid (62 mg, 54%). Mp 196 °C. ^1^H NMR (400 MHz, DMSO-*d*_*6*_) δ 10.64 (s, 1H), 8.24 – 8.18 (m, 1H), 8.10 – 8.04 (m, 1H), 7.76 – 7.71 (m, 1H), 7.37 (s, 2H), 7.35 – 7.26 (m, 1H), 6.87 (d, *J* = 8.4 Hz, 1H), 6.82 (t, *J* = 8.7 Hz, 1H), 3.80 (s, 3H), 3.74 (d, *J* = 1.3 Hz, 2H), 2.50 (s, 6H). ^13^C NMR (101 MHz, DMSO-*d*_*6*_) δ 168.84, 161.33 (d, *J* = 242.4 Hz), 158.63 (d, *J* = 8.4 Hz), 158.16, 146.14, 140.73, 139.65, 130.40 (q, *J* = 31.9 Hz), 128.80 (d, *J* = 10.5 Hz), 123.92 (q, *J* = 272.6 Hz), 120.54, 117.72, 115.39 – 115.05 (m), 110.97 (d, *J* = 18.6 Hz), 107.33 (d, *J* = 22.6 Hz), 106.89 (d, *J* = 2.1 Hz), 56.15, 30.34, 24.01. HRMS (GC/EI+): *m/z* calculated 432.1455 for C_23_H_20_F_4_N_2_O_2_, found 432.1453 ([M]^·+^). qHNMR (400 MHz, DMSO-*d*_6_) purity = 95.3%.

##### 2-(2-(Difluoromethoxy)-6-fluorophenyl)-N-(3-(2,6-dimethylpyridin-4-yl)-5-(trifluoromethyl)phenyl)acetamide (53)

Preparation according to general procedure C from **8** (69 mg, 0.26 mmol, 1.0 eq.) and 2-(2-(difluoromethoxy)-6-fluorophenyl)acetic acid (**SM29**, 70 mg, 0.30 mmol, 1.2 eq.). Further purification by RP column chromatography yielded a colorless solid (56 mg, 47%). Mp 190 °C. ^1^H NMR (400 MHz, DMSO-*d*_*6*_) δ 10.76 (s, 1H), 8.21 – 8.16 (m, 1H), 8.08 – 8.03 (m, 1H), 7.77 – 7.72 (m, 1H), 7.47 – 7.39 (m, 1H), 7.37 (s, 2H), 7.24 (t, *J* = 73.5 Hz, 1H), 7.16 (t, *J* = 8.6 Hz, 1H), 7.09 (d, *J* = 8.3 Hz, 1H), 3.83 (s, 2H), 2.50 (s, 6H). ^13^C NMR (101 MHz, DMSO-*d*_*6*_) δ 168.09, 161.30 (d, *J* = 245.1 Hz), 158.17, 150.62 (d, *J* = 7.8 Hz), 146.10, 140.54, 139.71, 130.44 (q, *J* = 31.7 Hz), 129.32 (d, *J* = 10.2 Hz), 123.90 (q, *J* = 272.5 Hz), 120.66, 118.02 – 117.85 (m), 117.73, 116.56 (t, *J* = 258.2 Hz), 115.49 – 115.23 (m), 114.77 (d, *J* = 19.2 Hz), 113.90 (d, *J* = 3.2 Hz), 112.07 (d, *J* = 22.2 Hz), 30.65, 24.00. HRMS (GC/EI+): *m/z* calculated 468.1267 for C_23_H_18_F_6_N_2_O_2_, found 468.1265 ([M]^·+^). qHNMR (400 MHz, DMSO-*d*_6_) purity = 95.6%.

##### 1-(2-Fluorophenyl)-N-(3-(1-methyl-1H-pyrrolo[2,3-b]pyridin-5-yl)-5-(trifluoromethyl)phenyl)methanesulfonamide (54)

Preparation according to general procedure D from **6** (171 mg, 0.59 mmol, 1.1 eq.) and (2-fluorophenyl)methanesulfonyl chloride (**SM30**, 113 mg, 0.54 mmol, 1.0 eq.). The crude product was purified by flash column and RP column chromatography to yield a colorless solid (136 mg, 55%). Mp 159 °C. ^1^H NMR (400 MHz, DMSO-*d*_6_) δ 10.53 (s, 1H), 8.52 (d, *J* = 2.2 Hz, 1H), 8.22 (d, *J* = 2.2 Hz, 1H), 7.68 – 7.66 (m, 1H), 7.65 – 7.64 (m, 1H), 7.61 (d, *J* = 3.4 Hz, 1H), 7.49 – 7.41 (m, 1H) 7.40 – 7.33 (m, 2H), 7.23 – 7.11 (m, 2H), 6.58 (d, *J* = 3.4 Hz, 1H), 4.66 (s, 2H), 3.87 (s, 3H). ^13^C NMR (101 MHz, DMSO-*d*_6_) δ 160.89 (d, *J* = 248.1 Hz), 147.34, 141.25, 141.18, 140.66, 133.31 (d, *J* = 3.2 Hz), 131.33, 130.85 (d, *J* = 8.3 Hz), 130.54 (q, *J* = 31.7 Hz), 126.87, 126.58, 124.41 (d, *J* = 3.6 Hz), 123.93 (q, *J* = 272.5 Hz), 120.31, 119.95, 117.99 – 117.26 (m), 116.76 (d, *J* = 14.8 Hz), 115.40 (d, *J* = 21.8 Hz), 113.37 – 112.76 (m), 99.45, 51.43, 31.14 – 30.83 (m). HRMS (DEP/EI+): *m/z* calculated 463.0972 for C_22_H_17_F_4_N_3_O_2_S, found 463.0972 ([M]^·+^). qHNMR (400 MHz, DMSO-*d*_6_) purity = 95.1%.

##### 1-(2,6-Difluorophenyl)-N-(3-(1-methyl-1H-pyrrolo[2,3-b]pyridin-5-yl)-5-(trifluoromethyl)phenyl)methanesulfonamide (55)

Preparation according to general procedure D from **6** (232 mg, 0.80 mmol, 1.4 eq.) and (2,6-difluorophenyl)methanesulfonyl chloride (**SM34**, 143 mg, 0.60 mmol, 1.0 eq.). The crude product was purified by flash column and RP column chromatography to yield a colorless solid (99 mg, 36%). Mp 182 °C. ^1^H NMR (400 MHz, DMSO-*d*_6_) δ 10.68 (s, 1H), 8.53 (d, *J* = 2.2 Hz, 1H), 8.23 (d, *J* = 2.2 Hz, 1H), 7.77 – 7.70 (m, 1H), 7.70 – 7.67 (m, 1H), 7.61 (d, *J* = 3.4 Hz, 1H), 7.51 – 7.38 (m, 2H), 7.16 – 7.06 (m, 2H), 6.58 (d, *J* = 3.5 Hz, 1H), 4.65 (s, 2H), 3.87 (s, 3H). ^13^C NMR (101 MHz, DMSO-*d*_6_) δ 162.43 (d, *J* = 7.1 Hz), 159.94 (d, *J* = 7.1 Hz), 147.36, 141.27, 141.22, 140.26, 131.58 (t, *J* = 10.2 Hz), 131.37, 130.60 (q, *J* = 32.0 Hz), 126.87, 126.51, 123.90 (q, *J* = 272.7 Hz), 120.07, 119.97, 118.14 – 117.80 (m), 112.94 – 112.47 (m), 111.94 – 111.65 (m), 111.71 – 111.37 (m), 106.13 (t, *J* = 19.4 Hz), 99.47, 46.27, 31.00. HRMS (DEP/EI+): *m/z* calculated 481.0878 for C_22_H_16_F_5_N_3_O_2_S, found 481.1664 ([M]^·+^). qHNMR (400 MHz, DMSO-*d*_6_) purity = 96.4%.

##### N-(3-(1-Methyl-1H-pyrrolo[2,3-b]pyridin-5-yl)-5- (trifluoromethyl)phenyl)-1-(2-(trifluoromethyl)phenyl)methanesulfonamide (56)

Preparation according to general procedure D from **6** (160 mg, 0.55 mmol, 1.1 eq.) and 2-(trifluoromethyl)benzylsulfonyl chloride (**SM35**, 127 mg, 0.49 mmol, 1.0 eq.). The crude product was purified by flash column and RP column chromatography to yield a colorless solid (154 mg, 60%). Mp 171 °C. ^1^H NMR (400 MHz, DMSO-*d*_6_) δ 10.65 (s, 1H), 8.53 (d, *J* = 2.2 Hz, 1H), 8.23 (d, *J* = 2.2 Hz, 1H), 7.73 (d, *J* = 7.9 Hz, 1H), 7.70 – 7.66 (m, 4H), 7.61 (d, *J* = 3.5 Hz, 1H), 7.59 – 7.51 (m, 1H), 7.42 – 7.38 (m, 1H), 6.57 (d, *J* = 3.4 Hz, 1H), 4.80 (s, 2H), 3.87 (s, 3H). ^13^C NMR (101 MHz, DMSO-*d*_6_) δ 147.36, 141.35, 141.22, 139.87, 133.92, 132.43, 131.37, 130.67 (q, *J* = 31.6 Hz), 129.23, 128.45 (q, *J* = 29.8 Hz), 127.06 – 126.96 (m), 126.87, 126.48 (q, *J* = 5.4 Hz), 126.39, 124.00 (q, *J* = 274.4 Hz), 123.84 (q, *J* = 272.5 Hz), 120.36, 119.95, 118.30 – 117.96 (m), 113.08 – 112.69 (m), 99.45, 54.82, 31.00 (d, *J* = 2.6 Hz).. HRMS (DEP/EI+): *m/z* calculated 513.0940 for C_23_H_17_F_6_N_3_O_2_S, found 513.0941 ([M]^·+^). qHNMR (400 MHz, DMSO-*d*_6_) purity = 96.2%.

##### 2-(2-(Difluoromethoxy)-6-fluorophenyl)-N-(3’-methyl-5-(trifluoromethyl)-[1,1’-biphenyl]-3-yl)acetamide (58)

Preparation according to general procedure C from **57** (78 mg, 0.34 mmol, 1.1 eq.) and 2-(2-(difluoromethoxy)-6-fluorophenyl)acetic acid (**SM29**, 78 mg, 0.31 mmol, 1.0 eq.). Further purification by RP column chromatography yielded a colorless solid (94 mg, 67%). Mp 173 °C. ^1^H NMR (400 MHz, DMSO-*d*_*6*_) δ 10.71 (s, 1H), 8.10 – 8.06 (m, 1H), 8.04 – 7.99 (m, 1H), 7.65 – 7.61 (m, 1H), 7.53 – 7.35 (m, 4H), 7.28 – 7.02 (m, 4H), 3.82 (s, 2H), 2.39 (s, 3H). ^13^C NMR (101 MHz, DMSO-*d*_*6*_) δ 168.00, 161.30 (d, *J* = 245.1 Hz), 151.12 – 150.02 (m), 142.14, 140.38, 138.48, 138.44, 130.21 (q, *J* = 31.6 Hz), 129.29 (d, *J* = 10.2 Hz), 129.05 (d, *J* = 2.1 Hz), 127.44, 124.03 (q, *J* = 272.5 Hz), 123.94, 120.63, 117.72, 116.57 (t, *J* = 258.9 Hz), 114.85 (d, *J* = 19.2 Hz), 114.06 – 113.97 (m), 113.98 – 113.81 (m), 112.07 (d, *J* = 22.1 Hz), 30.66, 20.99. HRMS (GC/EI+): *m/z* calculated 453.1158 for C_23_H_17_F_6_NO_2_, found 453.1156 ([M]^·+^). qHNMR (400 MHz, DMSO-*d*_6_) purity = 96.4%.

##### N-(3’-Methyl-5-(trifluoromethyl)-[1,1’-biphenyl]-3-yl)acetamide (59)

Acetyl chloride (**SM30**, 42.7 µL, 0.60 mmol, 1.5 eq.) was added dropwise to a stirring solution of **57** (101 mg, 0.40 mmol, 1.0 eq.) in CHCl_3_ (dry, 6 mL) at 0°C. The mixture was allowed to warm up to rt and stirred for 2 h. The reaction was quenched with water (20 mL) and extracted with CH_2_Cl_2_ (3 × 10 mL). The combined organic layers were washed with brine, dried over Na_2_SO_4_ and concentrated. Purification by RP column chromatography yielded a colorless solid (60.2 mg, 51%). Mp 139 °C. ^1^H NMR (500 MHz, CD_2_Cl_2_) δ 7.90 – 7.87 (m, 1H), 7.87 – 7.84 (m, 1H), 7.60 – 7.56 (m, 1H), 7.46 (s, 1H), 7.42 (td, *J* = 1.8, 0.9 Hz, 1H), 7.41 – 7.37 (m, 1H), 7.35 (t, *J* = 7.5 Hz, 1H), 7.26 – 7.20 (m, 1H), 2.42 (s, 3H), 2.19 (s, 3H). ^13^C NMR (126 MHz, CD_2_Cl_2_) δ 168.83, 143.45, 139.63, 139.58, 139.24, 131.85 (q, *J* = 32.2 Hz), 129.38, 129.26, 128.22, 124.57, 124.47 (q, *J* = 272.7 Hz), 121.56, 119.65 (q, *J* = 4.0 Hz), 115.27 (q, *J* = 3.9 Hz), 24.83, 21.59. HRMS (GC/EI+): *m/z* calculated 293.1022 for C_16_H_14_F_3_NO, found 293.1023 ([M] ^·+^). qHNMR (400 MHz, DMSO-*d*_6_) purity = 99.3%.

##### N-Benzyl-3-bromo-5-(trifluoromethyl)aniline (60)

3-Bromo-5-(trifluoromethyl)aniline (**SM1**, 866 µL, 6.00 mmol, 1.0 eq.), benzyl bromide (**SM2**, 1.08 mL, 9.00 mmol, 1.5 eq.) and K_2_CO_3_ (1.28 g, 9.25 mmol, 1.5 eq.) were dissolved in dry DMF (8 mL) and the reaction mixture was stirred at 90°C for 20h. After cooling to rt, the mixture was diluted with EtOAc, washed with brine (3x), dried over Na_2_SO_4_ and concentrated. The residue was purified by flash column chromatography and lyophilized to give a pale-yellow oil (1.02 g, 62%). ^1^H NMR (400 MHz, DMSO-*d*_6_) δ 7.38 – 7.33 (m, 4H), 7.29 – 7.23 (m, 1H), 7.05 (t, *J* = 5.9 Hz, 1H), 6.99 – 6.95 (m, 1H), 6.94 – 6.91 (m, 1H), 6.88 – 6.85 (m, 1H), 4.33 (d, *J* = 5.9 Hz, 2H). ^13^C NMR (101 MHz, DMSO-*d*_6_) δ 150.54, 138.81, 131.39 (q, *J* = 31.7 Hz), 128.47, 127.26, 127.00, 123.47 (q, *J* = 273.5 Hz), 122.79, 117.32, 114.11 – 113.34 (m), 107.93 – 107.40 (m), 45.95. MS (APCI+): m/z 330.2 ([M]^·+^).

##### N-(3-Bromo-5-(trifluoromethyl)phenyl)-2-phenylacetamide (61)

Preparation according to general procedure C from phenylacetic acid (**SM3**, 686 mg, 5.04 mmol, 1.0 eq.) and 3-bromo-5-(trifluoromethyl)aniline (**SM1**, 800 µL, 5.55 mmol, 1.1 eq.) to give a pale-yellow solid (1.24 g, 69%). ^1^H NMR (400 MHz, DMSO-*d*_6_) δ 10.65 (s, 1H), 8.15 – 8.09 (m, 1H), 8.01 – 7.95 (m, 1H), 7.64 – 7.59 (m, 1H), 7.33 (d, *J* = 4.4 Hz, 4H), 7.29 – 7.23 (m, 1H), 3.68 (s, 2H). ^13^C NMR (101 MHz, DMSO-*d*_6_) δ 170.06, 141.35, 135.22, 131.24 (q, *J* = 32.3 Hz), 129.20, 128.37, 126.74, 124.87, 123.08 (q, *J* = 272.8 Hz), 122.37, 122.11 (q, *J* = 3.2 Hz), 114.21 (q, *J* = 3.9 Hz), 43.30. MS (APCI+): m/z 358.1 ([M]^·+^).

##### N-(3-Bromo-5-(trifluoromethyl)phenyl)-N-methyl-2-phenylacetamide (62)

To a stirred solution of **61** (378 mg, 1.06 mmol, 1.0 eq.) in dry DMF (2.5 mL) at 0 °C was added NaH (60% in mineral oil, 63 mg, 1.58 mmol, 1.5 eq.) and the mixture was stirred for 10 minutes. MeI (131 µL, 2.11 mmol, 2.0 eq.) was added and the solution was allowed to warm up to rt and stirred for 6 h. After quenching with aq. sat. NH_4_Cl, the mixture was extracted with EtOAc and washed three more times with aq. sat. NH_4_Cl. The organic layer was dried over Na_2_SO_4_ and concentrated. After purification by flash column chromatography and lyophilization, the product was yielded as a yellow oil (106 mg, 27%). ^1^H NMR (400 MHz, acetone-*d*_6_) δ 7.85 (s, 1H), 7.82 – 7.78 (m, 1H), 7.68 – 7.63 (m, 1H), 7.30 – 7.16 (m, 3H), 7.16 – 7.04 (m, 2H), 3.62 (s, 2H), 3.32 (s, 3H). ^13^C NMR (101 MHz, acetone-*d*_6_) δ 147.43, 136.45, 135.88 – 135.28 (m), 130.24, 130.01, 129.40, 129.21, 127.47, 125.38, 123.61, 122.67, 41.97, 37.76. MS (APCI+): m/z 372.4 ([M]^·+^).

##### 1-(4-(4,4,5,5-Tetramethyl-1,3,2-dioxaborolan-2-yl)thiophen-2-yl)ethan-1-one (63)

A 250 mL Schlenk flask was charged with B_2_pin_2_ (1426 mg, 5.62 mmol, 1.1 eq.), Pd(dppf)Cl_2_ (224 mg, 0.27 mmol, 5 mol%), KOAc (1512 mg, 15.4 mmol, 3.0 eq.) and a stir bar. The flask was evacuated and backfilled with N_2_ (3x). 2-Acetyl-4-bromothiophene (**SM7**, 658 µL, 5.10 mmol, 1.0 eq.) and dioxane (dry, 45 mL) were added and the reaction mixture was stirred at 90 °C overnight. After cooling to rt, the mixture was filtered over a pad of celite and concentrated in vacuo. The residue was partitioned between water and EtOAc, and the combined organic layers were washed with brine, dried over Na_2_SO_4_ and concentrated. The crude product was purified by flash column and RP column chromatography to yield a colorless solid (522,41%). ^1^H NMR (500 MHz, DMSO-*d*_6_) δ 8.36 (d, *J* = 1.1 Hz, 1H), 8.01 (d, *J* = 1.2 Hz, 1H), 2.56 (s, 3H), 1.30 (s, 12H). ^13^C NMR (126 MHz, DMSO-*d*_6_) δ 191.21, 145.22, 144.22, 138.19, 83.94, 26.84, 26.78, 24.65. MS (APCI+): m/z 253.1 ([M+H]^+^).

##### 3-Bromo-N-phenethyl-5-(trifluoromethyl)aniline (64)

3-Bromo-5-(trifluoromethyl)aniline (**SM1**, 866 µL, 6.00 mmol, 1.5 eq.), 2-phenylethylbromid (**SM8**, 540 µL, 4.00 mmol, 1.0 eq.) and K_2_CO_3_ (756 mg, 5.47 mmol, 1.4 eq.) were dissolved in dry DMF (4 mL) and the reaction mixture was stirred at 90 °C for 20 h. After cooling to rt, the mixture was diluted with EtOAc, washed with brine (3x), dried over Na_2_SO_4_ and concentrated. The residue was purified by flash column chromatography and lyophilized to give a yellow oil (201 mg, 15%). ^1^H NMR (400 MHz, methanol-*d*_*4*_) δ 7.31 – 7.16 (m, 6H), 6.93 – 6.90 (m, 1H), 6.90 – 6.87 (m, 1H), 6.78 – 6.72 (m, 1H), 3.34 (t, *J* = 7.2 Hz, 2H), 2.88 (t, *J* = 7.2 Hz, 2H). ^13^C NMR (101 MHz, methanol-*d*_*4*_) δ 151.90, 140.74, 133.81 (q, *J* = 32.1 Hz), 129.81, 129.53, 127.36, 125.03 (q, *J* = 271.8 Hz), 124.34, 118.52, 115.59 (q, *J* = 4.0 Hz), 108.47 (q, *J* = 4.1 Hz), 45.75, 36.19. MS (APCI+): m/z 344.5 ([M]^·+^).

##### 1-Bromo-3-phenethoxy-5-(trifluoromethyl)benzene (65)

To a solution of 3-bromo-5-(trifluoromethyl)phenol (**SM9**, 690 µL, 5.00 mmol, 1.0 eq.) in ACN (25 mL) was added 2-phenylethylbromid (**SM8**, 1000 µL, 7.40 mmol, 1.5 eq.) and K_2_CO_3_ (1385 mg, 10.02 mmol, 2 eq.). The reaction mixture was stirred at 80 °C overnight, then concentrated under reduced pressure. The residue was taken up in EtOAc, filtered and washed with 5% HCl solution (2x) and brine. The organic layer was dried over Na_2_SO_4_, concentrated and purified by flash column chromatography to yield a colorless crystalline solid (694 mg, 40%). ^1^H NMR (400 MHz, DMSO-*d*_6_) δ 7.49 (d, *J* = 2.2 Hz, 1H), 7.48 (s, 1H), 7.36 – 7.19 (m, 6H), 4.32 (t, *J* = 6.8 Hz, 2H), 3.04 (t, *J* = 6.8 Hz, 2H). ^13^C NMR (101 MHz, DMSO-*d*_6_) δ 159.68, 137.92, 131.78 (q, *J* = 32.6 Hz), 128.97, 128.31, 126.35, 123.01 (q, *J* = 272.4 Hz), 122.95, 121.48, 119.86 (q, *J* = 4.2 Hz), 111.02 (q, *J* = 3.8 Hz), 69.11, 34.64. MS (GC/EI+): *m/z* 344.0 ([M]^·+^).

##### N-Benzyl-3-bromo-5-(trifluoromethyl)benzamide (66)

Preparation according to general procedure C from 3-bromo-5-(trifluoromethyl)benzoic acid (**SM10**, 1.36 g, 5.04 mmol, 1.0 eq.) and benzylamine (**SM12**, 667 µL, 6.04 mmol, 1.2 eq.) to yield a colorless solid (1.27 g, 70%). ^1^H NMR (400 MHz, DMSO-*d*_6_) δ 9.38 (s, 1H), 8.42 – 8.36 (m, 1H), 8.26 – 8.22 (m, 1H), 8.20 – 8.16 (m, 1H), 7.34 (d, *J* = 4.5 Hz, 4H), 7.29 – 7.23 (m, 1H), 4.50 (s, 2H). ^13^C NMR (101 MHz, DMSO-*d*_6_) δ 163.25, 138.93, 137.05, 134.20, 131.10 (q, *J* = 32.8 Hz), 130.62 (q, *J* = 3.6 Hz), 128.35, 127.43, 126.94, 123.16 (q, *J* = 4.1 Hz), 122.97 (q, *J* = 272.9 Hz), 122.55, 42.96. MS (APCI+): m/z 358.4 ([M]^·+^).

##### N-Benzyl-3-bromo-5-(trifluoromethyl)benzenesulfonamide (67)

Preparation according to general procedure D from 3-bromo-5-(trifluoromethyl)benzenesulfonyl chloride (**SM11**, 545 µL, 3.00 mmol, 1.0 eq.) and benzylamine (**SM12**, 364 µL, 3.30 mmol, 1.1 eq.). Purification by flash column chromatography and lyophilization yielded a colorless solid (681 mg, 58%). ^1^H NMR (400 MHz, DMSO-*d*_6_) δ 8.55 (s, 1H), 8.23 – 8.19 (m, 1H), 8.10 – 8.04 (m, 1H), 7.94 – 7.89 (m, 1H), 7.23 – 7.15 (m, 5H), 4.12 (s, 2H). ^13^C NMR (101 MHz, DMSO-*d*_6_) δ 144.13, 136.59, 133.01, 131.71 (q, *J* = 3.8 Hz), 131.47 (q, *J* = 33.5 Hz), 128.11, 127.76, 127.27, 123.12, 122.42 (q, *J* = 273.7 Hz), 122.05 (q, *J* = 4.1 Hz), 46.25. MS (APCI-): m/z 393.3 ([M-H]^-^).

##### N-(3-Bromo-5-(trifluoromethyl)phenyl)benzenesulfonamide (68)

Preparation according to general procedure E from 3-bromo-5-(trifluoromethyl)aniline (**SM1**, 1.44 mL, 10.00 mmol, 2.0 eq.) and benzenesulfonyl chloride (**SM13**, 645 µL, 5.00 mmol, 1.0 eq.). The crude product was purified by flash column and RP column chromatography to yield a colorless solid (465 mg, 24%). ^1^H NMR (400 MHz, DMSO-*d*_6_) δ 11.02 (s, 1H), 7.83 – 7.81 (m, 1H), 7.81 – 7.78 (m, 1H), 7.70 – 7.64 (m, 1H), 7.63 – 7.57 (m, 3H), 7.54 – 7.48 (m, 1H), 7.40 – 7.35 (m, 1H). ^13^C NMR (101 MHz, DMSO-*d*_6_) δ 140.20, 138.62, 133.60, 131.60 (q, *J* = 32.7 Hz), 129.64, 126.64, 125.15, 122.98 (q, *J* = 3.9 Hz), 122.83, 122.72 (q, *J* = 273.3 Hz), 114.24 (q, *J* = 4.2 Hz). MS (APCI+): m/z 380.4 ([M]^·+^).

### In vitro characterization

#### Hybrid Reporter Gene Assays

TLX modulation was determined in a Gal4 hybrid reporter gene assay in HEK293T cells (German Collection of Microorganisms and Cell Culture GmbH, DSMZ) using pFR-Luc (Stratagene, La Jolla, CA, USA; reporter), pRL-SV40 (Promega, Madison, WI, USA; internal control), pECE-SV40-Gal4-VP16^37^ (#71728, Addgene, Watertown, MA), and pFA-CMV-hTLX-LBD^24^, coding for the hinge region and ligand binding domain of the canonical isoform of human TLX. Cells were cultured in Dulbecco’s modified Eagle’s medium (DMEM), high glucose supplemented with 10% fetal calf serum (FCS), sodium pyruvate (1 mM), penicillin (100 U/mL), and streptomycin (100 μg/mL) at 37 °C and 5% CO2 and seeded in 96-well plates (3×10^4^ cells/well). After 24 h, medium was changed to Opti-MEM without supplements and cells were transiently transfected using Lipofectamine LTX reagent (Invitrogen, Carlsbad, CA, USA) according to the manufacturer’s protocol. Five hours after transfection, cells were incubated with the test compounds in Opti-MEM supplemented with penicillin (100 U/mL), streptomycin (100 μg/mL) and 0.1% DMSO for 16 h before luciferase activity was measured using the Dual-Glo Luciferase Assay System (Promega) according to the manufacturer’s protocol on a Tecan Spark luminometer (Tecan Deutschland GmbH, Crailsheim, Germany). Firefly luminescence was divided by Renilla luminescence and multiplied by 1000 resulting in relative light units (RLU) to normalize for transfection efficiency and cell growth. Fold activation was obtained by dividing the mean RLU of test compound by the mean RLU of the untreated control. All samples were tested in at least three biologically independent experiments in duplicates. For dose-response curve fitting and calculation of EC_50_ values and remaining activity (= “bottom”), the equation “[Agonist] vs. response -- Variable slope (four parameters)” was used in GraphPad Prism (version 7, GraphPad Software, La Jolla, CA, USA). Selectivity profiling was performed with pFA-CMV-THRα-LBD^38^, pFA-CMV-RARα-LBD^39^, pFA-CMV-PPARα-LBD^40^, pFA-CMV-PPARγ-LBD^40^, pFA-CMV-PPARδ-LBD^40^, pFA-CMV-revERBα-LBD^41^, pFA-CMV-RORα-LBD^42^, pFA-CMV-VDR-LBD^43^, pFA-CMV-CAR-LBD^43^, pFA-CMV-PXR-LBD^43^, pFA-CMV-LXRα-LBD^43^, pFA-CMV-FXR-LBD^43^, pFA-CMV-HNF4α-LBD^44^, pFA-CMV-hRXRα-LBD^45^, pFA-CMV-hRXRβ-LBD^45^, pFA-CMV-hRXRγ-LBD^45^, pFA-CMV-TR2-LBD^40^, pFA-CMV-TR4-LBD^40^, pFA-CMV-PNR-LBD^46^, pFA-CMV-ERα-LBD, GAL-AR-1-503^47^ (#89093, Addgene), pFA-CMV-Nur77-LBD^48^, pFA-CMV-hNurr1-LBD^48^, pFA-CMV-hNOR1-LBD^48^, pFA-CMV-SF1-LBD^41^ and pFA-CMV-LRH1-LBD. The following reference ligands were used: T3 (1 µM, THRα), Tretinoin (1 µM, RARα), GW7674 (1 µM, PPARα), Pioglitazone (1 µM, PPARγ), L165,041 (1 µM, PPARδ), SR1001 (1 µM, revERBα and RORα), calcitriol (1 µM, VDR), SR12813 (1 µM, PXR), CITCO (10 µM, CAR), T0901317 (1 µM, LXRα), GW4064 (1µM, FXR), Bexarotene (1 µM, RXRα/β/γ), PR3 (1 µM, PNR), diethylstilbestrol (1 µM, ERα), spironolactone (10 µM, AR), compound 29 from Vietor et al.^49^ (0.3 µM, Nur77 and NOR1) and fluvastatin (10 µM, Nurr1).

#### Affinity Selection Mass Spectrometry (ASMS)

A mixture containing compounds **35** and **53** (1 µM final concentration each) was incubated (at 4 °C for 1h) with TLX-LBD protein (35 µM) in buffer (50 mM Tris, 200 mM NaCl, pH 7.4) containing 1% (v/v) DMSO in a total volume of 55 µL (N=7). In parallel, this procedure was performed for denatured (82 °C, 30 min) TLX-LBD (35 µM) under otherwise identical conditions (N=3). After equilibration of the PD SpinTrap G-25 columns containing Sephadex G-25 medium (Cytiva, Little Chalfont, United Kingdom) according to the manufacturer’s instructions, 50 µL of each binding sample were transferred onto an SEC column followed by centrifugation (800 g, 2 min) to generate the eluates E1. In the same way the pure compound mixture (1 µM in buffer with 1% DMSO) without TLX incubation was subjected to SEC as further negative control (N=3). Next, aliquots of 40 µL of each E1 eluate were diluted to 400 µL with 0.1% HCOOH in H_2_O and 0.1% HCOOH in MeCN (50:50, v/v) containing 100 nM Terfenadine as internal standard for quantification by LC-MS. In the same way samples containing 10 nM test compounds and 100 nM Terfenadine, as well as blanks, were generated by dilution with 40 µL of pure buffer as LC-ESI-MS/MS control. Subsequently, the generated samples were sonicated for 5 min, vortexed and finally centrifuged (15.000 g, 5 min). After transfer of the samples to a 96 deep well plate LC-ESI-MS/MS was performed with an API5500 QTrap mass spectrometer (Sciex, Darmstadt, Germany) coupled to an Agilent 1260 infinity HPLC system (Agilent Technologies, Waldbronn, Germany) equipped with a SIL-20AHT autosampler (Shimadzu, Duisburg, Germany) and a rack changer II (Shimadzu, Duisburg, Germany) controlled by the Analyst software 1.6.3. For chromatography, a Triart C18 column (3 µm, 50 mm x 2 mm, YMC-Europe, protected with a 0.5 µm and 0.2 µm frit) as stationary phase in combination with 0.1 % (v/v) HCOOH in H_2_O (A) and 0.1 % (v/v) HCOOH in MeCN (B) as mobile phase under gradient elution (0-2 min: 90%A/10%B, 2-11 min: 90%A/10%B → 5%A/95%B, 11-12 min: 5%A/95%B, 12-13 min: 5%A/95%B → 90%A/10%B, 13-16 min: 90%A/10%B) was used at a flow rate of 400 µL/min, injecting 50 µL per sample. MS data acquisition was performed under positive ESI conditions in the MRM mode recording **35** at *m/z* 446 → 291, **53** at *m/z* 469 → 293 and Terfenadine at *m/z* 472 → 436 from 4.7 to 7.0 min. Quantification of the test compounds was based on area ratios of analyte vs internal standard.

#### Evaluation of TLX homodimer formation (HTRF)

Modulation of TLX LBD homodimerization was investigated by titration of sGFP-labeled TLX LBD (FRET acceptor) against a fixed concentration of Tb^3+^-cryptate as streptavidin conjugate (Tb-SA, 610SATLF, Revvity; FRET donor) coupled to biotinylated TLX LBD protein^29^, in presence of a fixed concentration of test compounds (10 µM, solubilized with DMSO) or absence of compounds as a negative control (1% DMSO). Assay solutions were prepared in HTRF assay buffer (25 mM HEPES pH 7.5, 150 mM KF, 5% (m/v) glycerol, 5 mM DTT and 0.1% (m/v) CHAPS). The FRET donor complex was formed from biotinylated TLX LBD (0.5 nM) and Tb-SA (1 nM). The FRET acceptor sGFP-TLX was titrated from 1.2 µM, while free sGFP was added to keep a constant concentration of sGFP at 1.2 µM. Samples were equilibrated at room temperature for 2 h and, after excitation at 340 nm, fluorescence intensities (FI) were recorded for the sGFP acceptor at 520 nm and for the Tb-SA donor at 620 nm on a Tecan SPARK plate reader (Tecan). FI250 was divided by FI620 and multiplied by 10,000 to give a dimensionless HTRF signal. The HTRF data were normalized to control samples containing only biotinylated TLX LBD and sGFP (ΔHTRF) and fit with a one-site binding model in GraphPad Prism (version 9.5.1, GraphPad Software, La Jolla, CA).

#### Evaluation of Microsomal Stability

Metabolic stability of **35** and **53** (final concentration 10 µM) was assessed by incubation with pooled human liver microsomes (#M0317, Merck KGaA, Darmstadt; 0.5 mg/mL) in a 100 mM potassium phosphate based buffer at pH 7.4 containing 1 mM NADPH. Each compound was tested in three independent replicates. 100 µL samples were drawn from each replicate at timepoints 0, 30, 60 and 120 minutes after onset of incubation, microsomal activity was terminated by addition of 500 µL MeCN, samples were centrifuged at 10000 g for 5 minutes, and the supernatants were aspirated. Metaboliuc degradation of 7-ethoxycoumarin (7-EC) was evaluated equally for comparison. A reaction mixture containing heat inactivated microsomes (95 °C, 10 min) served as matrix control. Concentrations of remaining compound at each timepoint were determined by LC-MS/MS on an API-3200-QTrap (Sciex) coupled with an Agilent Technologies 1100 series setup including a binary pump (G1311A), a degasser (G1322A), and a Shimadzu SIL 20A HT autosampler under the control of Analyst 1.6 (Sciex). An XBridge C18 (3.5 µm, 150 mm x 3 mm, Waters, protected with a 0.5 µm frit) stationary phase was used under isocratic conditions with 0.1% formic acid in water (A) and MeCN (B) as mobile phase (50%:50% v/v = A:B). 10 µL of aspirated supernatant diluted in mobile phase was loaded onto the column, separated at a flow rate of 400 µL/min, and detected and quantified per area of MRM (multiple reaction monitoring). Compounds were identified and quantified according to the following *m/z* transitions: 7-ethoxycoumarin, *m/z* 190.990 → *m/z* 163.000 (quantifier), *m/z* 190.990 → *m/z* 107.100 (qualifier); **35**, *m/z* 445.752 → *m/z* 291.000 (quantifier), *m/z* 445.752 → *m/z* 264,000 (qualifier); **53**, *m/z* 468.782 → *m/z* 293.000 (quantifier), *m/z* 468.782 → *m/z* 239.100 (qualifier). % remaining compound data were fit to a one-phase decay (constraint: plateau = 0) in GraphPad Prism (version 7, GraphPad software).

#### transcriptomic analysis of retinal pigment epithelial cells (ARPE-19)

ARPE-19 human retinal pigment epithelial cells (American Type Culture Collection, ATCC) were incubated under serum-reduced conditions with vehicle control (DMSO) or **35** (1 µM) for 16 h. Three independent biological replicates were generated per condition. Cells were harvested and processed for RNA isolation immediately after incubation. RNA was isolated from cell lysates using the Maxwell RSC 48 miRNA Tissue Kit (AS1460, Promega) according to the manufacturer’s instructions. The quality of the isolated RNA was assessed using a Nanodrop ND-1000 (Thermo Fisher Scientific) and a 2100 Bioanalyzer (RNA 6000 Nano, Agilent). Libraries were prepared with the CORALL mRNA-Seq V2 Kit (Lexogen) following the manufacturer’s guidelines and paired-end sequencing was carried out on Illumina NextSeq systems. Samples were sequenced across two technical runs; corresponding FASTQ files were concatenated per sample prior to downstream processing. Adapter and quality trimming was performed using fastp (20 threads). Trimmed reads were aligned to the human reference genome (GRCh38 primary assembly) using STAR in two-pass mode. Genome indices were generated from the GRCh38 primary assembly FASTA and Ensembl gene annotation (release 115) with splice junction overhang set to 99. Alignments were written as coordinate-sorted BAM files and indexed using samtools. Sequencing and alignment quality were evaluated using FastQC, MultiQC, STAR alignment statistics and Qualimap. Across all samples, a mean of 16.7 million paired-end reads per sample were obtained (range 10.9–21.1 million). On average, 80.3% of reads aligned to the reference genome (range 75.0–85.7%), with 69.7% uniquely mapped reads (range 64.9–76.1%). Average mapped read length was approximately 100–102 bp and mismatch rates were low (~1.2–1.3%). Alignment metrics were consistent across biological replicates, and no technical outliers were observed. Gene-level counts were generated from aligned BAM files using featureCounts (Subread) in paired-end mode with strand-specific counting enabled (forward-stranded; -s 1), using the Ensembl GRCh38.115 annotation and 20 processing threads. Count matrices from separate sequencing runs were combined for downstream statistical analysis. Differential expression analysis was performed in R (version 4.3.3) using DESeq2. A negative binomial generalized linear model was fitted using the design formula ~ batch + condition, where batch corresponded to sequencing run and condition corresponded to treatment. Samples from DMSO treated cells were used as the reference level. Genes with fewer than 10 total reads across all samples were filtered prior to model fitting. Wald tests were used for inference and P values were adjusted for multiple testing using the Benjamini–Hochberg procedure. For effect-size reporting and visualization, log2 fold changes were shrunk using apeglm (lfcShrink), while statistical significance was taken from the corresponding unshrunk DESeq2 results. For sample-level visualization, count data were transformed using the DESeq2 variance stabilizing transformation (VST). Principal component analysis (PCA) was performed on VST-transformed expression values. Sample-to-sample distances were computed as Euclidean distances between VST profiles and visualized as a clustered heatmap. To visualize transcriptional patterns across the DMSO and **35** groups, the top 100 most variable genes (ranked by variance across the selected subset) were extracted from the VST matrix, mean-centered per gene, and displayed as a heatmap. Volcano plots were generated using shrunken log2 fold changes on the x-axis and the corresponding unshrunk adjusted p values on the y-axis. Genes meeting adjusted p value < 0.05 and absolute log2 fold change ≥ |0.3| as the defining inclusion criteria were considered for differential expression analysis. Pathway enrichment analysis was performed using gene set enrichment analysis (GSEA) implementedin the clusterProfiler package. Genes were ranked based on the DESeq2 Wald statistic derived from the differential expression model. Ensembl gene identifiers were mapped to Entrez identifiers, and where multiple genes mapped to the same Entrez ID, the statistic with the largest absolute value was retained. Ranked gene lists were analyzed against KEGG pathway gene sets for Homo sapiens (“hsa”). Enrichment scores were normalized (NES), and p values were adjusted using the Benjamini–Hochberg method.

## Supplementary Material

**Supporting Information**

**Supporting Information** (pdf) containing dose-response curves for compounds **35** and **53** in the Gal4-TLX and Gal4-RORα reporter gene assays (Figure S1), degradation of compounds **35** and **53** by HLM (Figure S2), transcriptomic effects of **35** mapped to selected KEGG pathways (Figures S3-4), and NMR spectra (^1^H, ^13^C, qH) of **10**–**56**, **58** and **59**.

**Molecular formula strings** (csv) containing chemical structures and activity data of **10**–**56**, **58** and **59**.

jm6c00734_si_002

Supporting info.

## Figures and Tables

**Table 1:** Preliminary evaluation of fragment extension

	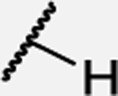 EC_50_ (remain. act.) ^a^ (ID)	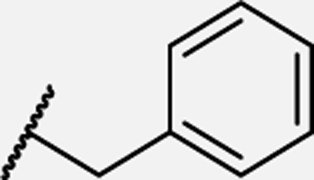 EC_50_ (remain. act.) ^a^ (ID)	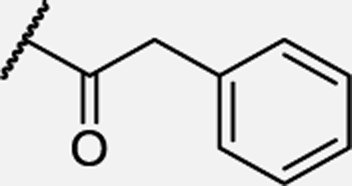 EC_50_ (remain. act.) ^a^ (ID)
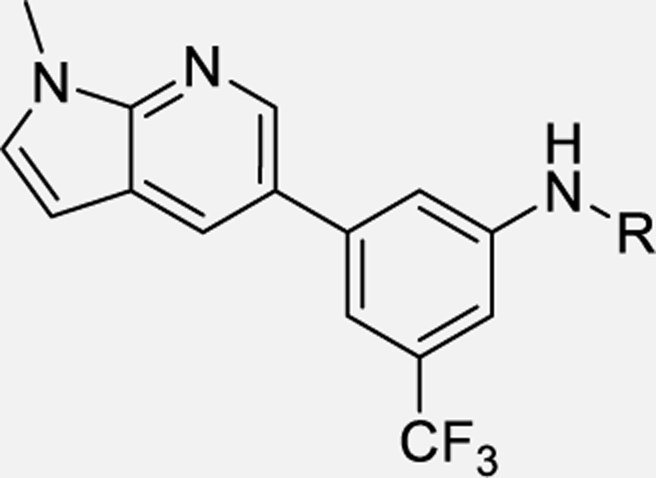	0.7±0.2 μM (0.27±0.06) (**6**)	inactive (10 μM ^b^) (**10**)	0.5±0.2 μM (0.3±0.1) (**14**)
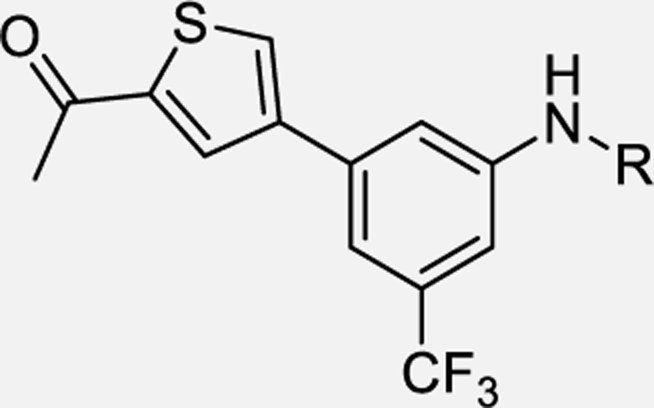	1.5±0.5 μM (0.19±0.06) (**7**)	inactive (10 μM ^b^) (**11**)	0.3±0.1 μM (0.31±0.08) (**15**)
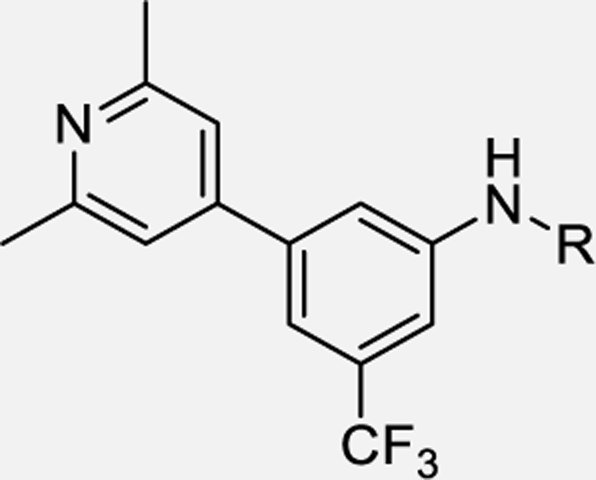	1.6±0.8 μM (0.31±0.08) (**8**)	inactive (10 μM ^b^) (**12**)	0.19±0.02 μM (0.27±0.03) (**16**)
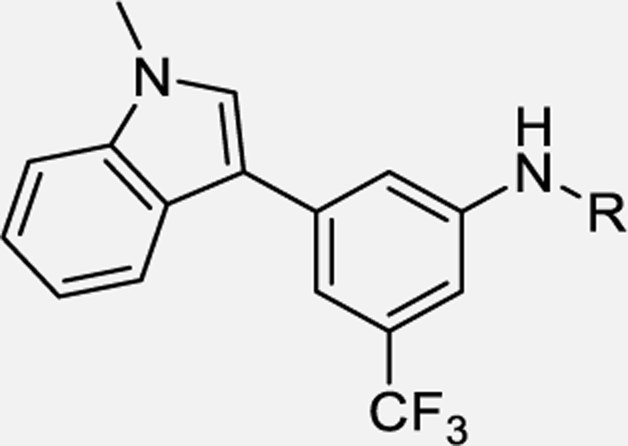	9.1±0.7 μM (0.19±0.05) (**9**)	inactive (10 μM ^b^) (**13**)	0.5±0.1 μM (0.10±0.07) (**17**)

a TLX modulation was determined in a hybrid reporter gene assay measuring the Gal4-TLX-mediated repression of Gal4-VP16-induced reporter activity. Agonism is defined as activation of the receptor’s constitutive repressor activity and observed as a reduction in reporter activity. Remaining activity (remain. act.) refers to the reporter activity vs. DMSO (0.1%) treated cells. Data are the mean±S.E.M., n≥3. All compounds were counterscreened to exclude non-specific effects on Gal4-VP16 activity. Data for **6**-**9** from reference ^25^.

b Highest non-toxic concentration.

**Table 2:** SAR of the acetamide linker

	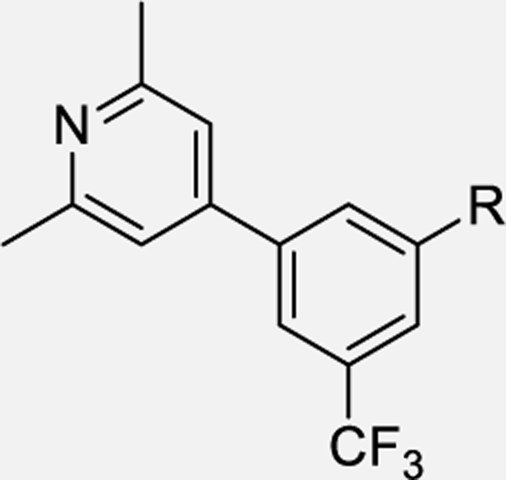 EC_50_ (remain. act.) ^a^ (ID)	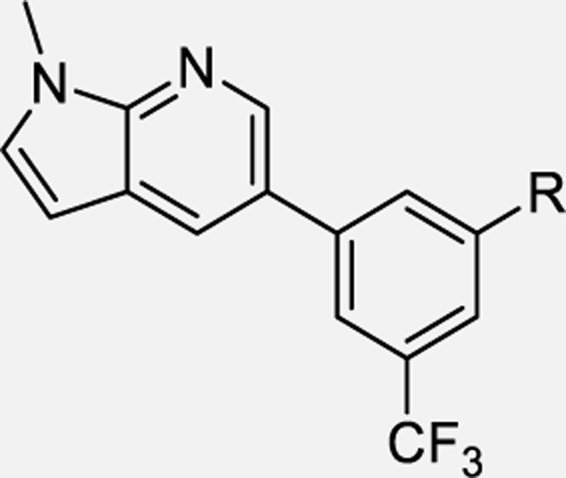 EC_50_ (remain. act.) ^a^ (ID)
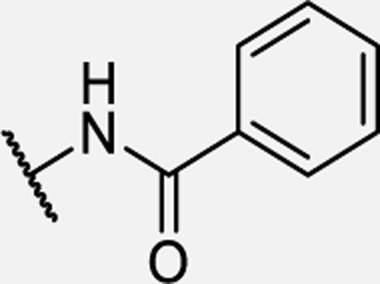	inactive (10 μM ^b^) (**18**)	inactive (10 μM ^b^) (**19**)
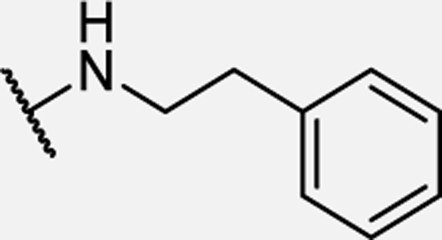	inactive (10 μM ^b^) (**20**)	inactive (10 μM ^b^) (**21**)
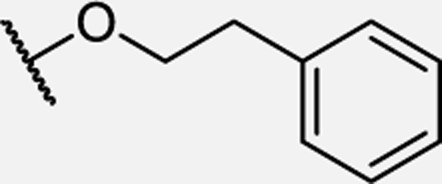	inactive (10 μM ^b^) (**22**)	inactive (10 μM ^b^) (**23**)
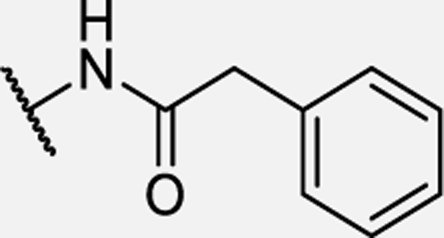	0.19±0.02 μM (0.27±0.03) (**16**)	0.5±0.2 μM (0.3±0.1) (**14**)
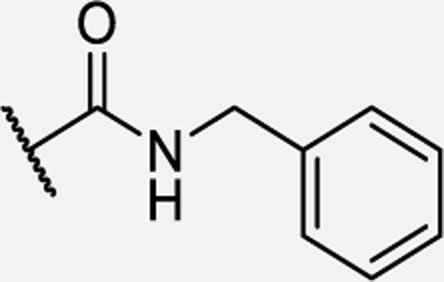	0.40±0.08 μM (0.26±0.06) (**24**)	1.4±0.2 μM (0.31±0.04) (**25**)
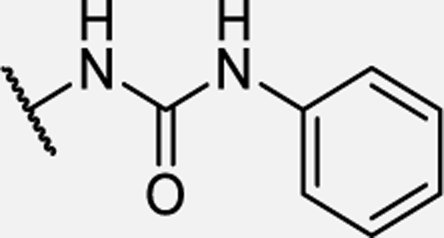	0.5±0.2 μM (0.41±0.09) (**26**)	> 6 μM (0.31±0.05 at 6 μM ^b^) (**27**)
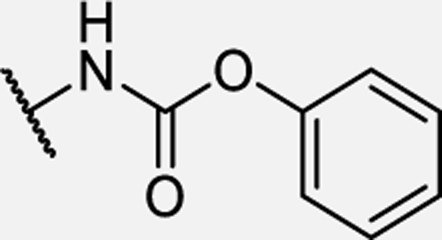	1.2±0.4 μM (0.67±0.06) (**28**)	1.0±0.3 μM (0.56±0.06) (**29**)
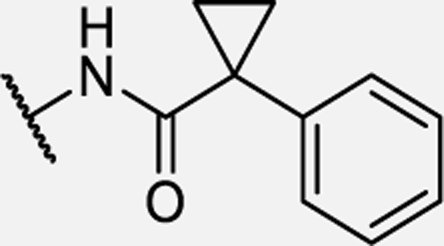	0.7±0.3 μM (0.64±0.08) (**30**)	inactive (10 μM ^b^) (**31**)
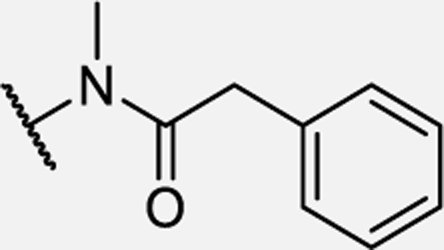	0.16±0.09 μM (0.61±0.08) (**32**)	0.2±0.1 μM (0.63±0.06) (**33**)
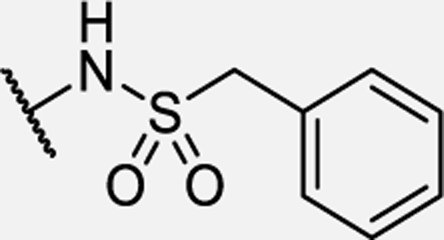	0.4±0.2 μM (0.63±0.08) (**34**)	0.010±0.006 μM (0.65±0.04) (**35**)
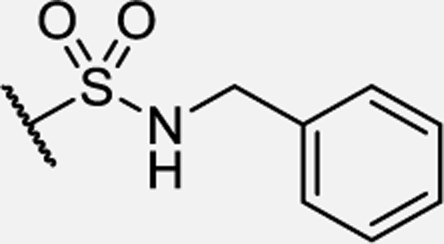	0.28±0.05 μM (0.60±0.04) (**36**)	0.32±0.08 μM (0.47±0.04) (**37**)
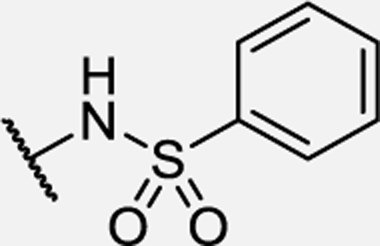	0.6±0.2 μM (0.4±0.1) (**38**)	3±1 μM (0.5±0.1) (**39**)

a TLX modulation was determined in a hybrid reporter gene assay measuring the Gal4-TLX-mediated repression of Gal4-VP16-induced reporter activity. Agonism is defined as activation of the receptor’s constitutive repressor activity and observed as a reduction in reporter activity. Remaining activity (remain. act.) refers to the reporter activity vs. DMSO (0.1%) treated cells. Data are the mean±S.E.M., n≥3. All compounds were counterscreened to exclude non-specific effects on Gal4-VP16 activity.

b Highest non-toxic concentration.

**Table 3:** Evaluation of substituents on the phenylacetamide

ID	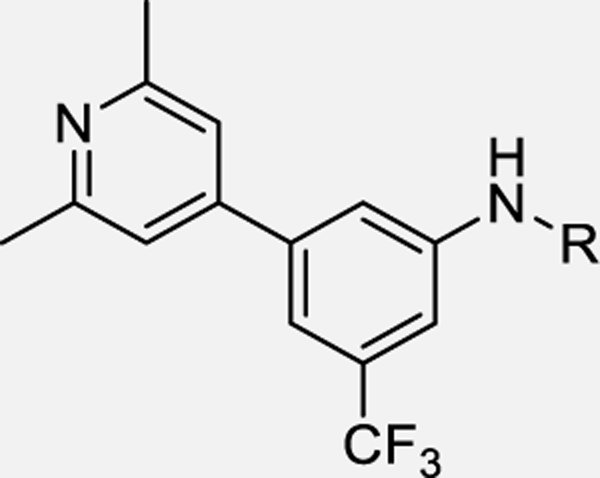 R=	EC_50_ (TLX) (remain. Act.) ^a^
**8**	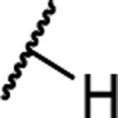	1.6±0.8 μM (0.31±0.08)
**16**	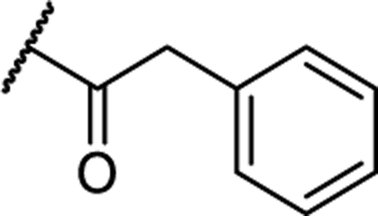	0.19±0.02 μM (0.27±0.03)
**40**	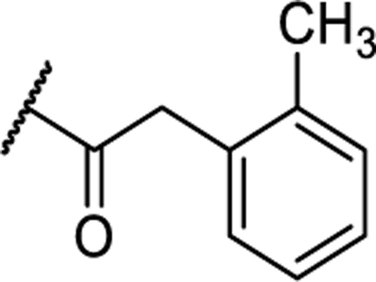	0.27±0.09 μM (0.28±0.08)
**41**	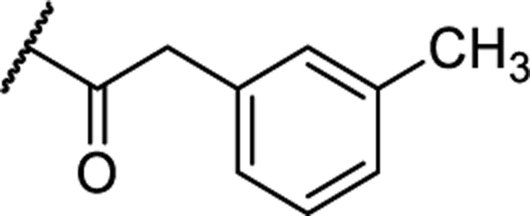	0.4±0.1 μM (0.43±0.05)
**42**	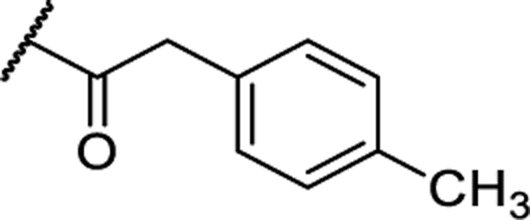	0.4±0.1 μM (0.57±0.03)
**43**	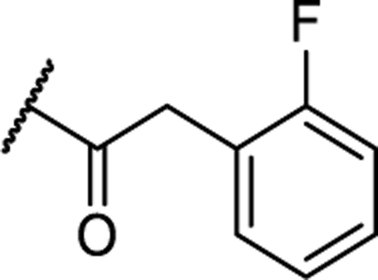	0.17±0.07 μM (0.24±0.08)
**44**	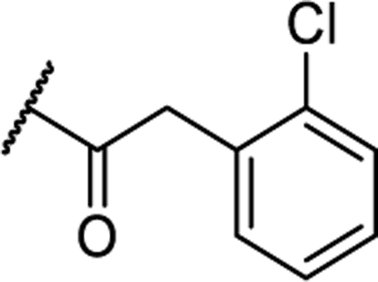	0.16±0.06 μM (0.58±0.06)
**45**	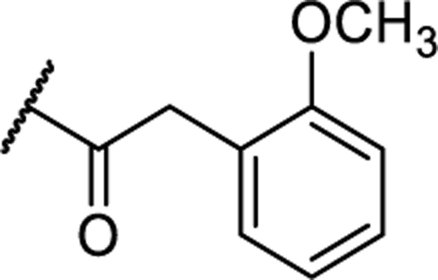	0.21±0.09 μM (0.36±0.04)

a TLX modulation was determined in a hybrid reporter gene assay measuring the Gal4-TLX-mediated repression of Gal4-VP16-induced reporter activity. Agonism is defined as activation of the receptor’s constitutive repressor activity and observed as a reduction in reporter activity. Remaining activity (remain. act.) refers to the reporter activity vs. DMSO (0.1%) treated cells. Data are the mean±S.E.M., n≥3. All compounds were counterscreened to exclude non-specific effects on Gal4-VP16 activity.

**Table 4:** Double substitution on the phenylacetamide

ID	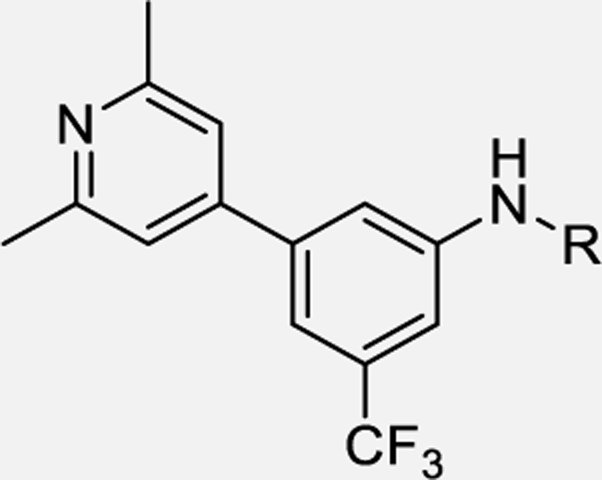 R=	EC_50_ (TLX) (remain. Act.) ^a^
**46**	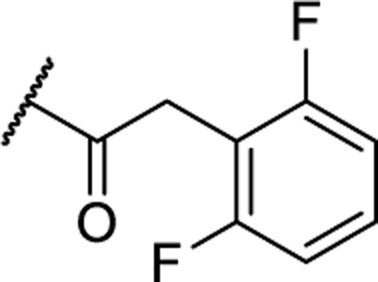	0.04±0.01 μM (0.34±0.03)
**47**	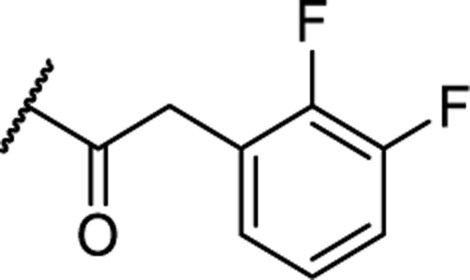	0.38±0.09 μM (0.41±0.04)
**48**	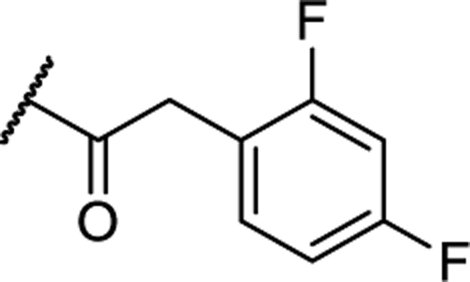	0.22±0.12 μM (0.33±0.11)
**49**	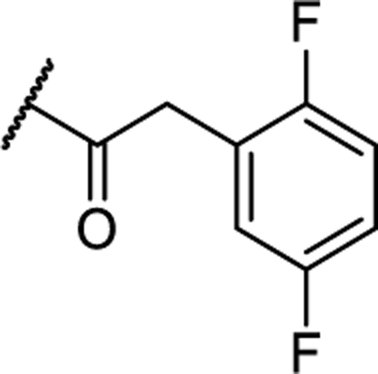	0.13±0.04 μM (0.38±0.07)
**50**	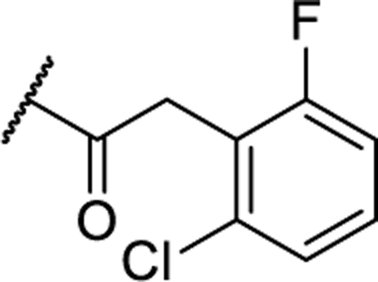	0.11±0.04 μM (0.45±0.05)
**51**	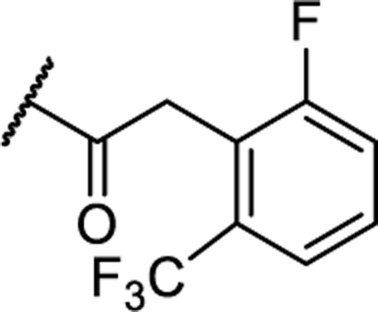	0.05±0.01 μM (0.61±0.05)
**52**	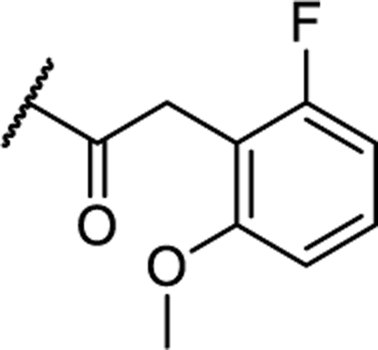	0.04±0.01 μM (0.53±0.02)
**53**	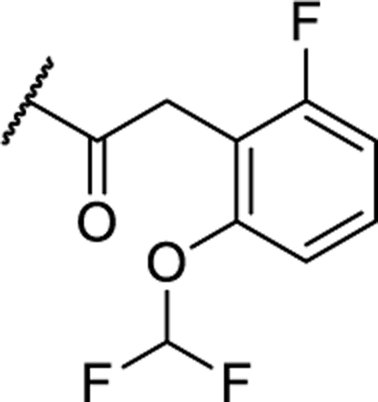	0.015±0.004 μM (0.56±0.04)

a TLX modulation was determined in a hybrid reporter gene assay measuring the Gal4-TLX-mediated repression of Gal4-VP16-induced reporter activity. Agonism is defined as activation of the receptor’s constitutive repressor activity and observed as a reduction in reporter activity. Remaining activity (remain. act.) refers to the reporter activity vs. DMSO (0.1%) treated cells. Data are the mean±S.E.M., n≥3. All compounds were counterscreened to exclude non-specific effects on Gal4-VP16 activity.

**Table 5:** SAR transfer to the phenylmethanesulfonamide 35

ID	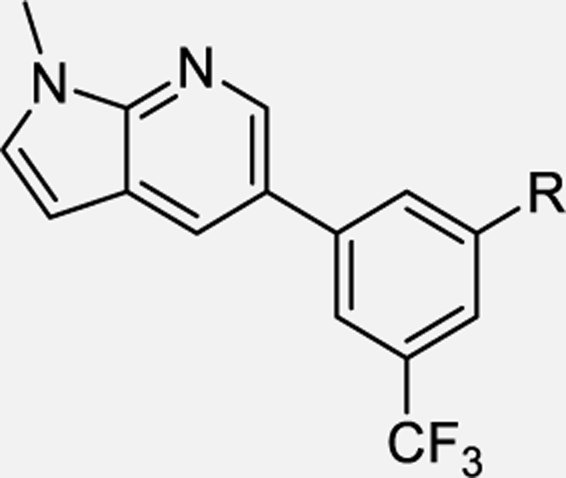 R=	EC_50_ (TLX) (remain. Act.) ^a^
**35**	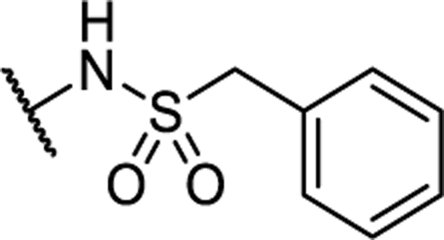	0.010±0.006 μM (0.65±0.04)
**54**	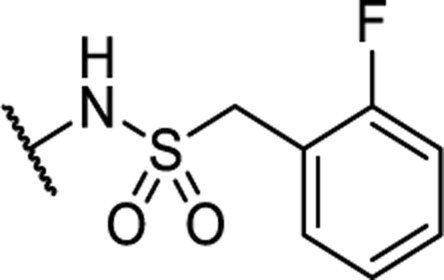	0.03±0.01 μM (0.57±0.04)
**55**	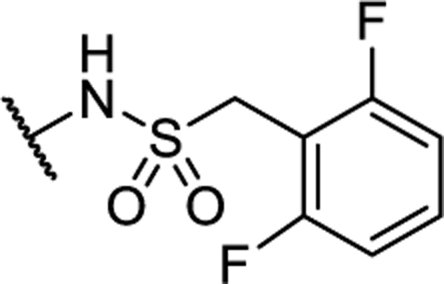	0.18±0.09 μM (0.78±0.06)
**56**	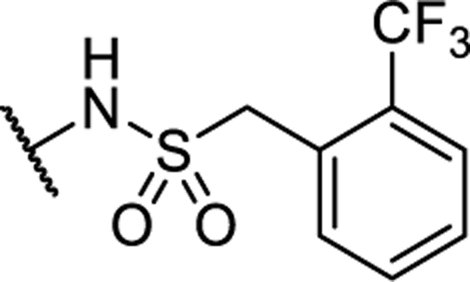	0.6±0.2 μM (0.65±0.06)

a TLX modulation was determined in a hybrid reporter gene assay measuring the Gal4-TLX-mediated repression of Gal4-VP16-induced reporter activity. Agonism is defined as activation of the receptor’s constitutive repressor activity and observed as a reduction in reporter activity. Remaining activity (remain. act.) refers to the reporter activity vs. DMSO (0.1%) treated cells. Data are the mean±S.E.M., n≥3. All compounds were counterscreened to exclude non-specific effects on Gal4-VP16 activity.

**Table 6:** SAR transfer to inverse agonist fragment 57

ID	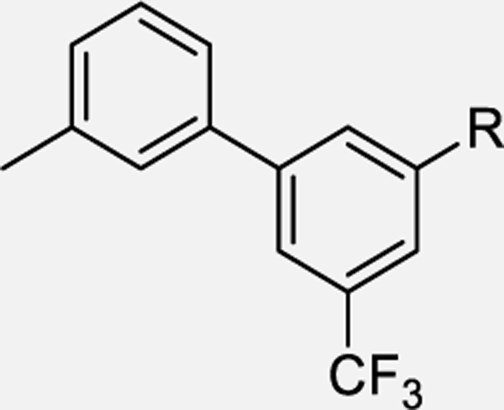 R=	IC_50_ (TLX) (remain act./fold act.) ^a^
**57**	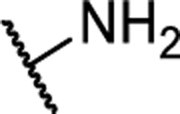	16±3 (2.5±0.1 fold)^25^
**58**	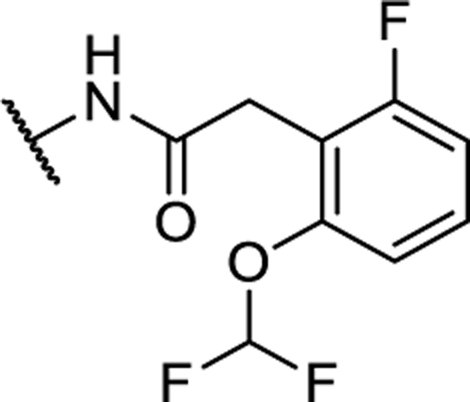	inactive (10 μM^b^)
**59**	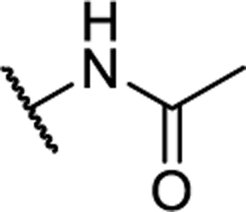	agonist (0.6±0.2 remain. At 10 μM ^b^)

a TLX modulation was determined in a hybrid reporter gene assay measuring the Gal4-TLX-mediated repression of Gal4-VP16-induced reporter activity. Agonism is defined as activation of the receptor’s constitutive repressor activity and observed as a reduction in reporter activity. Inverse agonism is defined as inhibition of the receptor’s constitutive repressor activity and observed as increase in reporter activity. Remaining activity (remain. act.) and fold activation (fold act.) refer to the reporter activity vs. DMSO (0.1%) treated cells. Data are the mean±S.E.M., n≥3. All compounds were counterscreened to exclude non-specific effects on Gal4-VP16 activity.

b Highest non-toxic concentration.

**Table 7:** Activity profiles of the TLX agonists 35 and 53

	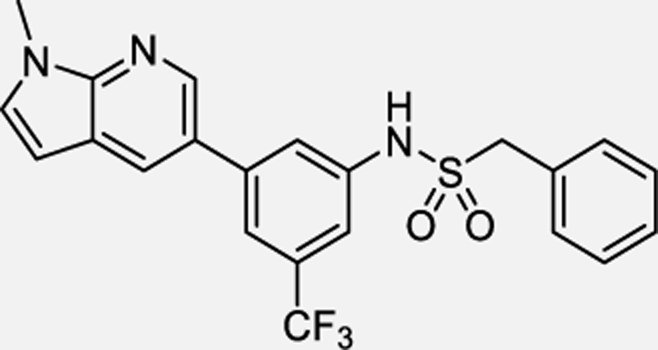 35	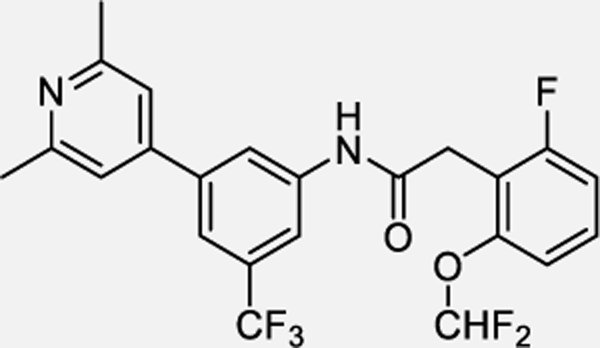 53
TLX agonism ^a^	EC_50_ 0.010±0.006 μM (0.65±0.04 remain.)	EC_50_ 0.015±0.004 μM (0.56±0.04 remain.)
NR selectivity ^b^	> 30-fold	active at 3 μM on TR2, TR4, PNR
inverse RORα agonism ^b^	IC_50_ 8±3 μM	IC_50_ > 3 μM
microsomal stability (t_1/2_) ^c^	113±27 min	114±3 min

a From Gal4-TLX hybrid assay (dose-response curves for TLX agonism in Figure S1).

b Nuclear receptor selectivity was determined in Gal4-hybrid reporter gene assays for THRα, RARα, PPARα/γ/δ, revERBα, RORα, VDR, PXR, CAR, LXRα, FXR, HNF4α, Erα, AR, RXRα/β/γ, TR2, TR4, PNR, SF1, LRH1, Nur77, Nurr1 and NOR1, n≥3 (Figure 2a; dose-response curves for inverse RORα agonism in Figure S1). ^c^ Stability against degradation by human liver microsomes (HLM) was determined by LC-MS/MS; data are the mean±S.E.M., n=3 (degradation curves in Figure S2).

## Abbreviations Used

ASAH1: acid ceramidase
ASMS: affinity-selection-mass-spectrometry
BACE: β-secretase
BLOC1: biogenesis of lysosome-related organelles complex-1
GSEA: gene set enrichment analysis
HTRF: homogenous time-resolved fluorescence resonance energy transfer
LBD: ligand binding domain
NR: nuclear receptor
NSC: neural stem cells
PCA: principal component analysis
PINK1: PTEN-induced kinase 1
pten: phosphatase and tensin homologue
RNAseq: RNA sequencing
ROR: retinoic acid receptor-related orphan receptor
RPC: retinal progenitor cells
SNAP23: synaptosome-associated protein of 23 kDa
TLX: tailless homologue
V-ATPase: vacuolar-type H+-adenosine triphosphatase.
