# Supplementary material for: An activator of tailless’ repressor function stimulates anti-neurodegenerative gene expression
Source: J Med Chem. Author manuscript; Available in PMC 2026 Oct 11. (PMC7619541; doi:10.1021/acs.jmedchem.6c00734)
Supplement: Supplementary file 1 [file EMS215449-supplement-Supporting_info_.pdf]

- Supporting Information -

An activator of tailless' repressor function stimulates anti-neurodegenerative gene expression

Emily C. Hank[1], Romy Busch[1], Úrsula López-García[1], Till Kasch[1], Loris Knümann[1], Vasily Morozov[1], Georg Höfner[1], Julian A. Marschner[1], Daniel Merk[1]*

[1] Ludwig-Maximilians-Universität (LMU) München, Department of Pharmacy, 81377 Munich, Germany
* daniel.merk@cup.lmu.de

Table of Contents

Supplementary Figures S2
NMR spectra of compounds **10-56**, **58** and **59**.. S5
Supplementary References S79

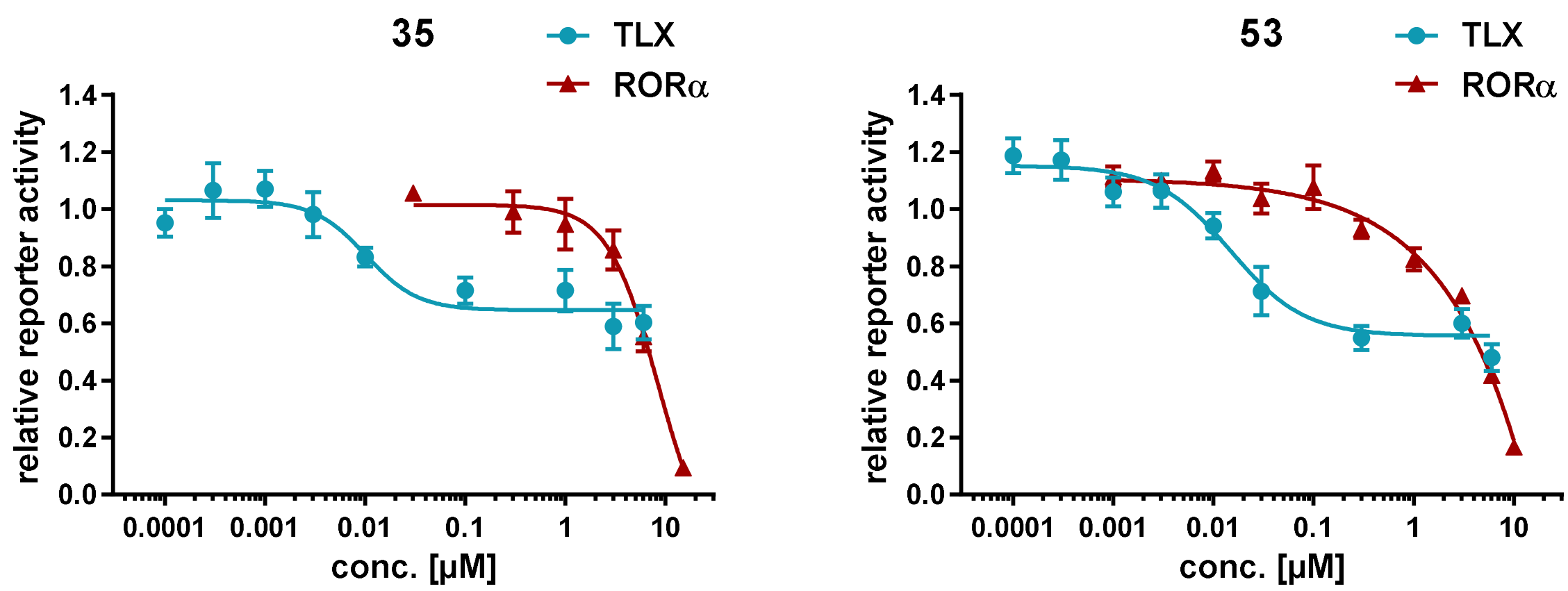

Figure S1. Dose-response curves of compounds 35 and 53 in the Gal4-TLX and Gal4-RORα hybrid reporter gene assays. Relative reporter activity as fold over the DMSO-control (0.1% DMSO: relative reporter activity = 1). Data are the mean±S.E.M.; *n* ≥3.

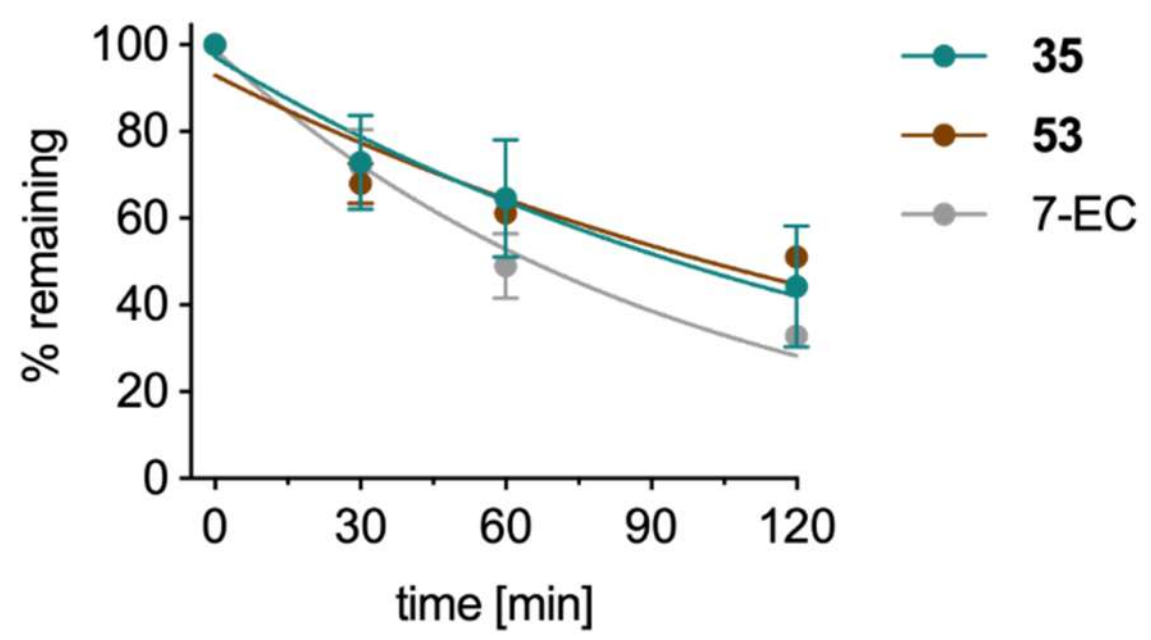

Figure S2. In vitro metabolic stability evaluation of 35 and 53 using human liver microsomes (HLM). 7-Ethoxycoumarin (7-EC) for comparison. Data are the mean±S.E.M., n=3.

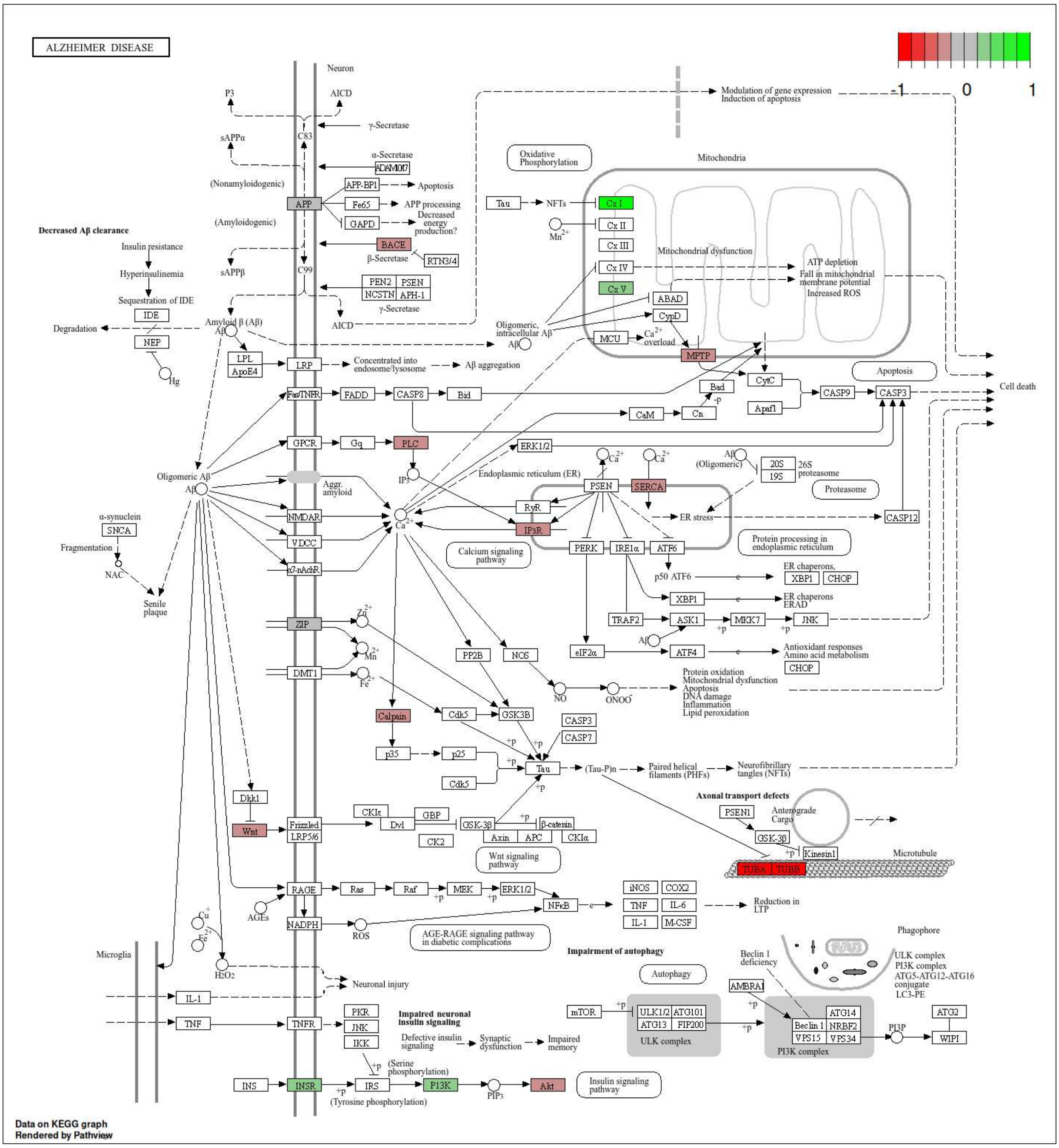

Figure S3. Transcriptomic effects of compound 35 mapped on the KEGG pathway[1] 'Alzheimer Disease' (5010).

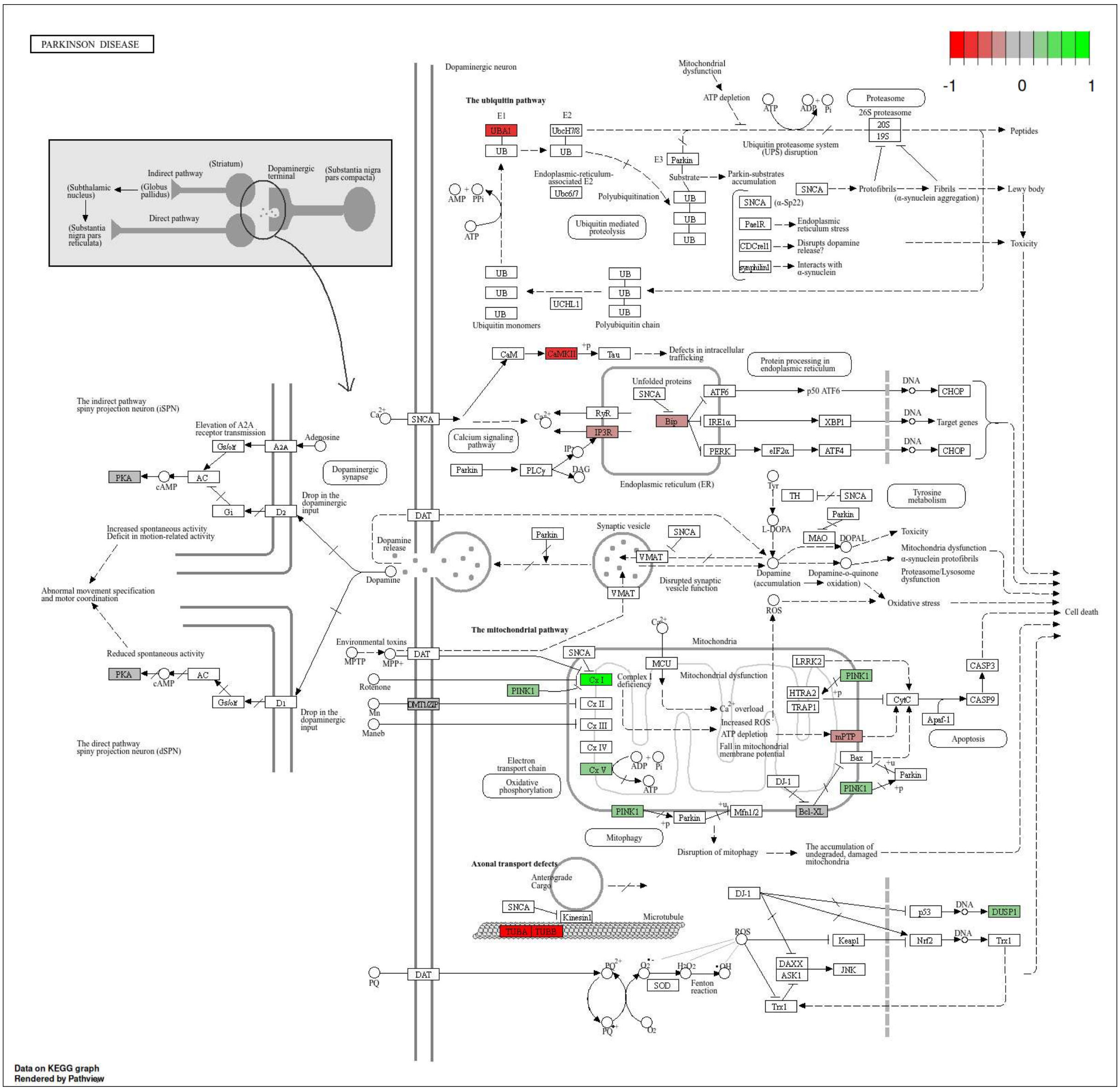

Figure S4. **Transcriptomic effects of compound 35 mapped on the KEGG pathway[1] 'Parkinson Disease' (5012).**

NMR spectra (^{1}H, ^{13}C, qH NMR) of **10-56**, **58** and **59**:

Compound **10**:

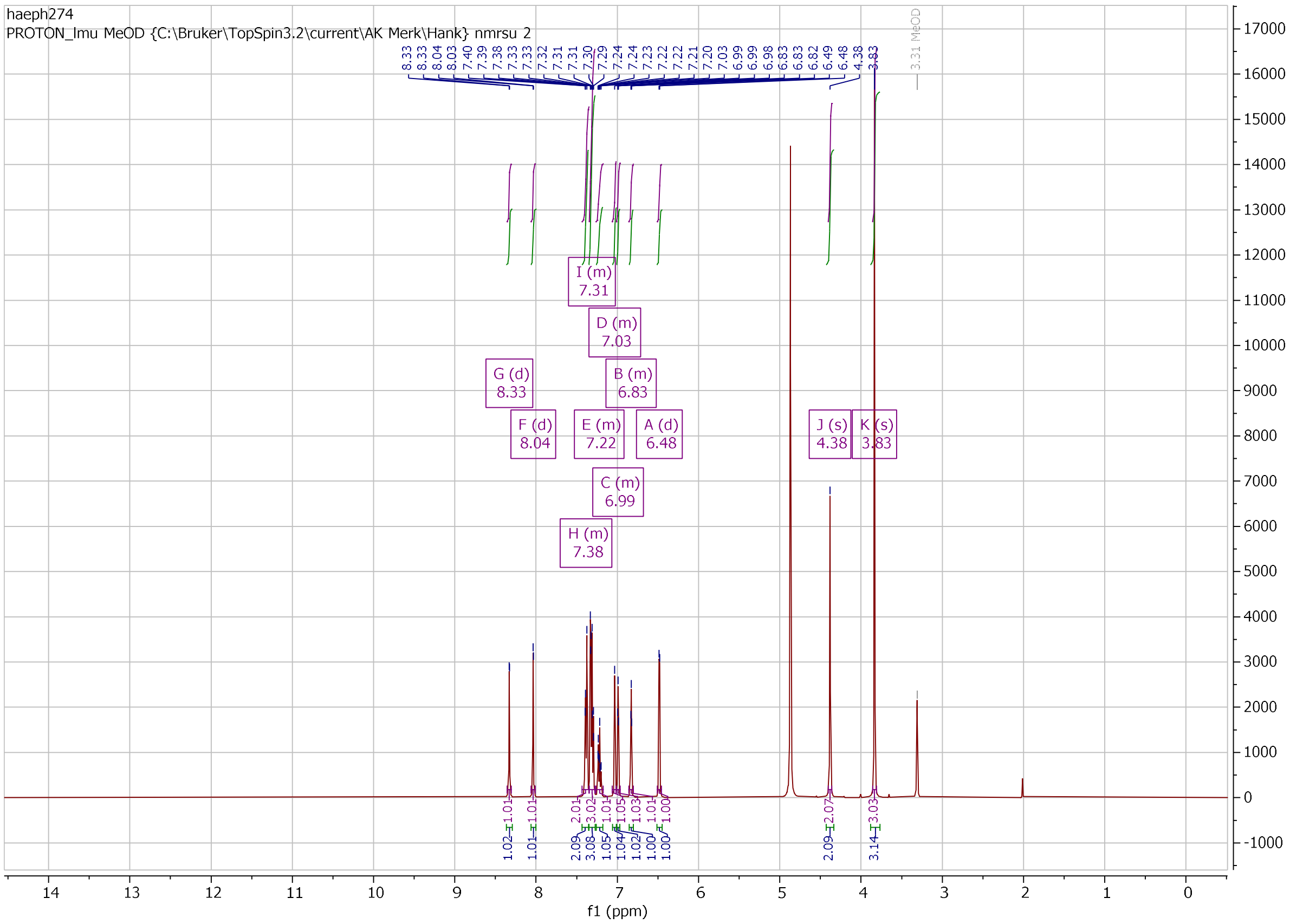

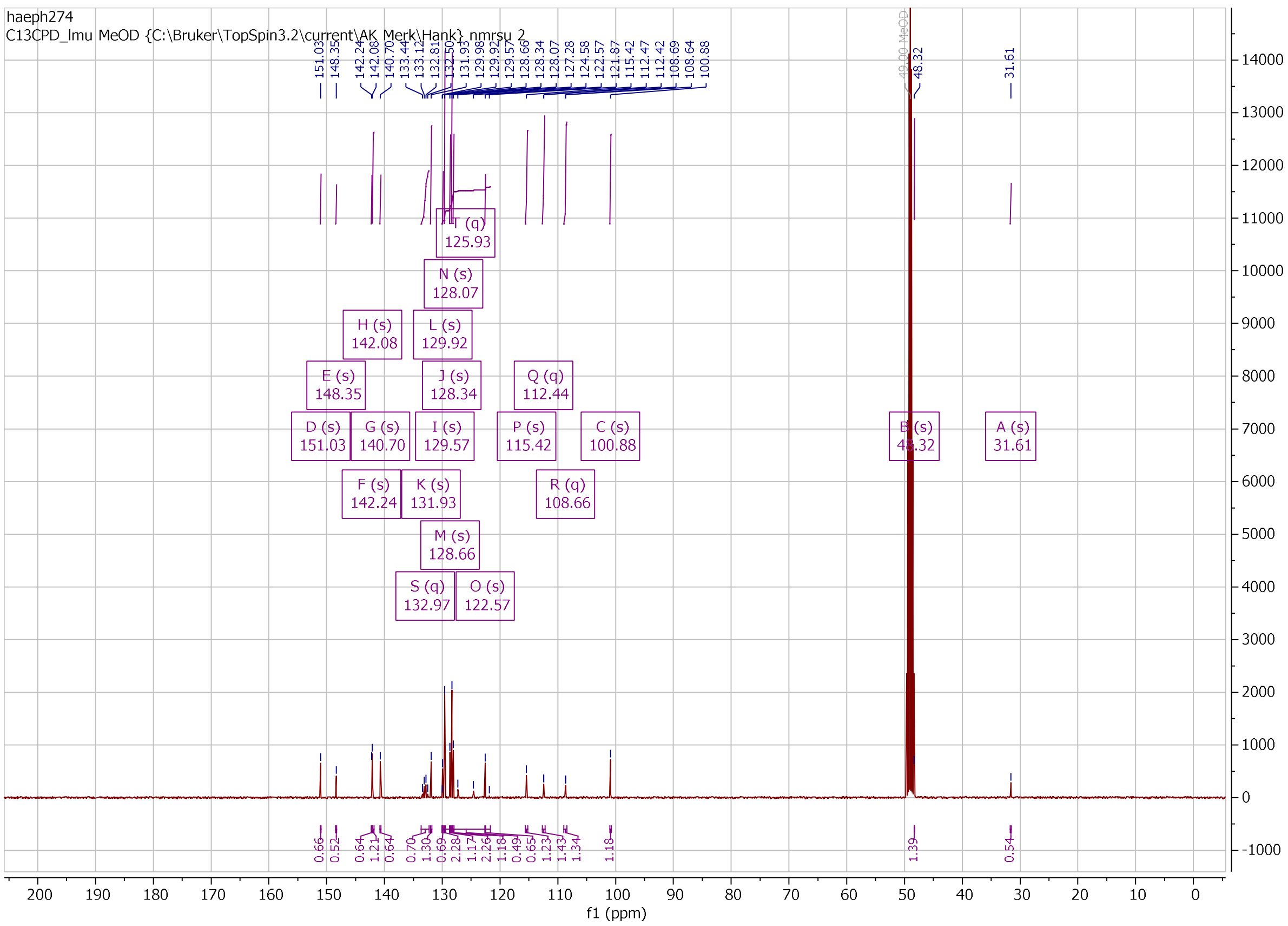

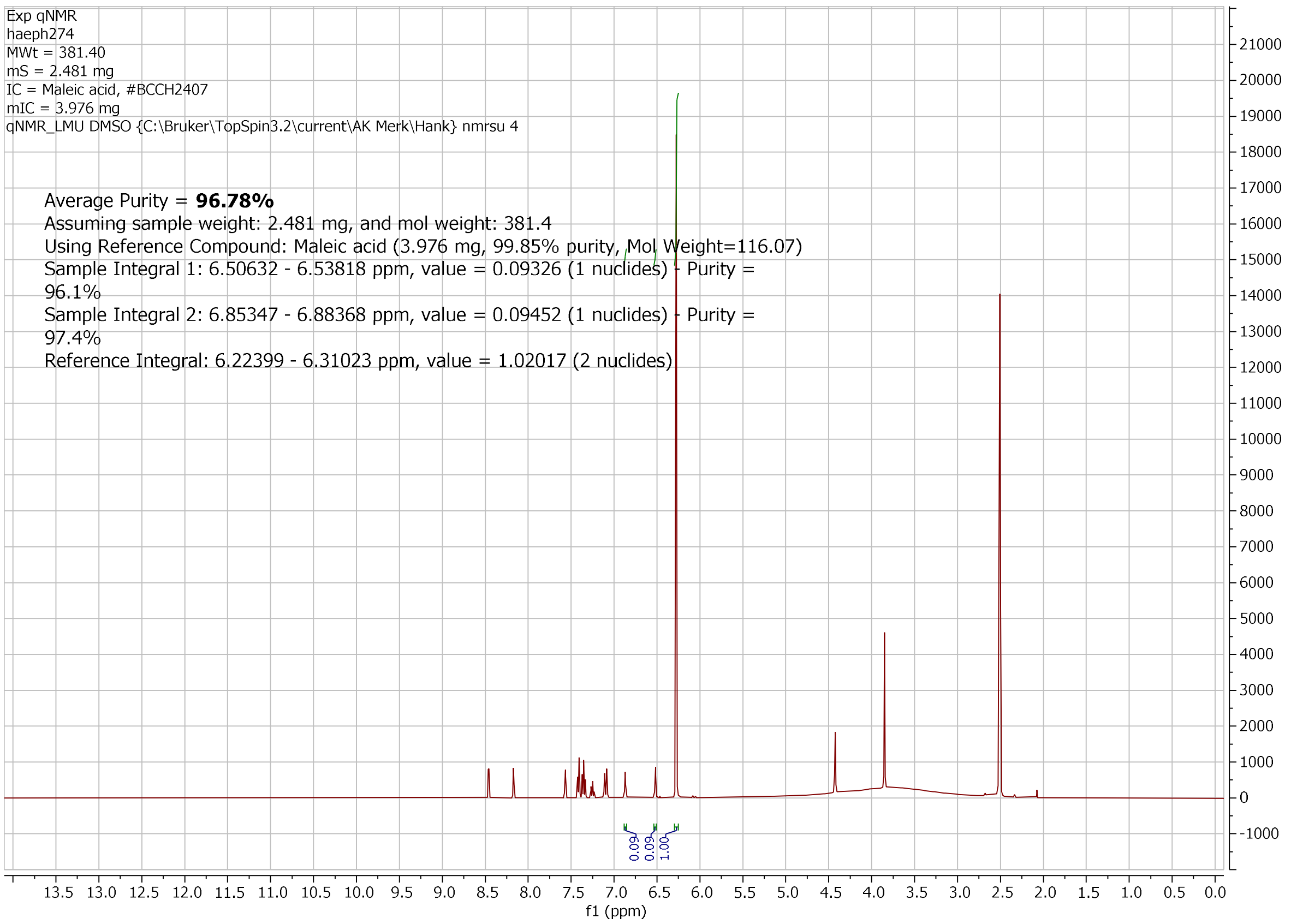
Exp qNMR
haeph274
MWt = 381.40
mS = 2.481 mg
IC = Maleic acid, #BCCH2407
mIC = 3.976 mg
qNMR_LMU DMSO {C:\Bruker\TopSpin3.2\current\AK Merk\Hank} nmrsu 4
Average Purity = **96.78%**
Assuming sample weight: 2.481 mg, and mol weight: 381.4
Using Reference Compound: Maleic acid (3.976 mg, 99.85% purity, Mol Weight=116.07)
Sample Integral 1: 6.50632 - 6.53818 ppm, value = 0.09326 (1 nuclides) - Purity = 96.1%
Sample Integral 2: 6.85347 - 6.88368 ppm, value = 0.09452 (1 nuclides) - Purity = 97.4%
Reference Integral: 6.22399 - 6.31023 ppm, value = 1.02017 (2 nuclides)
f1 (ppm)

Compound **11**:

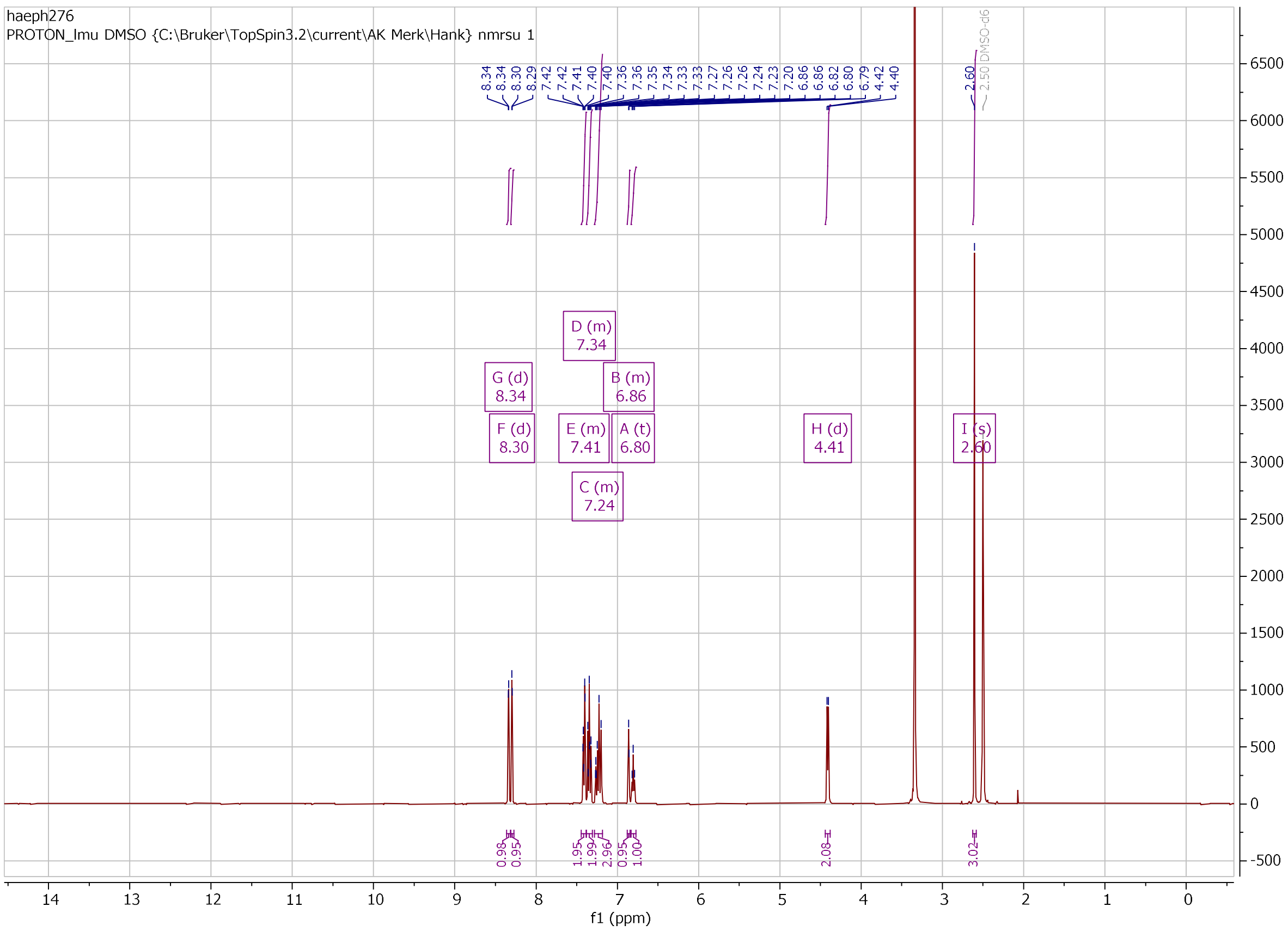
haeph276
PROTON_lmu DMSO {C:\Bruker\TopSpin3.2\current\AK Merk\Hank} nmrsu 1
D (m) 7.34
G (d) 8.34
B (m) 6.86
F (d) 8.30
E (m) 7.41
A (t) 6.80
H (d) 4.41
I (s) 2.60
C (m) 7.24
f1 (ppm)

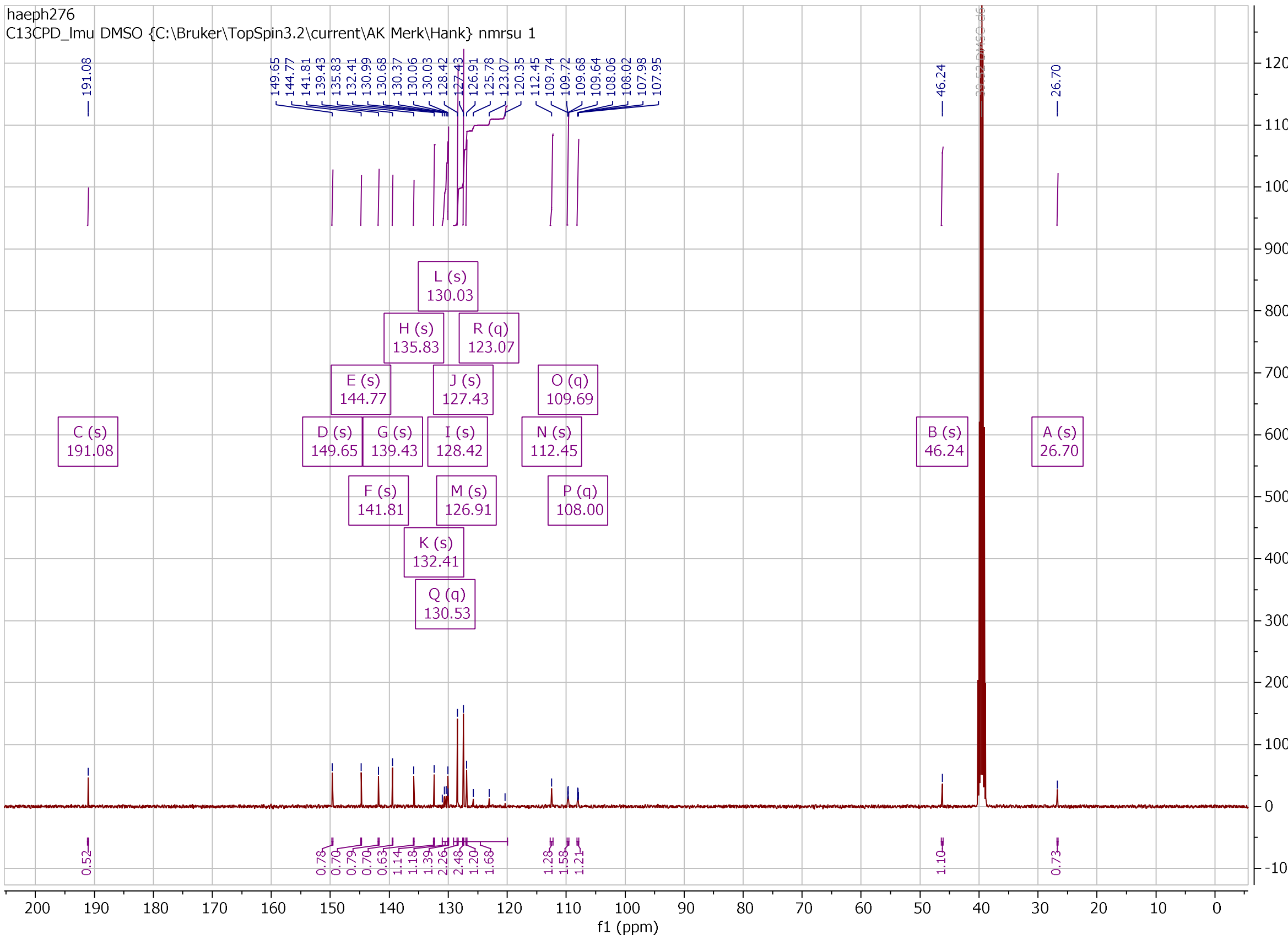
haeph276
C13CPD_lmu DMSO {C:\Bruker\TopSpin3.2\current\AK Merk\Hank} nmrsu 1
L (s) 130.03
H (s) 135.83
R (q) 123.07
E (s) 144.77
J (s) 127.43
O (q) 109.69
C (s) 191.08
D (s) 149.65
G (s) 139.43
I (s) 128.42
N (s) 112.45
B (s) 46.24
A (s) 26.70
F (s) 141.81
M (s) 126.91
P (q) 108.00
K (s) 132.41
Q (q) 130.53
f1 (ppm)

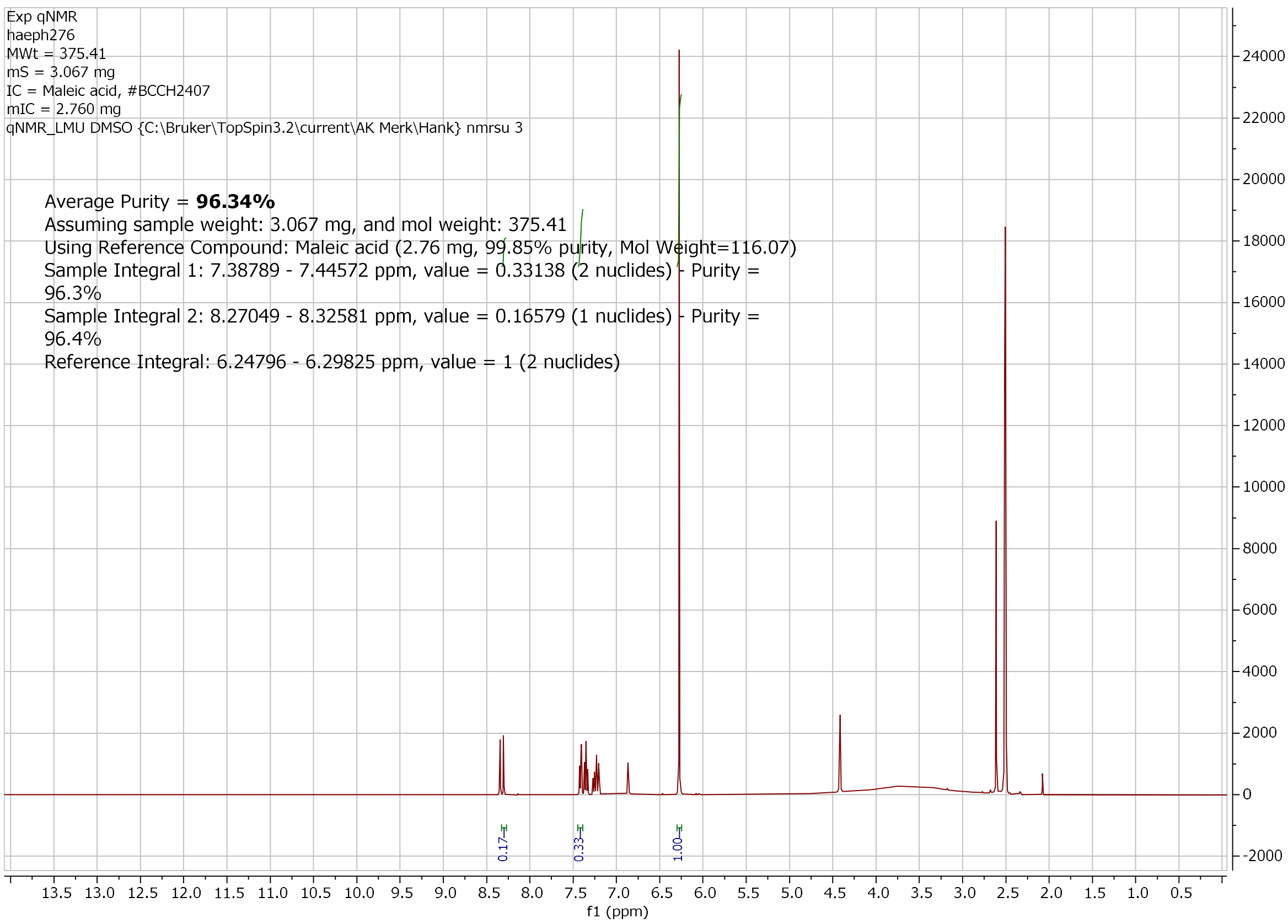
Exp qNMR
haeph276
MWt = 375.41
mS = 3.067 mg
IC = Maleic acid, #BCCH2407
mIC = 2.760 mg
qNMR_LMU DMSO {C:\Bruker\TopSpin3.2\current\AK Merk\Hank} nmrsu 3
Average Purity = 96.34%
Assuming sample weight: 3.067 mg, and mol weight: 375.41
Using Reference Compound: Maleic acid (2.76 mg, 99.85% purity, Mol Weight=116.07)
Sample Integral 1: 7.38789 - 7.44572 ppm, value = 0.33138 (2 nuclides) - Purity = 96.3%
Sample Integral 2: 8.27049 - 8.32581 ppm, value = 0.16579 (1 nuclides) - Purity = 96.4%
Reference Integral: 6.24796 - 6.29825 ppm, value = 1 (2 nuclides)
f1 (ppm)

Compound **12**:

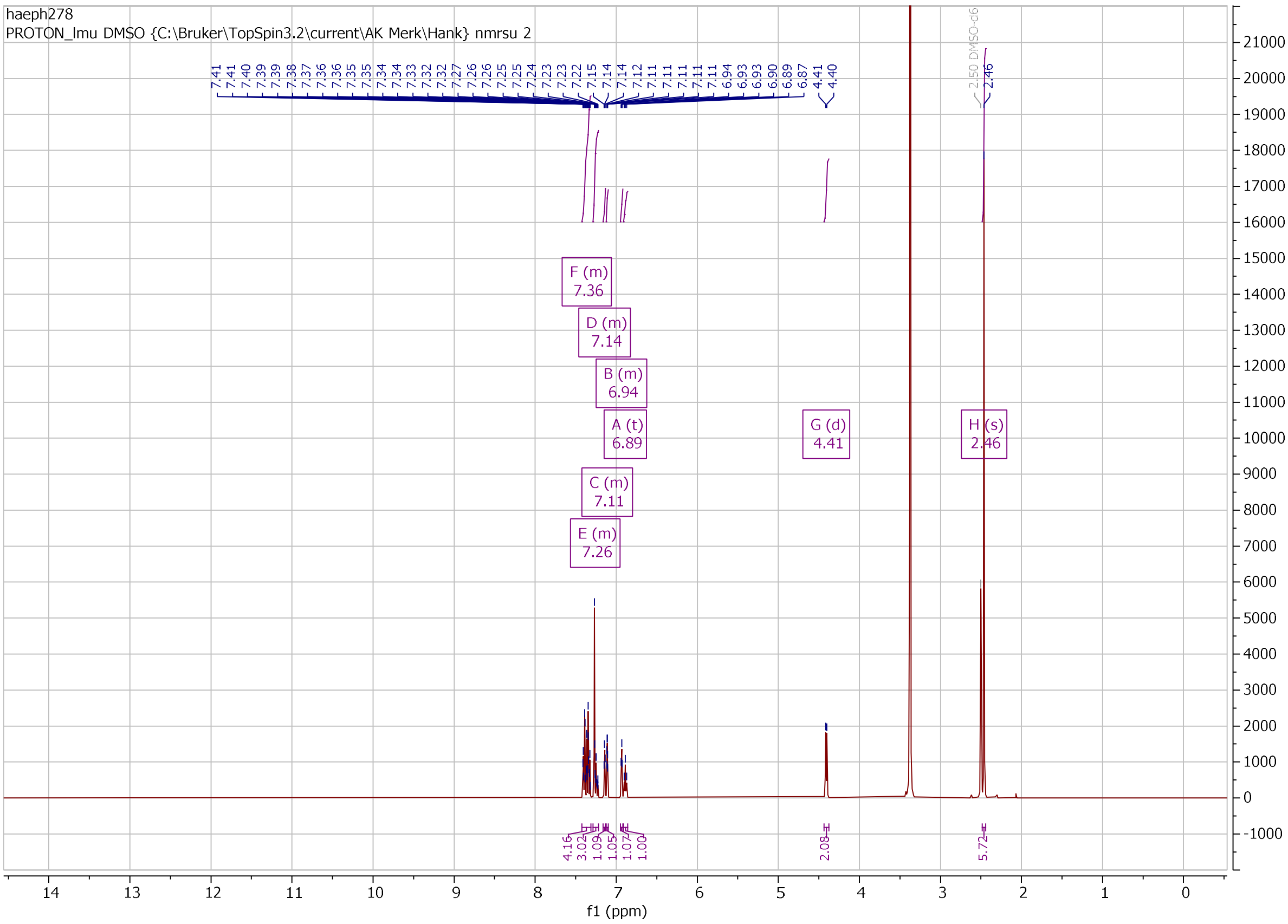
haeph278
PROTON_Imu DMSO {C:\Bruker\TopSpin3.2\current\AK Merk\Hank} nmrsu 2
F (m) 7.36
D (m) 7.14
B (m) 6.94
A (t) 6.89
C (m) 7.11
E (m) 7.26
G (d) 4.41
H (s) 2.46
f1 (ppm)

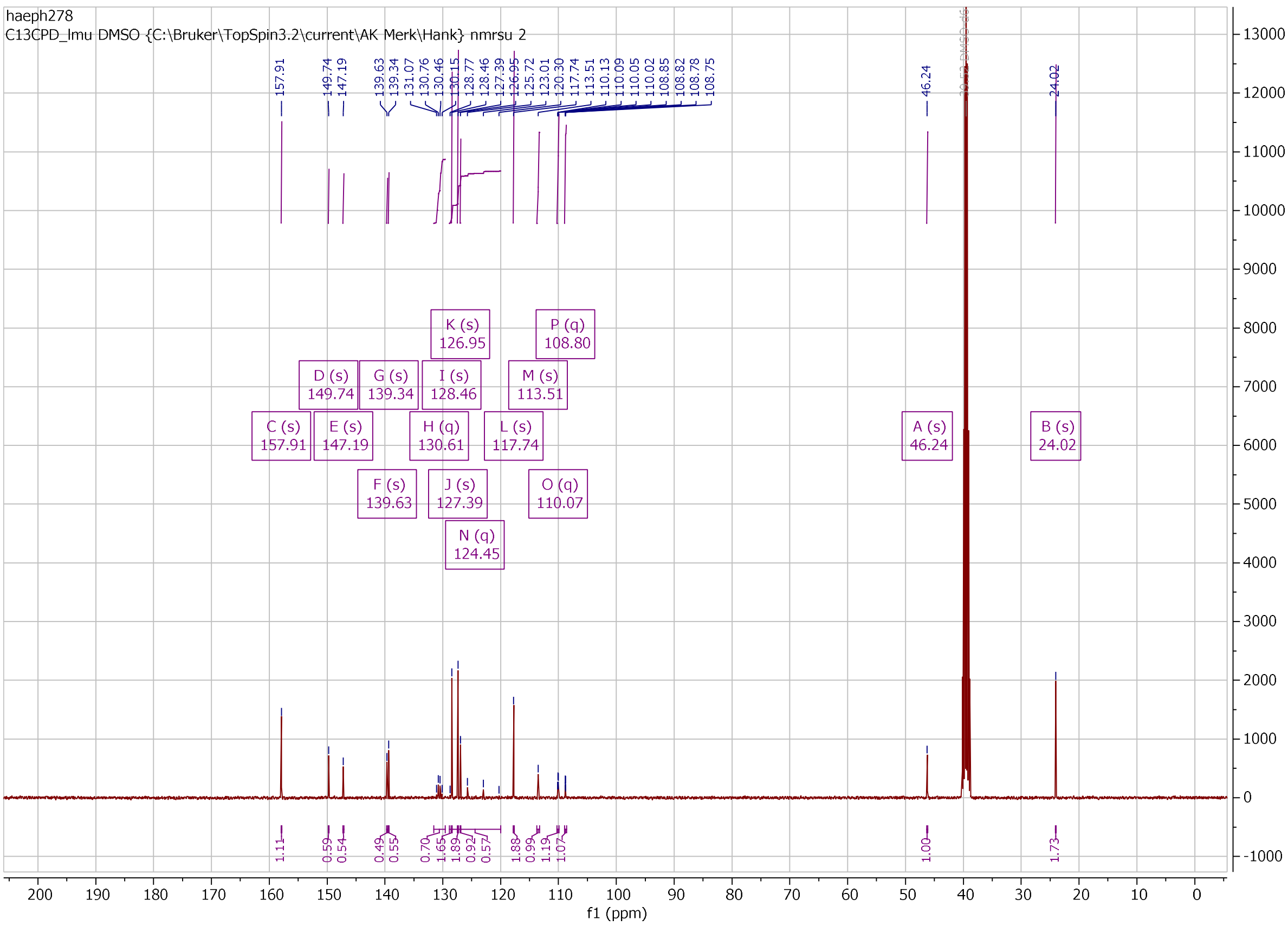
haeph278
C13CPD_Imu DMSO {C:\Bruker\TopSpin3.2\current\AK Merk\Hank} nmrsu 2
K (s) 126.95
P (q) 108.80
D (s) 149.74
G (s) 139.34
I (s) 128.46
M (s) 113.51
C (s) 157.91
E (s) 147.19
H (q) 130.61
L (s) 117.74
A (s) 46.24
B (s) 24.02
F (s) 139.63
J (s) 127.39
O (q) 110.07
N (q) 124.45
f1 (ppm)

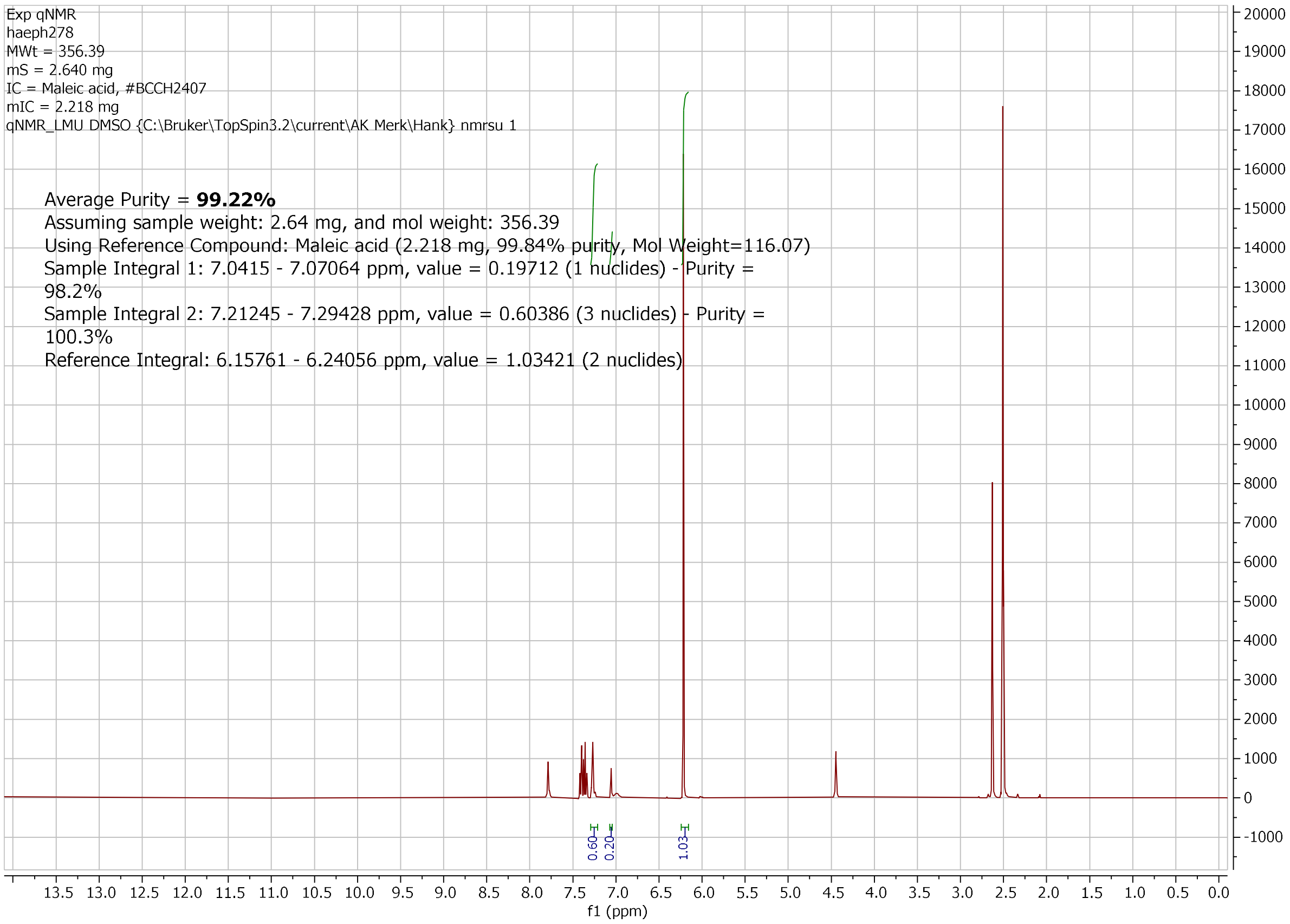
Exp qNMR
haeph278
MWt = 356.39
mS = 2.640 mg
IC = Maleic acid, #BCCH2407
mIC = 2.218 mg
qNMR_LMU DMSO {C:\Bruker\TopSpin3.2\current\AK Merk\Hank} nmrsu 1
Average Purity = 99.22%
Assuming sample weight: 2.64 mg, and mol weight: 356.39
Using Reference Compound: Maleic acid (2.218 mg, 99.84% purity, Mol Weight=116.07)
Sample Integral 1: 7.0415 - 7.07064 ppm, value = 0.19712 (1 nuclides) - Purity = 98.2%
Sample Integral 2: 7.21245 - 7.29428 ppm, value = 0.60386 (3 nuclides) - Purity = 100.3%
Reference Integral: 6.15761 - 6.24056 ppm, value = 1.03421 (2 nuclides)
f1 (ppm)

Compound **13**:

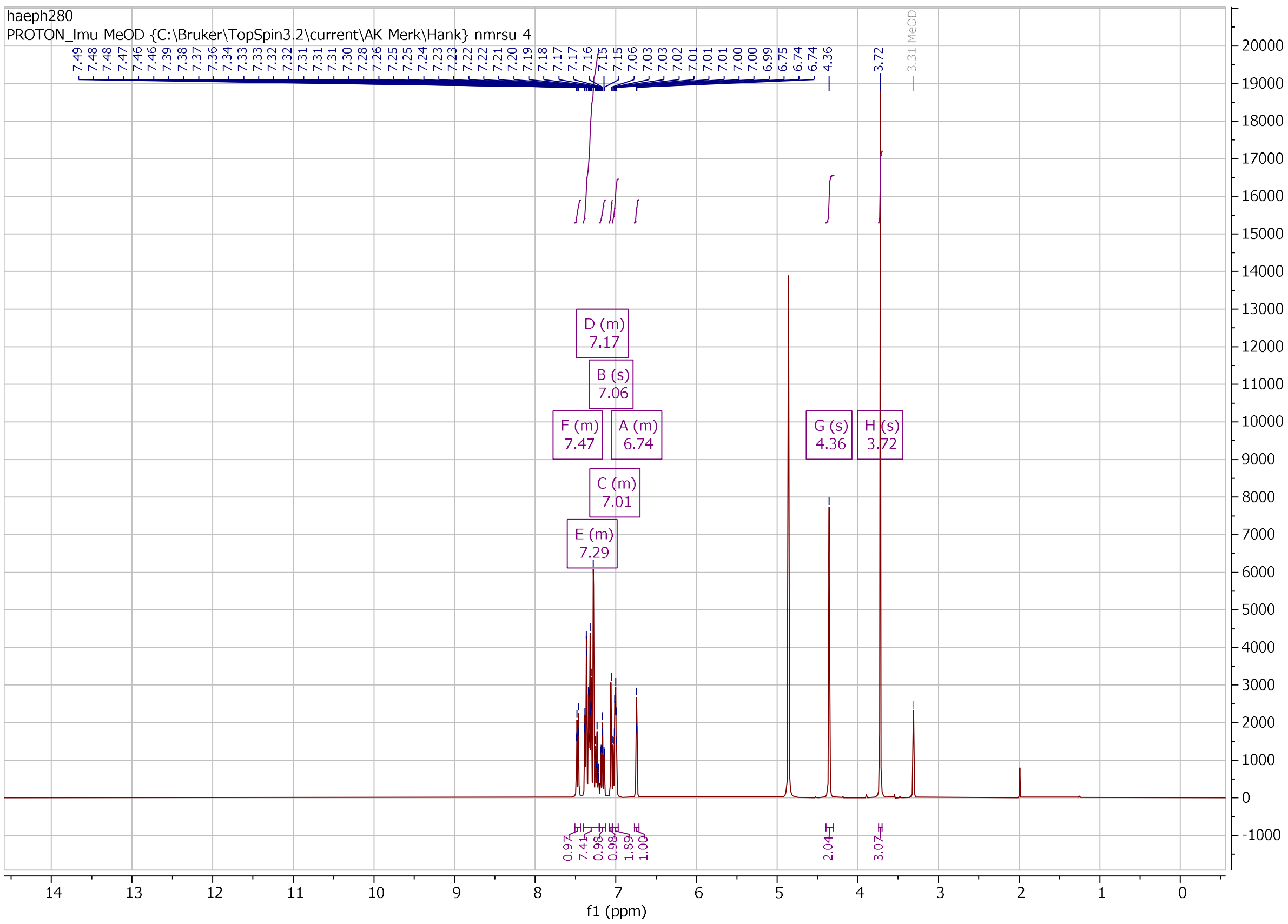
haeph280
PROTON_lmu MeOD {C:\Bruker\TopSpin3.2\current\AK Merk\Hank} nmrsu 4
D (m) 7.17
B (s) 7.06
F (m) 7.47
A (m) 6.74
C (m) 7.01
E (m) 7.29
G (s) 4.36
H (s) 3.72
f1 (ppm)

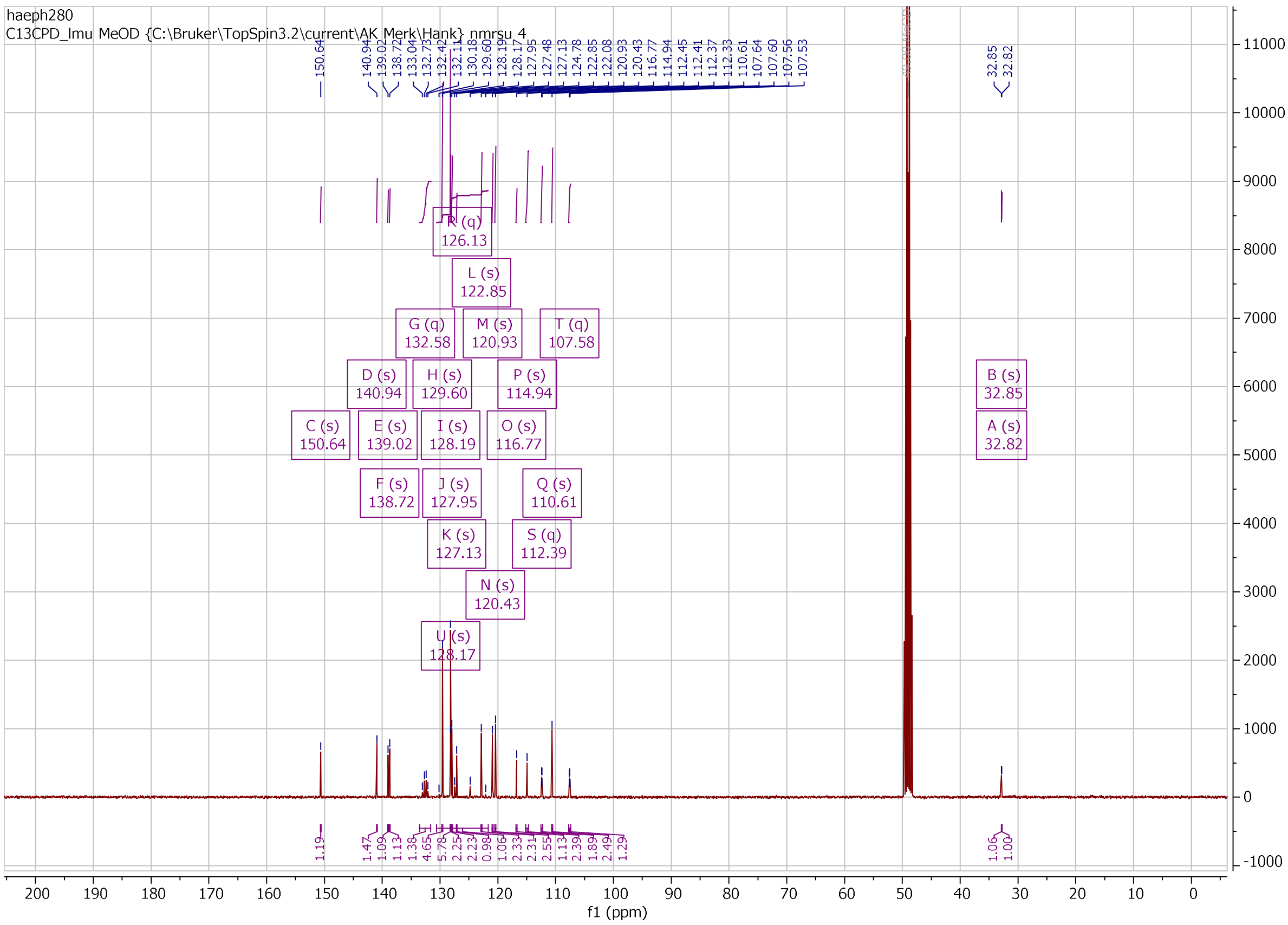

haeph280
C13CPD_lmu MeOD {C:\Bruker\TopSpin3.2\current\AK Merk\Hank} nmrsu 4
f1 (ppm)

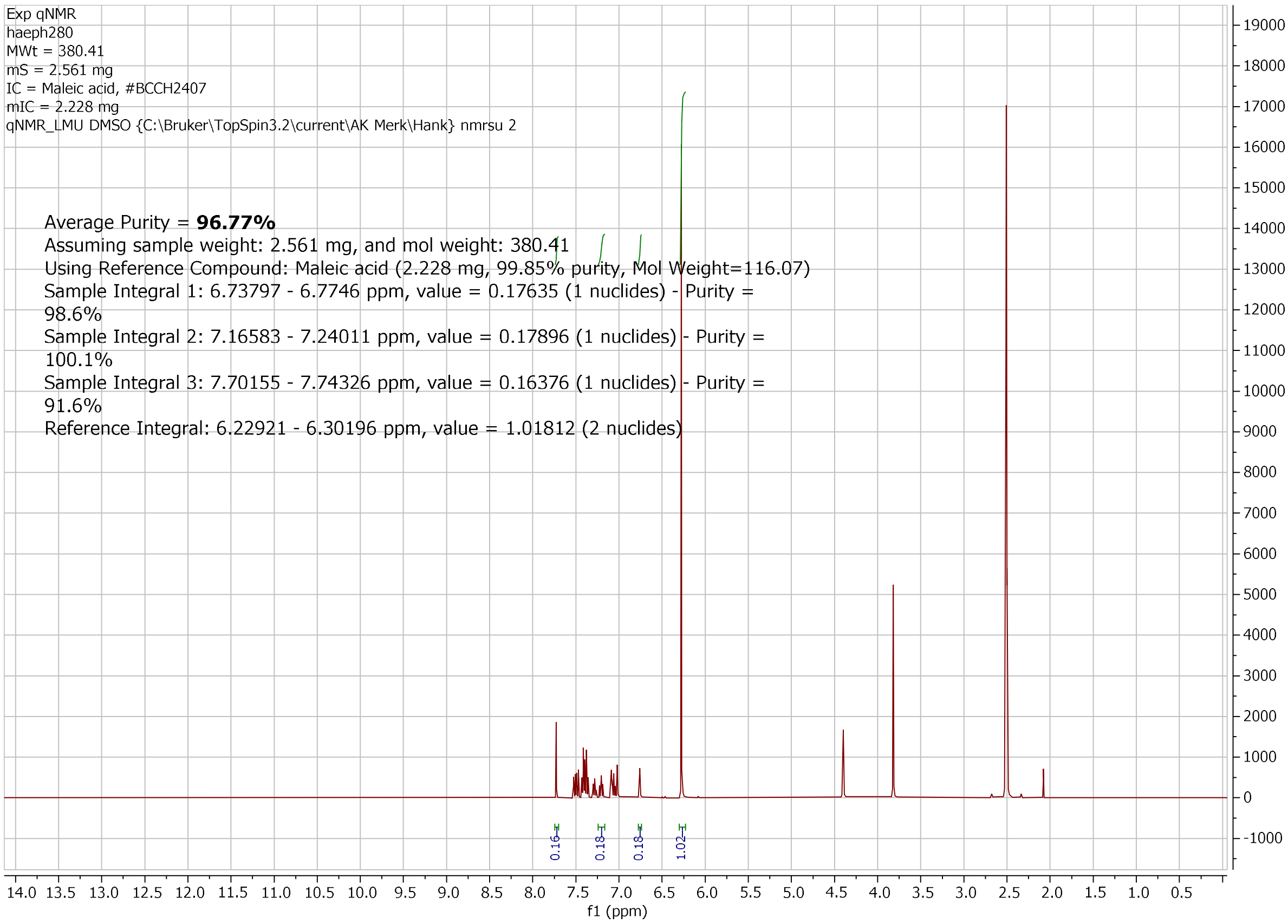

Exp qNMR
haeph280
MWt = 380.41
mS = 2.561 mg
IC = Maleic acid, #BCCH2407
mIC = 2.228 mg
qNMR_LMU DMSO {C:\Bruker\TopSpin3.2\current\AK Merk\Hank} nmrsu 2
Average Purity = 96.77%
Assuming sample weight: 2.561 mg, and mol weight: 380.41
Using Reference Compound: Maleic acid (2.228 mg, 99.85% purity, Mol Weight=116.07)
Sample Integral 1: 6.73797 - 6.7746 ppm, value = 0.17635 (1 nuclides) - Purity = 98.6%
Sample Integral 2: 7.16583 - 7.24011 ppm, value = 0.17896 (1 nuclides) - Purity = 100.1%
Sample Integral 3: 7.70155 - 7.74326 ppm, value = 0.16376 (1 nuclides) - Purity = 91.6%
Reference Integral: 6.22921 - 6.30196 ppm, value = 1.01812 (2 nuclides)
f1 (ppm)

Compound **14**:

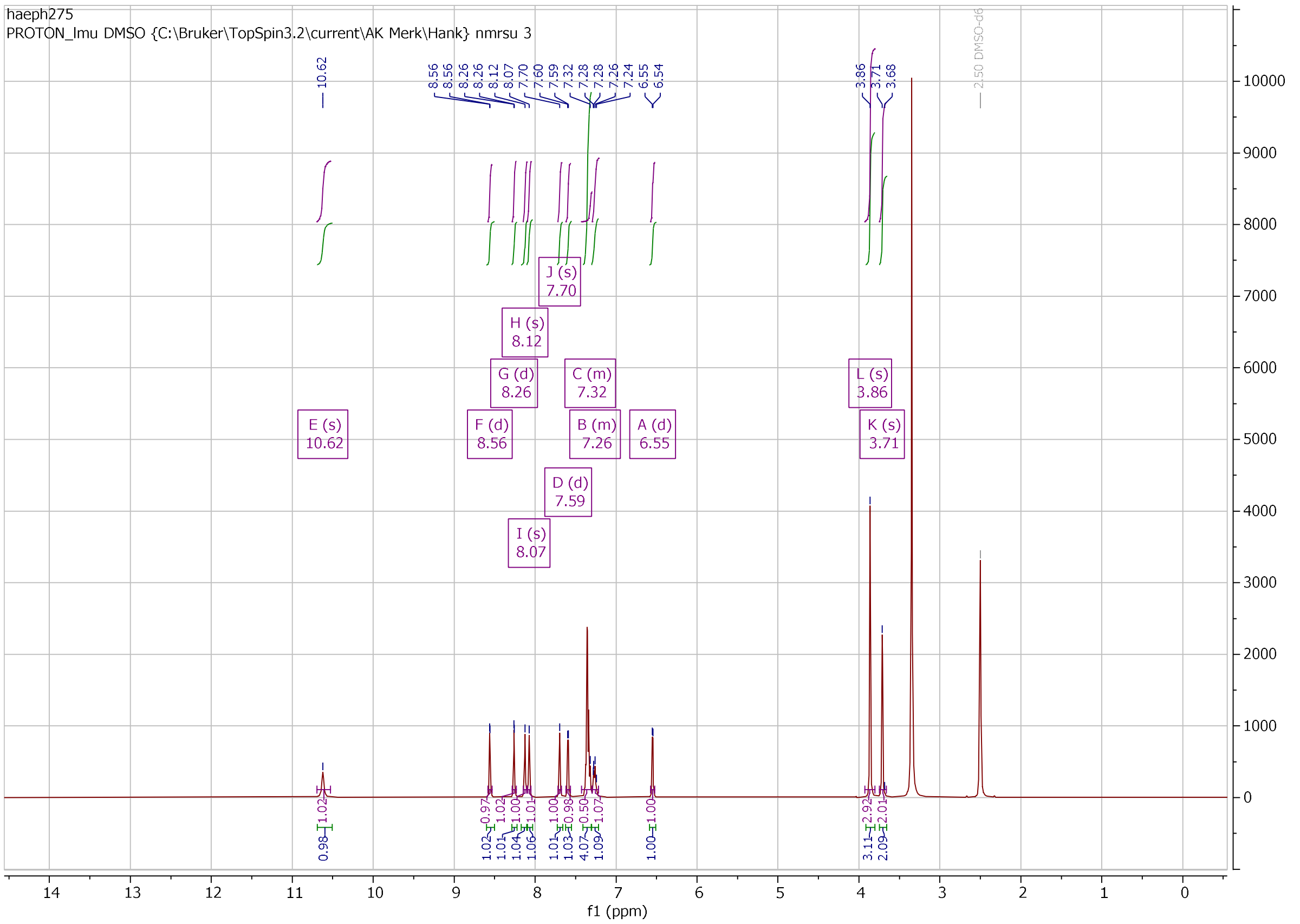

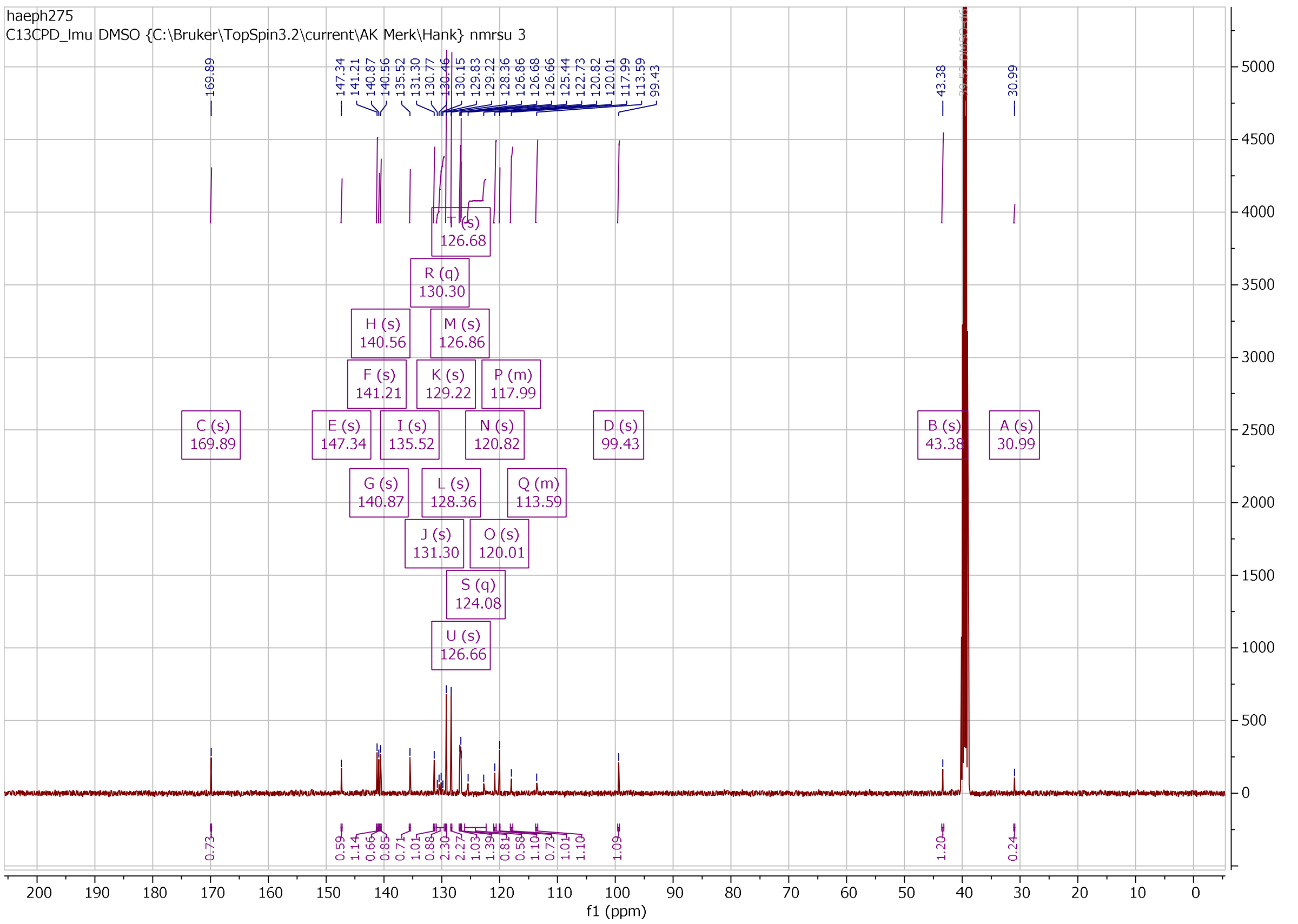

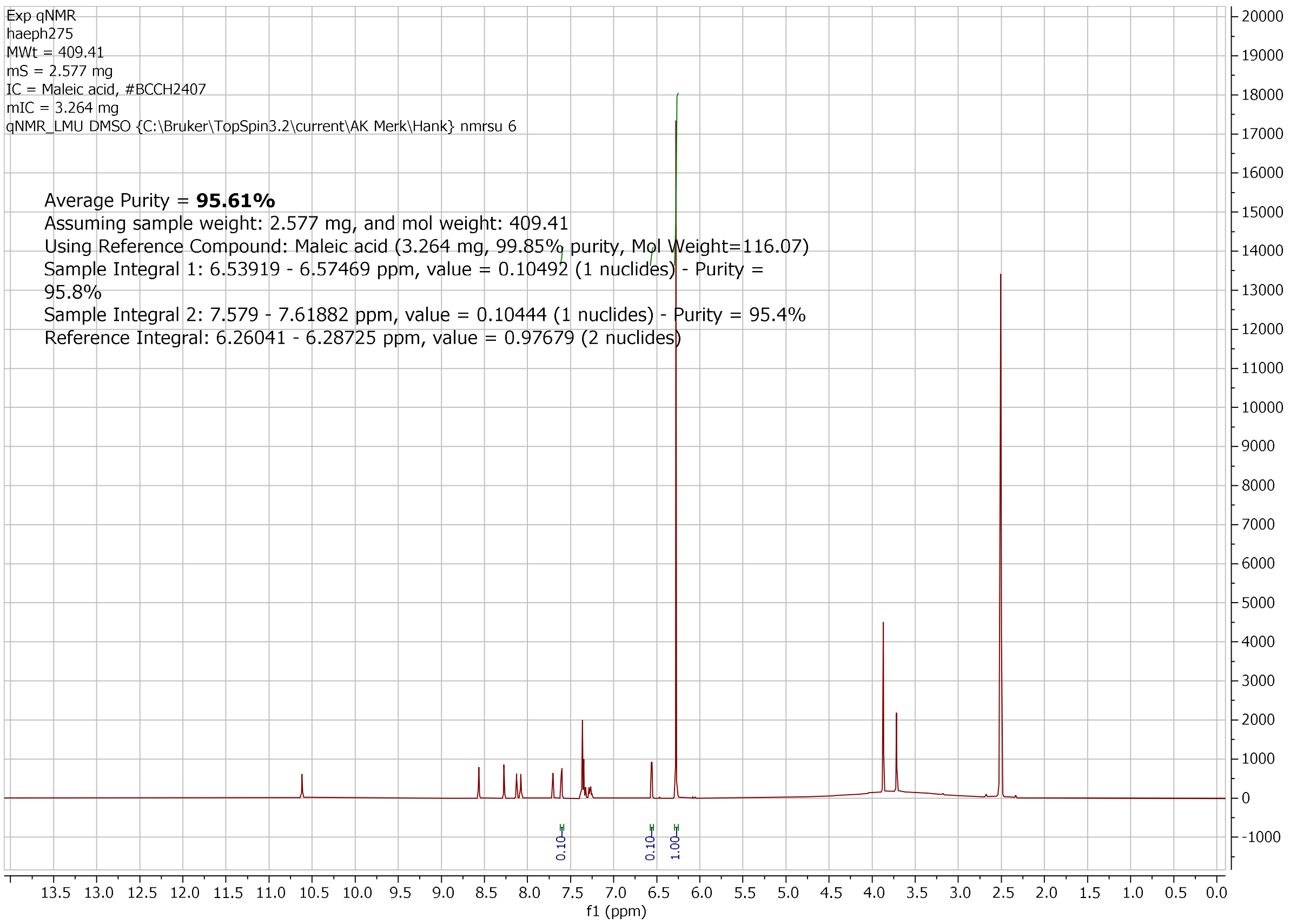
Exp qNMR
haeph275
MWt = 409.41
mS = 2.577 mg
IC = Maleic acid, #BCCH2407
mIC = 3.264 mg
qNMR_LMU DMSO {C:\Bruker\TopSpin3.2\current\AK Merk\Hank} nmrsu 6
Average Purity = **95.61%**
Assuming sample weight: 2.577 mg, and mol weight: 409.41
Using Reference Compound: Maleic acid (3.264 mg, 99.85% purity, Mol Weight=116.07)
Sample Integral 1: 6.53919 - 6.57469 ppm, value = 0.10492 (1 nuclides) - Purity = 95.8%
Sample Integral 2: 7.579 - 7.61882 ppm, value = 0.10444 (1 nuclides) - Purity = 95.4%
Reference Integral: 6.26041 - 6.28725 ppm, value = 0.97679 (2 nuclides)
f1 (ppm)

Compound **15**:

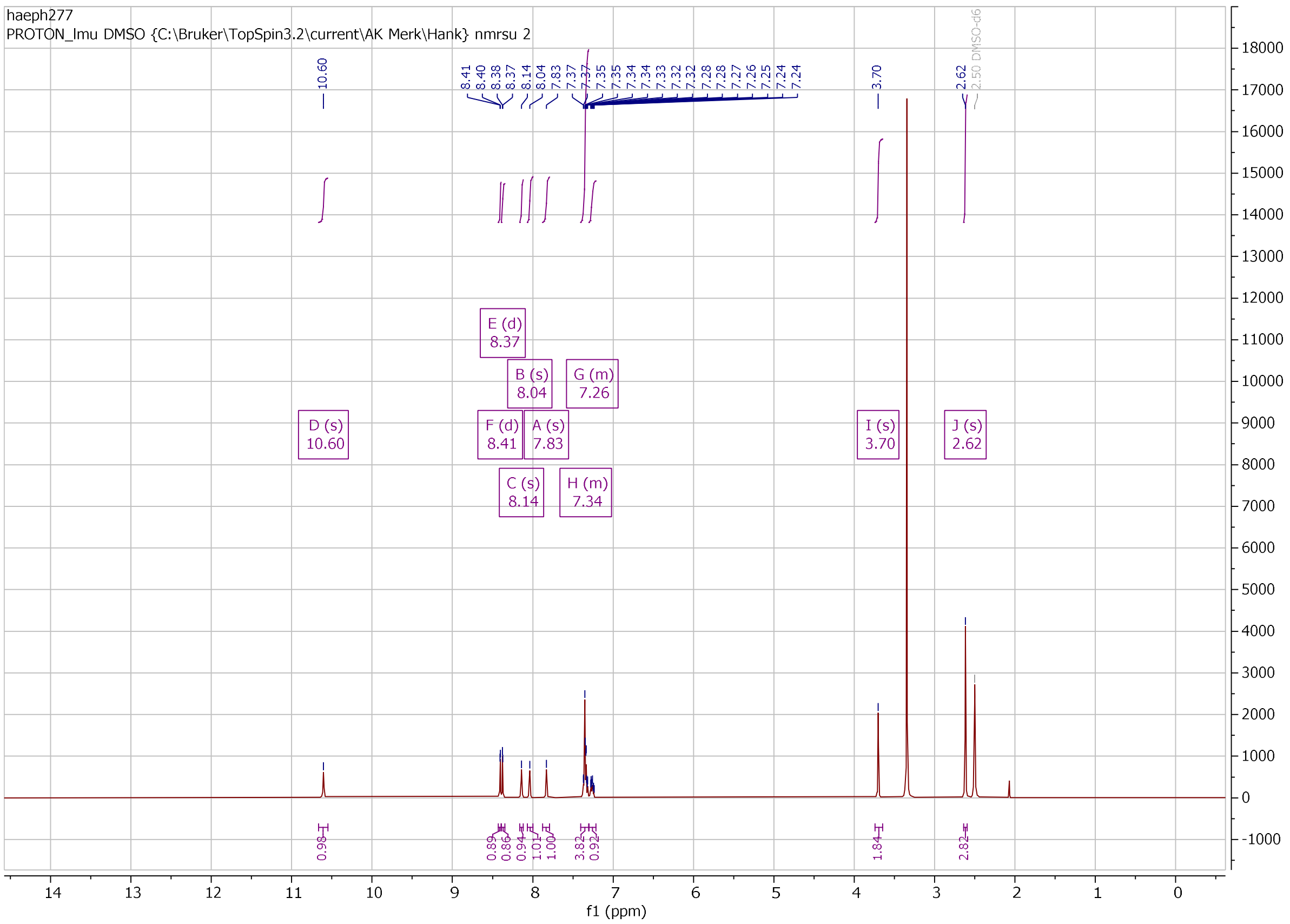
haeph277
PROTON_lmu DMSO {C:\Bruker\TopSpin3.2\current\AK Merk\Hank} nmrsu 2
E (d)
8.37
B (s)
8.04
G (m)
7.26
D (s)
10.60
F (d)
8.41
A (s)
7.83
I (s)
3.70
J (s)
2.62
C (s)
8.14
H (m)
7.34
2.50 DMSO-d6
f1 (ppm)

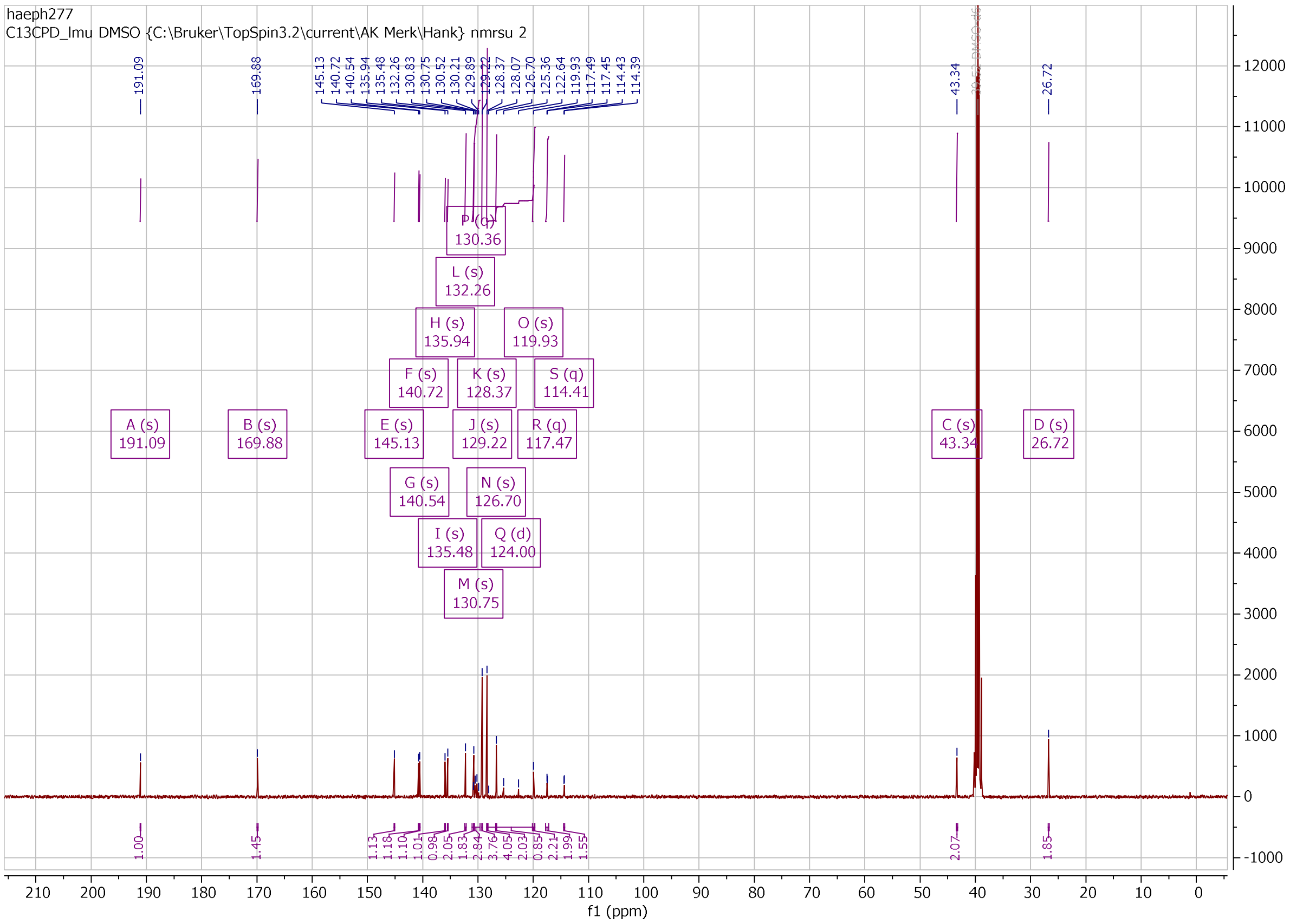
haeph277
C13CPD_lmu DMSO {C:\Bruker\TopSpin3.2\current\AK Merk\Hank} nmrsu 2
A (s) 191.09
B (s) 169.88
C (s) 43.34
D (s) 26.72
E (s) 145.13
F (s) 140.72
G (s) 140.54
H (s) 135.94
I (s) 135.48
J (s) 129.22
K (s) 128.37
L (s) 132.26
M (s) 130.75
N (s) 126.70
O (s) 119.93
P (d) 130.36
Q (d) 124.00
R (q) 117.47
S (q) 114.41
f1 (ppm)

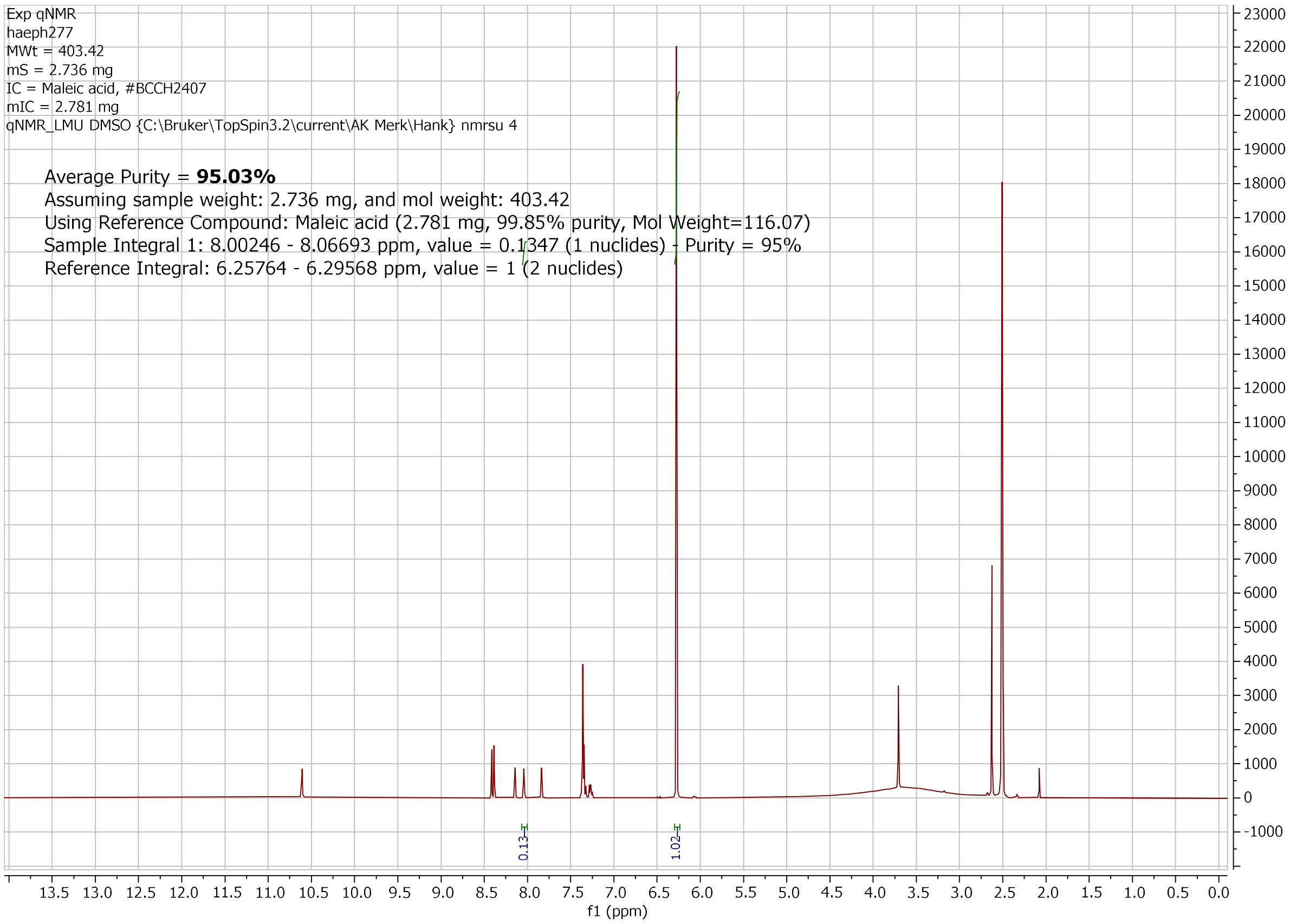
Exp qNMR
haeph277
MWt = 403.42
mS = 2.736 mg
IC = Maleic acid, #BCCH2407
mIC = 2.781 mg
qNMR_LMU DMSO {C:\Bruker\TopSpin3.2\current\AK Merk\Hank} nmrsu 4
Average Purity = 95.03%
Assuming sample weight: 2.736 mg, and mol weight: 403.42
Using Reference Compound: Maleic acid (2.781 mg, 99.85% purity, Mol Weight=116.07)
Sample Integral 1: 8.00246 - 8.06693 ppm, value = 0.1347 (1 nuclides) - Purity = 95%
Reference Integral: 6.25764 - 6.29568 ppm, value = 1 (2 nuclides)
f1 (ppm)

Compound **16**:

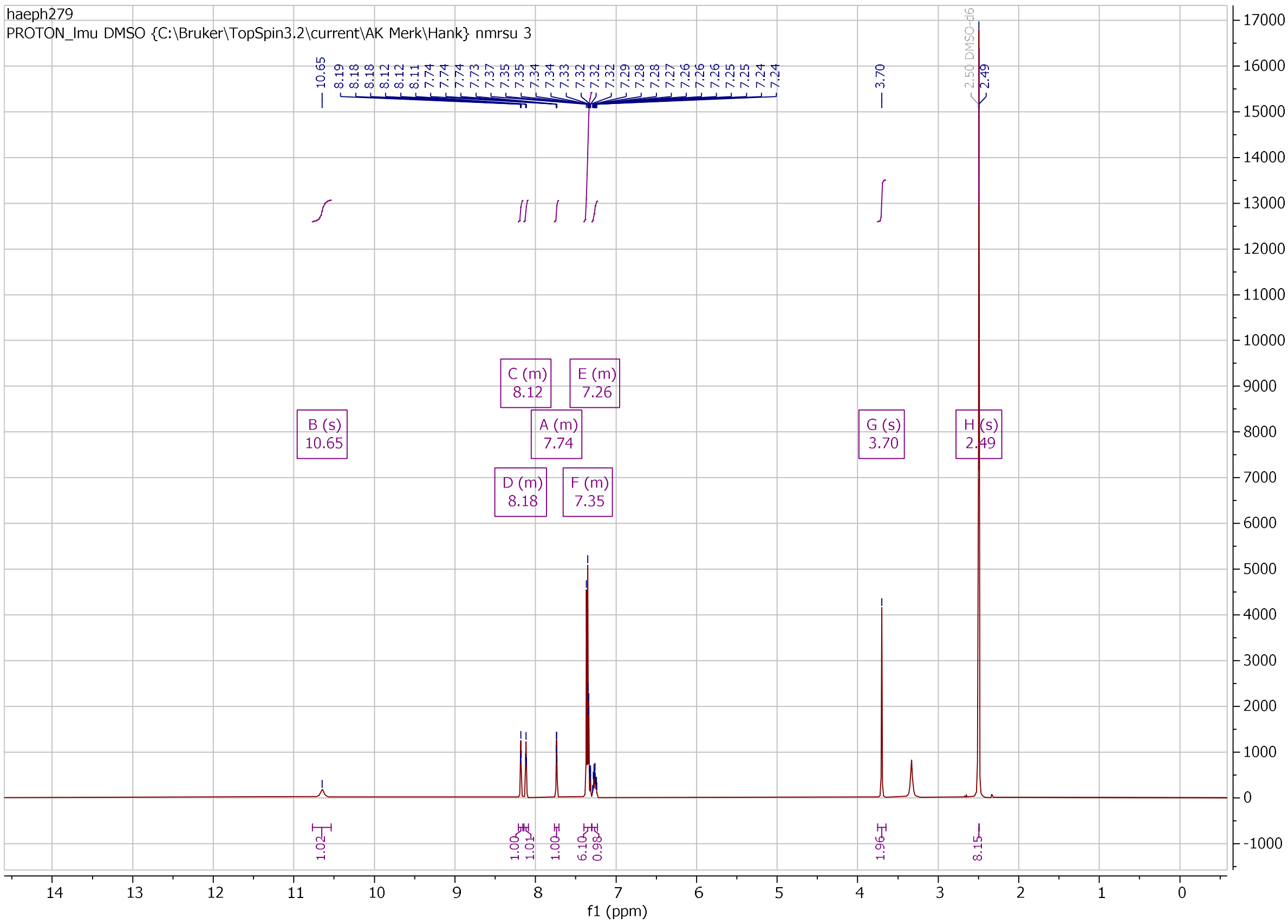
haeph279
PROTON_Imu DMSO {C:\Bruker\TopSpin3.2\current\AK Merk\Hank} nmrsu 3
B (s) 10.65
C (m) 8.12
D (m) 8.18
A (m) 7.74
E (m) 7.26
F (m) 7.35
G (s) 3.70
H (s) 2.49
f1 (ppm)

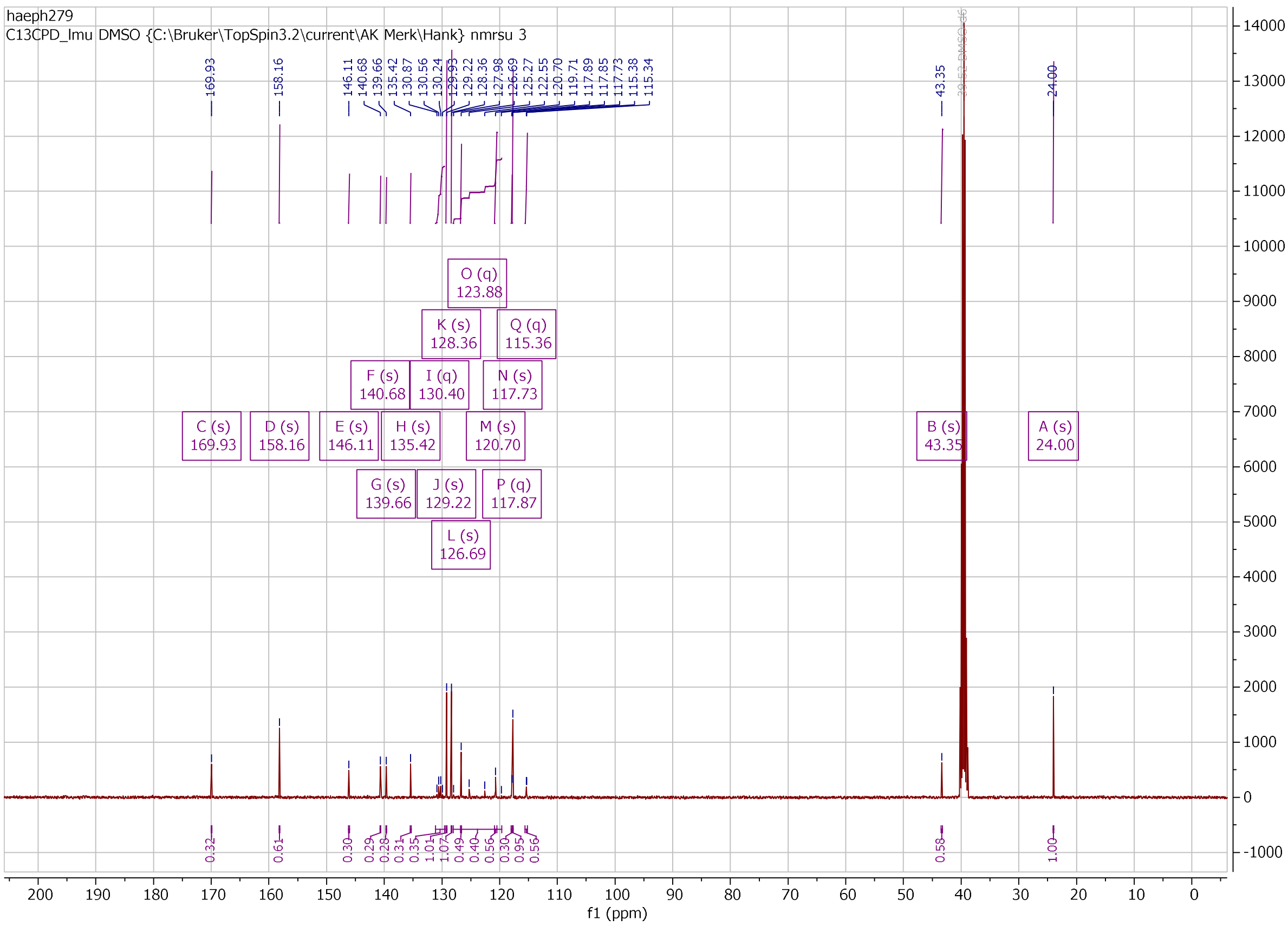
haeph279
C13CPD_Imu DMSO {C:\Bruker\TopSpin3.2\current\AK Merk\Hank} nmrsu 3
O (q) 123.88
K (s) 128.36
Q (q) 115.36
F (s) 140.68
I (q) 130.40
N (s) 117.73
C (s) 169.93
D (s) 158.16
E (s) 146.11
H (s) 135.42
M (s) 120.70
B (s) 43.35
A (s) 24.00
G (s) 139.66
J (s) 129.22
P (q) 117.87
L (s) 126.69
f1 (ppm)

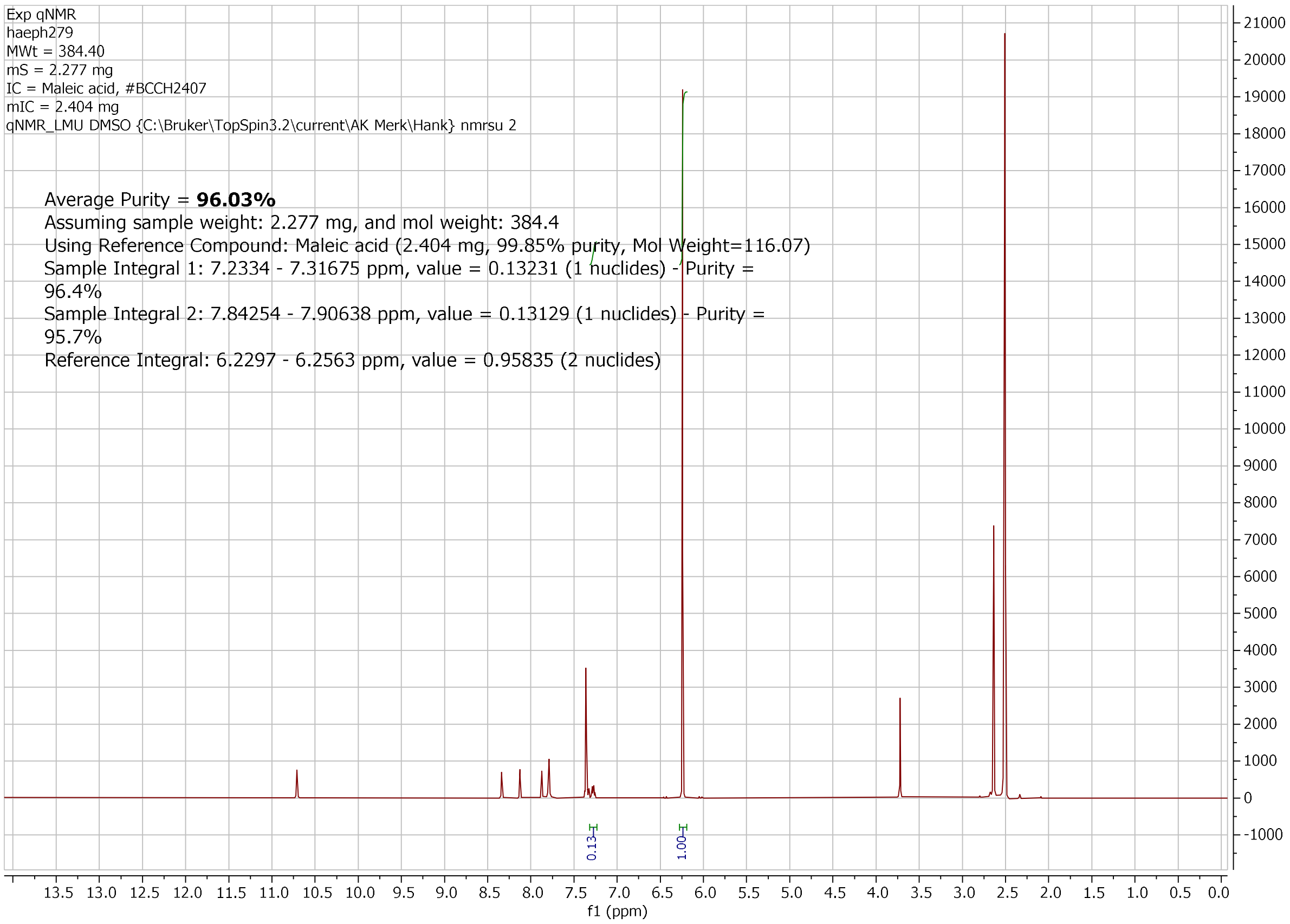
Exp qNMR
haeph279
MWt = 384.40
mS = 2.277 mg
IC = Maleic acid, #BCCH2407
mIC = 2.404 mg
qNMR_LMU DMSO {C:\Bruker\TopSpin3.2\current\AK Merk\Hank} nmrsu 2
Average Purity = **96.03%**
Assuming sample weight: 2.277 mg, and mol weight: 384.4
Using Reference Compound: Maleic acid (2.404 mg, 99.85% purity, Mol Weight=116.07)
Sample Integral 1: 7.2334 - 7.31675 ppm, value = 0.13231 (1 nuclides) - Purity = 96.4%
Sample Integral 2: 7.84254 - 7.90638 ppm, value = 0.13129 (1 nuclides) - Purity = 95.7%
Reference Integral: 6.2297 - 6.2563 ppm, value = 0.95835 (2 nuclides)
0.13
1.00
f1 (ppm)

Compound **17**:

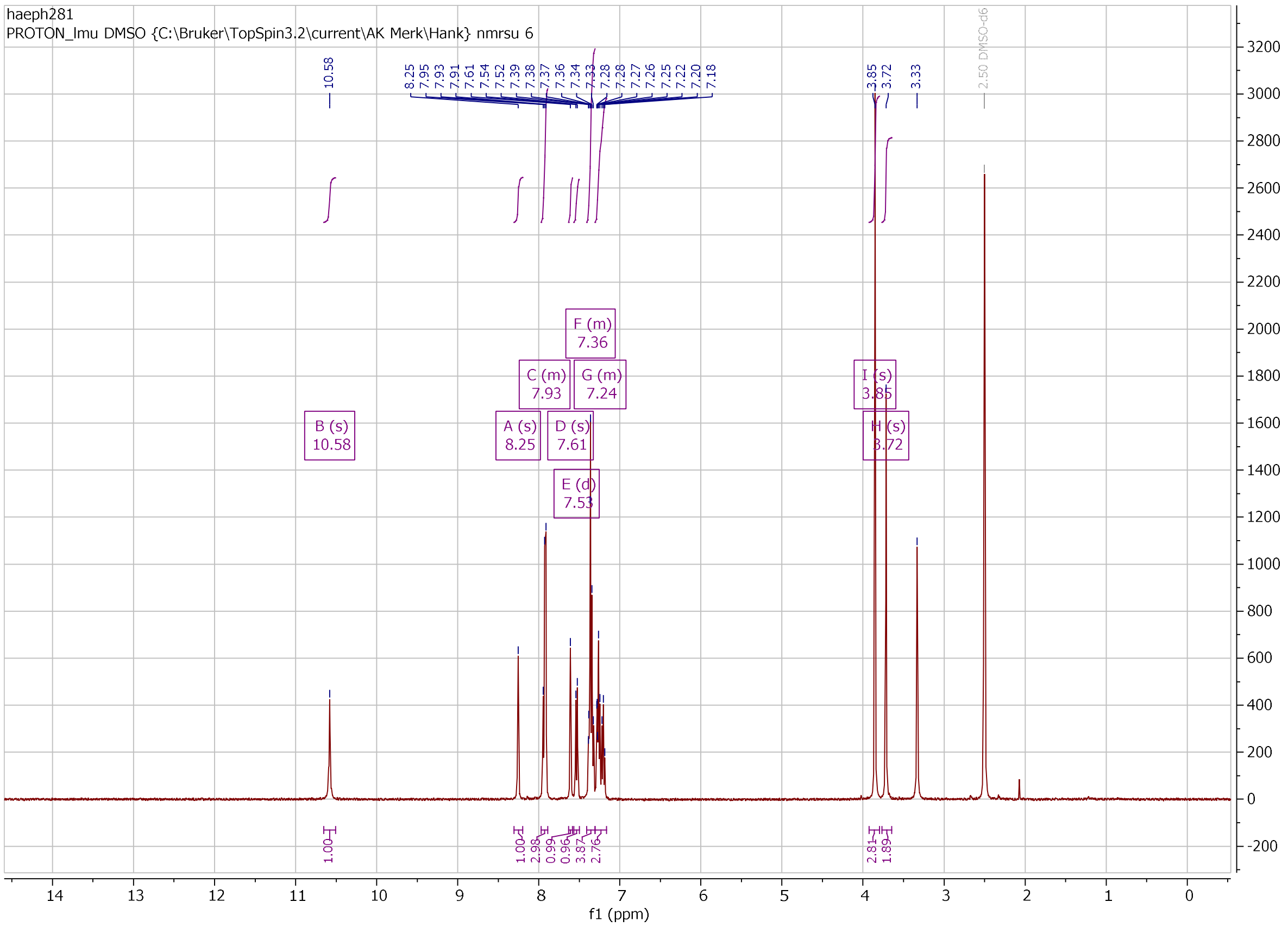
haeph281
PROTON_lmu DMSO {C:\Bruker\TopSpin3.2\current\AK Merk\Hank} nmrsu 6
10.58
8.25
7.95
7.93
7.91
7.61
7.54
7.52
7.39
7.38
7.37
7.36
7.34
7.33
7.28
7.28
7.27
7.26
7.25
7.22
7.20
7.18
3.85
3.72
3.33
2.50 DMSO-d6
F (m)
7.36
C (m)
7.93
G (m)
7.24
I (s)
3.85
B (s)
10.58
A (s)
8.25
D (s)
7.61
H (s)
3.72
E (d)
7.53
1.00
1.00
2.98
0.99
0.96
3.87
2.76
2.81
1.89
f1 (ppm)

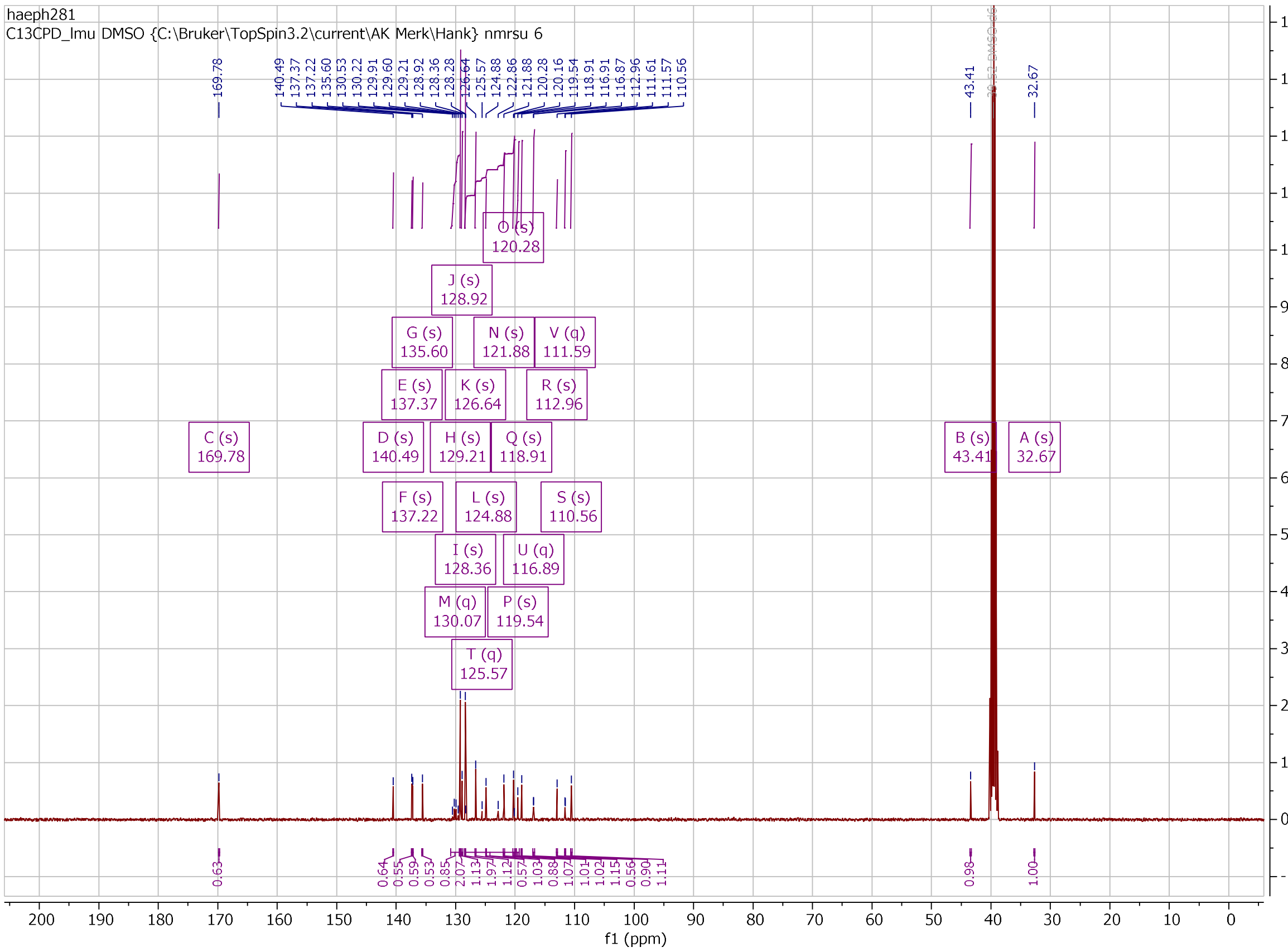
haeph281
C13CPD_lmu DMSO {C:\Bruker\TopSpin3.2\current\AK Merk\Hank} nmrsu 6
f1 (ppm)

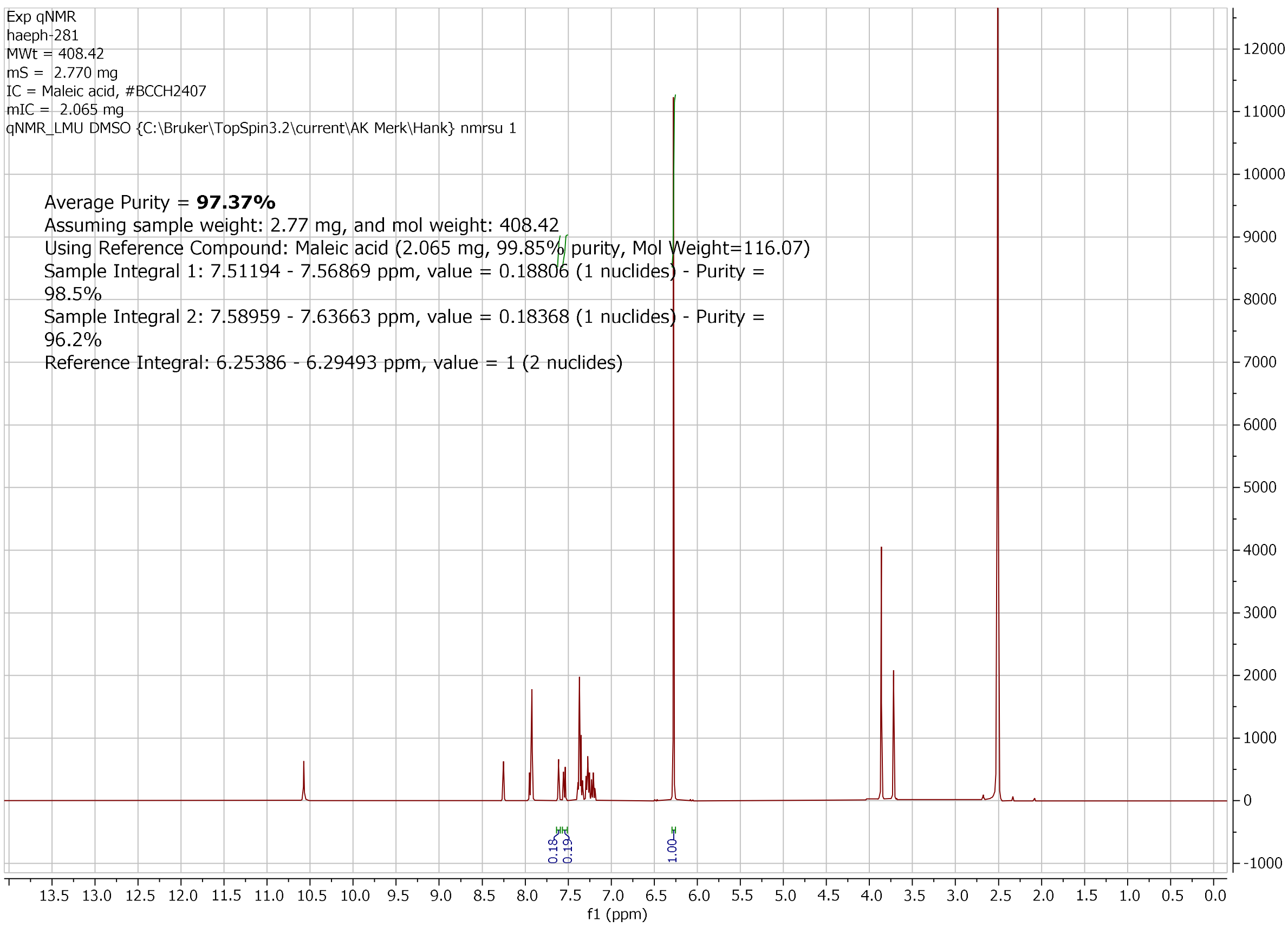
Exp qNMR
haeph-281
MWt = 408.42
mS = 2.770 mg
IC = Maleic acid, #BCCH2407
mIC = 2.065 mg
qNMR_LMU DMSO {C:\Bruker\TopSpin3.2\current\AK Merk\Hank} nmrsu 1
Average Purity = 97.37%
Assuming sample weight: 2.77 mg, and mol weight: 408.42
Using Reference Compound: Maleic acid (2.065 mg, 99.85% purity, Mol Weight=116.07)
Sample Integral 1: 7.51194 - 7.56869 ppm, value = 0.18806 (1 nuclides) - Purity = 98.5%
Sample Integral 2: 7.58959 - 7.63663 ppm, value = 0.18368 (1 nuclides) - Purity = 96.2%
Reference Integral: 6.25386 - 6.29493 ppm, value = 1 (2 nuclides)
f1 (ppm)

Compound **18**:

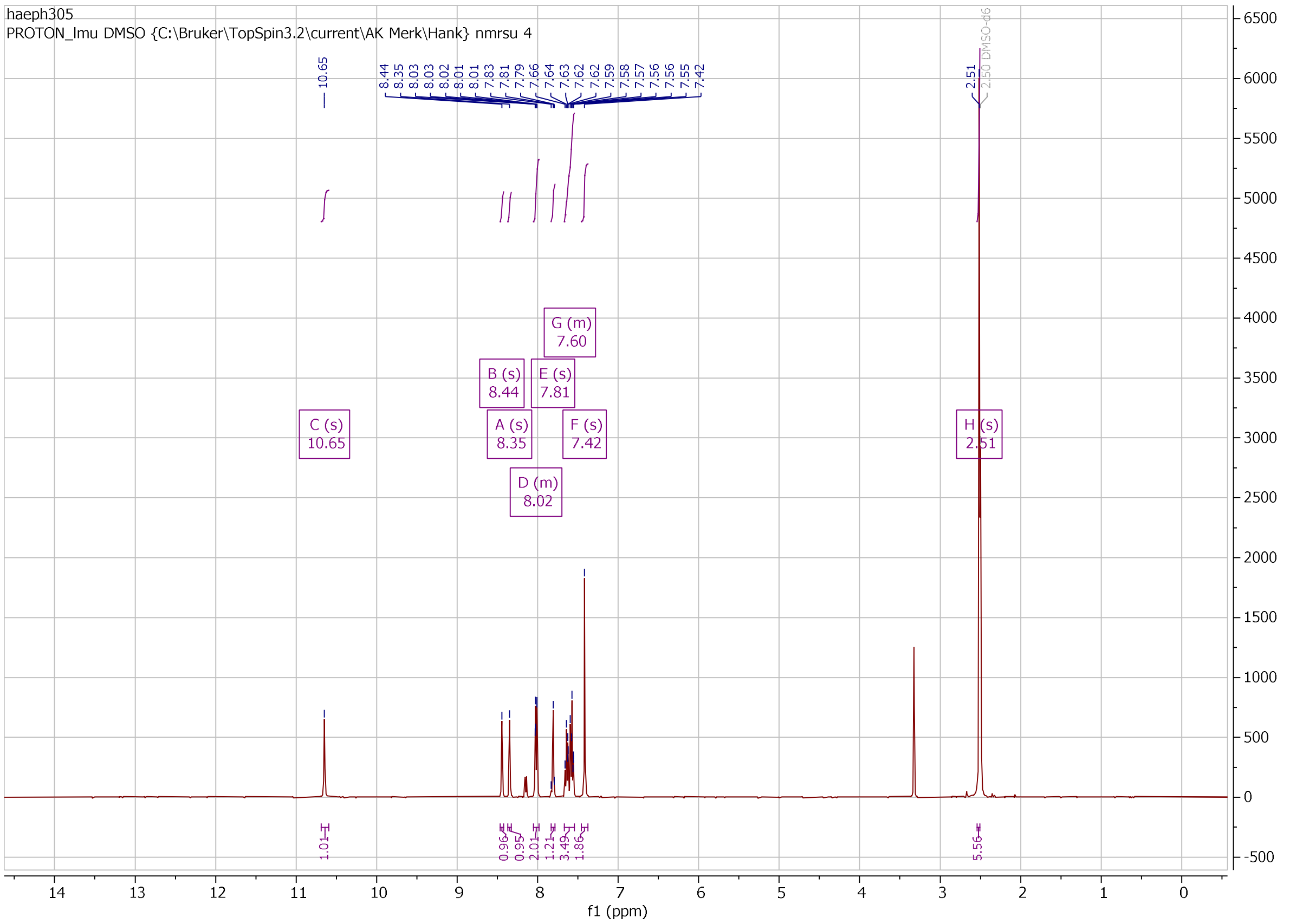

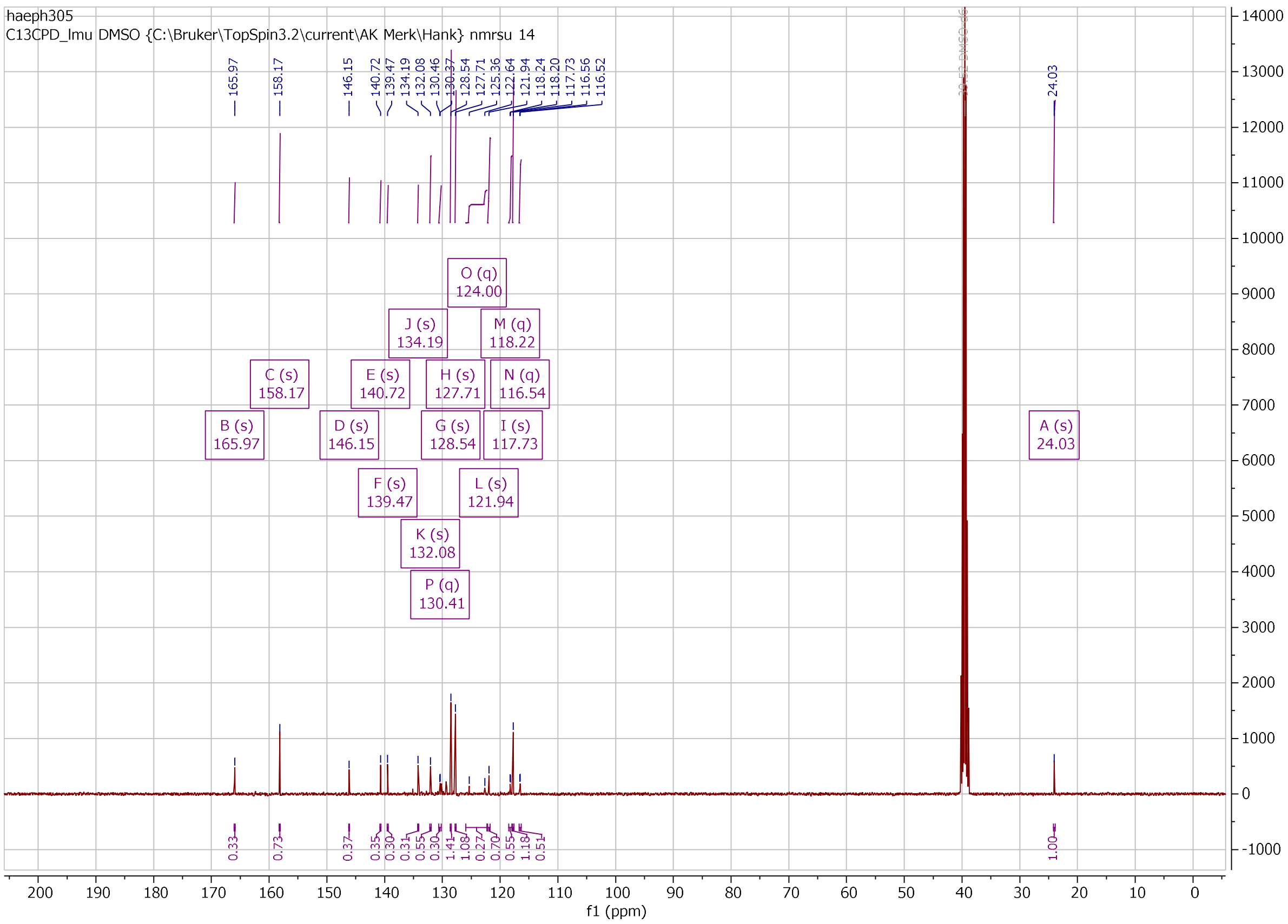

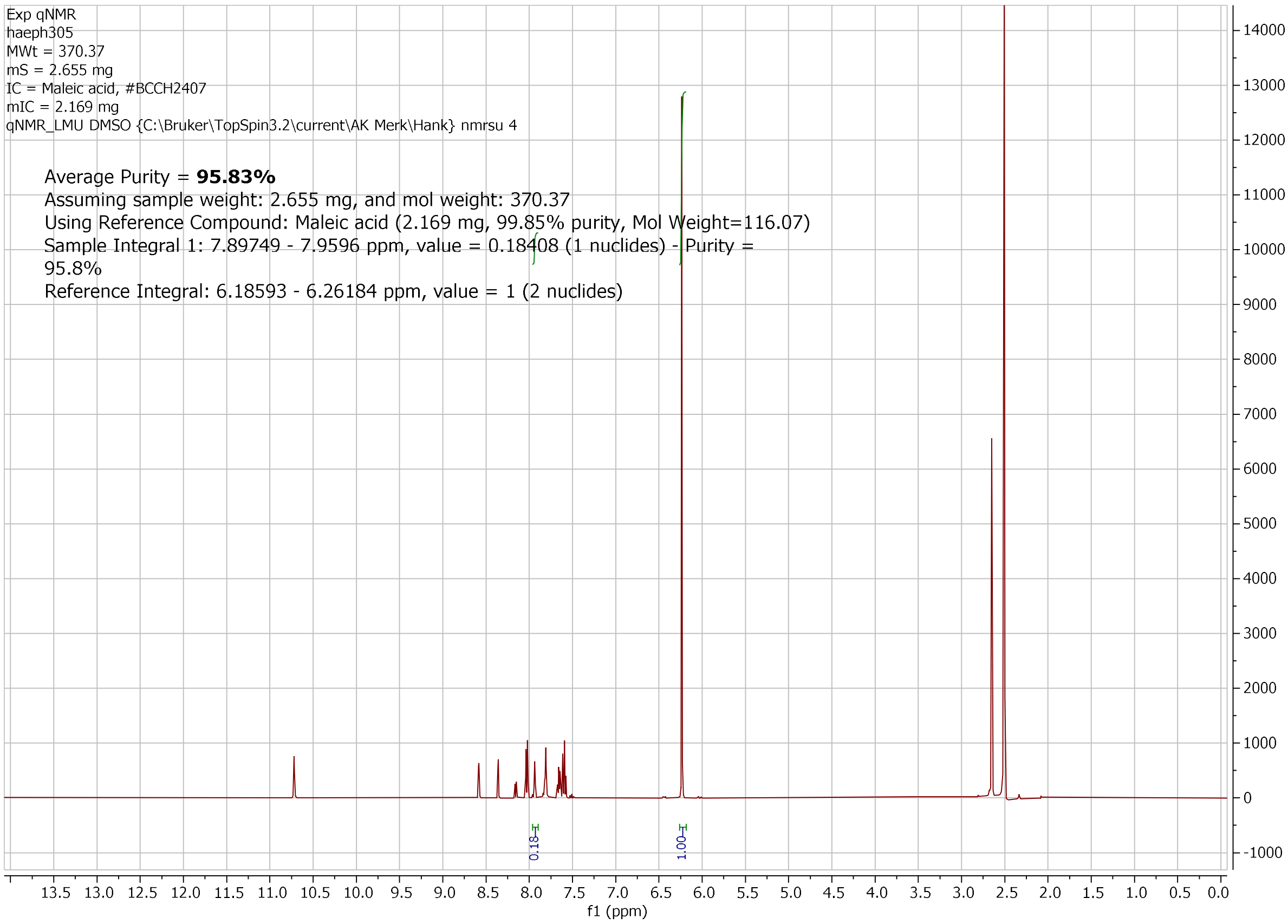
Exp qNMR
haeph305
MWt = 370.37
mS = 2.655 mg
IC = Maleic acid, #BCCH2407
mIC = 2.169 mg
qNMR_LMU DMSO {C:\Bruker\TopSpin3.2\current\AK Merk\Hank} nmrsu 4
Average Purity = 95.83%
Assuming sample weight: 2.655 mg, and mol weight: 370.37
Using Reference Compound: Maleic acid (2.169 mg, 99.85% purity, Mol Weight=116.07)
Sample Integral 1: 7.89749 - 7.9596 ppm, value = 0.18408 (1 nuclides) - Purity = 95.8%
Reference Integral: 6.18593 - 6.26184 ppm, value = 1 (2 nuclides)
f1 (ppm)

Compound **19**:

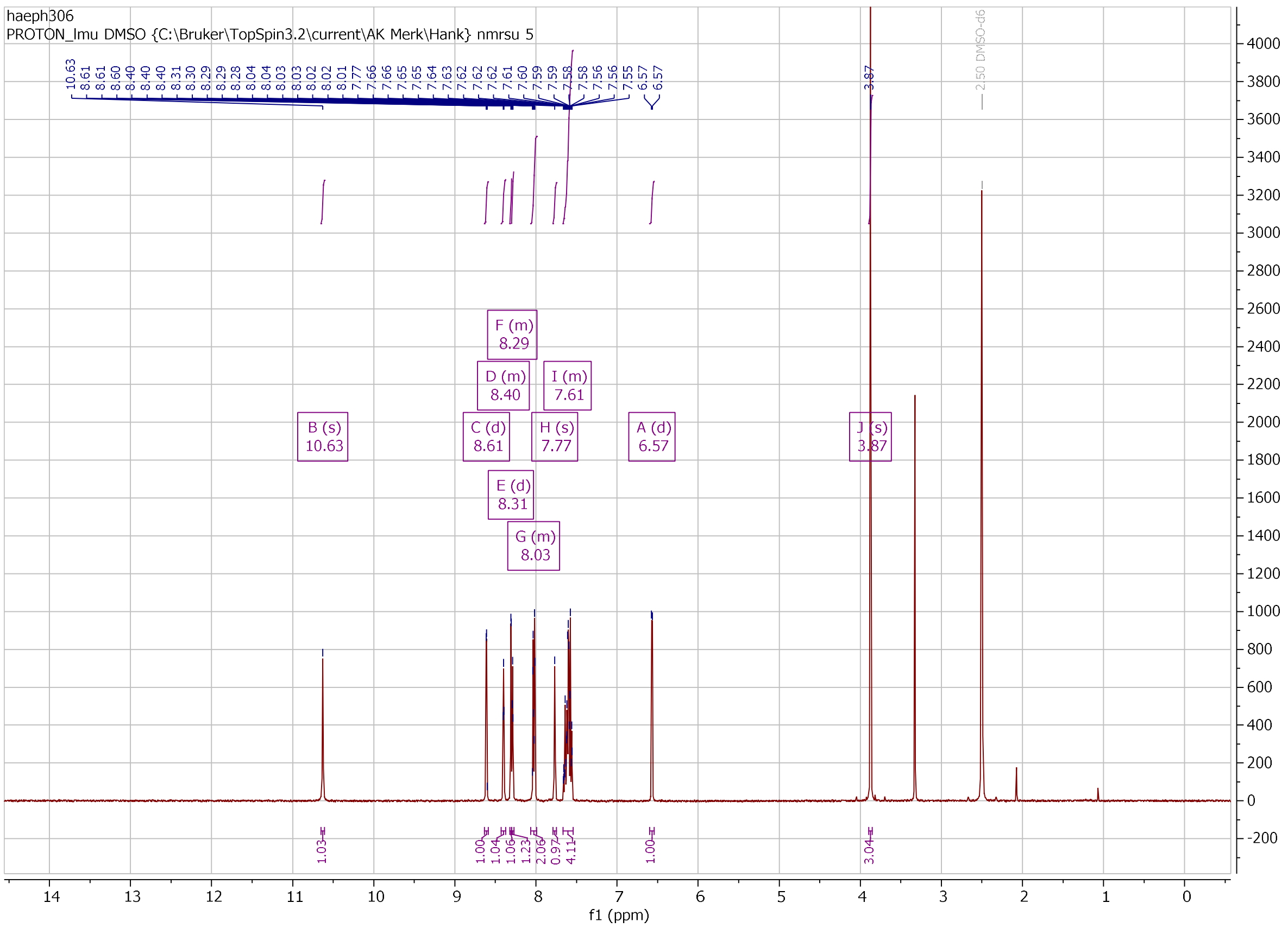
haeph306
PROTON_lmu DMSO {C:\Bruker\TopSpin3.2\current\AK Merk\Hank} nmrsu 5
B (s) 10.63
C (d) 8.61
D (m) 8.40
E (d) 8.31
F (m) 8.29
G (m) 8.03
H (s) 7.77
I (m) 7.61
A (d) 6.57
J (s) 3.87
f1 (ppm)

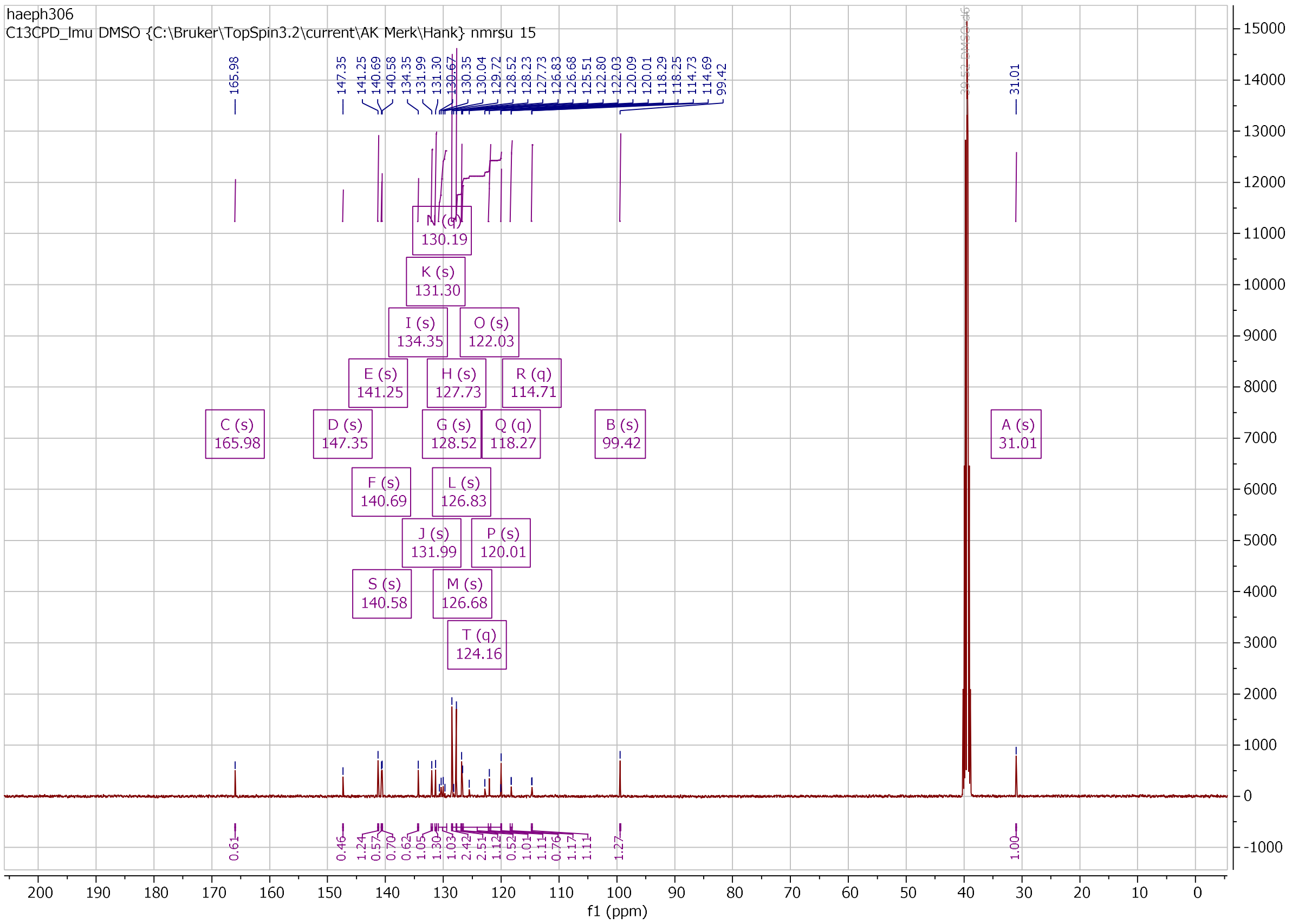
haeph306
C13CPD_lmu DMSO {C:\Bruker\TopSpin3.2\current\AK Merk\Hank} nmrsu 15
f1 (ppm)

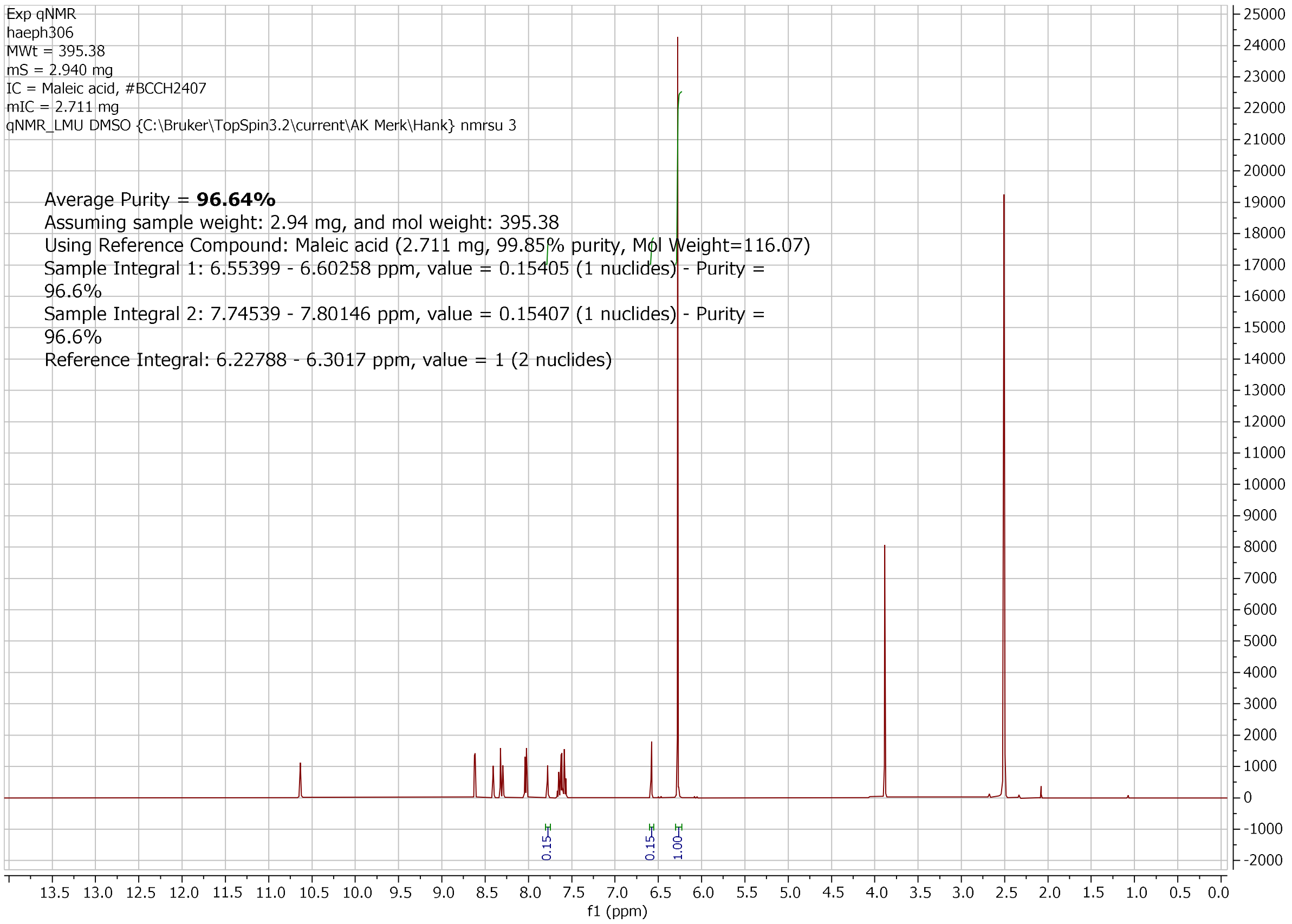
Exp qNMR
haeph306
MWt = 395.38
mS = 2.940 mg
IC = Maleic acid, #BCCH2407
mIC = 2.711 mg
qNMR_LMU DMSO {C:\Bruker\TopSpin3.2\current\AK Merk\Hank} nmrsu 3
Average Purity = **96.64%**
Assuming sample weight: 2.94 mg, and mol weight: 395.38
Using Reference Compound: Maleic acid (2.711 mg, 99.85% purity, Mol Weight=116.07)
Sample Integral 1: 6.55399 - 6.60258 ppm, value = 0.15405 (1 nuclides) - Purity = 96.6%
Sample Integral 2: 7.74539 - 7.80146 ppm, value = 0.15407 (1 nuclides) - Purity = 96.6%
Reference Integral: 6.22788 - 6.3017 ppm, value = 1 (2 nuclides)
f1 (ppm)

Compound **20**:

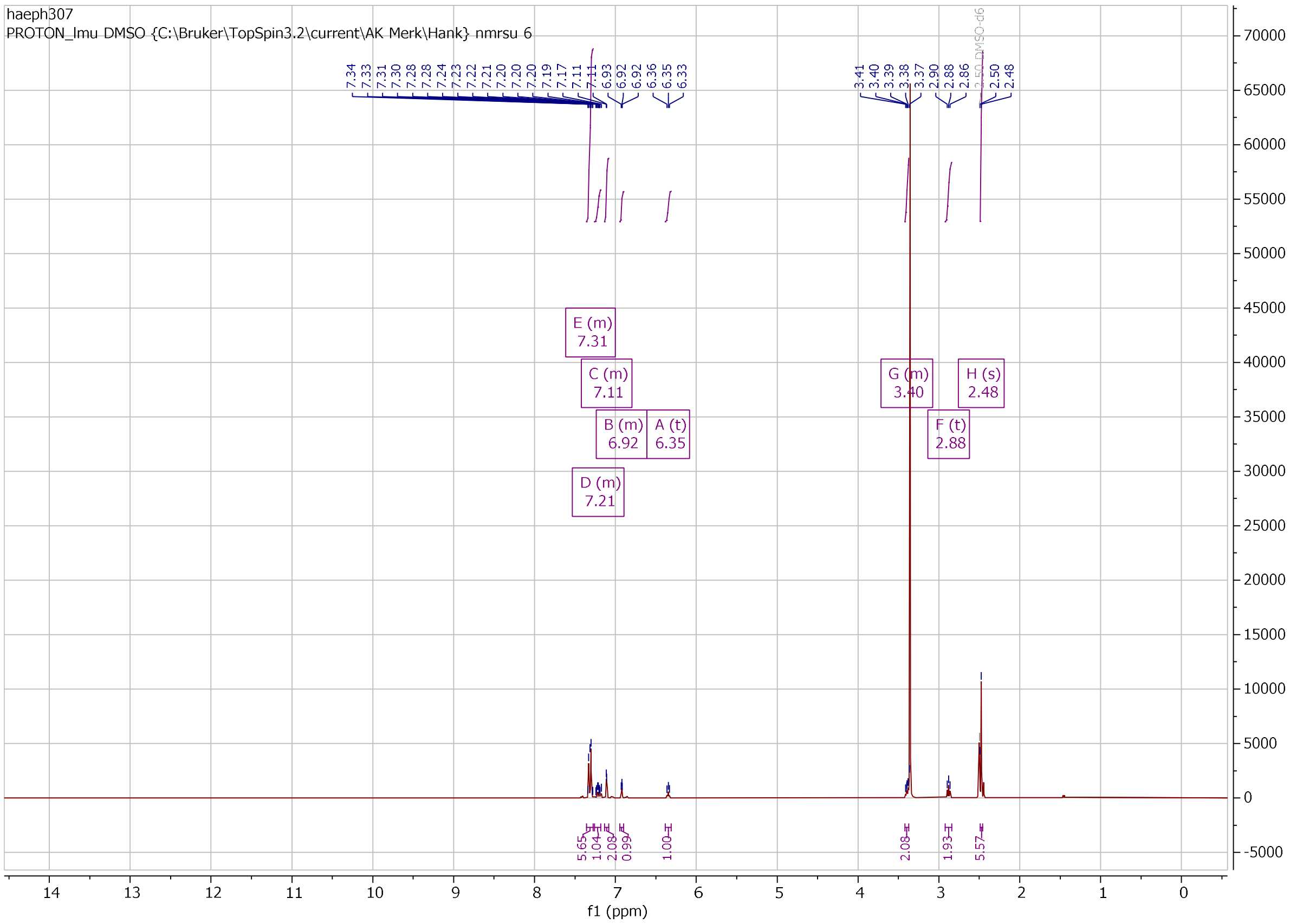
haeph307
PROTON_lmu DMSO {C:\Bruker\TopSpin3.2\current\AK Merk\Hank} nmrsu 6
E (m) 7.31
C (m) 7.11
B (m) 6.92
A (t) 6.35
D (m) 7.21
G (m) 3.40
H (s) 2.48
F (t) 2.88
f1 (ppm)

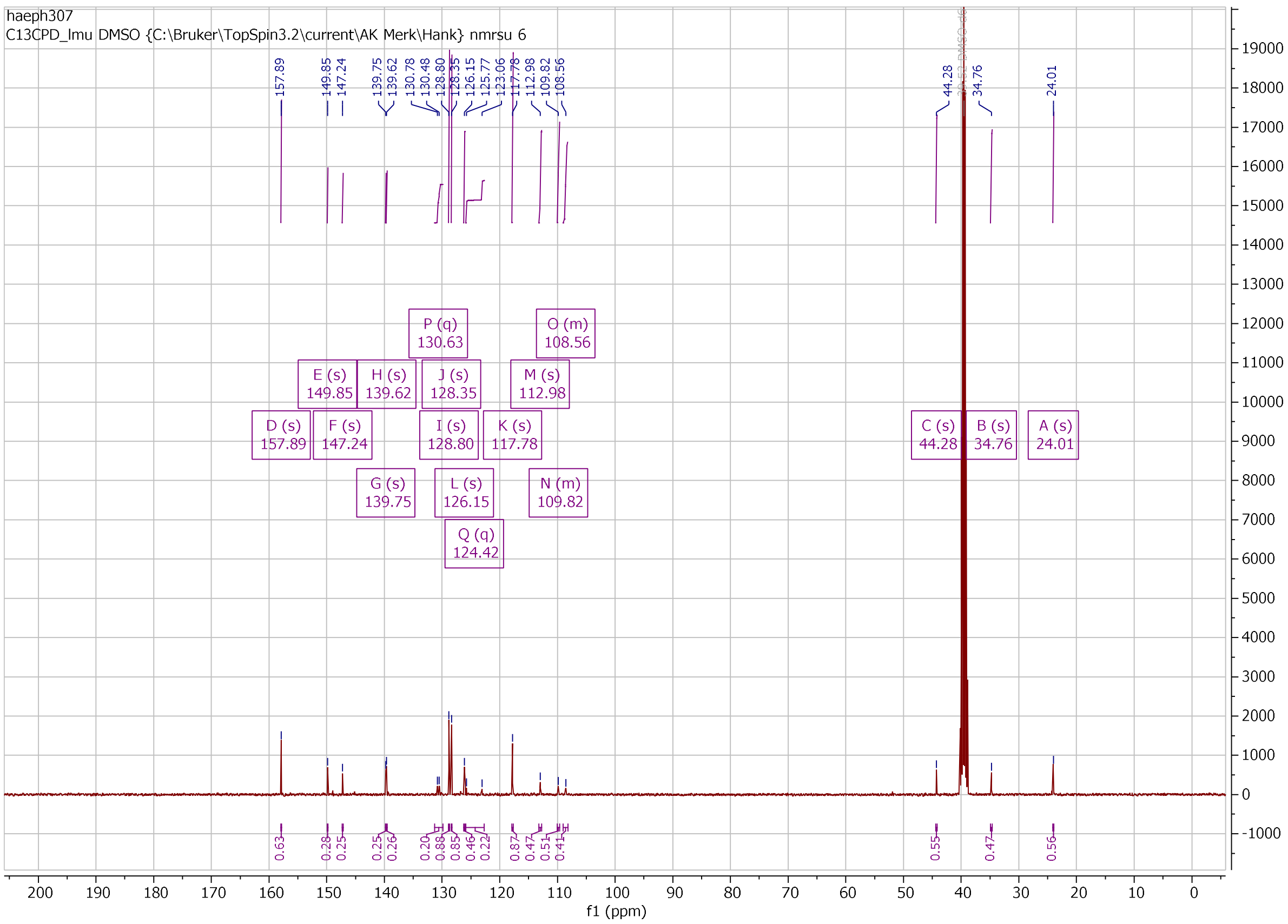
haeph307
C13CPD_lmu DMSO {C:\Bruker\TopSpin3.2\current\AK Merk\Hank} nmrsu 6
P (q) 130.63
O (m) 108.56
E (s) 149.85
H (s) 139.62
J (s) 128.35
M (s) 112.98
D (s) 157.89
F (s) 147.24
I (s) 128.80
K (s) 117.78
G (s) 139.75
L (s) 126.15
N (m) 109.82
Q (q) 124.42
C (s) 44.28
B (s) 34.76
A (s) 24.01
f1 (ppm)

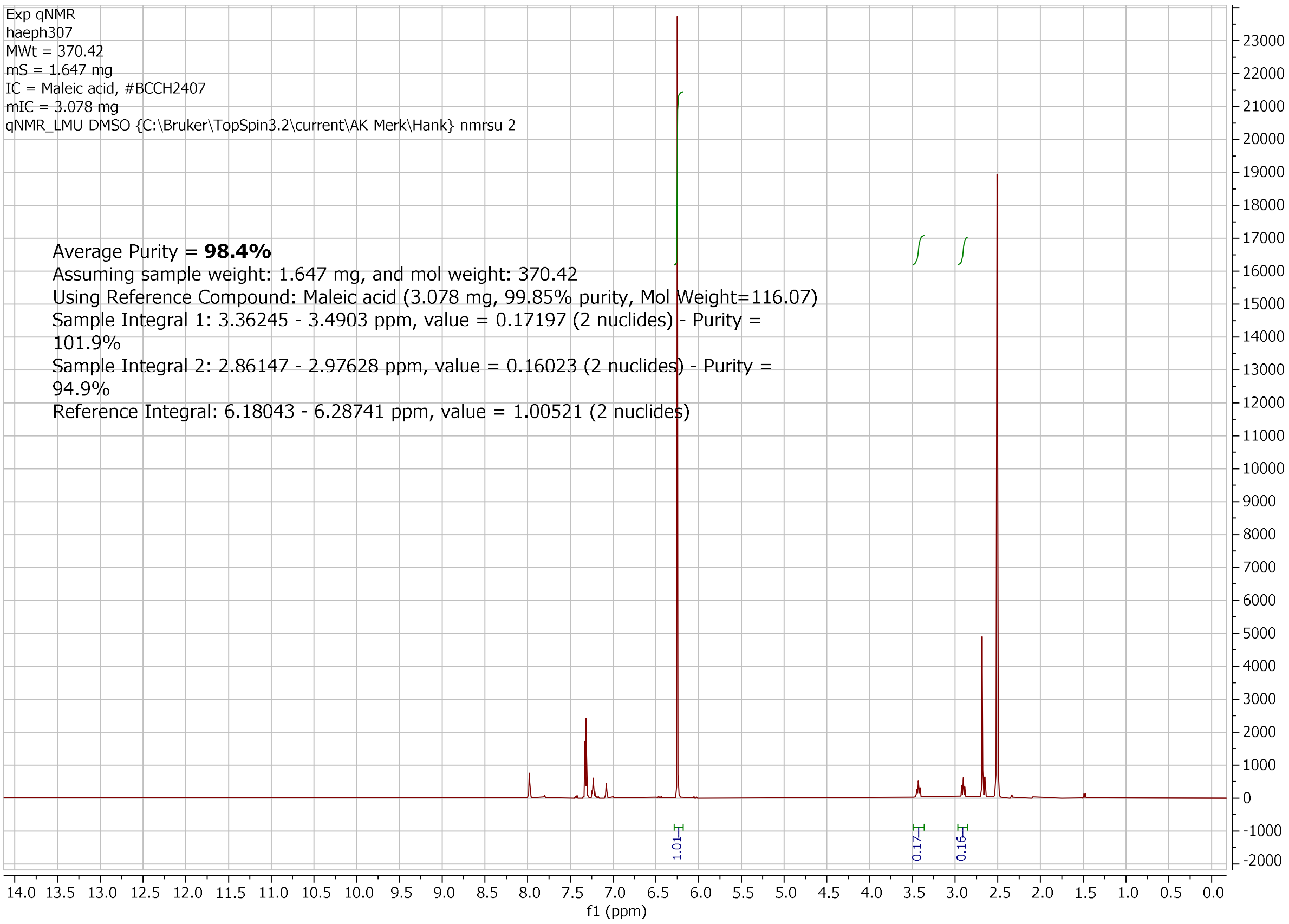

Exp qNMR
haeph307
MWt = 370.42
mS = 1.647 mg
IC = Maleic acid, #BCCH2407
mIC = 3.078 mg
qNMR_LMU DMSO {C:\Bruker\TopSpin3.2\current\AK Merk\Hank} nmrsu 2
Average Purity = 98.4%
Assuming sample weight: 1.647 mg, and mol weight: 370.42
Using Reference Compound: Maleic acid (3.078 mg, 99.85% purity, Mol Weight=116.07)
Sample Integral 1: 3.36245 - 3.4903 ppm, value = 0.17197 (2 nuclides) - Purity = 101.9%
Sample Integral 2: 2.86147 - 2.97628 ppm, value = 0.16023 (2 nuclides) - Purity = 94.9%
Reference Integral: 6.18043 - 6.28741 ppm, value = 1.00521 (2 nuclides)
f1 (ppm)

Compound **21**:

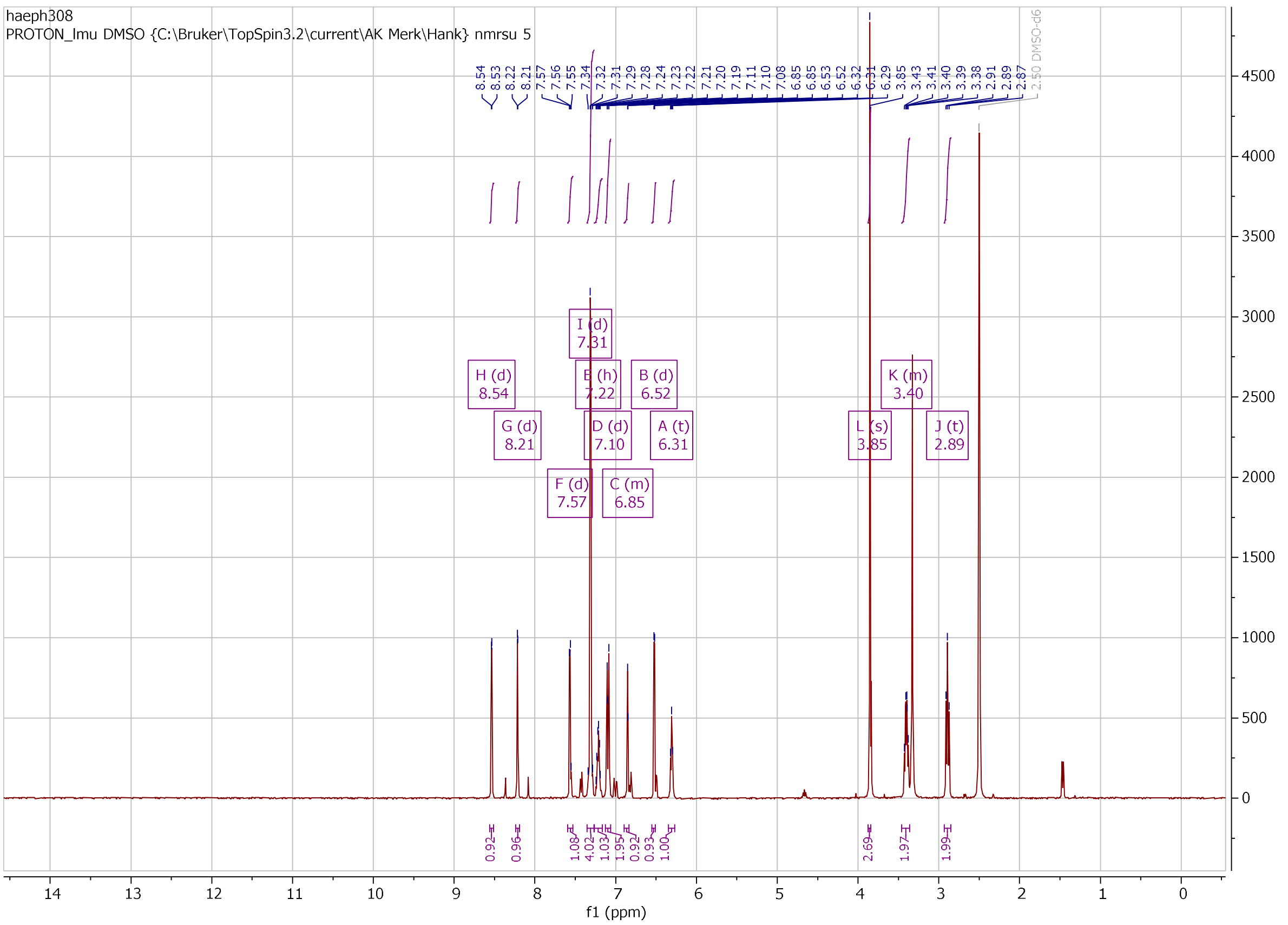

haeph308
PROTON_lmu DMSO {C:\Bruker\TopSpin3.2\current\AK Merk\Hank} nmrsu 5
H (d) 8.54
G (d) 8.21
F (d) 7.57
I (d) 7.31
E (h) 7.22
D (d) 7.10
C (m) 6.85
B (d) 6.52
A (t) 6.31
L (s) 3.85
K (m) 3.40
J (t) 2.89
2.50 DMSO-d6
f1 (ppm)

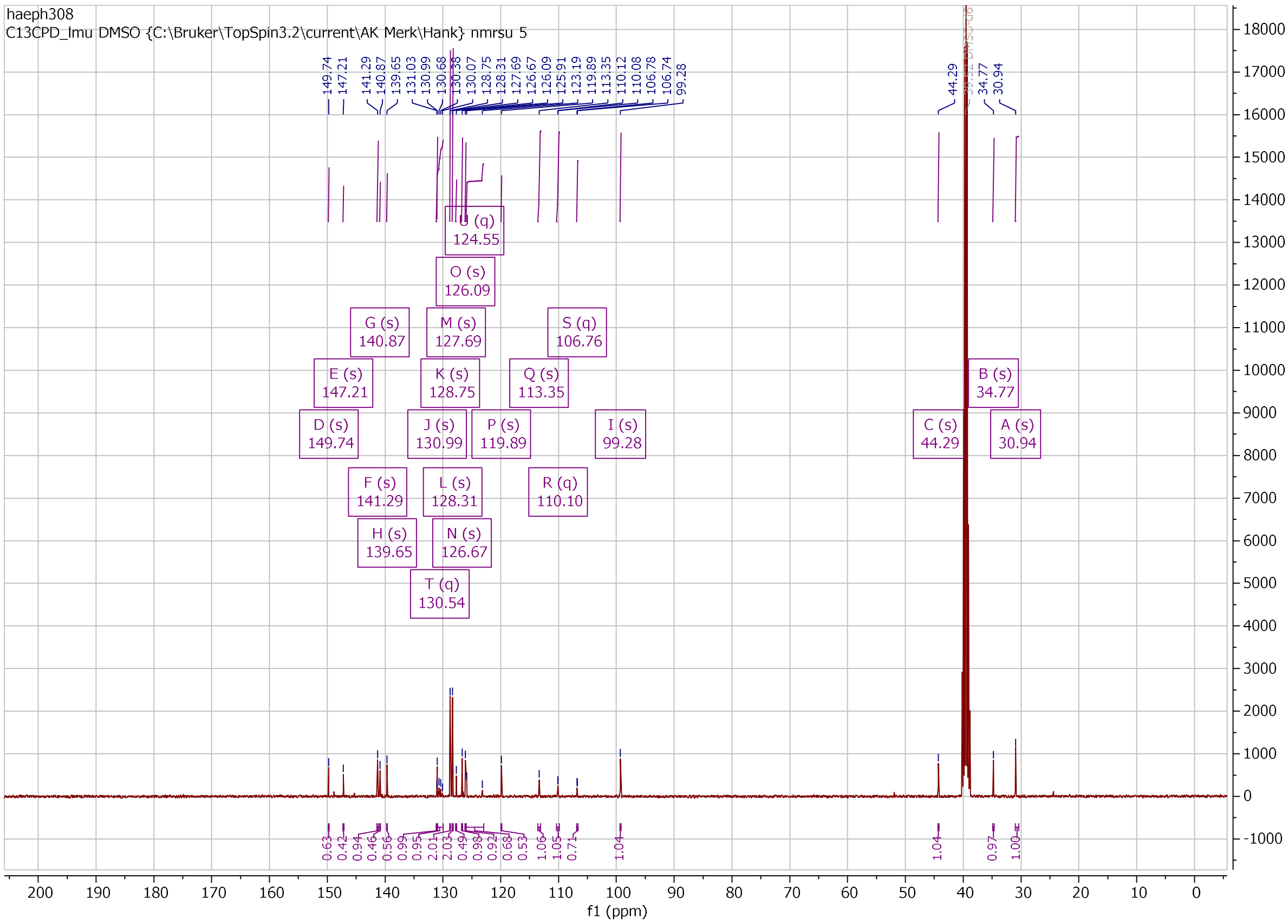
haeph308
C13CPD_lmu DMSO {C:\Bruker\TopSpin3.2\current\AK Merk\Hank} nmrsu 5
f1 (ppm)

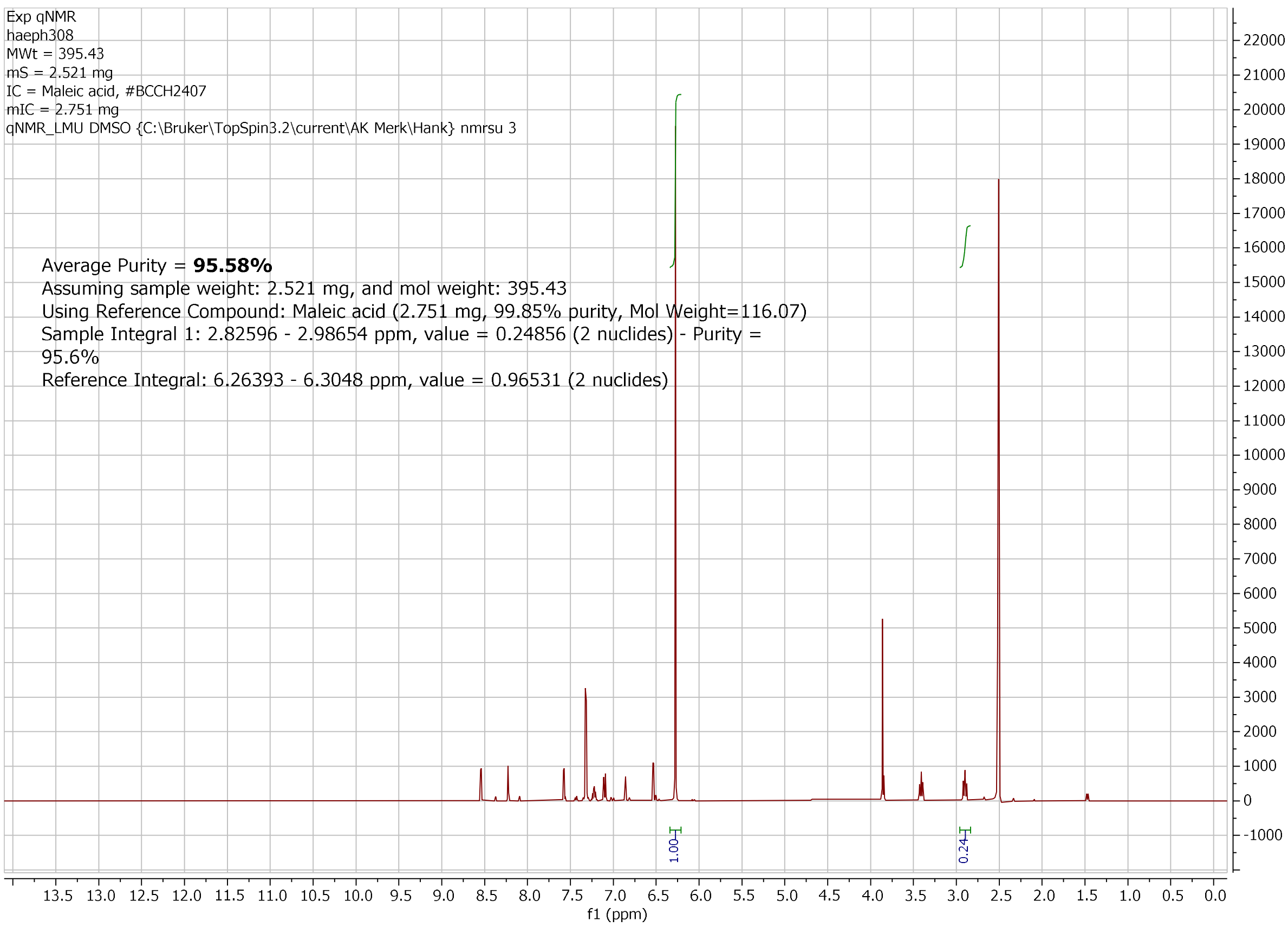
Exp qNMR
haeph308
MWt = 395.43
mS = 2.521 mg
IC = Maleic acid, #BCCH2407
mIC = 2.751 mg
qNMR_LMU DMSO {C:\Bruker\TopSpin3.2\current\AK Merk\Hank} nmrsu 3
Average Purity = 95.58%
Assuming sample weight: 2.521 mg, and mol weight: 395.43
Using Reference Compound: Maleic acid (2.751 mg, 99.85% purity, Mol Weight=116.07)
Sample Integral 1: 2.82596 - 2.98654 ppm, value = 0.24856 (2 nuclides) - Purity = 95.6%
Reference Integral: 6.26393 - 6.3048 ppm, value = 0.96531 (2 nuclides)
f1 (ppm)

Compound **22**:

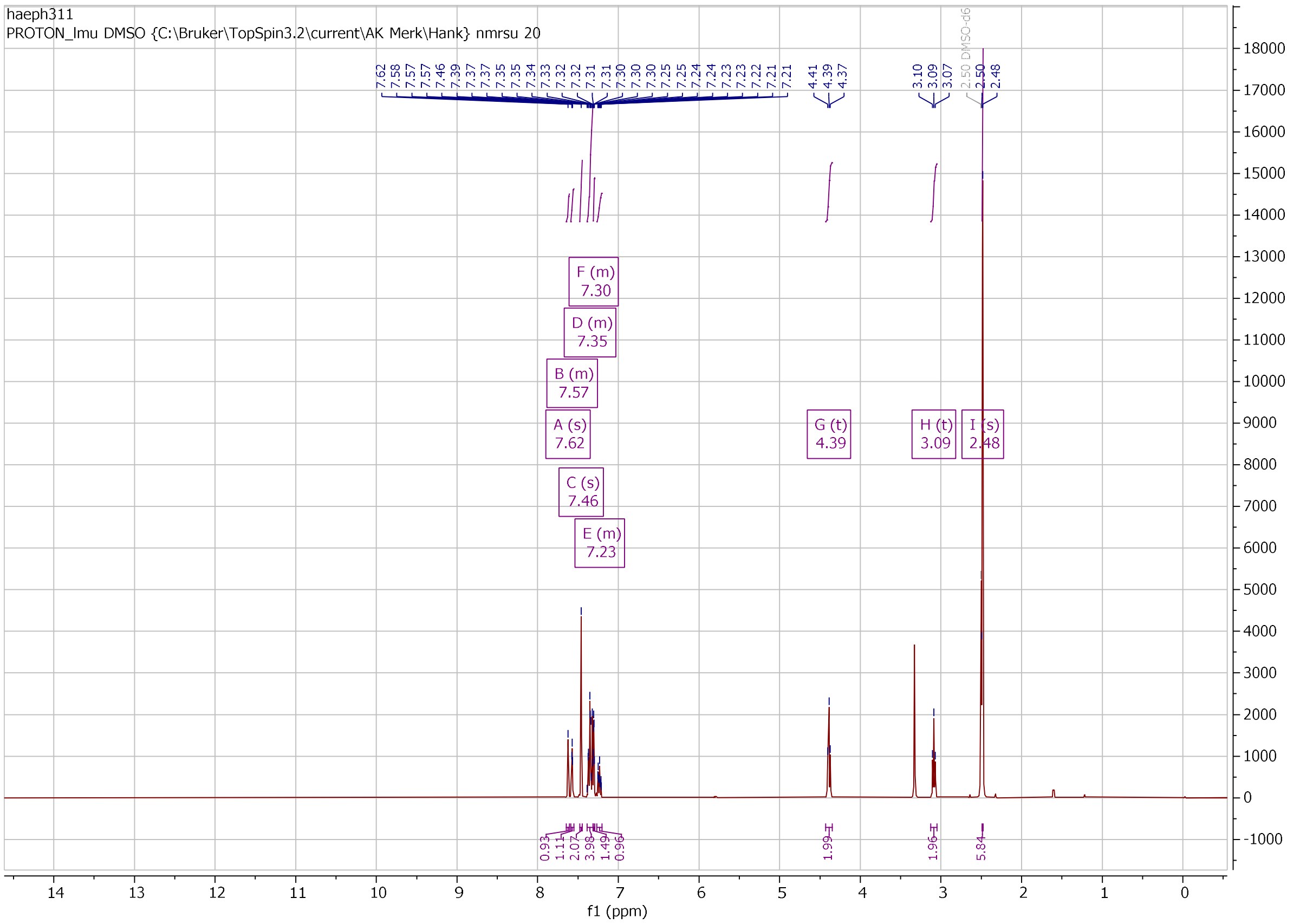

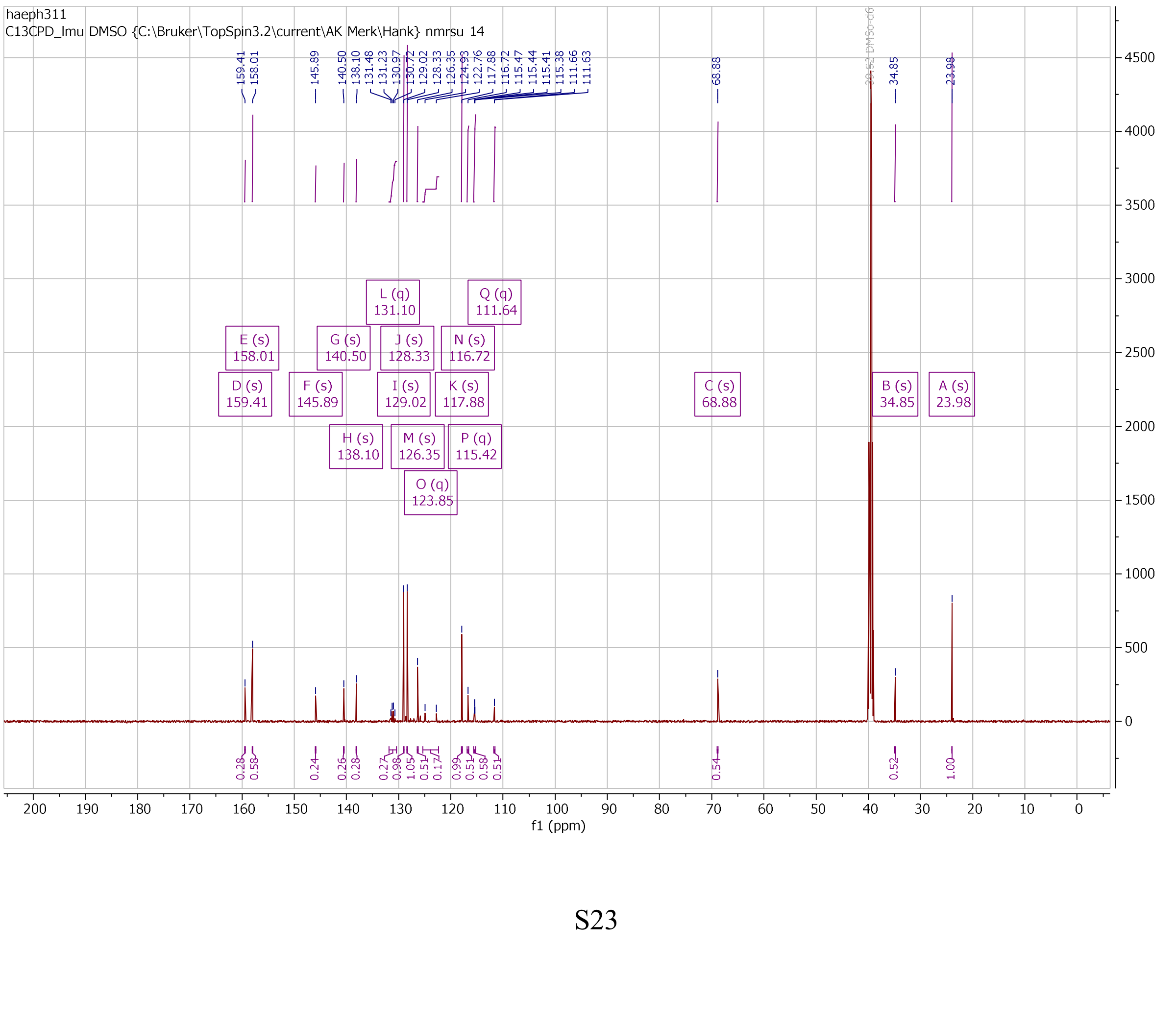

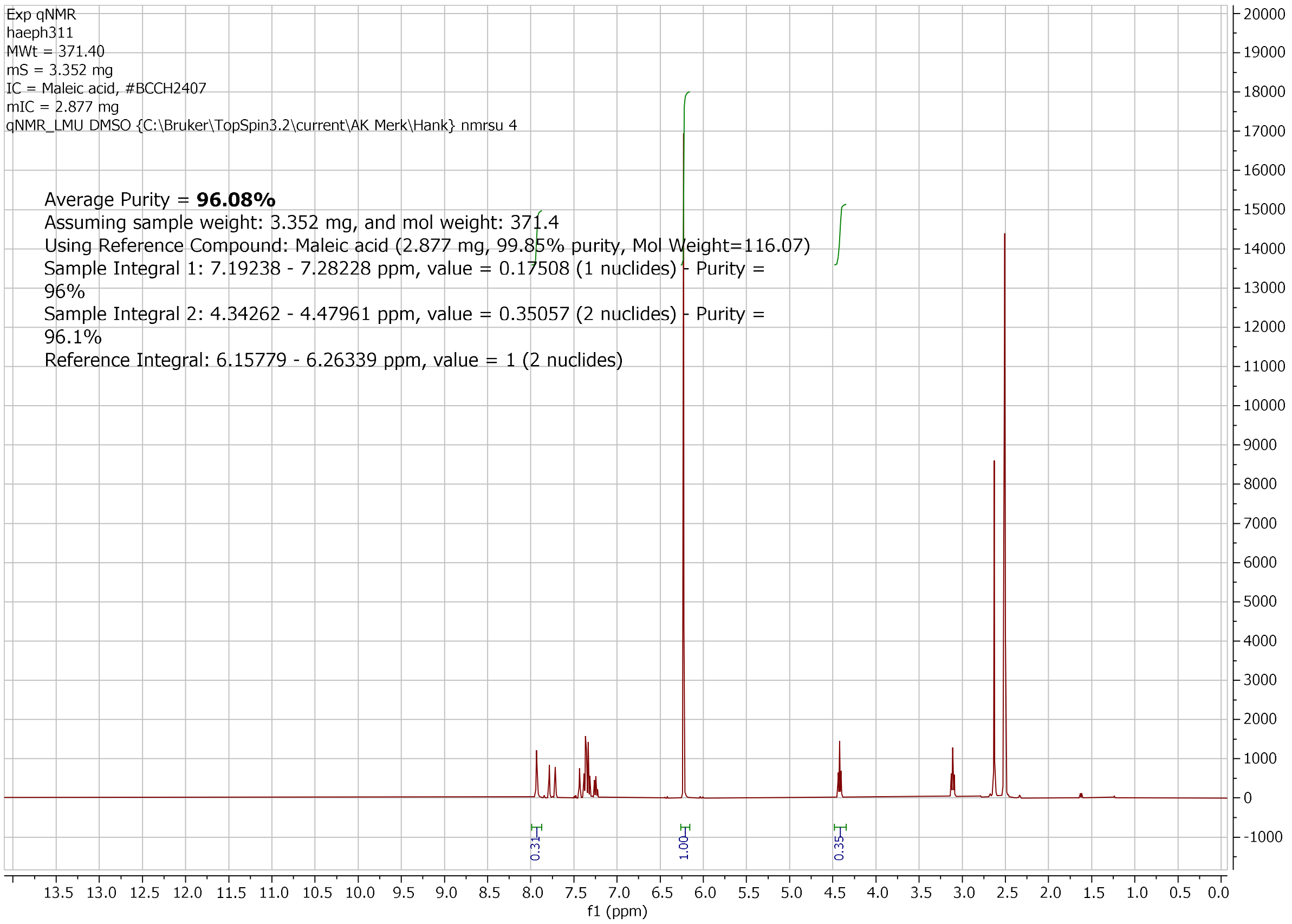
Exp qNMR
haeph311
MWt = 371.40
mS = 3.352 mg
IC = Maleic acid, #BCCH2407
mIC = 2.877 mg
qNMR_LMU DMSO {C:\Bruker\TopSpin3.2\current\AK Merk\Hank} nmrsu 4
Average Purity = **96.08%**
Assuming sample weight: 3.352 mg, and mol weight: 371.4
Using Reference Compound: Maleic acid (2.877 mg, 99.85% purity, Mol Weight=116.07)
Sample Integral 1: 7.19238 - 7.28228 ppm, value = 0.17508 (1 nuclides) - Purity = 96%
Sample Integral 2: 4.34262 - 4.47961 ppm, value = 0.35057 (2 nuclides) - Purity = 96.1%
Reference Integral: 6.15779 - 6.26339 ppm, value = 1 (2 nuclides)
f1 (ppm)

Compound **23**:

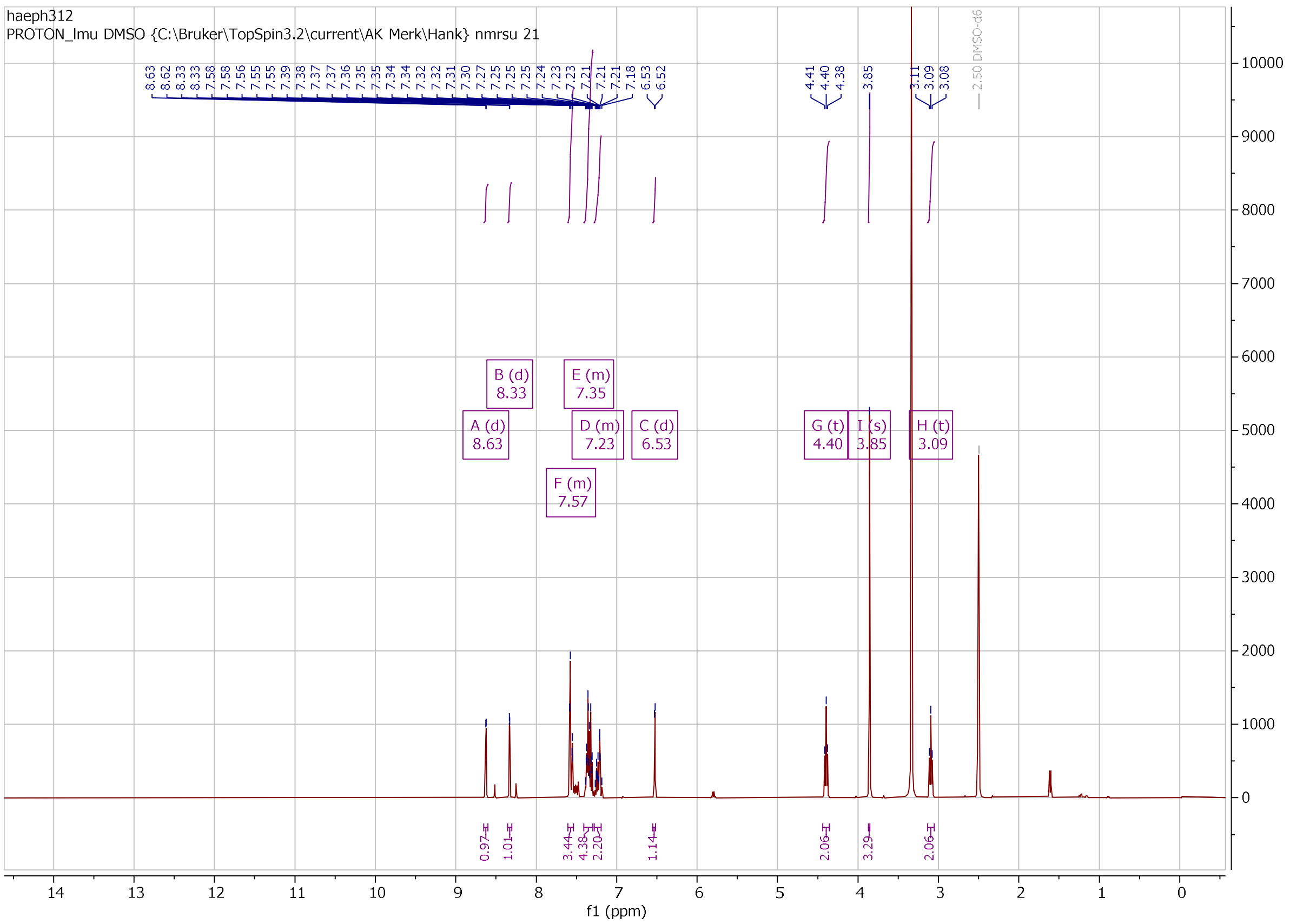
haeph312
PROTON_lmu DMSO {C:\Bruker\TopSpin3.2\current\AK Merk\Hank} nmrsu 21
A (d) 8.63
B (d) 8.33
C (d) 6.53
D (m) 7.23
E (m) 7.35
F (m) 7.57
G (t) 4.40
H (t) 3.09
I (s) 3.85
f1 (ppm)

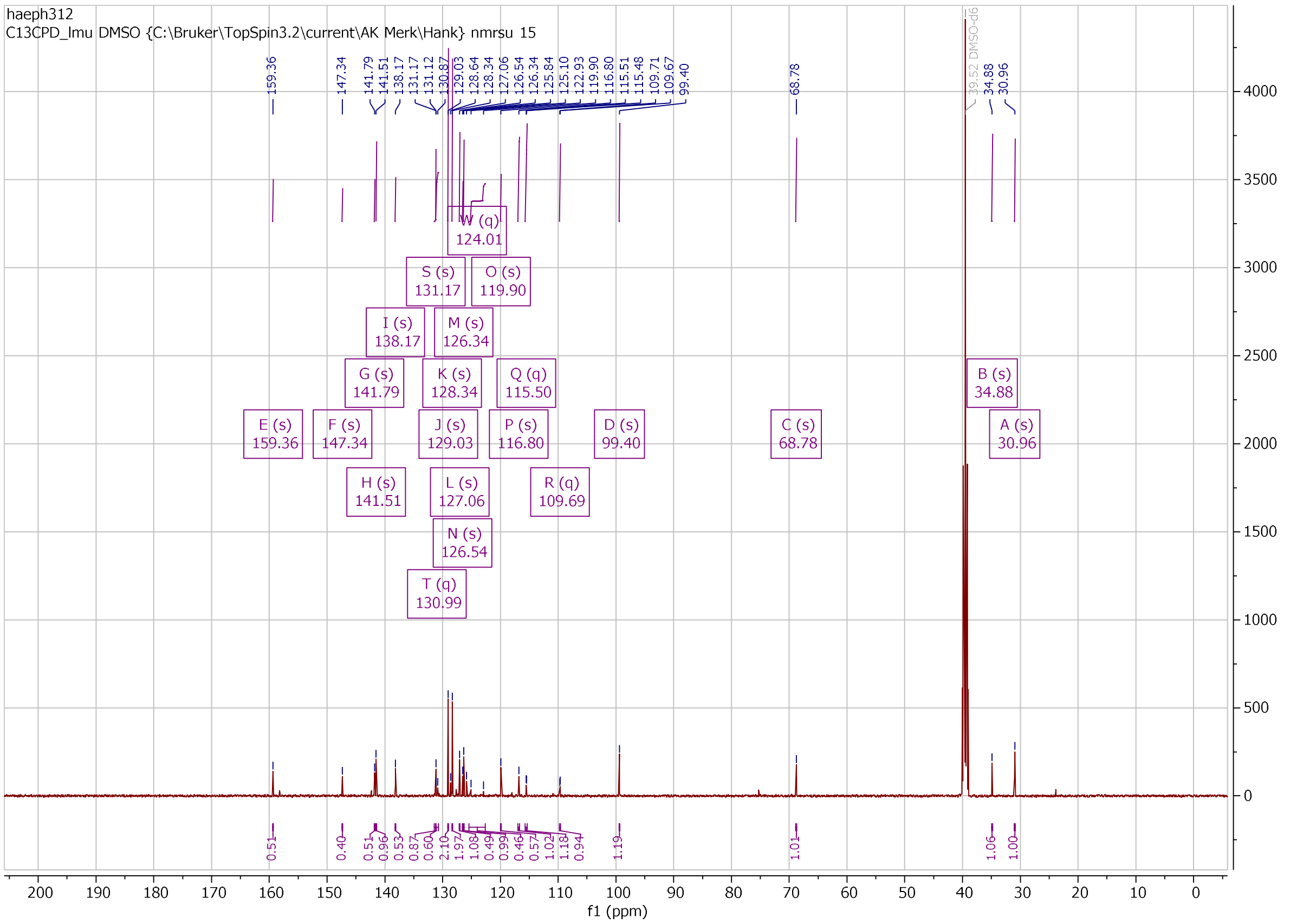
haeph312
C13CPD_lmu DMSO {C:\Bruker\TopSpin3.2\current\AK Merk\Hank} nmrsu 15
f1 (ppm)

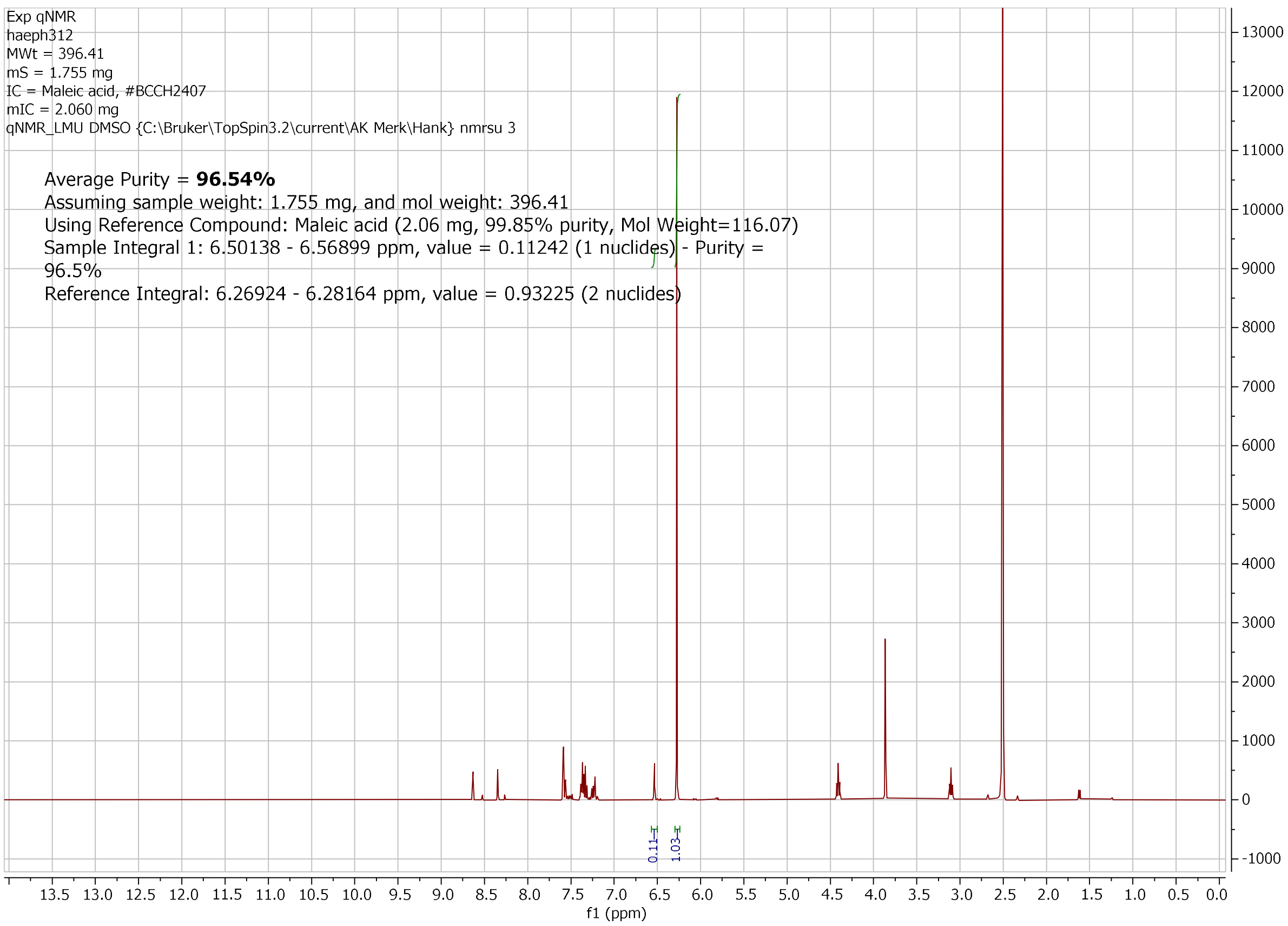
Exp qNMR
haeph312
MWt = 396.41
mS = 1.755 mg
IC = Maleic acid, #BCCH2407
mIC = 2.060 mg
qNMR_LMU DMSO {C:\Bruker\TopSpin3.2\current\AK Merk\Hank} nmrsu 3
Average Purity = **96.54%**
Assuming sample weight: 1.755 mg, and mol weight: 396.41
Using Reference Compound: Maleic acid (2.06 mg, 99.85% purity, Mol Weight=116.07)
Sample Integral 1: 6.50138 - 6.56899 ppm, value = 0.11242 (1 nuclides) - Purity = 96.5%
Reference Integral: 6.26924 - 6.28164 ppm, value = 0.93225 (2 nuclides)
f1 (ppm)

Compound **24**:

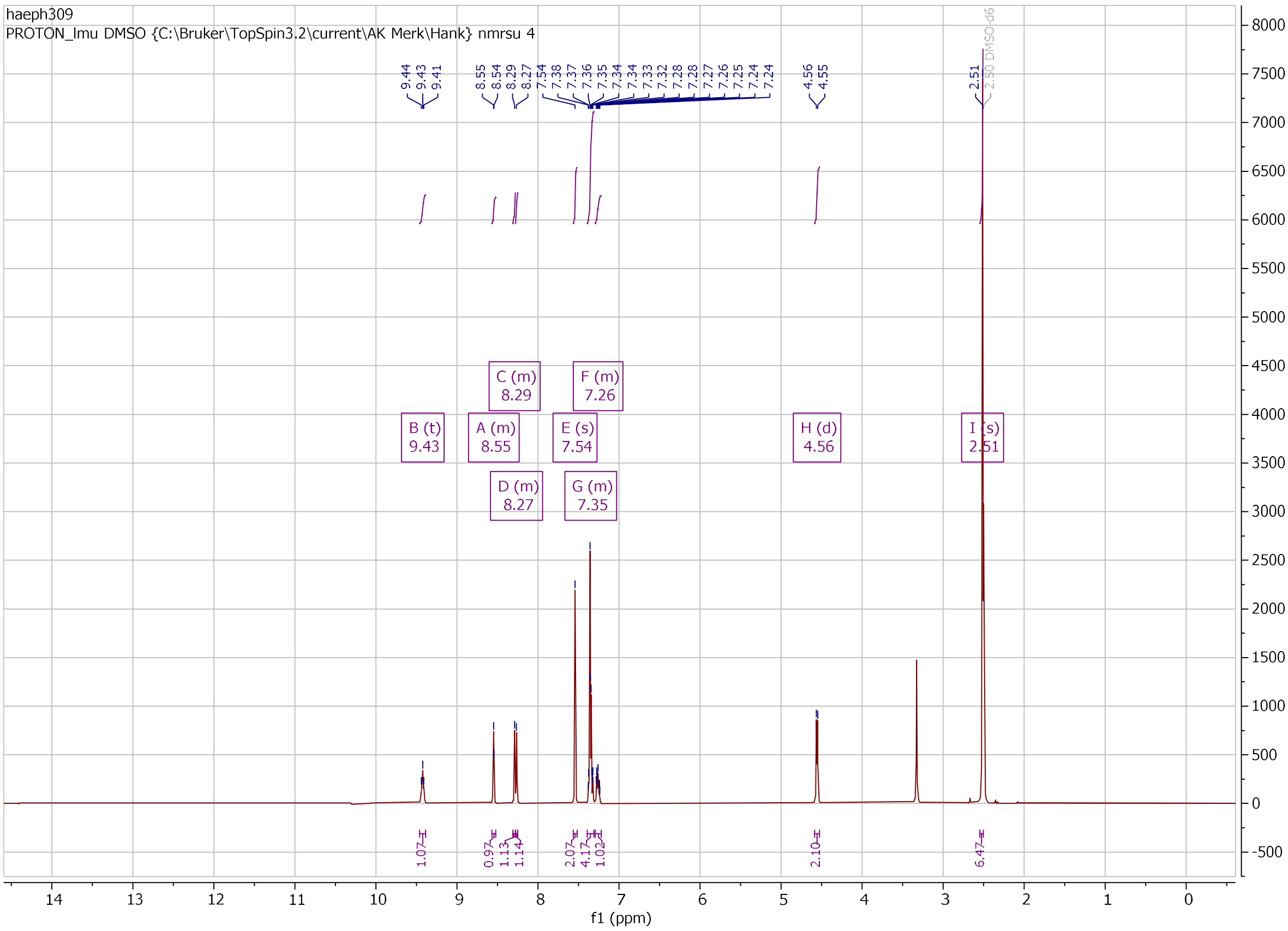
haeph309
PROTON_Imu DMSO {C:\Bruker\TopSpin3.2\current\AK Merk\Hank} nmrsu 4
B (t) 9.43
A (m) 8.55
C (m) 8.29
D (m) 8.27
E (s) 7.54
F (m) 7.26
G (m) 7.35
H (d) 4.56
I (s) 2.51
f1 (ppm)

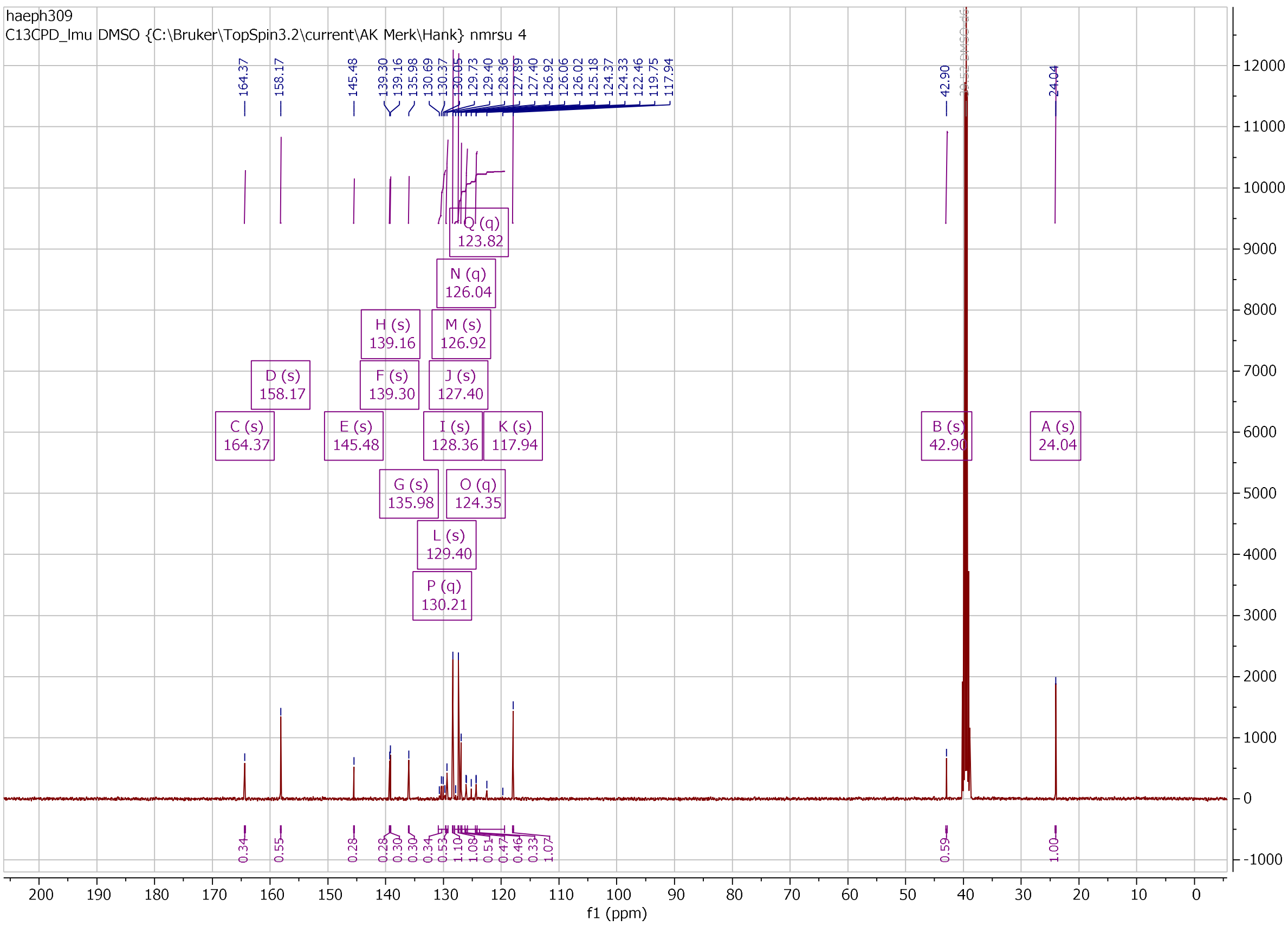
haeph309
C13CPD_Imu DMSO {C:\Bruker\TopSpin3.2\current\AK Merk\Hank} nmrsu 4
Q (q) 123.82
N (q) 126.04
H (s) 139.16
M (s) 126.92
D (s) 158.17
F (s) 139.30
J (s) 127.40
C (s) 164.37
E (s) 145.48
I (s) 128.36
K (s) 117.94
B (s) 42.90
A (s) 24.04
G (s) 135.98
O (q) 124.35
L (s) 129.40
P (q) 130.21
f1 (ppm)

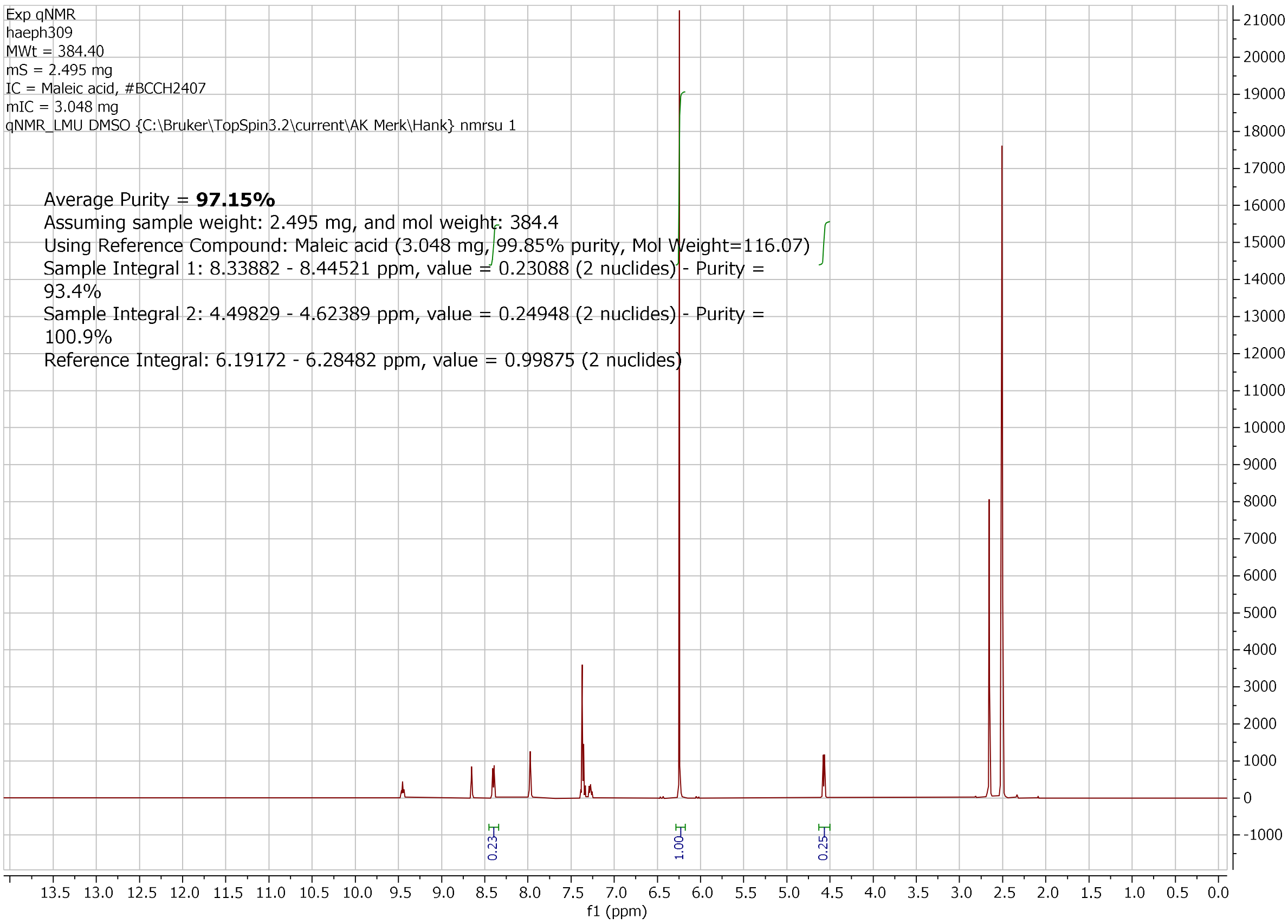
Exp qNMR
haeph309
MWt = 384.40
mS = 2.495 mg
IC = Maleic acid, #BCCH2407
mIC = 3.048 mg
qNMR_LMU DMSO {C:\Bruker\TopSpin3.2\current\AK Merk\Hank} nmrsu 1
Average Purity = 97.15%
Assuming sample weight: 2.495 mg, and mol weight: 384.4
Using Reference Compound: Maleic acid (3.048 mg, 99.85% purity, Mol Weight=116.07)
Sample Integral 1: 8.33882 - 8.44521 ppm, value = 0.23088 (2 nuclides) - Purity = 93.4%
Sample Integral 2: 4.49829 - 4.62389 ppm, value = 0.24948 (2 nuclides) - Purity = 100.9%
Reference Integral: 6.19172 - 6.28482 ppm, value = 0.99875 (2 nuclides)
f1 (ppm)

Compound **25**:

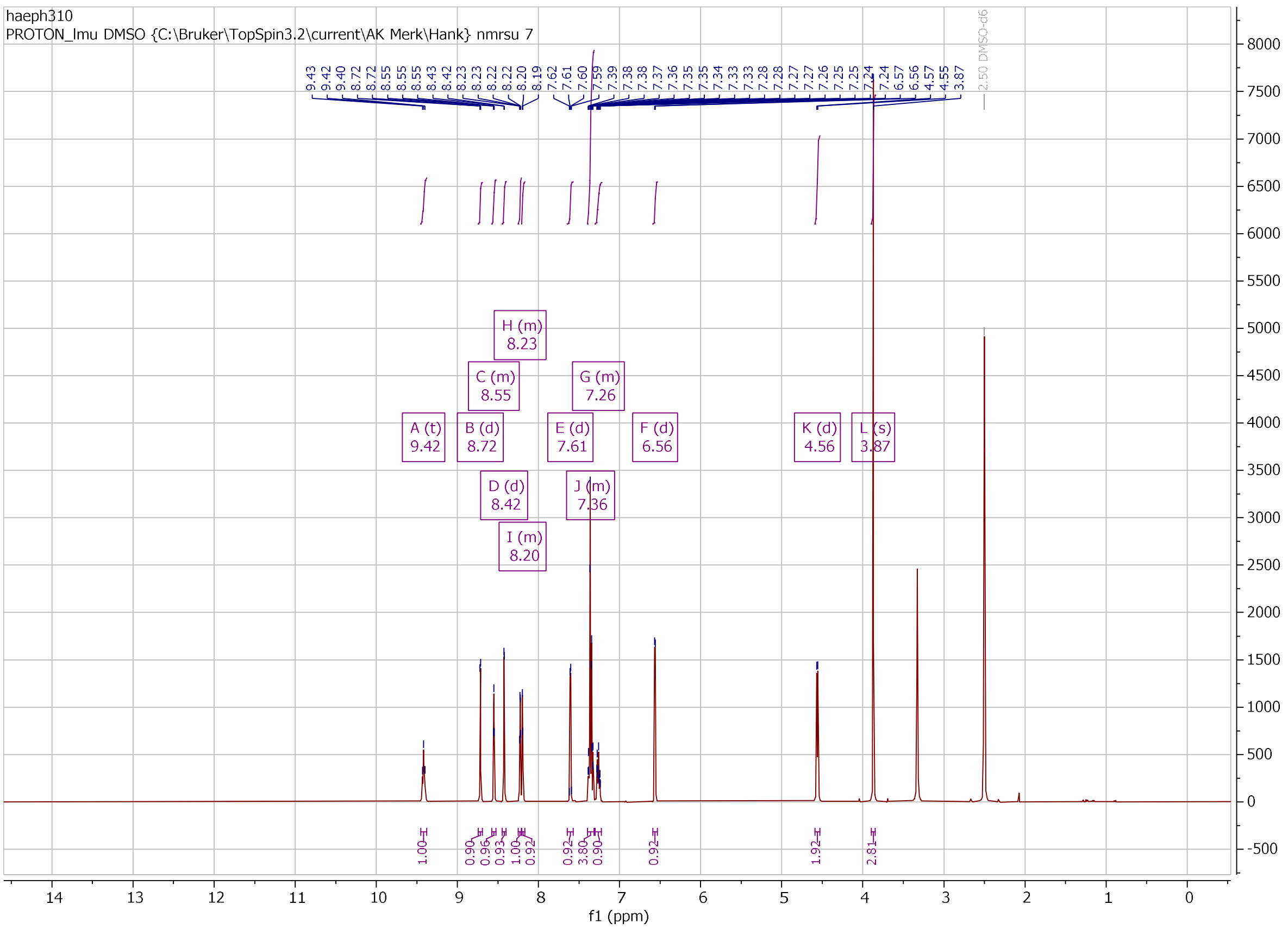
haeph310
PROTON_lmu DMSO {C:\Bruker\TopSpin3.2\current\AK Merk\Hank} nmrsu 7
A (t) 9.42
B (d) 8.72
C (m) 8.55
D (d) 8.42
H (m) 8.23
I (m) 8.20
E (d) 7.61
G (m) 7.26
J (m) 7.36
F (d) 6.56
K (d) 4.56
L (s) 3.87
f1 (ppm)

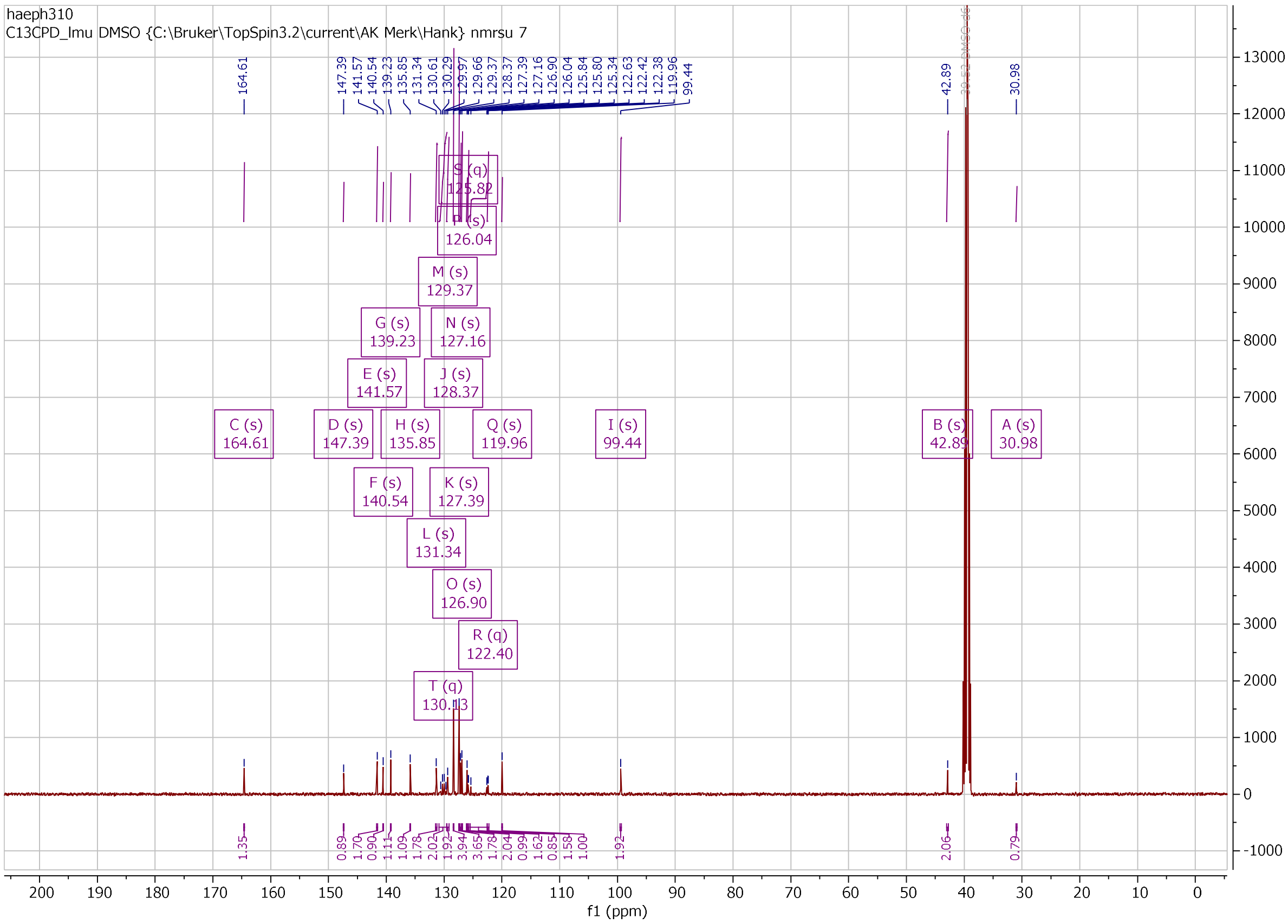
haeph310
C13CPD_lmu DMSO {C:\Bruker\TopSpin3.2\current\AK Merk\Hank} nmrsu 7
164.61
147.39
141.57
140.54
139.23
135.85
131.34
130.61
130.29
129.97
129.66
129.37
128.37
127.39
127.16
126.90
126.04
125.84
125.80
125.34
122.63
122.42
122.38
119.96
99.44
42.89
30.98
C (s) 164.61
D (s) 147.39
E (s) 141.57
F (s) 140.54
G (s) 139.23
H (s) 135.85
I (s) 99.44
J (s) 128.37
K (s) 127.39
L (s) 131.34
M (s) 129.37
N (s) 127.16
O (s) 126.90
Q (s) 119.96
R (q) 122.40
T (q) 130.13
B (s) 42.89
A (s) 30.98
f1 (ppm)

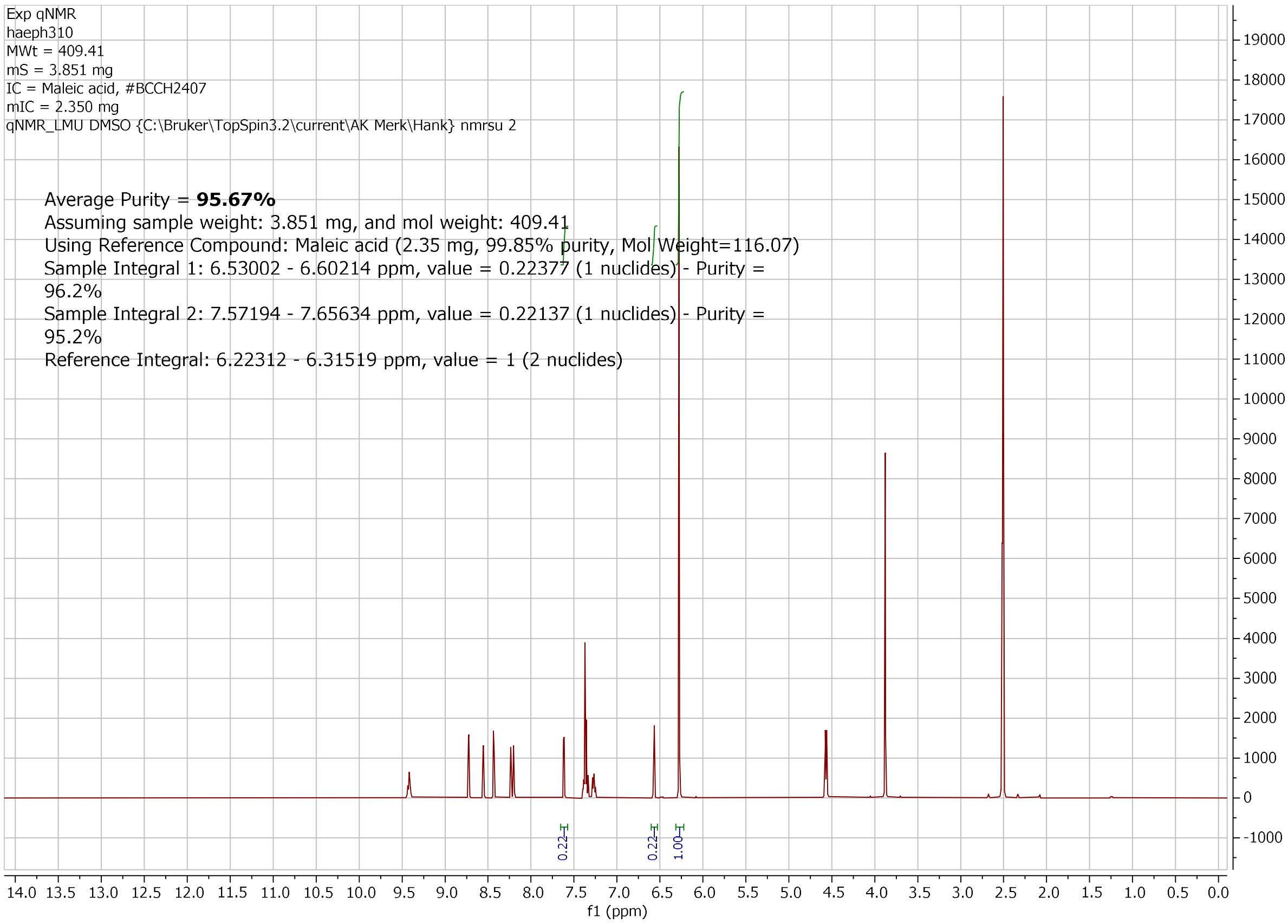
Exp qNMR
haeph310
MWt = 409.41
mS = 3.851 mg
IC = Maleic acid, #BCCH2407
mIC = 2.350 mg
qNMR_LMU DMSO {C:\Bruker\TopSpin3.2\current\AK Merk\Hank} nmrsu 2
Average Purity = 95.67%
Assuming sample weight: 3.851 mg, and mol weight: 409.41
Using Reference Compound: Maleic acid (2.35 mg, 99.85% purity, Mol Weight=116.07)
Sample Integral 1: 6.53002 - 6.60214 ppm, value = 0.22377 (1 nuclides) - Purity = 96.2%
Sample Integral 2: 7.57194 - 7.65634 ppm, value = 0.22137 (1 nuclides) - Purity = 95.2%
Reference Integral: 6.22312 - 6.31519 ppm, value = 1 (2 nuclides)
0.22
0.22
1.00
f1 (ppm)

Compound **26**:

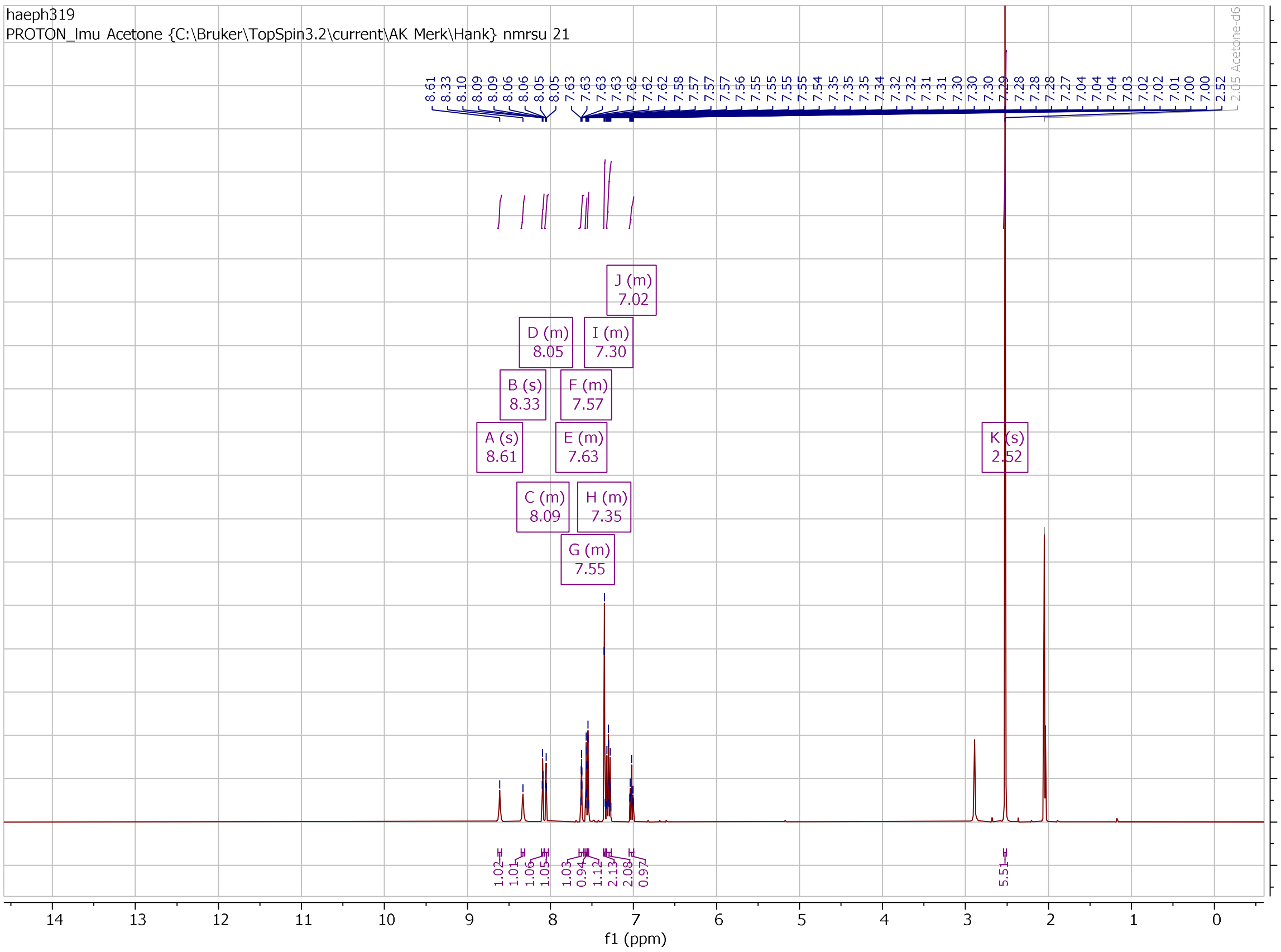

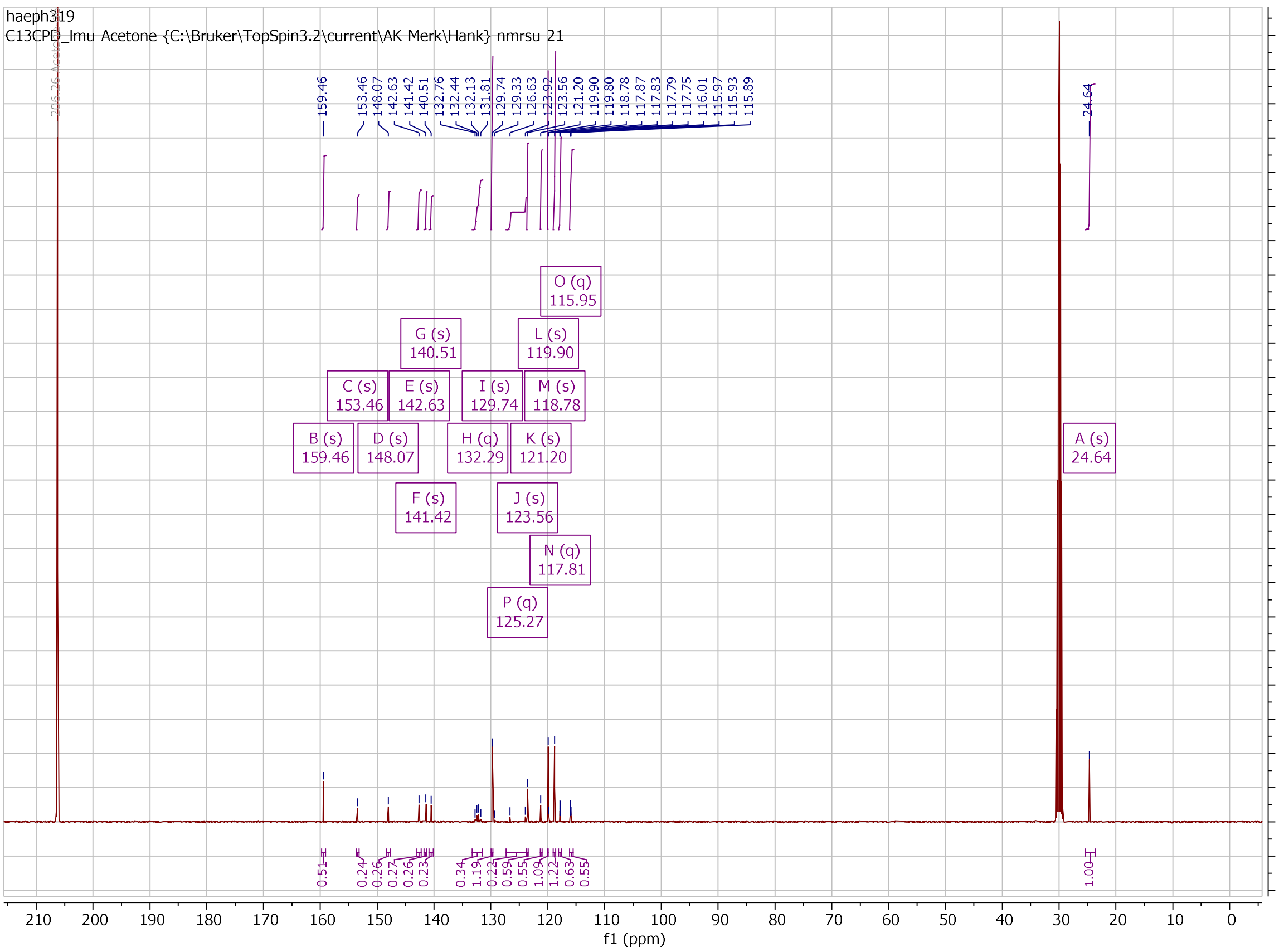

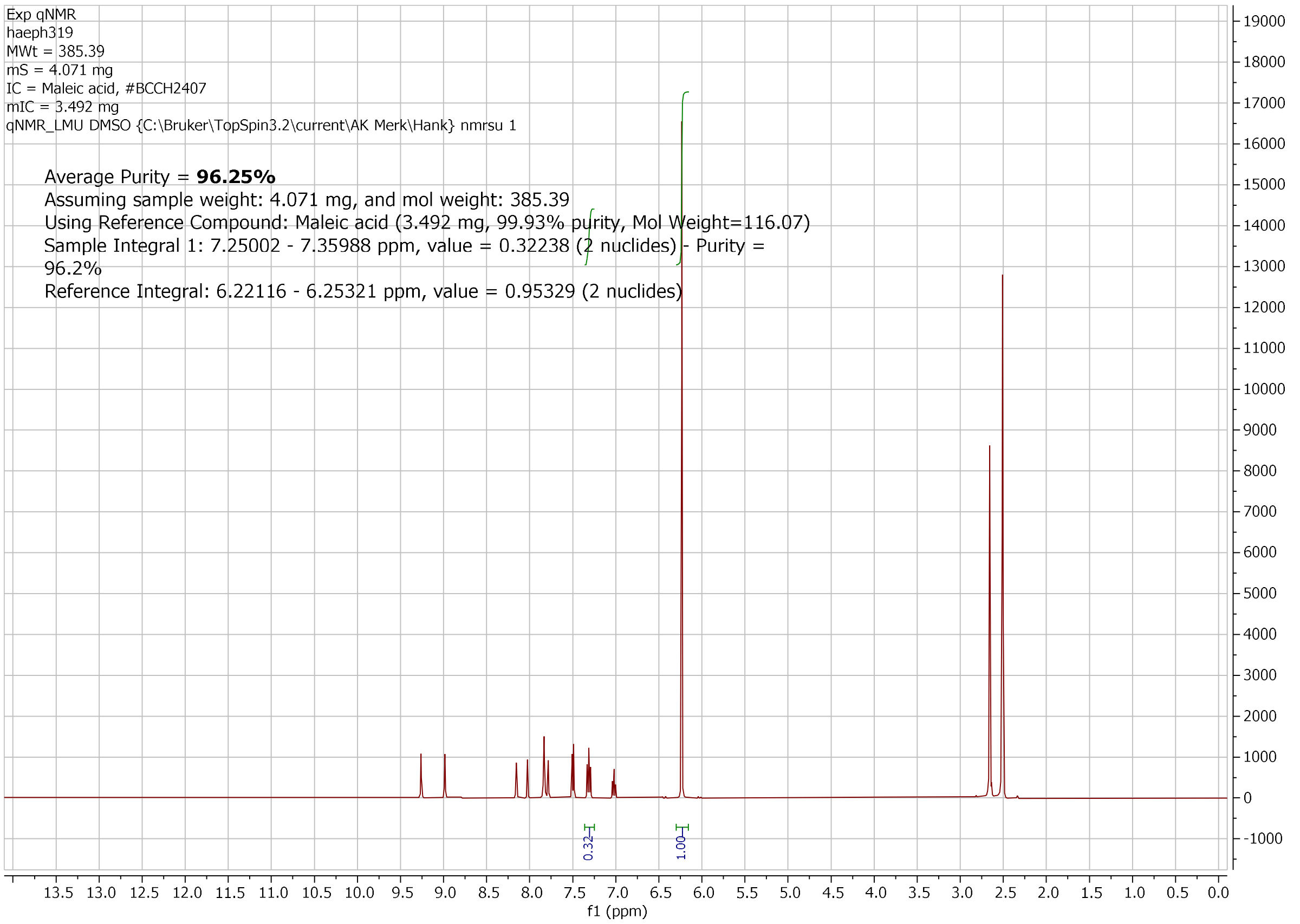
Exp qNMR
haeph319
MWt = 385.39
mS = 4.071 mg
IC = Maleic acid, #BCCH2407
mIC = 3.492 mg
qNMR_LMU DMSO {C:\Bruker\TopSpin3.2\current\AK Merk\Hank} nmrsu 1
Average Purity = 96.25%
Assuming sample weight: 4.071 mg, and mol weight: 385.39
Using Reference Compound: Maleic acid (3.492 mg, 99.93% purity, Mol Weight=116.07)
Sample Integral 1: 7.25002 - 7.35988 ppm, value = 0.32238 (2 nuclides) - Purity = 96.2%
Reference Integral: 6.22116 - 6.25321 ppm, value = 0.95329 (2 nuclides)
f1 (ppm)

Compound **27**:

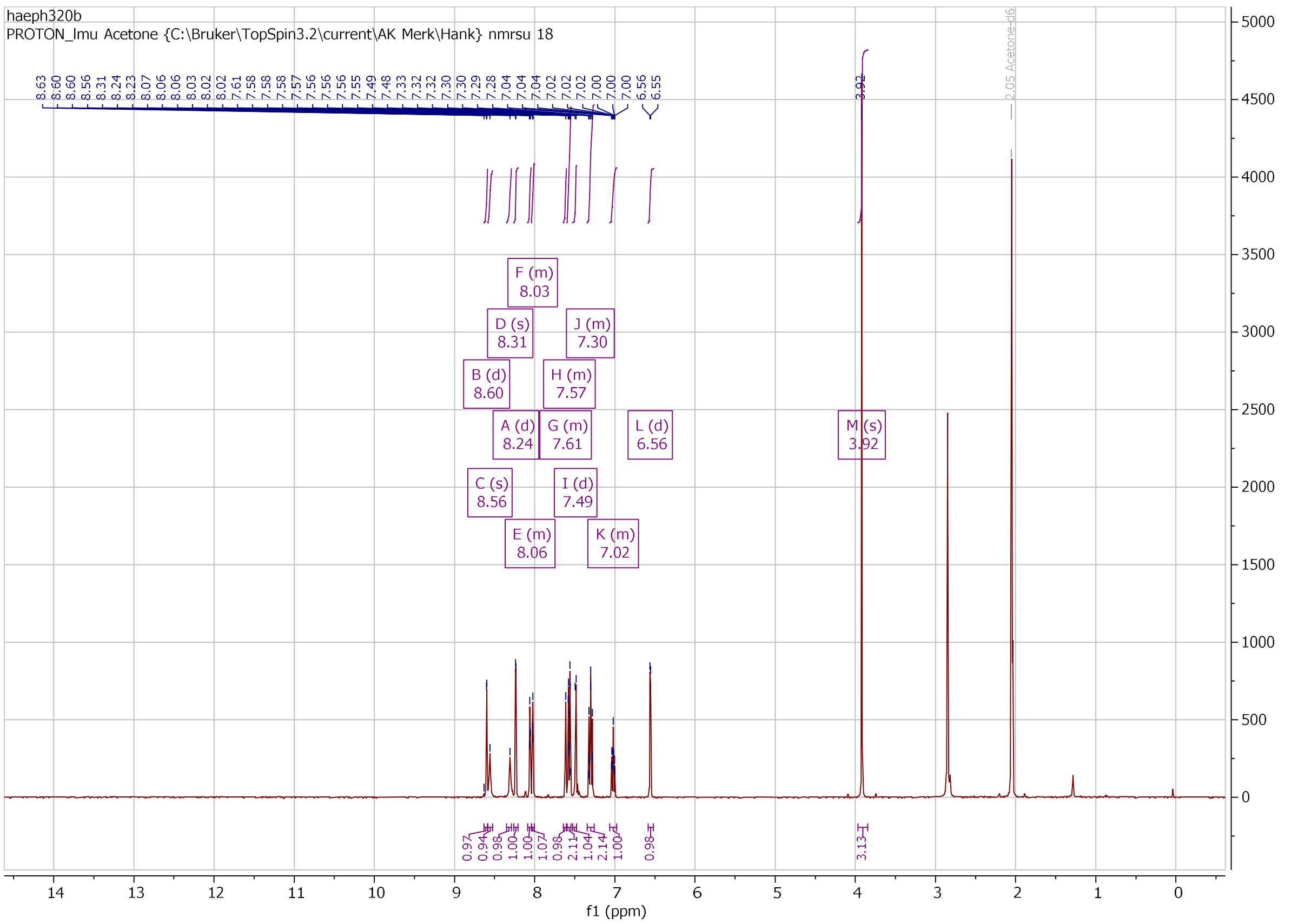
haeph320b
PROTON_lmu Acetone {C:\Bruker\TopSpin3.2\current\AK Merk\Hank} nmrsu 18
F (m) 8.03
D (s) 8.31
J (m) 7.30
B (d) 8.60
H (m) 7.57
A (d) 8.24
G (m) 7.61
L (d) 6.56
M (s) 3.92
C (s) 8.56
I (d) 7.49
E (m) 8.06
K (m) 7.02
f1 (ppm)

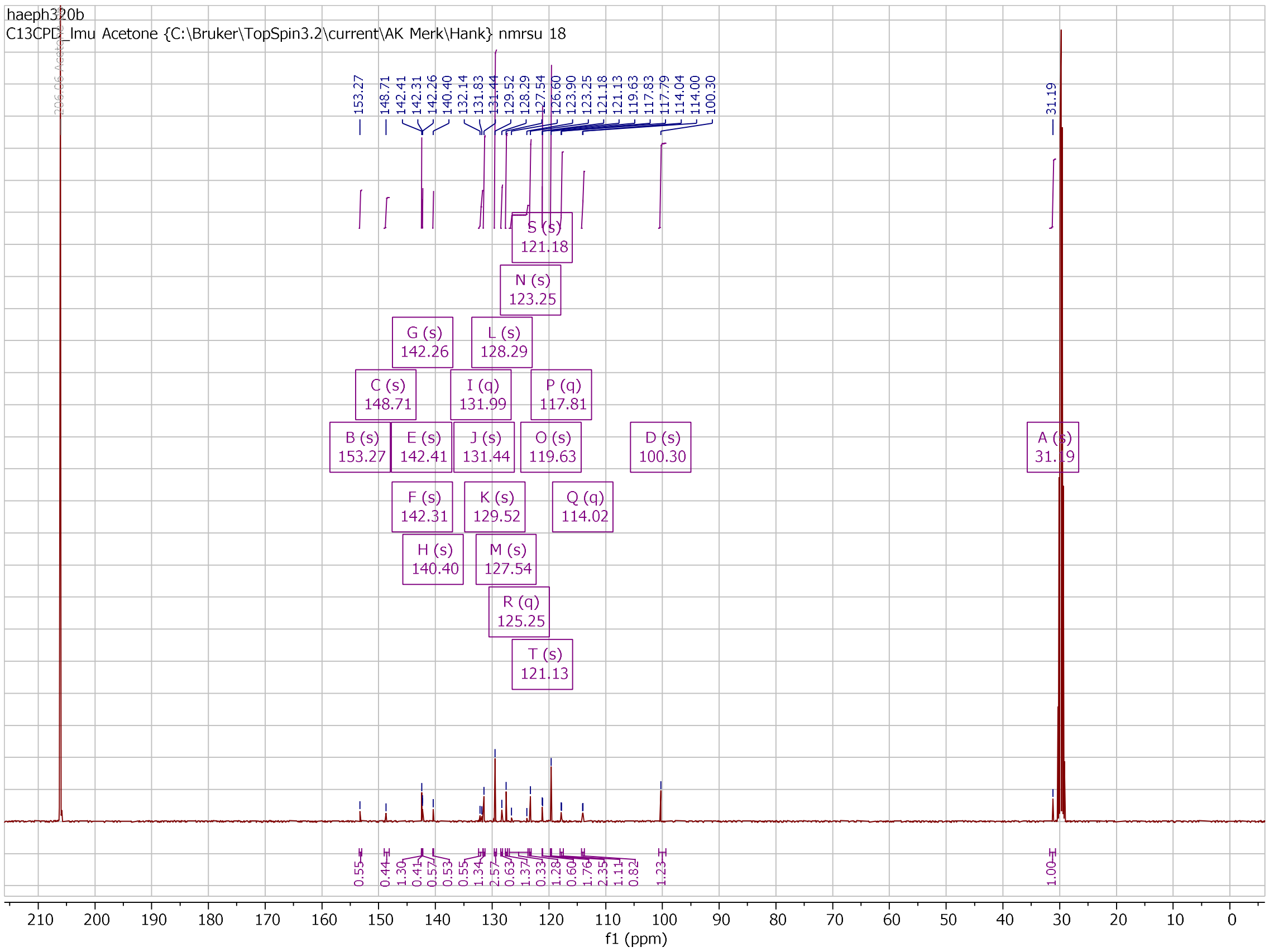
haeph320b
C13CPD_lmu Acetone {C:\Bruker\TopSpin3.2\current\AK Merk\Hank} nmrsu 18
153.27
148.71
142.41
142.31
142.26
140.40
132.14
131.83
131.44
129.52
128.29
127.54
126.60
123.90
123.25
121.18
121.13
119.63
117.83
117.79
114.04
114.00
100.30
31.19
S (s) 121.18
N (s) 123.25
G (s) 142.26
L (s) 128.29
C (s) 148.71
I (q) 131.99
P (q) 117.81
B (s) 153.27
E (s) 142.41
J (s) 131.44
O (s) 119.63
D (s) 100.30
A (s) 31.19
F (s) 142.31
K (s) 129.52
Q (q) 114.02
H (s) 140.40
M (s) 127.54
R (q) 125.25
T (s) 121.13
f1 (ppm)

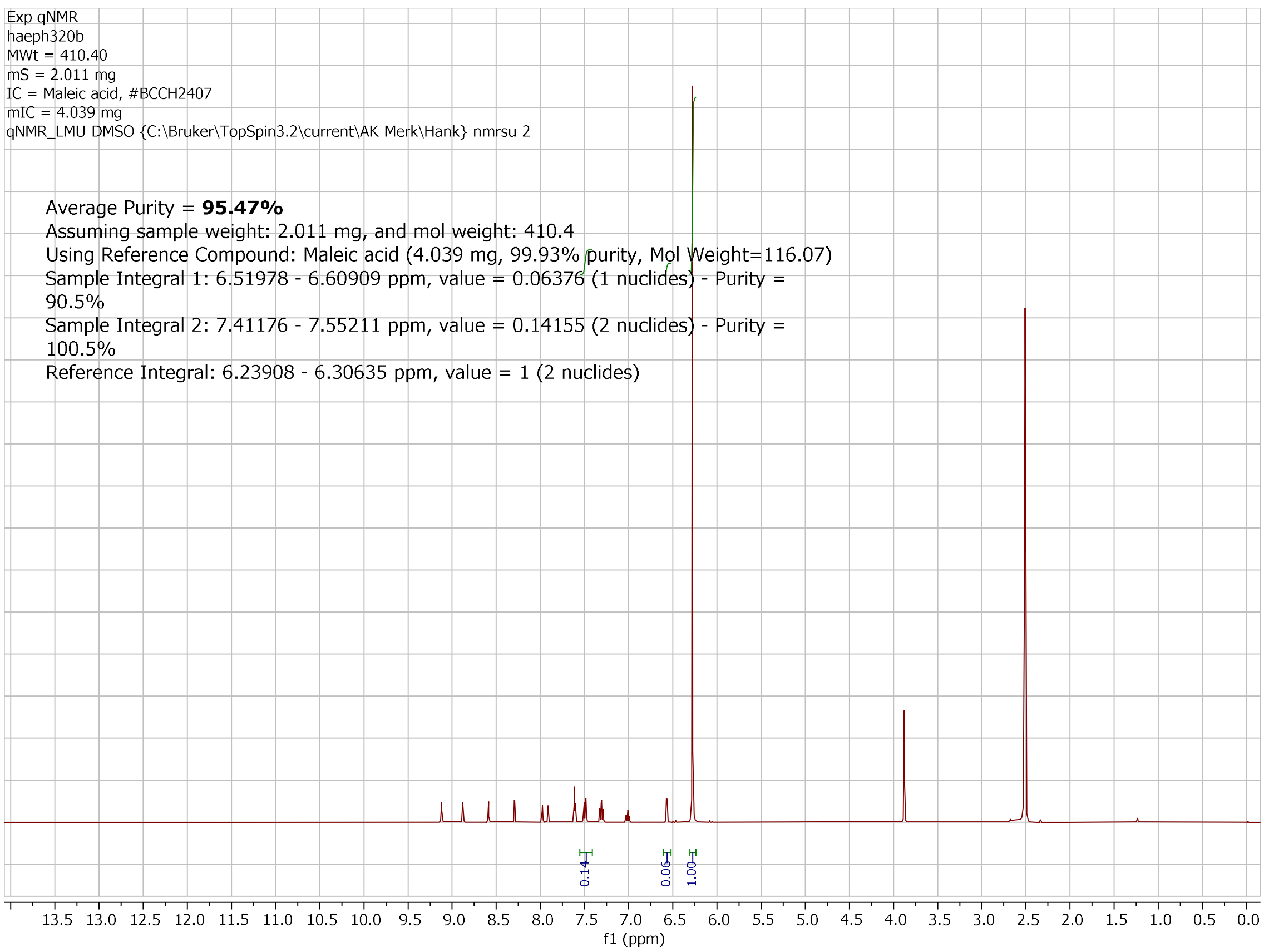
Exp qNMR
haeph320b
MWt = 410.40
mS = 2.011 mg
IC = Maleic acid, #BCCH2407
mIC = 4.039 mg
qNMR_LMU DMSO {C:\Bruker\TopSpin3.2\current\AK Merk\Hank} nmrsu 2
Average Purity = 95.47%
Assuming sample weight: 2.011 mg, and mol weight: 410.4
Using Reference Compound: Maleic acid (4.039 mg, 99.93% purity, Mol Weight=116.07)
Sample Integral 1: 6.51978 - 6.60909 ppm, value = 0.06376 (1 nuclides) - Purity = 90.5%
Sample Integral 2: 7.41176 - 7.55211 ppm, value = 0.14155 (2 nuclides) - Purity = 100.5%
Reference Integral: 6.23908 - 6.30635 ppm, value = 1 (2 nuclides)
f1 (ppm)

Compound **28**:

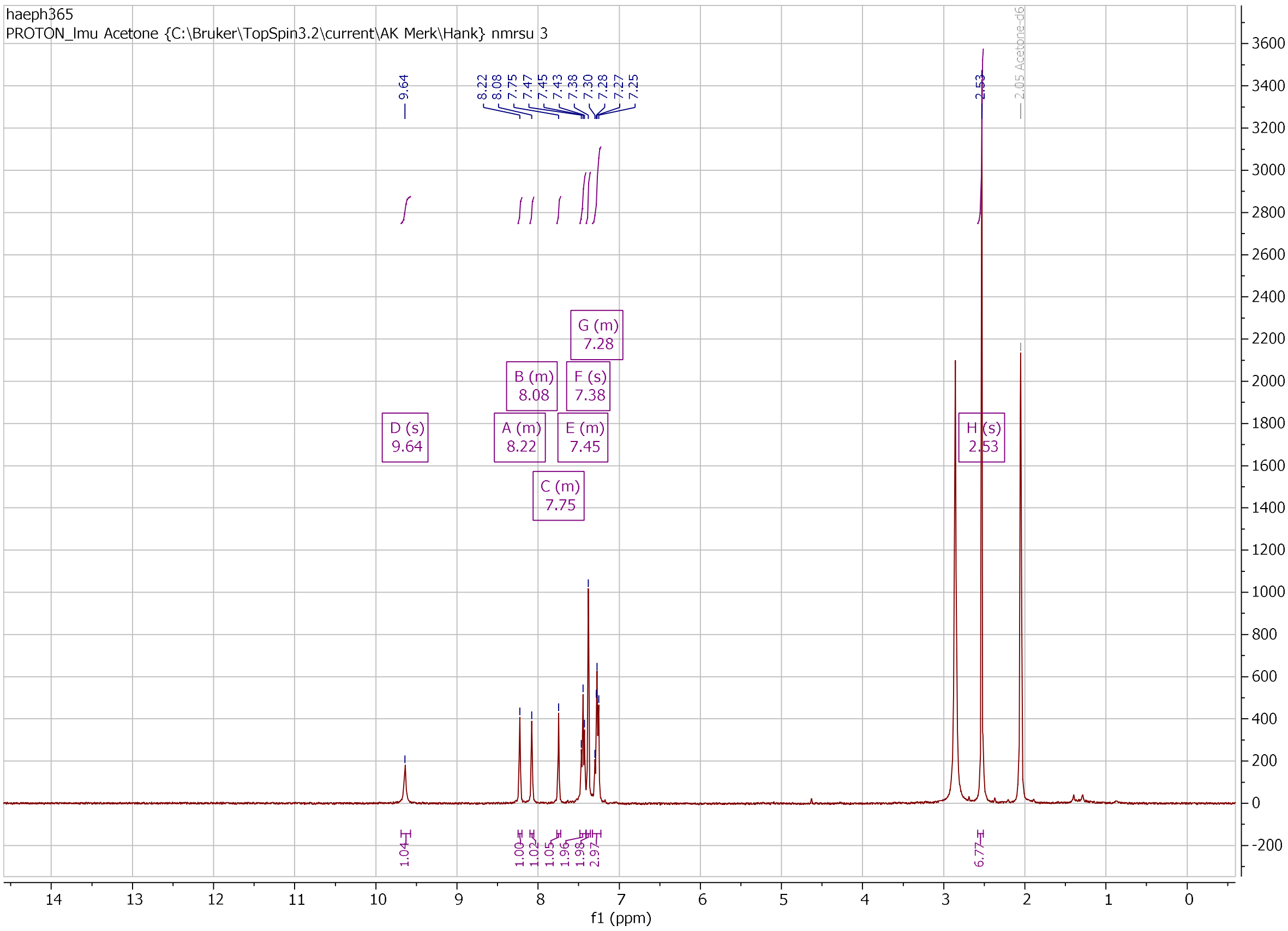

haeph365
PROTON_Imu Acetone {C:\Bruker\TopSpin3.2\current\AK Merk\Hank} nmrsu 3
9.64
8.22 8.08 7.75 7.47 7.45 7.43 7.38 7.30 7.28 7.27 7.25
2.53
2.05 Acetone-d6
G (m) 7.28
B (m) 8.08
F (s) 7.38
D (s) 9.64
A (m) 8.22
E (m) 7.45
H (s) 2.53
C (m) 7.75
1.04
1.00 1.02 1.05 1.96 1.98 2.97
6.77
f1 (ppm)

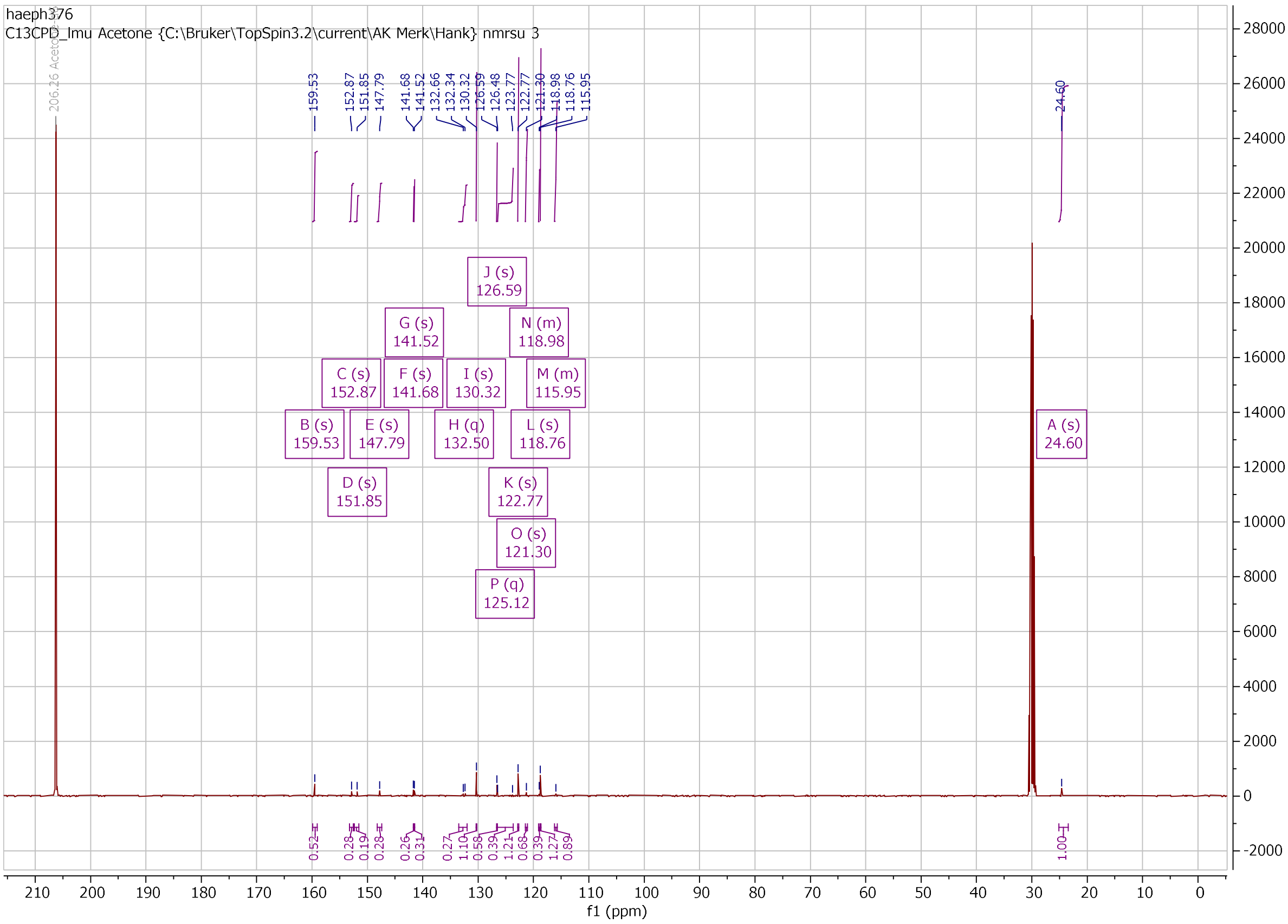

haeph376
C13CPD_Imu Acetone {C:\Bruker\TopSpin3.2\current\AK Merk\Hank} nmrsu 3
206.26 Acetone
159.53
152.87 151.85 147.79
141.68 141.52 132.66 132.34 130.32 126.59 126.48 123.77 122.77 121.30 118.98 118.76 115.95
24.60
J (s) 126.59
G (s) 141.52
N (m) 118.98
C (s) 152.87
F (s) 141.68
I (s) 130.32
M (m) 115.95
B (s) 159.53
E (s) 147.79
H (q) 132.50
L (s) 118.76
A (s) 24.60
D (s) 151.85
K (s) 122.77
O (s) 121.30
P (q) 125.12
0.52 0.28 0.19 0.28 0.26 0.31 0.27 1.10 0.58 0.39 1.21 0.68 0.39 1.27 0.89
1.00
f1 (ppm)

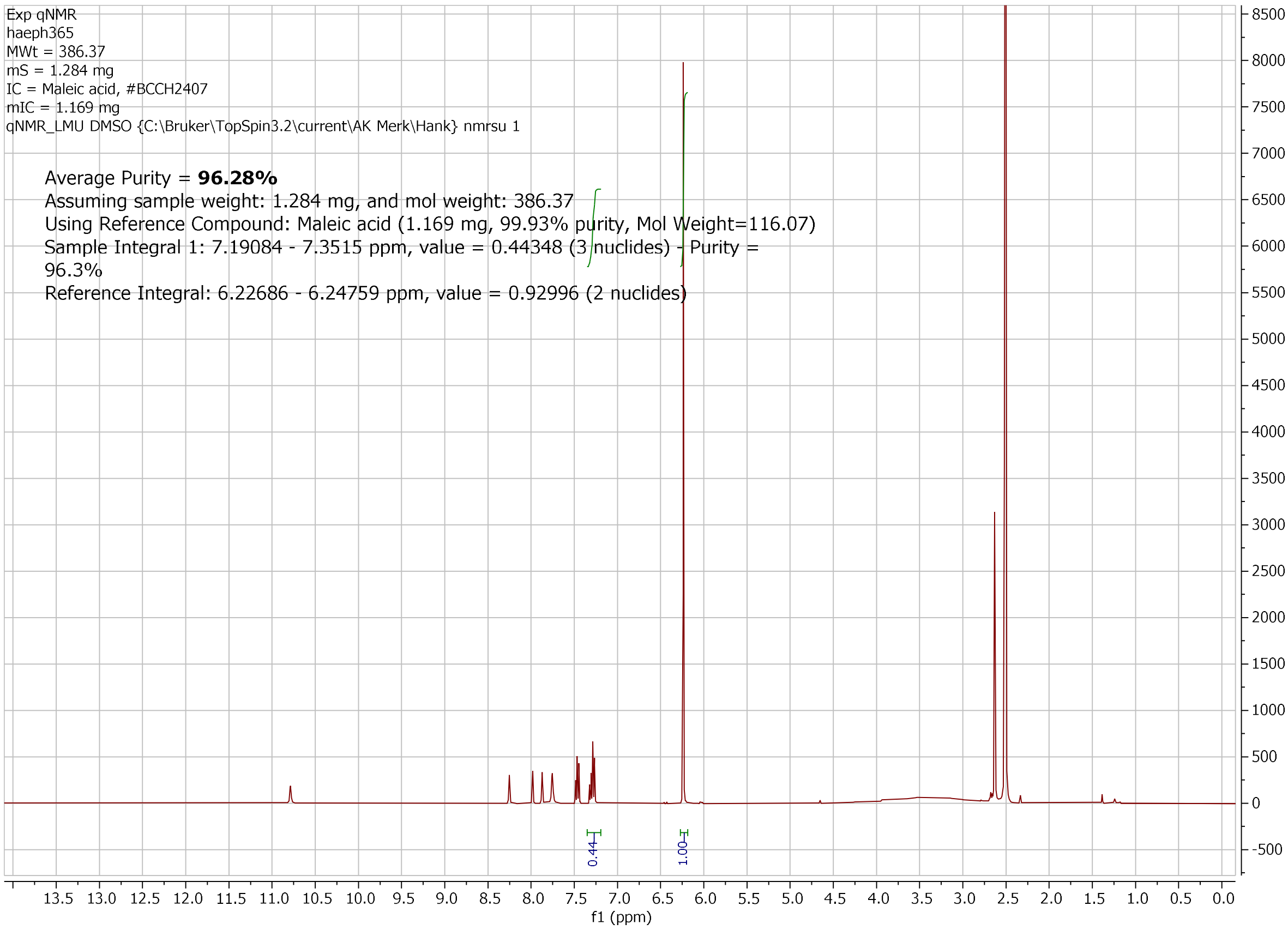
Exp qNMR
haeph365
MWt = 386.37
mS = 1.284 mg
IC = Maleic acid, #BCCH2407
mIC = 1.169 mg
qNMR_LMU DMSO {C:\Bruker\TopSpin3.2\current\AK Merk\Hank} nmrsu 1
Average Purity = 96.28%
Assuming sample weight: 1.284 mg, and mol weight: 386.37
Using Reference Compound: Maleic acid (1.169 mg, 99.93% purity, Mol Weight=116.07)
Sample Integral 1: 7.19084 - 7.3515 ppm, value = 0.44348 (3 nuclides) - Purity = 96.3%
Reference Integral: 6.22686 - 6.24759 ppm, value = 0.92996 (2 nuclides)
f1 (ppm)

Compound **29**:

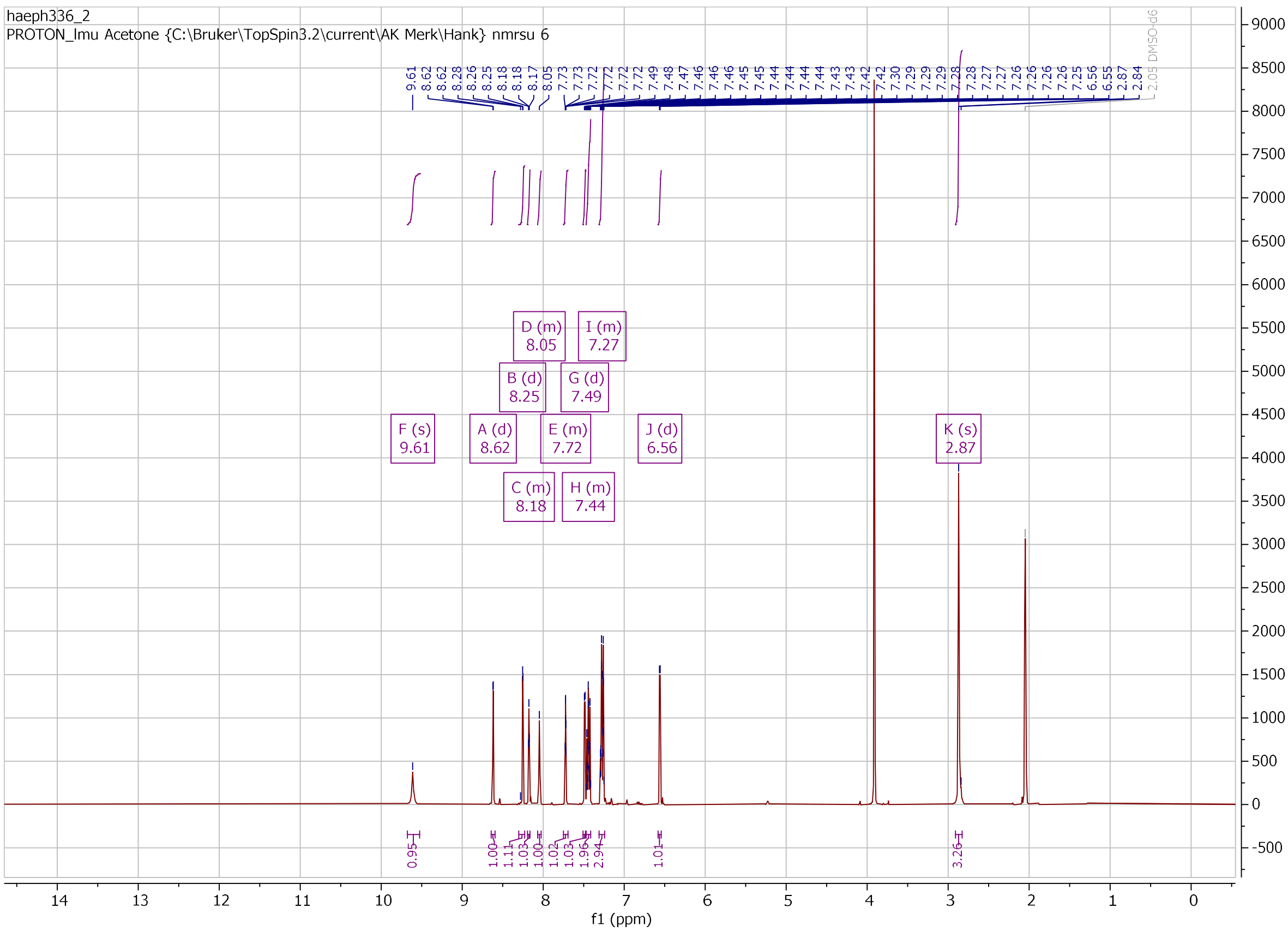
haeph336_2
PROTON_lmu Acetone {C:\Bruker\TopSpin3.2\current\AK Merk\Hank} nmrsu 6
D (m) 8.05
I (m) 7.27
B (d) 8.25
G (d) 7.49
F (s) 9.61
A (d) 8.62
E (m) 7.72
J (d) 6.56
K (s) 2.87
C (m) 8.18
H (m) 7.44
f1 (ppm)

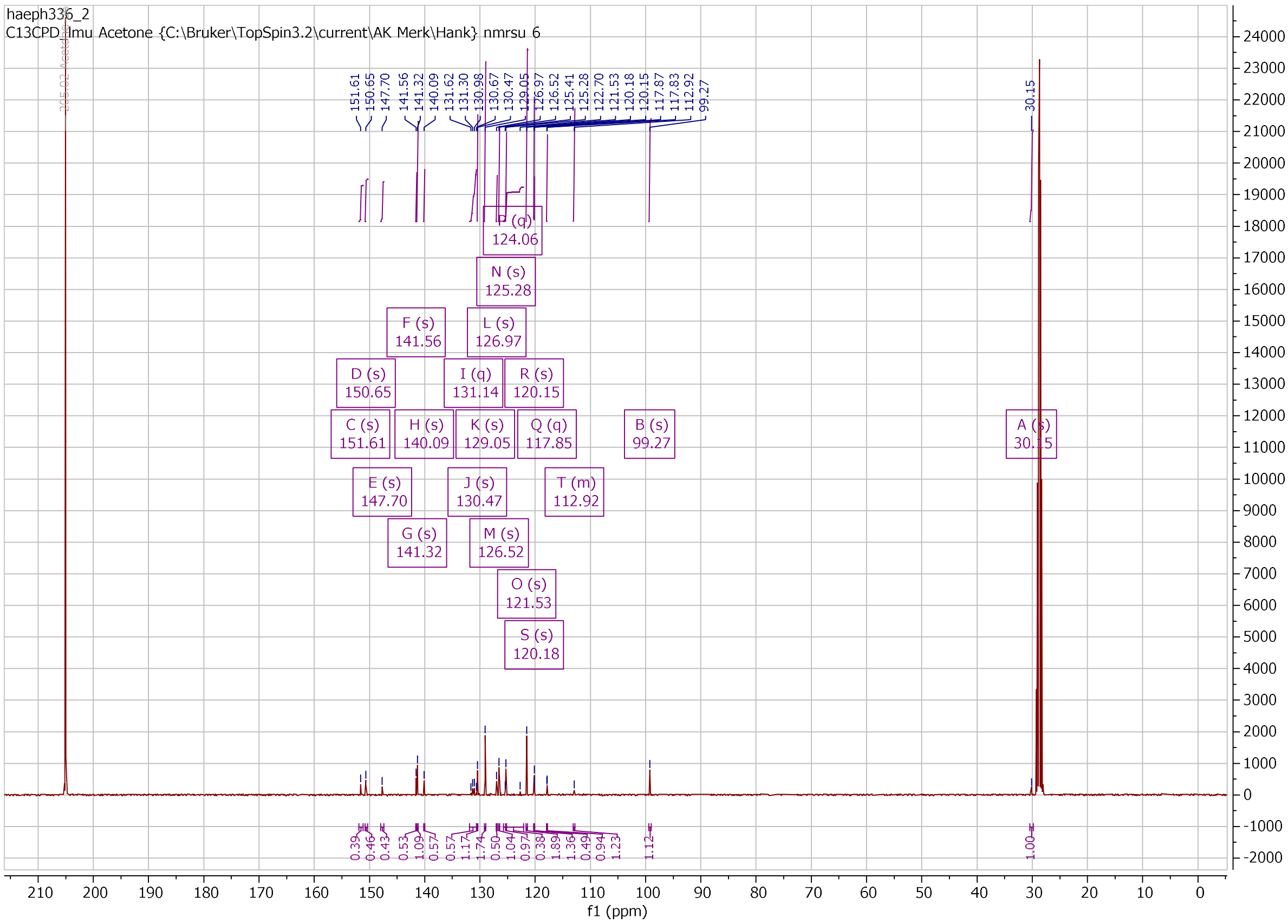
haeph336_2
C13CPD_lmu Acetone {C:\Bruker\TopSpin3.2\current\AK Merk\Hank} nmrsu 6
A (s) 30.15
B (s) 99.27
C (s) 151.61
D (s) 150.65
E (s) 147.70
F (s) 141.56
G (s) 141.32
H (s) 140.09
I (q) 131.14
J (s) 130.47
K (s) 129.05
L (s) 126.97
M (s) 126.52
N (s) 125.28
O (s) 121.53
P (q) 124.06
Q (q) 117.85
R (s) 120.15
S (s) 120.18
T (m) 112.92
f1 (ppm)

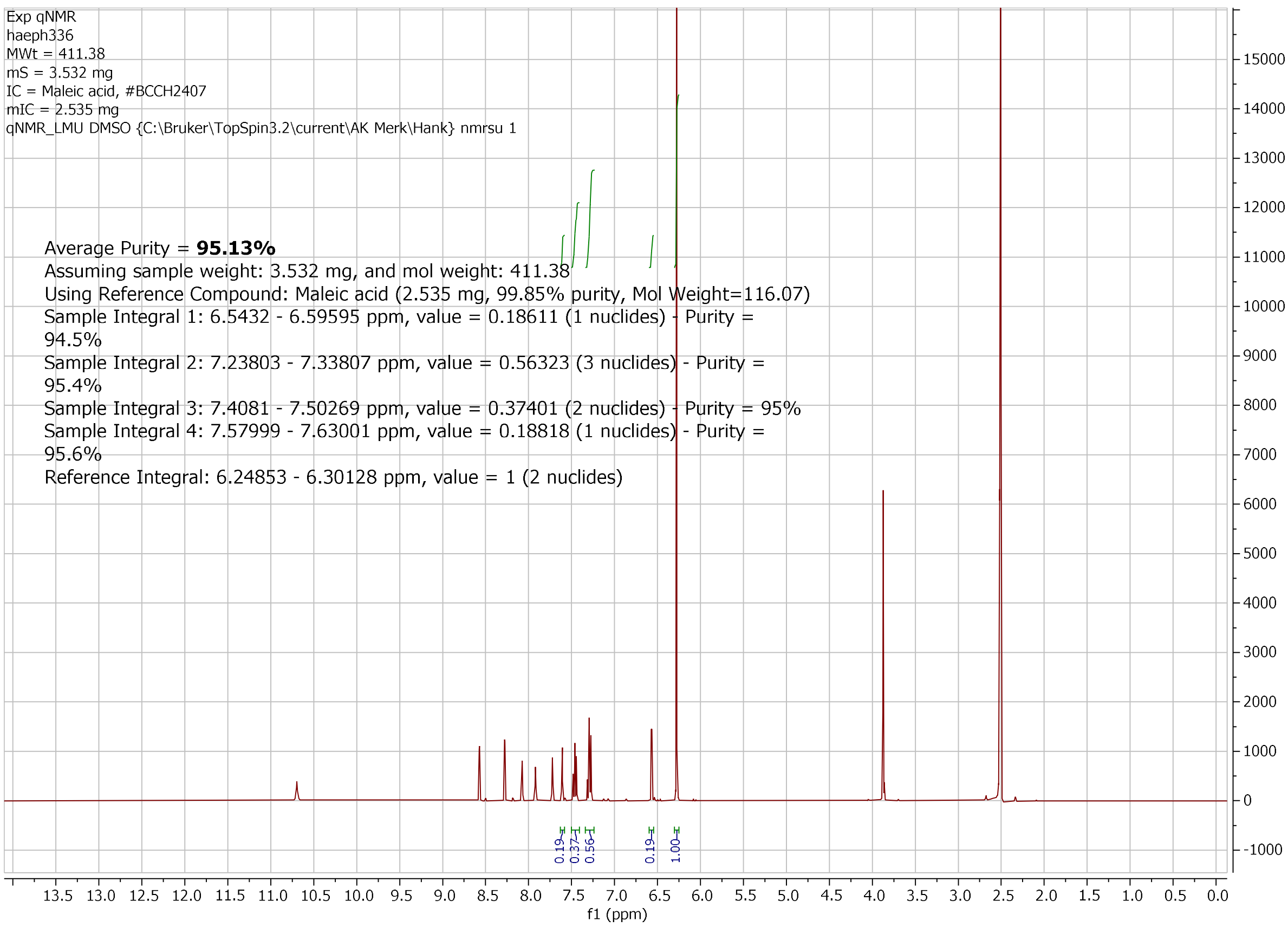
Exp qNMR
haeph336
MWt = 411.38
mS = 3.532 mg
IC = Maleic acid, #BCCH2407
mIC = 2.535 mg
qNMR_LMU DMSO {C:\Bruker\TopSpin3.2\current\AK Merk\Hank} nmrsu 1
Average Purity = 95.13%
Assuming sample weight: 3.532 mg, and mol weight: 411.38
Using Reference Compound: Maleic acid (2.535 mg, 99.85% purity, Mol Weight=116.07)
Sample Integral 1: 6.5432 - 6.59595 ppm, value = 0.18611 (1 nuclides) - Purity = 94.5%
Sample Integral 2: 7.23803 - 7.33807 ppm, value = 0.56323 (3 nuclides) - Purity = 95.4%
Sample Integral 3: 7.4081 - 7.50269 ppm, value = 0.37401 (2 nuclides) - Purity = 95%
Sample Integral 4: 7.57999 - 7.63001 ppm, value = 0.18818 (1 nuclides) - Purity = 95.6%
Reference Integral: 6.24853 - 6.30128 ppm, value = 1 (2 nuclides)
f1 (ppm)

Compound **30**:

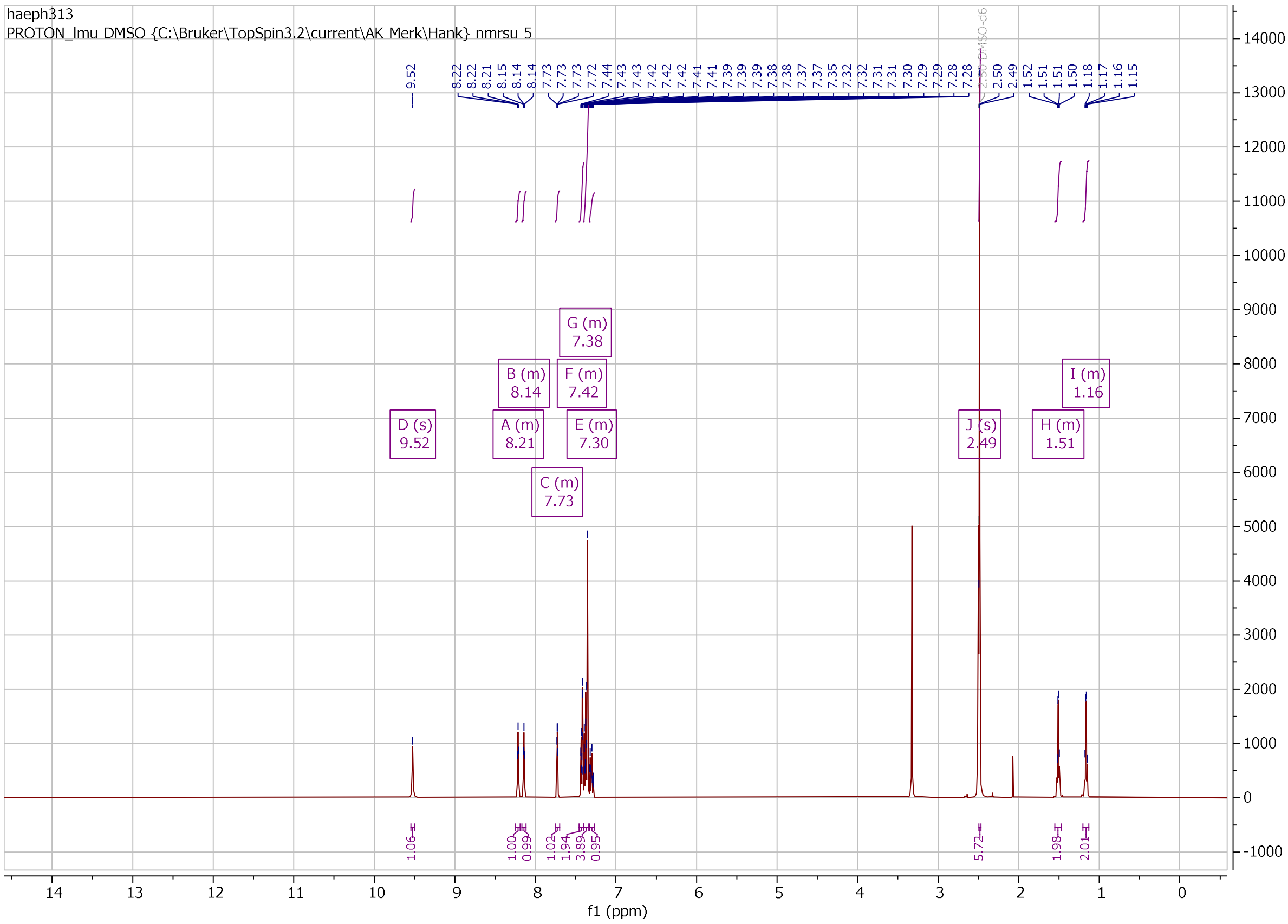
haeph313
PROTON_Imu DMSO {C:\Bruker\TopSpin3.2\current\AK Merk\Hank} nmrsu 5
D (s) 9.52
A (m) 8.21
B (m) 8.14
C (m) 7.73
F (m) 7.42
G (m) 7.38
E (m) 7.30
J (s) 2.49
H (m) 1.51
I (m) 1.16
f1 (ppm)

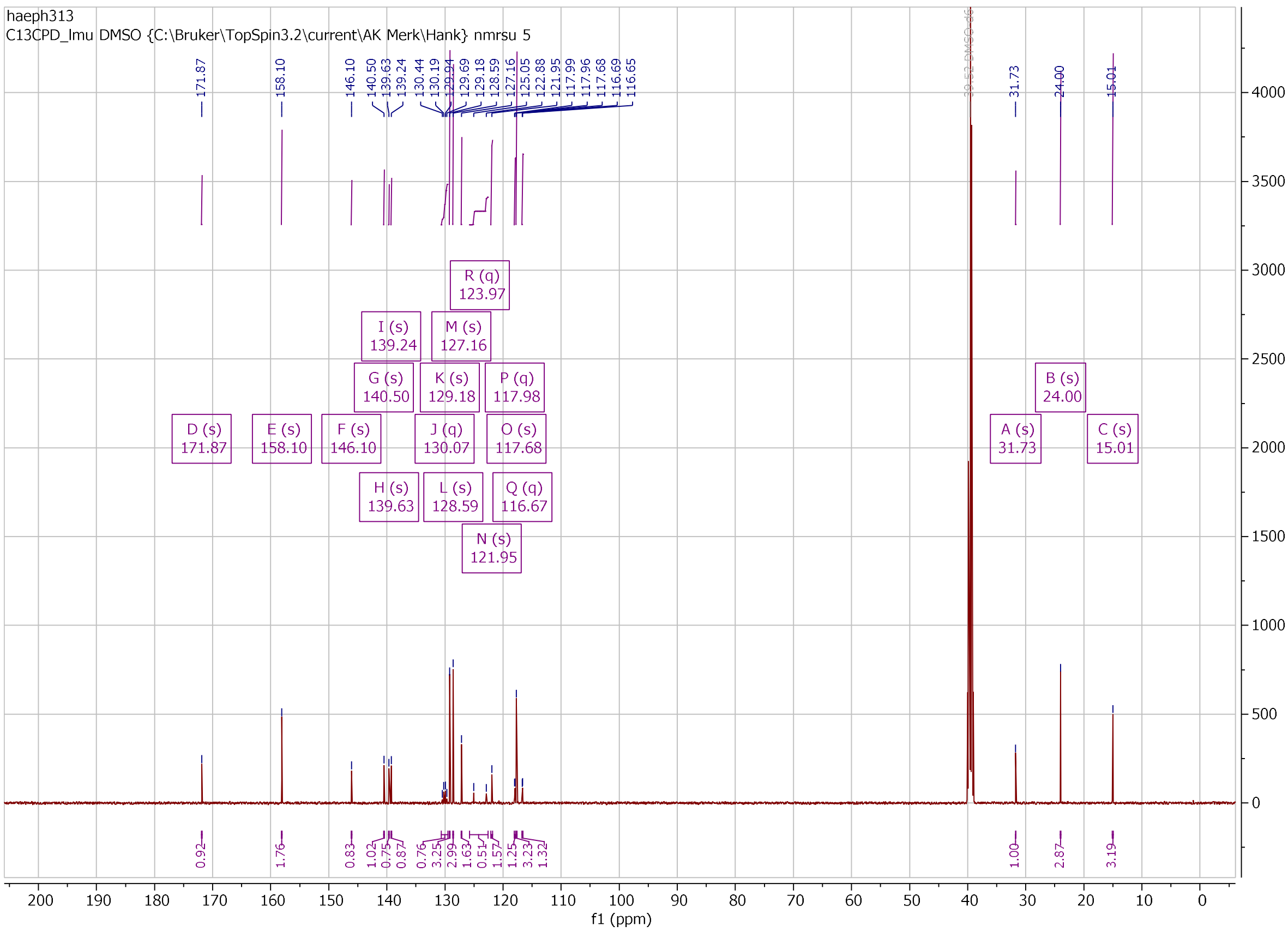
haeph313
C13CPD_Imu DMSO {C:\Bruker\TopSpin3.2\current\AK Merk\Hank} nmrsu 5
D (s) 171.87
E (s) 158.10
F (s) 146.10
G (s) 140.50
H (s) 139.63
I (s) 139.24
J (q) 130.07
K (s) 129.18
L (s) 128.59
M (s) 127.16
R (q) 123.97
N (s) 121.95
P (q) 117.98
O (s) 117.68
Q (q) 116.67
A (s) 31.73
B (s) 24.00
C (s) 15.01
f1 (ppm)

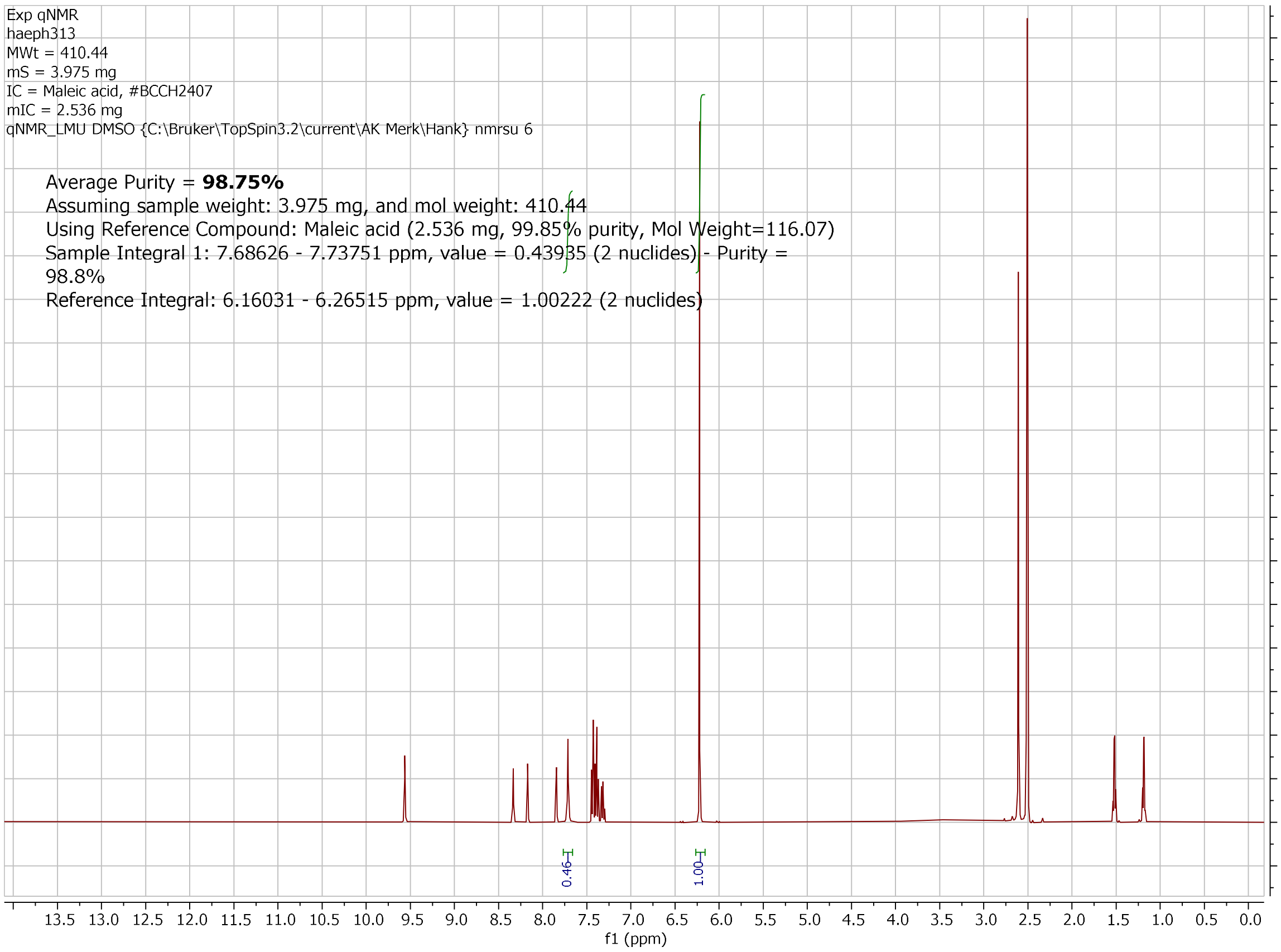
Exp qNMR
haeph313
MWt = 410.44
mS = 3.975 mg
IC = Maleic acid, #BCCH2407
mIC = 2.536 mg
qNMR_LMU DMSO {C:\Bruker\TopSpin3.2\current\AK Merk\Hank} nmrsu 6
Average Purity = **98.75%**
Assuming sample weight: 3.975 mg, and mol weight: 410.44
Using Reference Compound: Maleic acid (2.536 mg, 99.85% purity, Mol Weight=116.07)
Sample Integral 1: 7.68626 - 7.73751 ppm, value = 0.43935 (2 nuclides) - Purity = 98.8%
Reference Integral: 6.16031 - 6.26515 ppm, value = 1.00222 (2 nuclides)
0.46
1.00
f1 (ppm)

Compound **31**:

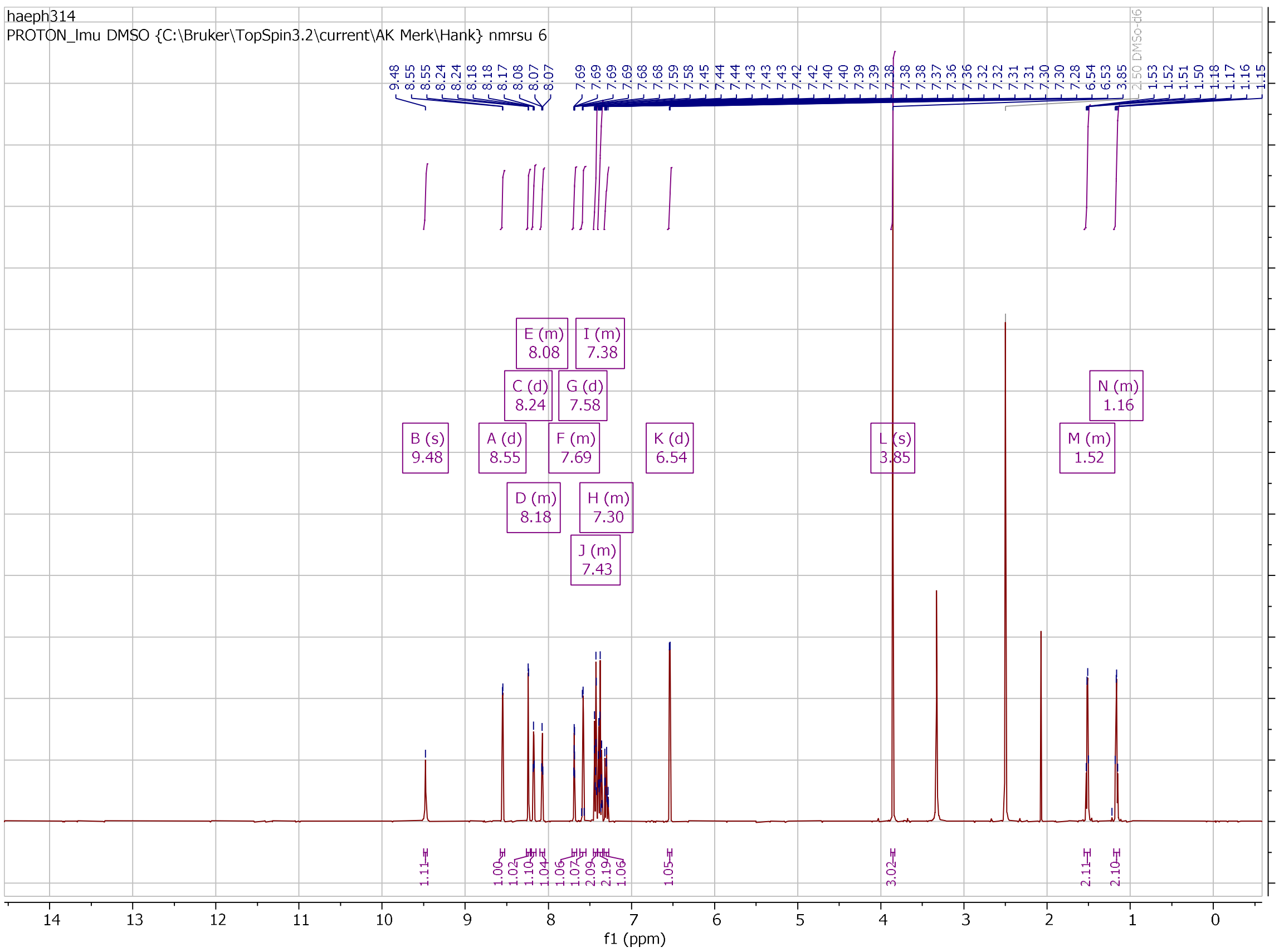
haeph314
PROTON_lmu DMSO {C:\Bruker\TopSpin3.2\current\AK Merk\Hank} nmrsu 6
E (m) 8.08
I (m) 7.38
C (d) 8.24
G (d) 7.58
N (m) 1.16
B (s) 9.48
A (d) 8.55
F (m) 7.69
K (d) 6.54
L (s) 3.85
M (m) 1.52
D (m) 8.18
H (m) 7.30
J (m) 7.43
1.11
1.00
1.02
1.10
1.04
1.06
1.07
2.09
2.19
1.06
1.05
3.02
2.11
2.10
f1 (ppm)

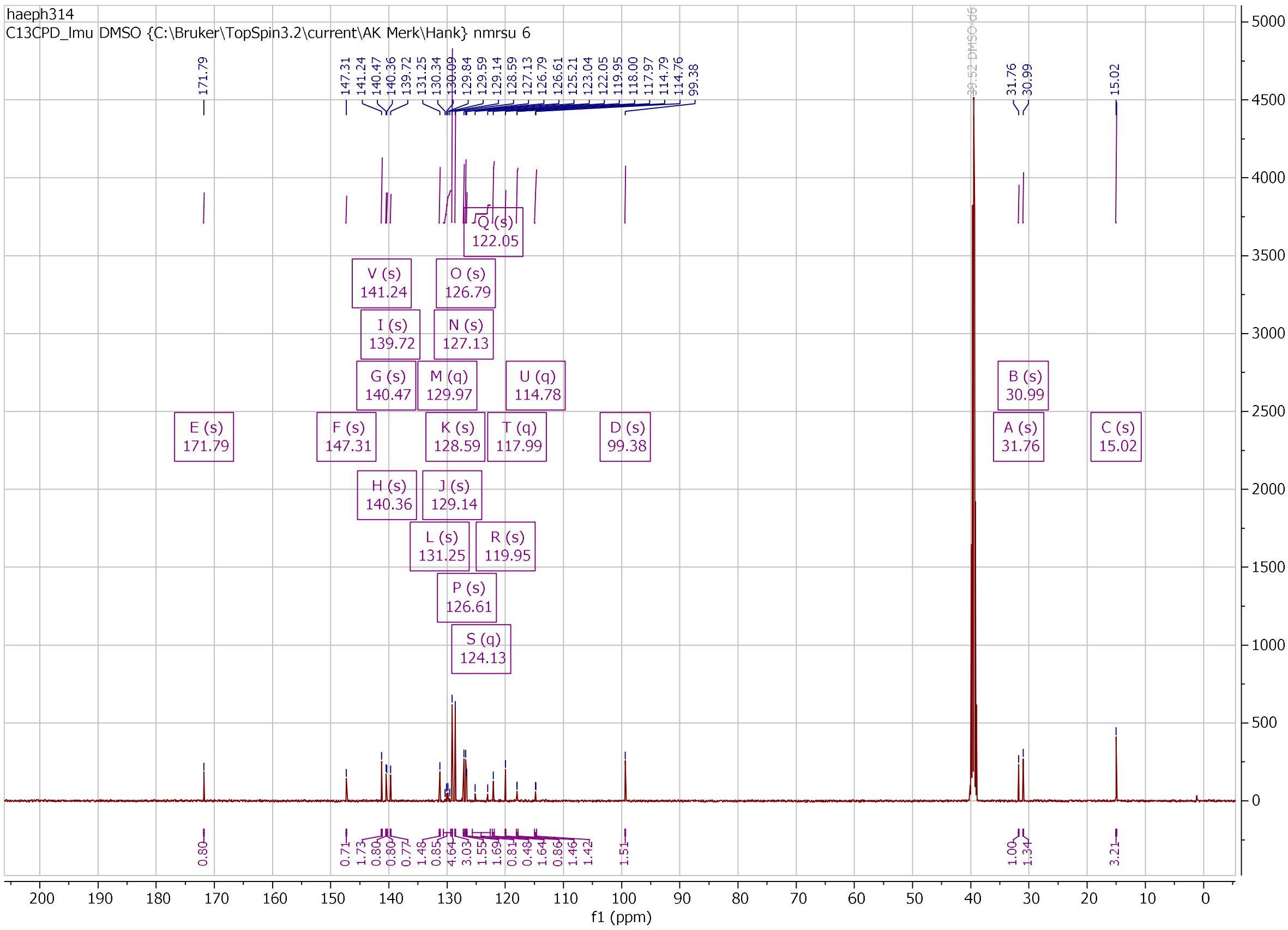
haeph314
C13CPD_lmu DMSO {C:\Bruker\TopSpin3.2\current\AK Merk\Hank} nmrsu 6
E (s) 171.79
F (s) 147.31
V (s) 141.24
G (s) 140.47
H (s) 140.36
I (s) 139.72
L (s) 131.25
M (q) 129.97
J (s) 129.14
K (s) 128.59
N (s) 127.13
O (s) 126.79
P (s) 126.61
S (q) 124.13
Q (s) 122.05
R (s) 119.95
T (q) 117.99
U (q) 114.78
D (s) 99.38
A (s) 31.76
B (s) 30.99
C (s) 15.02
f1 (ppm)

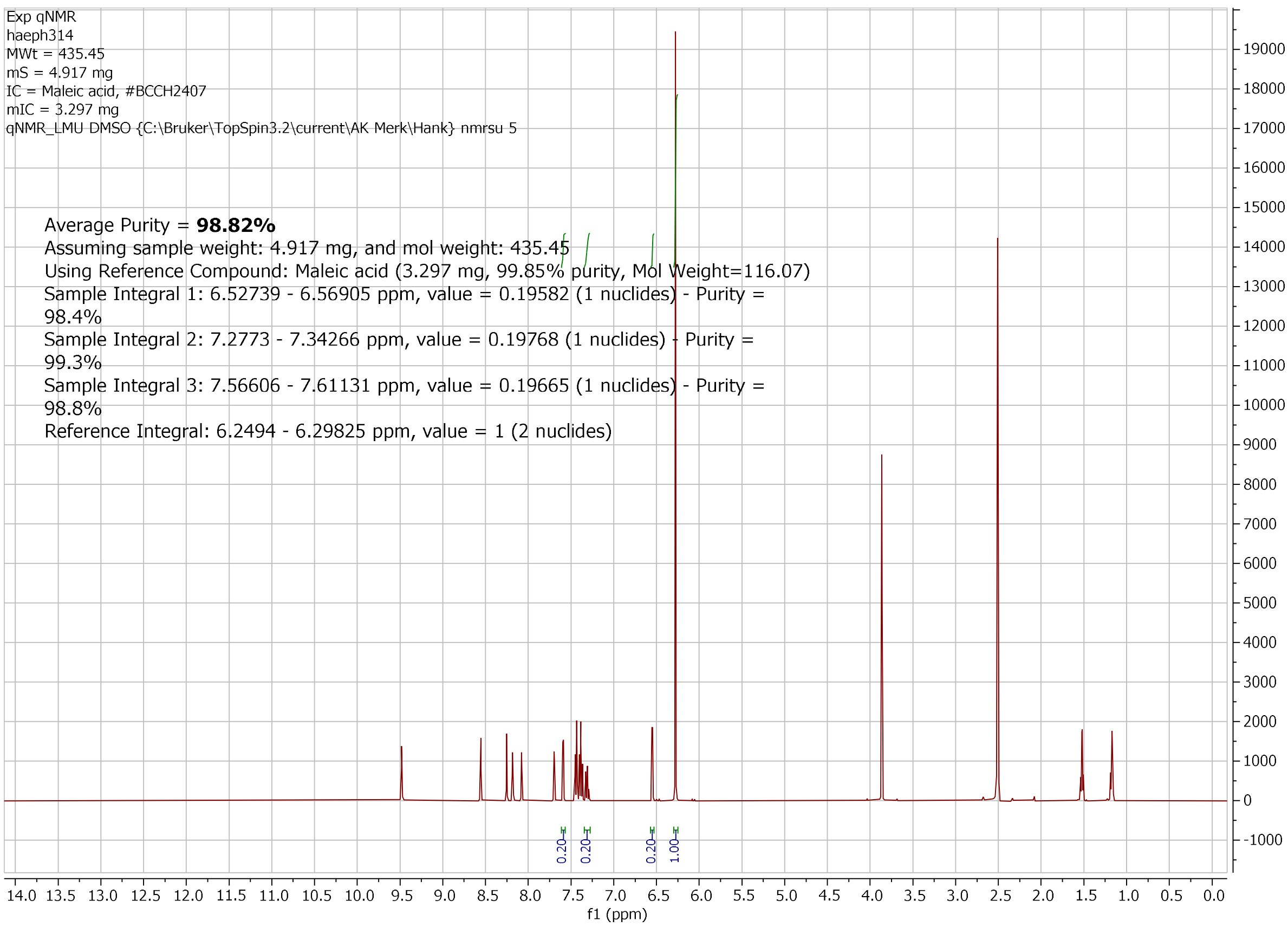
Exp qNMR
haeph314
MWt = 435.45
mS = 4.917 mg
IC = Maleic acid, #BCCH2407
mIC = 3.297 mg
qNMR_LMU DMSO {C:\Bruker\TopSpin3.2\current\AK Merk\Hank} nmrsu 5
Average Purity = 98.82%
Assuming sample weight: 4.917 mg, and mol weight: 435.45
Using Reference Compound: Maleic acid (3.297 mg, 99.85% purity, Mol Weight=116.07)
Sample Integral 1: 6.52739 - 6.56905 ppm, value = 0.19582 (1 nuclides) - Purity = 98.4%
Sample Integral 2: 7.2773 - 7.34266 ppm, value = 0.19768 (1 nuclides) - Purity = 99.3%
Sample Integral 3: 7.56606 - 7.61131 ppm, value = 0.19665 (1 nuclides) - Purity = 98.8%
Reference Integral: 6.2494 - 6.29825 ppm, value = 1 (2 nuclides)
f1 (ppm)

Compound **32**:

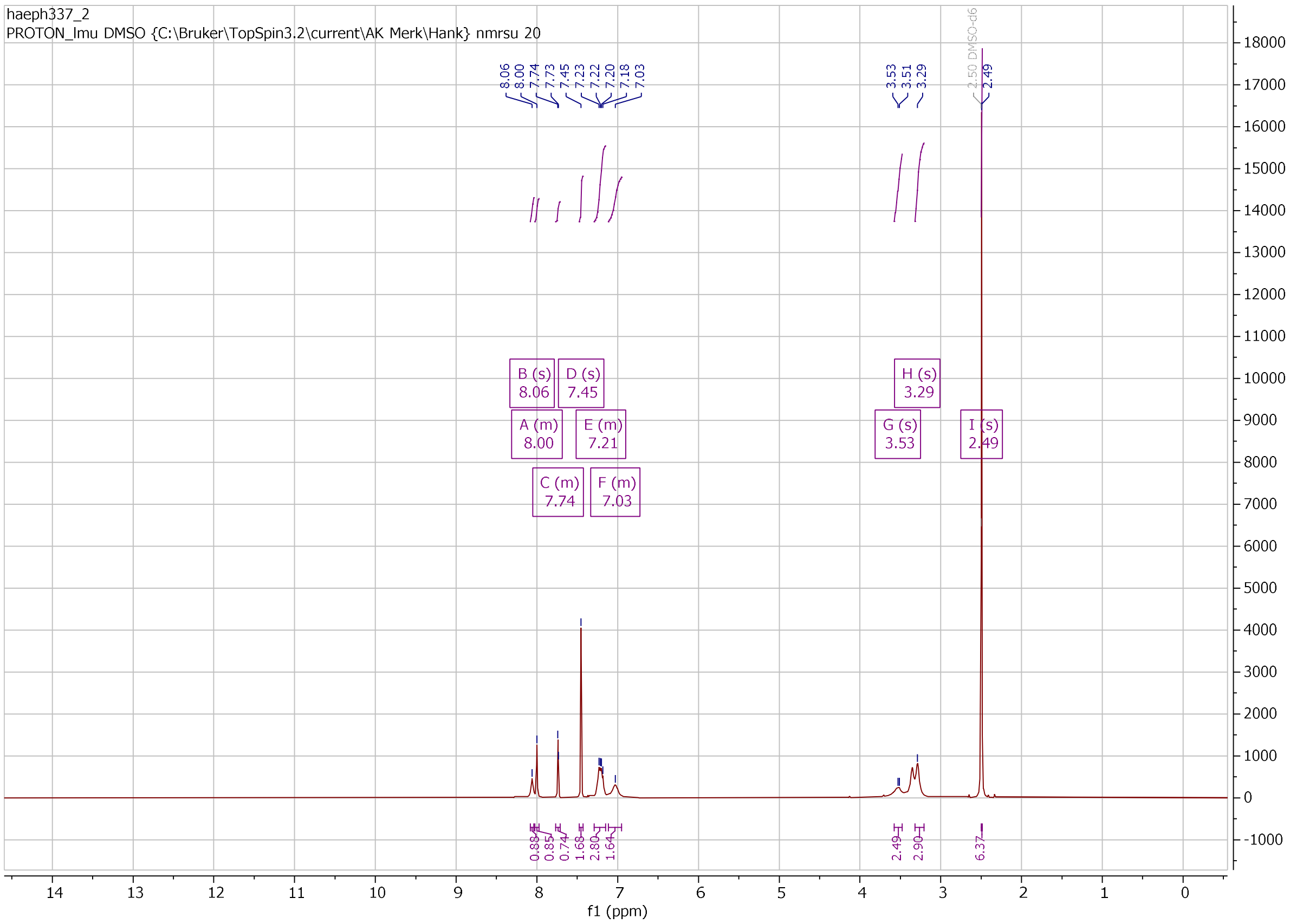
haeph337_2
PROTON_Imu DMSO {C:\Bruker\TopSpin3.2\current\AK Merk\Hank} nmrsu 20
B (s) 8.06
D (s) 7.45
A (m) 8.00
E (m) 7.21
C (m) 7.74
F (m) 7.03
H (s) 3.29
G (s) 3.53
I (s) 2.49
f1 (ppm)

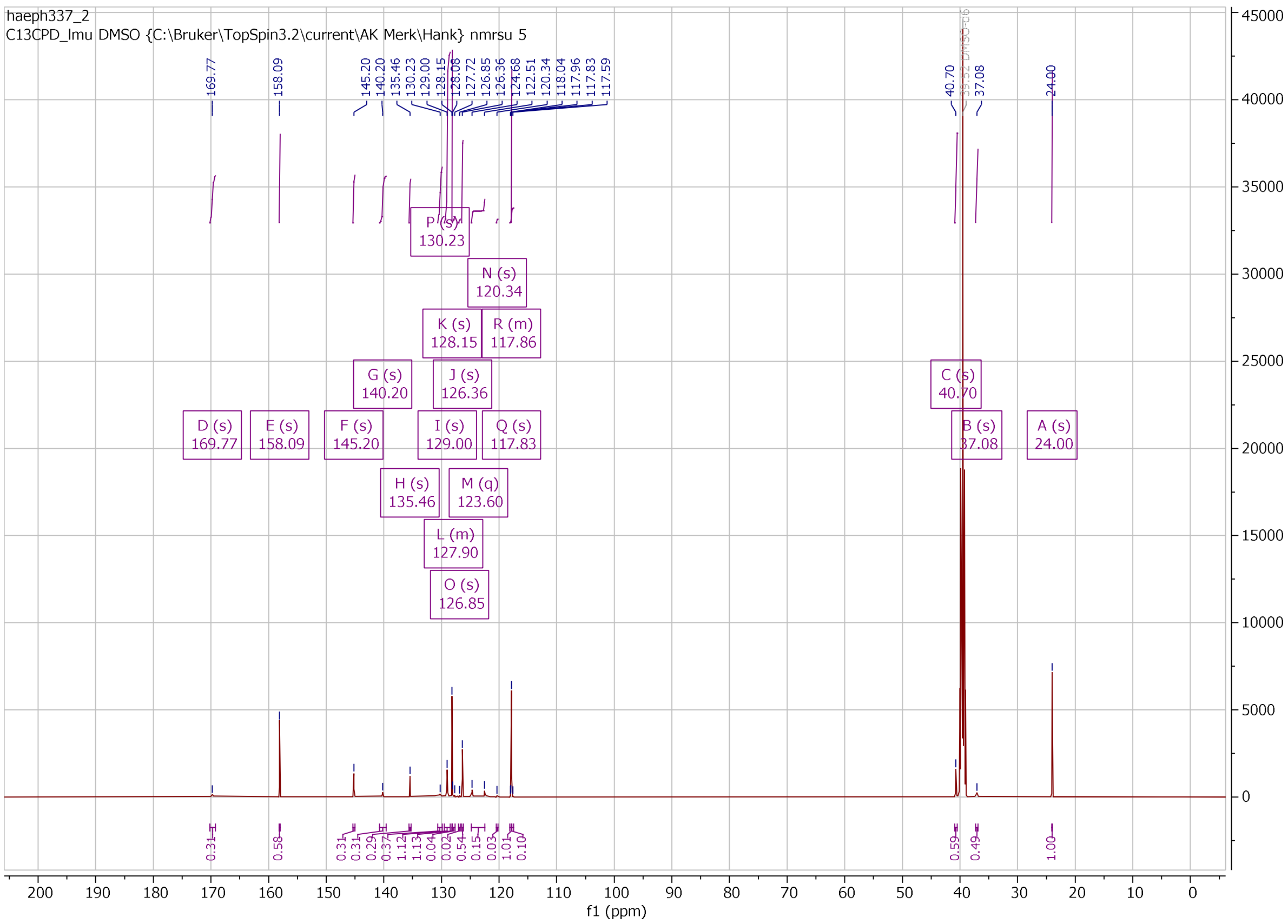
haeph337_2
C13CPD_Imu DMSO {C:\Bruker\TopSpin3.2\current\AK Merk\Hank} nmrsu 5
P (s) 130.23
N (s) 120.34
K (s) 128.15
R (m) 117.86
G (s) 140.20
J (s) 126.36
D (s) 169.77
E (s) 158.09
F (s) 145.20
I (s) 129.00
Q (s) 117.83
H (s) 135.46
M (q) 123.60
L (m) 127.90
O (s) 126.85
C (s) 40.70
B (s) 37.08
A (s) 24.00
f1 (ppm)

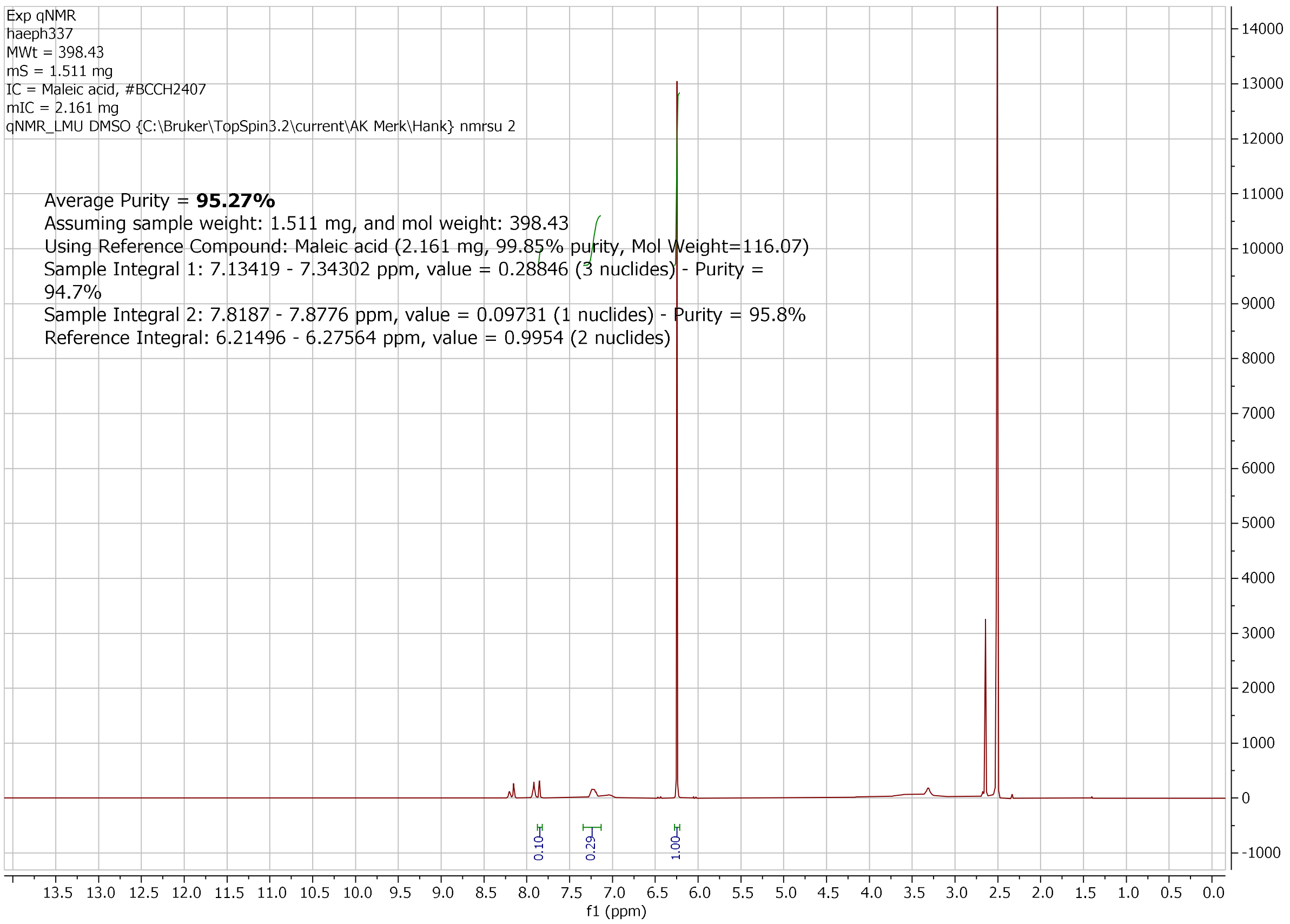
Exp qNMR
haeph337
MWt = 398.43
mS = 1.511 mg
IC = Maleic acid, #BCCH2407
mIC = 2.161 mg
qNMR_LMU DMSO {C:\Bruker\TopSpin3.2\current\AK Merk\Hank} nmrsu 2
Average Purity = **95.27%**
Assuming sample weight: 1.511 mg, and mol weight: 398.43
Using Reference Compound: Maleic acid (2.161 mg, 99.85% purity, Mol Weight=116.07)
Sample Integral 1: 7.13419 - 7.34302 ppm, value = 0.28846 (3 nuclides) - Purity = 94.7%
Sample Integral 2: 7.8187 - 7.8776 ppm, value = 0.09731 (1 nuclides) - Purity = 95.8%
Reference Integral: 6.21496 - 6.27564 ppm, value = 0.9954 (2 nuclides)
f1 (ppm)

Compound **33**:

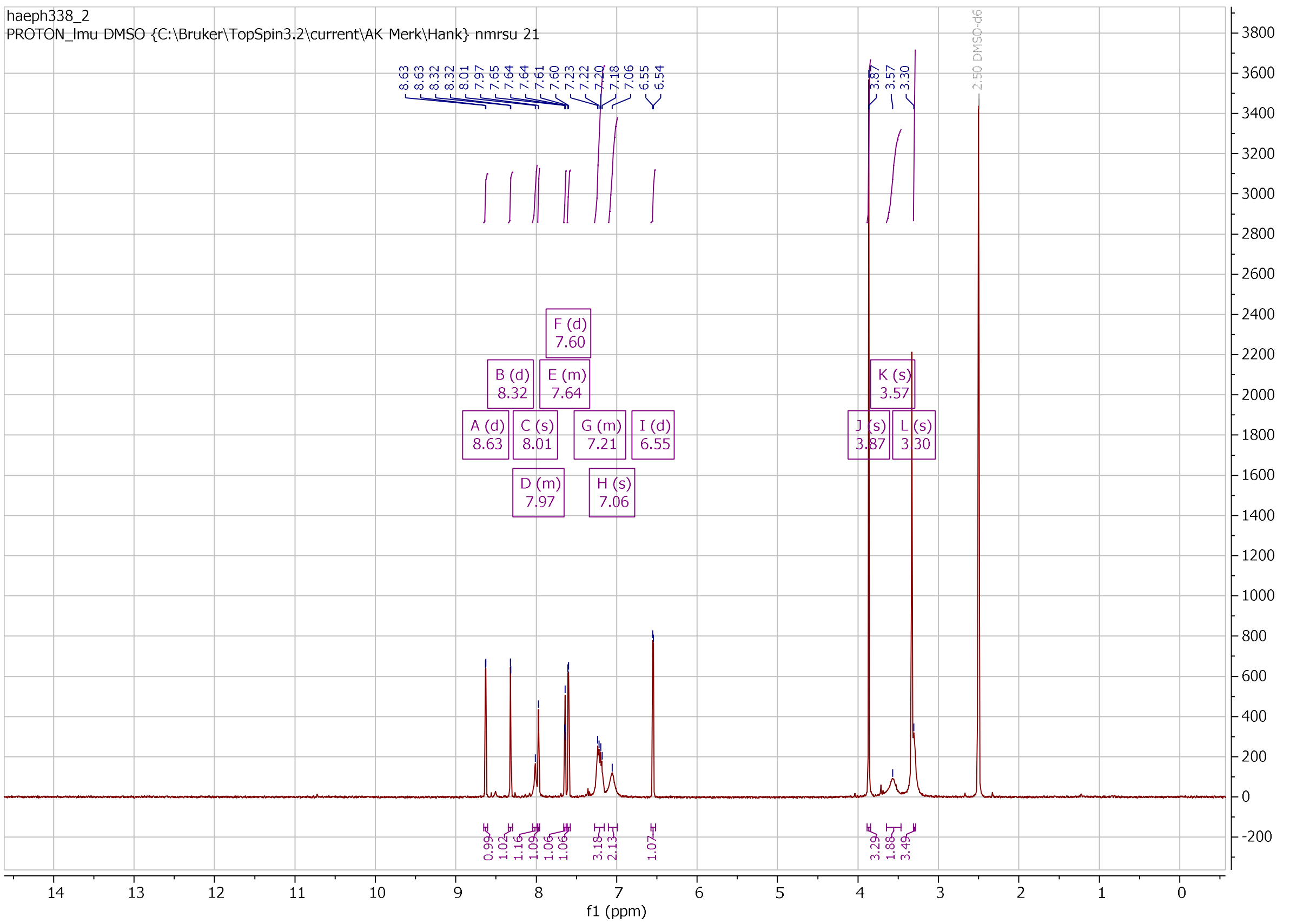
haeph338_2
PROTON_lmu DMSO {C:\Bruker\TopSpin3.2\current\AK Merk\Hank} nmrsu 21
2.50 DMSO-d6
A (d) 8.63
B (d) 8.32
C (s) 8.01
D (m) 7.97
E (m) 7.64
F (d) 7.60
G (m) 7.21
H (s) 7.06
I (d) 6.55
J (s) 3.87
K (s) 3.57
L (s) 3.30
f1 (ppm)

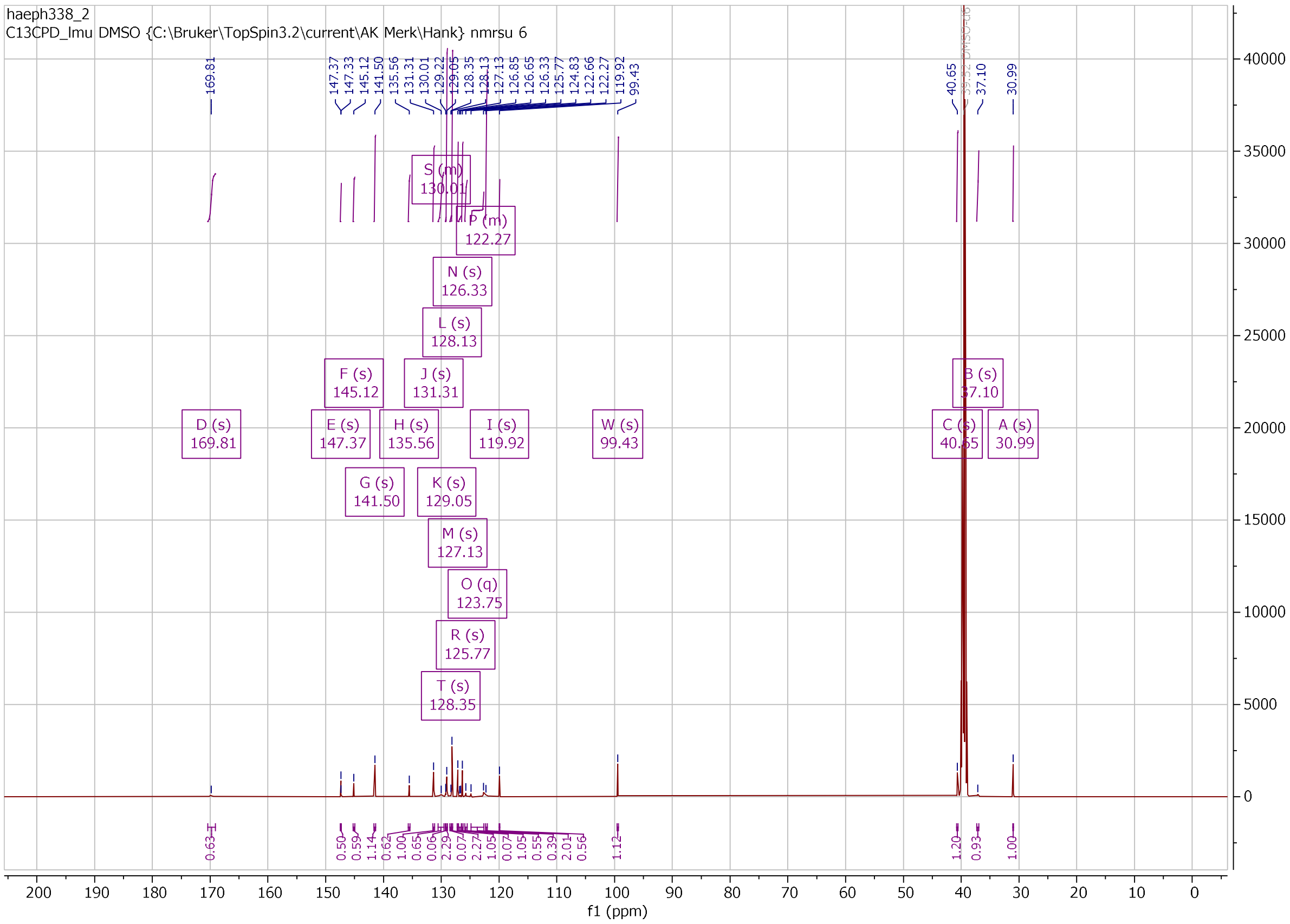
haeph338_2
C13CPD_lmu DMSO {C:\Bruker\TopSpin3.2\current\AK Merk\Hank} nmrsu 6
f1 (ppm)

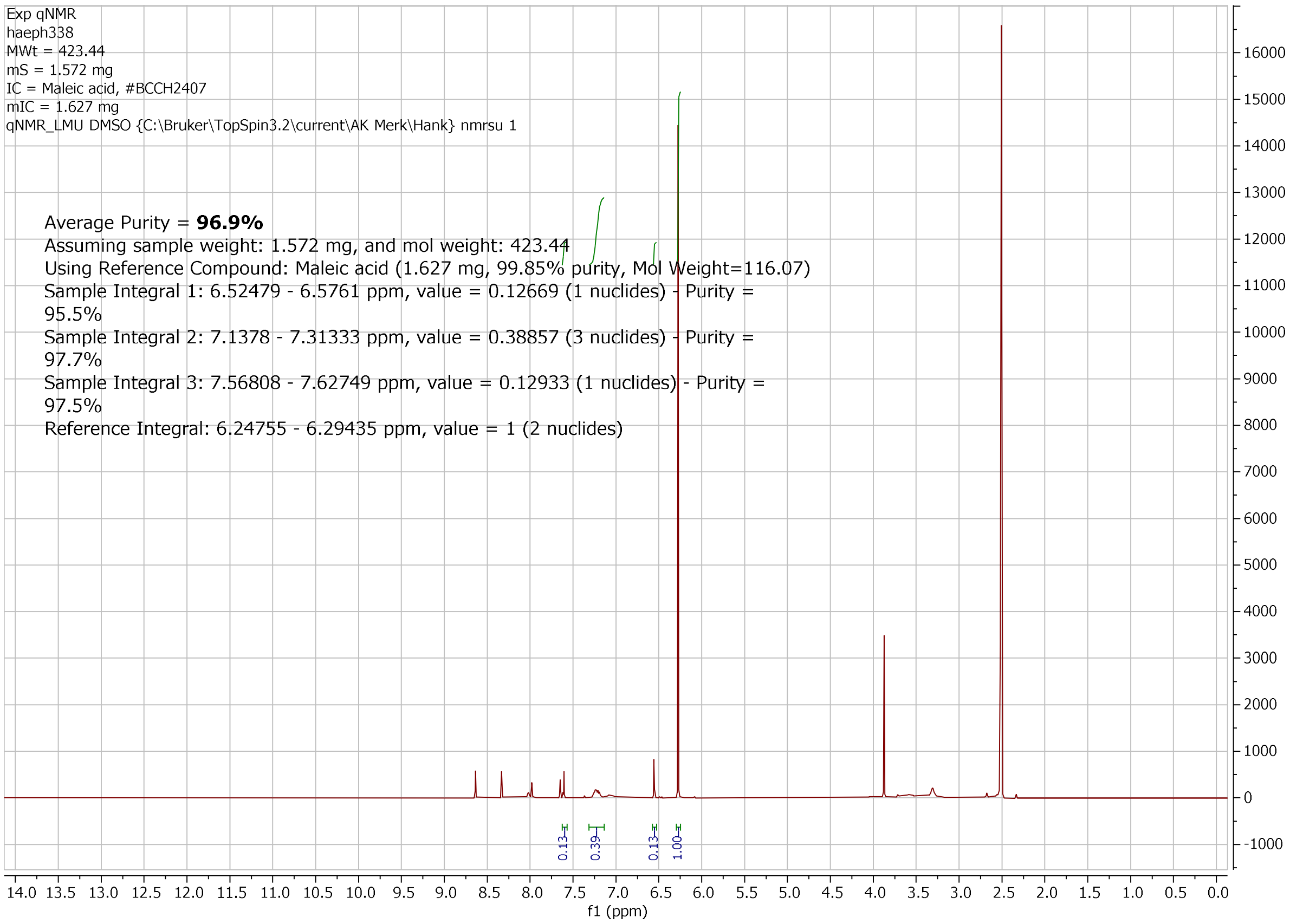
Exp qNMR
haeph338
MWt = 423.44
mS = 1.572 mg
IC = Maleic acid, #BCCH2407
mIC = 1.627 mg
qNMR_LMU DMSO {C:\Bruker\TopSpin3.2\current\AK Merk\Hank} nmrsu 1
Average Purity = **96.9%**
Assuming sample weight: 1.572 mg, and mol weight: 423.44
Using Reference Compound: Maleic acid (1.627 mg, 99.85% purity, Mol Weight=116.07)
Sample Integral 1: 6.52479 - 6.5761 ppm, value = 0.12669 (1 nuclides) - Purity = 95.5%
Sample Integral 2: 7.1378 - 7.31333 ppm, value = 0.38857 (3 nuclides) - Purity = 97.7%
Sample Integral 3: 7.56808 - 7.62749 ppm, value = 0.12933 (1 nuclides) - Purity = 97.5%
Reference Integral: 6.24755 - 6.29435 ppm, value = 1 (2 nuclides)
f1 (ppm)

Compound **34**:

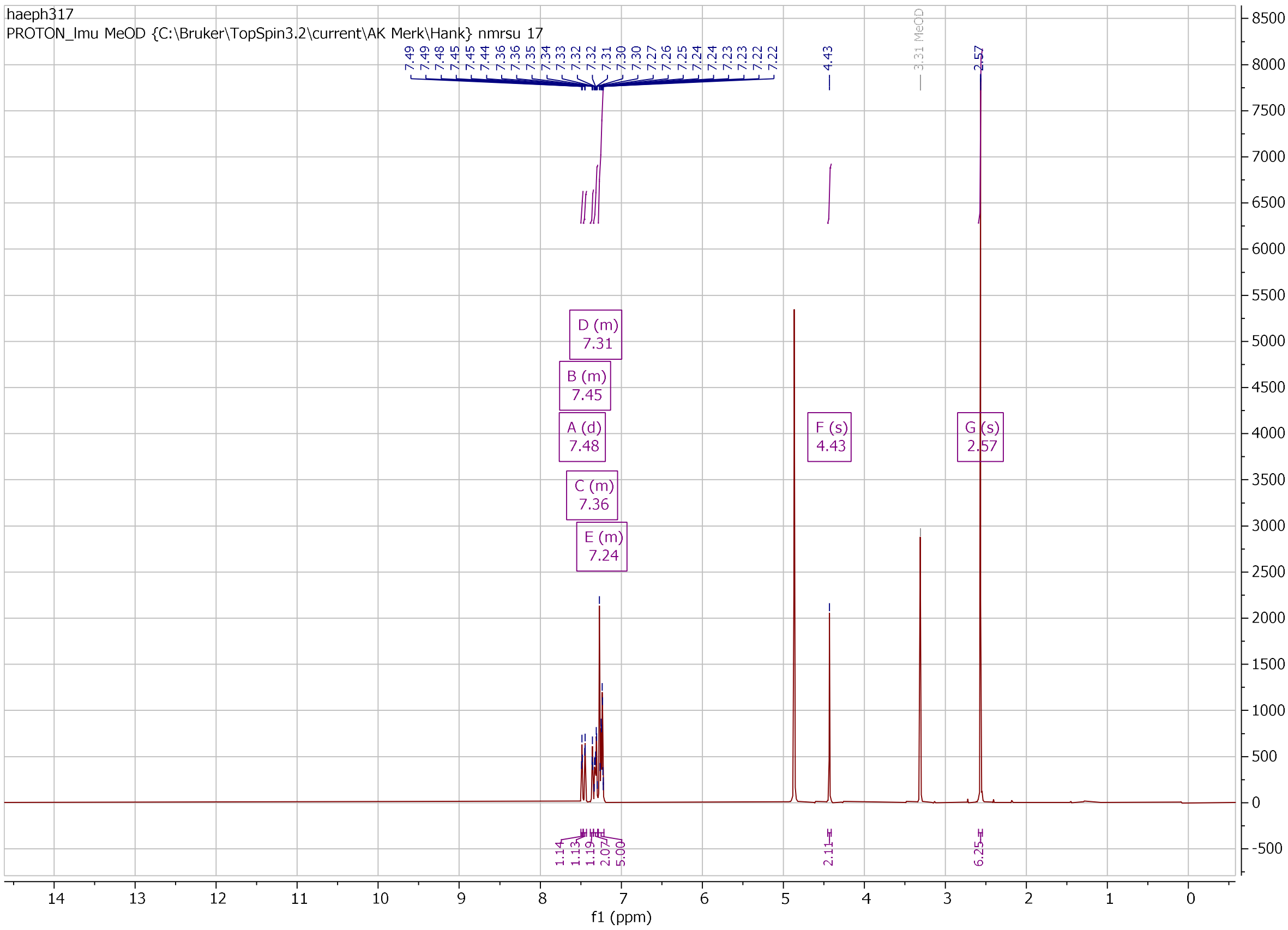
haeph317
PROTON_Imu MeOD {C:\Bruker\TopSpin3.2\current\AK Merk\Hank} nmrsu 17
D (m) 7.31
B (m) 7.45
A (d) 7.48
C (m) 7.36
E (m) 7.24
F (s) 4.43
G (s) 2.57
f1 (ppm)

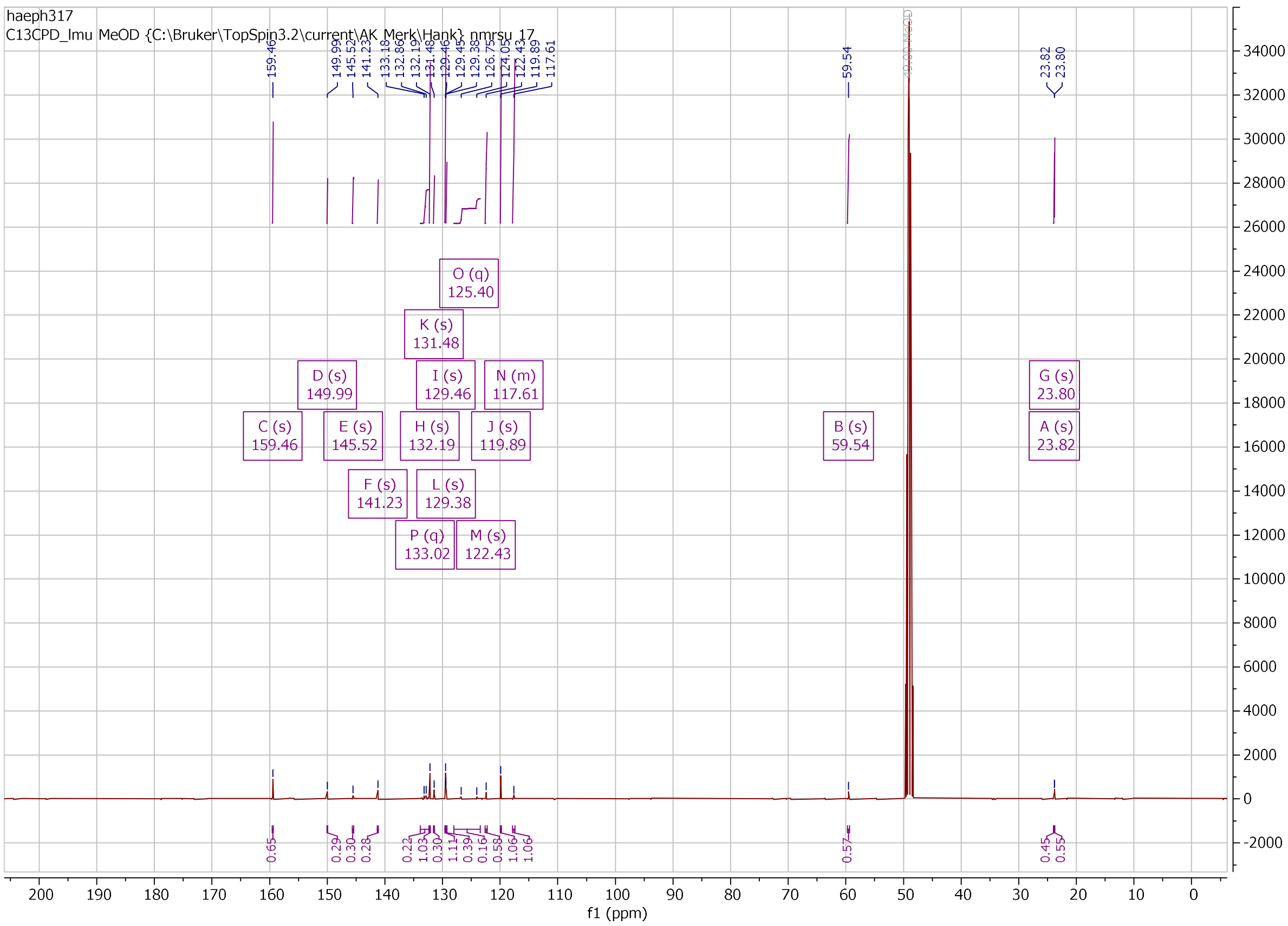
haeph317
C13CPD_Imu MeOD {C:\Bruker\TopSpin3.2\current\AK Merk\Hank} nmrsu 17
O (q) 125.40
K (s) 131.48
D (s) 149.99
I (s) 129.46
N (m) 117.61
C (s) 159.46
E (s) 145.52
H (s) 132.19
J (s) 119.89
B (s) 59.54
G (s) 23.80
A (s) 23.82
F (s) 141.23
L (s) 129.38
P (q) 133.02
M (s) 122.43
f1 (ppm)

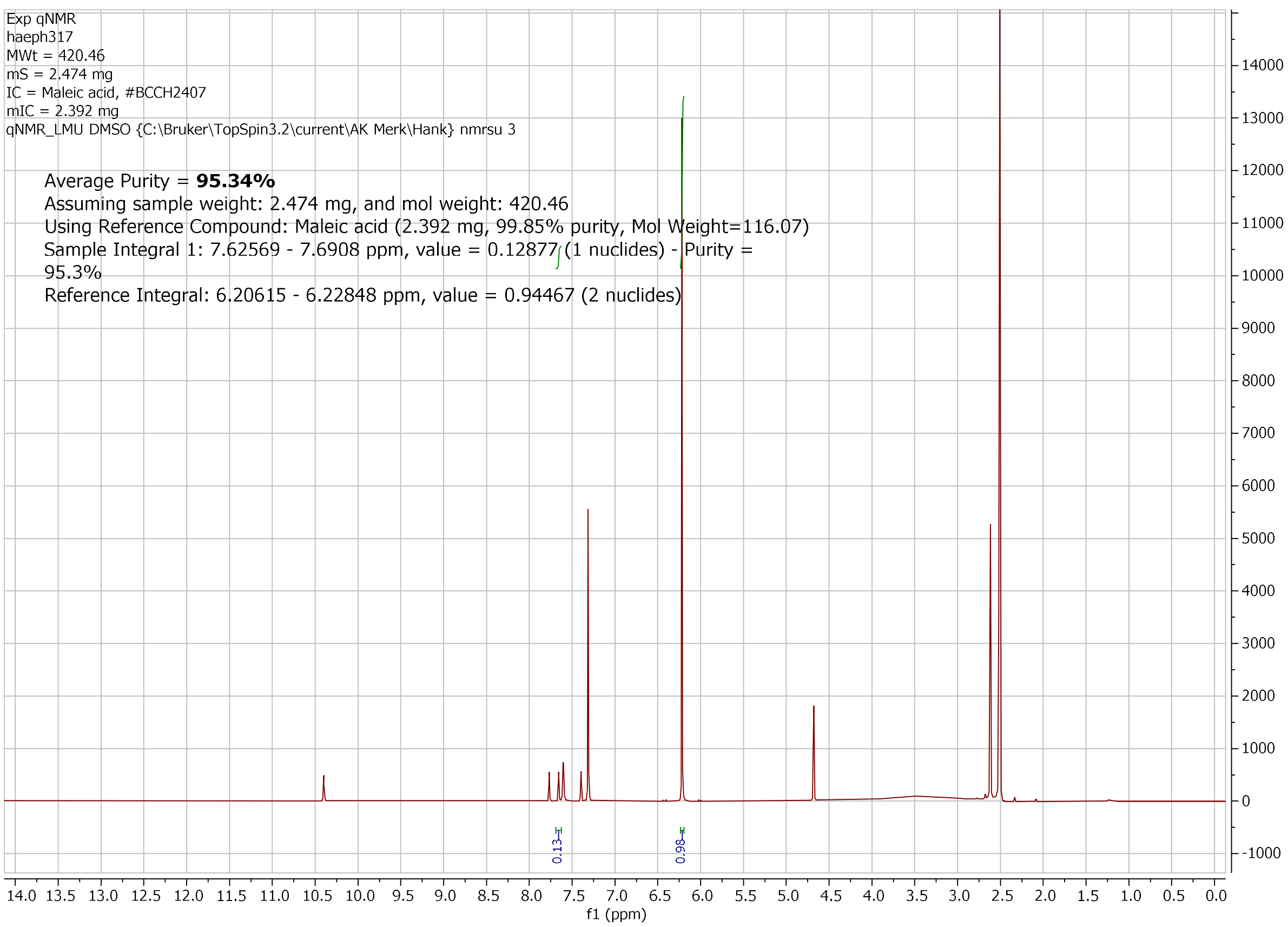
Exp qNMR
haeph317
MWt = 420.46
mS = 2.474 mg
IC = Maleic acid, #BCCH2407
mIC = 2.392 mg
qNMR_LMU DMSO {C:\Bruker\TopSpin3.2\current\AK Merk\Hank} nmrsu 3
Average Purity = 95.34%
Assuming sample weight: 2.474 mg, and mol weight: 420.46
Using Reference Compound: Maleic acid (2.392 mg, 99.85% purity, Mol Weight=116.07)
Sample Integral 1: 7.62569 - 7.6908 ppm, value = 0.12877 (1 nuclides) - Purity = 95.3%
Reference Integral: 6.20615 - 6.22848 ppm, value = 0.94467 (2 nuclides)
f1 (ppm)

Compound **35**:

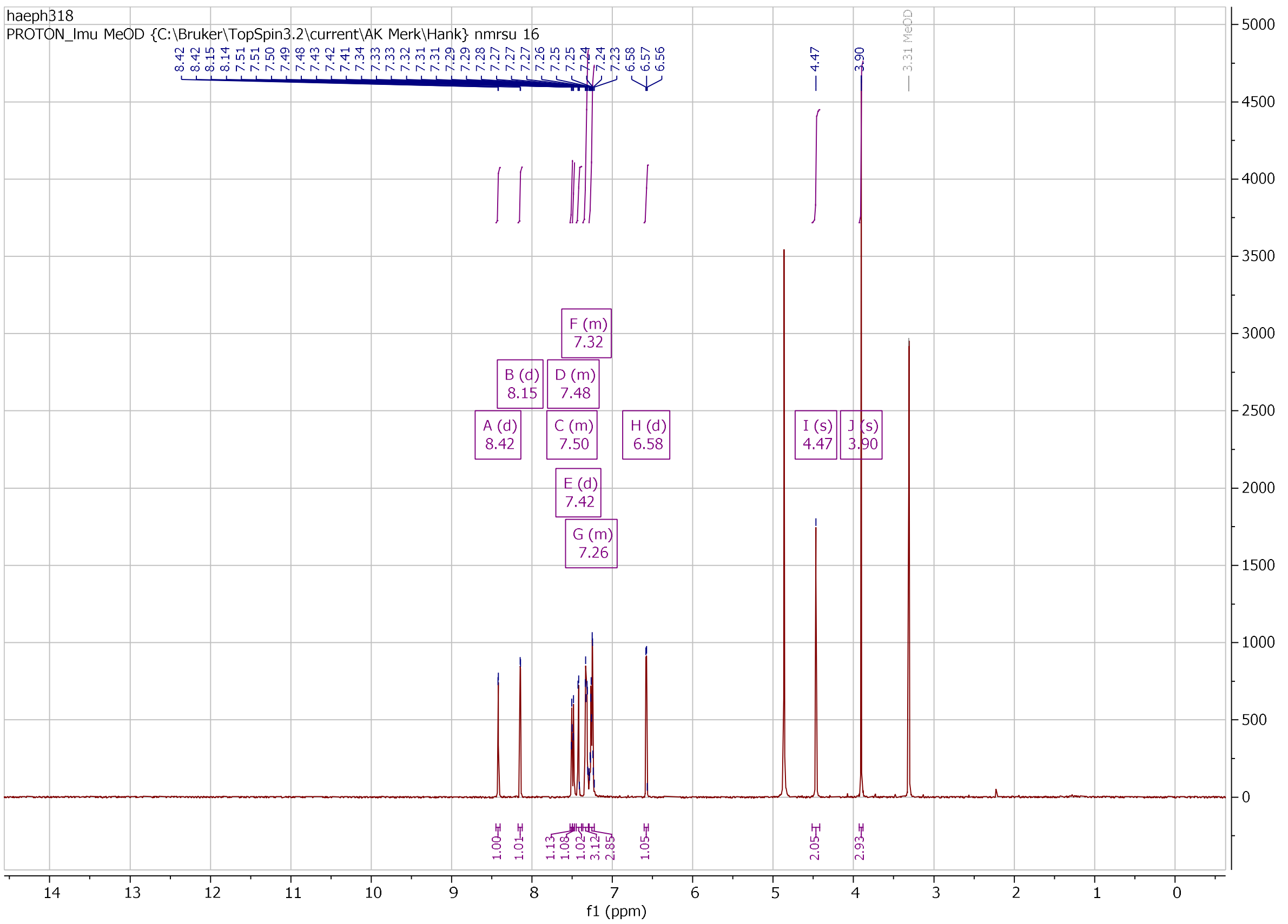
haeph318
PROTON_lmu MeOD {C:\Bruker\TopSpin3.2\current\AK Merk\Hank} nmrsu 16
3.31 MeOD
F (m) 7.32
B (d) 8.15
D (m) 7.48
A (d) 8.42
C (m) 7.50
H (d) 6.58
I (s) 4.47
J (s) 3.90
E (d) 7.42
G (m) 7.26
f1 (ppm)

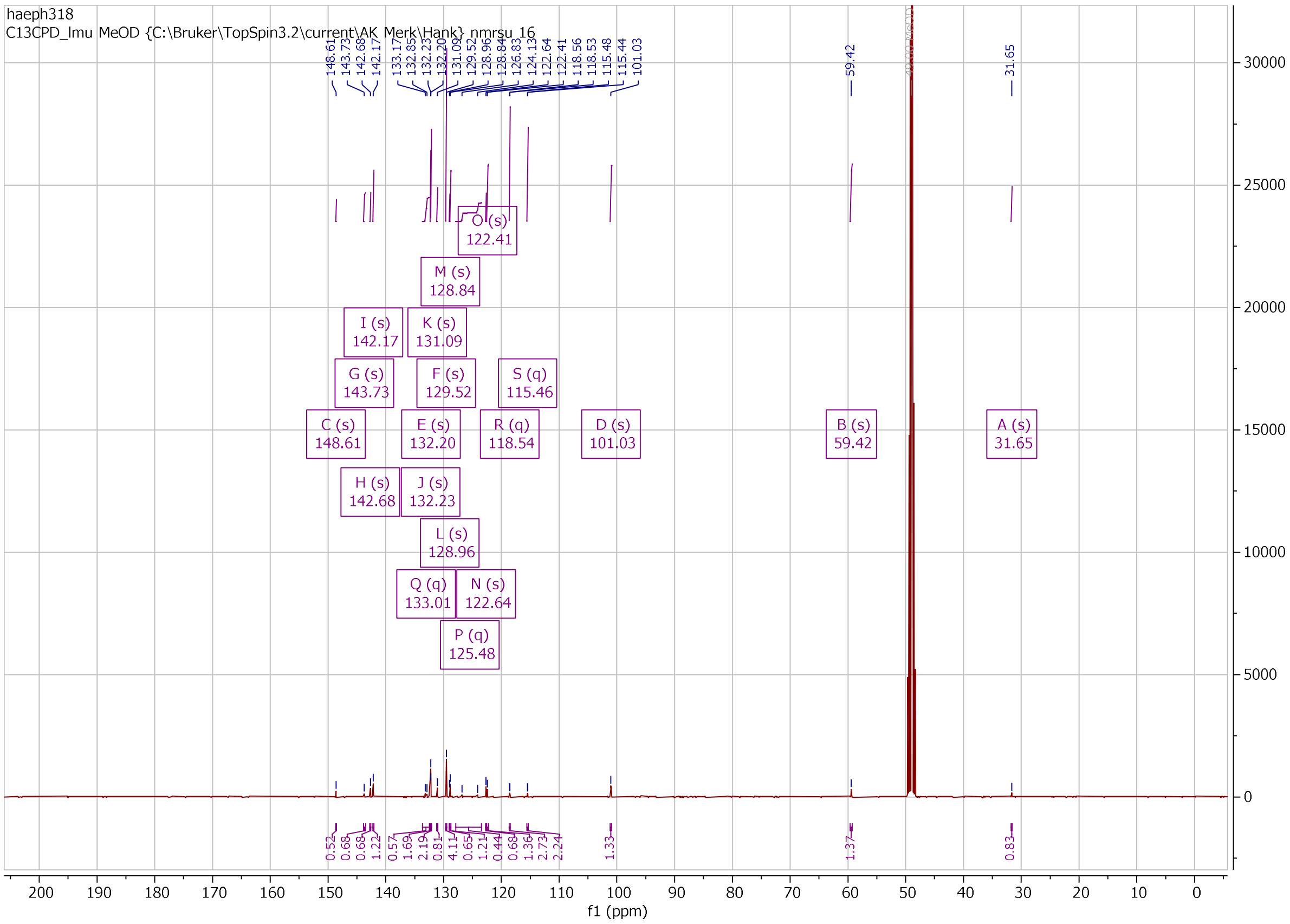
haeph318
C13CPD_lmu MeOD {C:\Bruker\TopSpin3.2\current\AK Merk\Hank} nmrsu 16
f1 (ppm)

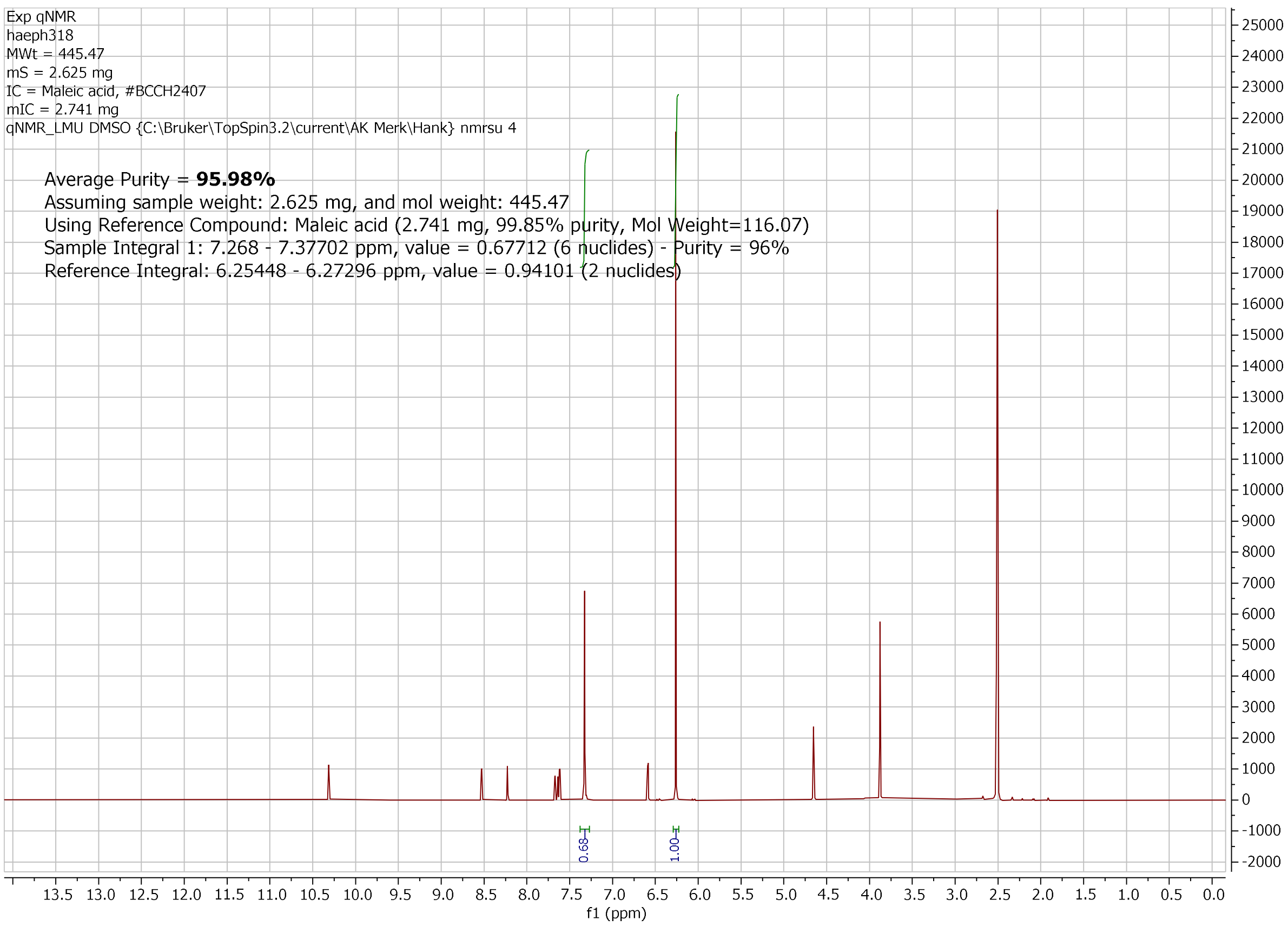
Exp qNMR
haeph318
MWt = 445.47
mS = 2.625 mg
IC = Maleic acid, #BCCH2407
mIC = 2.741 mg
qNMR_LMU DMSO {C:\Bruker\TopSpin3.2\current\AK Merk\Hank} nmrsu 4
Average Purity = 95.98%
Assuming sample weight: 2.625 mg, and mol weight: 445.47
Using Reference Compound: Maleic acid (2.741 mg, 99.85% purity, Mol Weight=116.07)
Sample Integral 1: 7.268 - 7.37702 ppm, value = 0.67712 (6 nuclides) - Purity = 96%
Reference Integral: 6.25448 - 6.27296 ppm, value = 0.94101 (2 nuclides)
f1 (ppm)

Compound **36**:

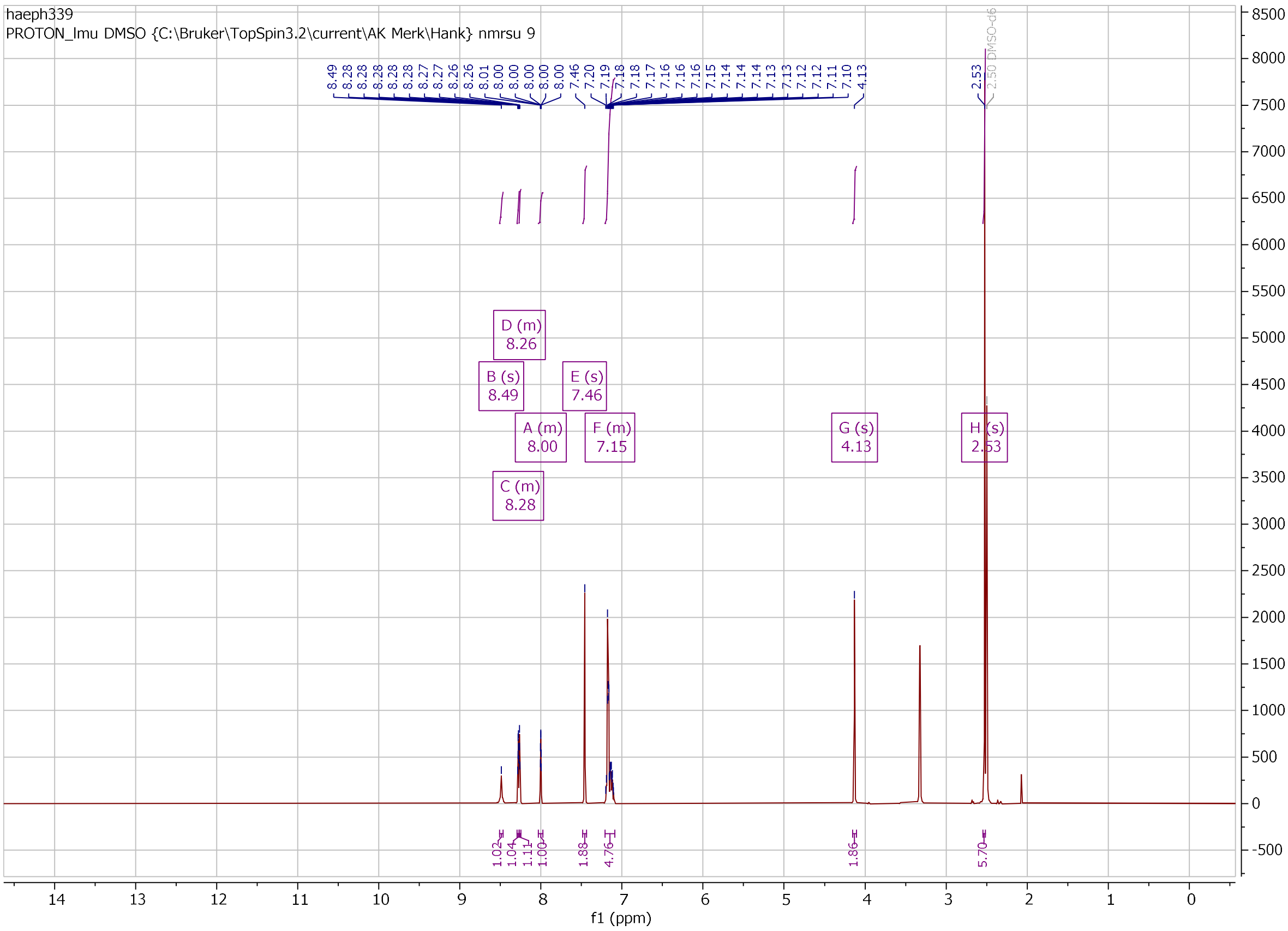
haeph339
PROTON_Imu DMSO {C:\Bruker\TopSpin3.2\current\AK Merk\Hank} nmrsu 9
D (m) 8.26
B (s) 8.49
E (s) 7.46
A (m) 8.00
F (m) 7.15
G (s) 4.13
H (s) 2.53
C (m) 8.28
f1 (ppm)

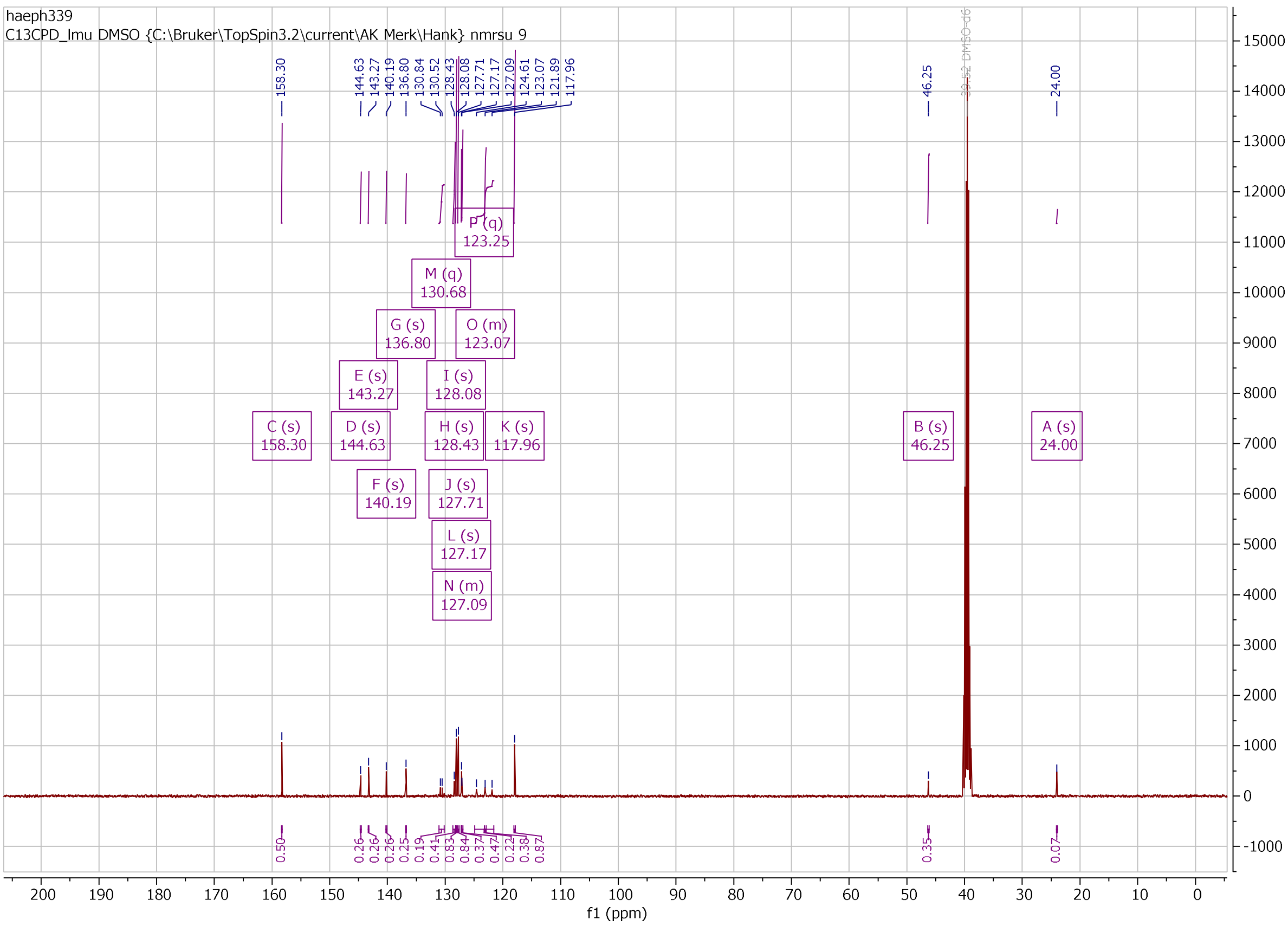
haeph339
C13CPD_Imu DMSO {C:\Bruker\TopSpin3.2\current\AK Merk\Hank} nmrsu 9
P (q) 123.25
M (q) 130.68
G (s) 136.80
O (m) 123.07
E (s) 143.27
I (s) 128.08
C (s) 158.30
D (s) 144.63
H (s) 128.43
K (s) 117.96
B (s) 46.25
A (s) 24.00
F (s) 140.19
J (s) 127.71
L (s) 127.17
N (m) 127.09
f1 (ppm)

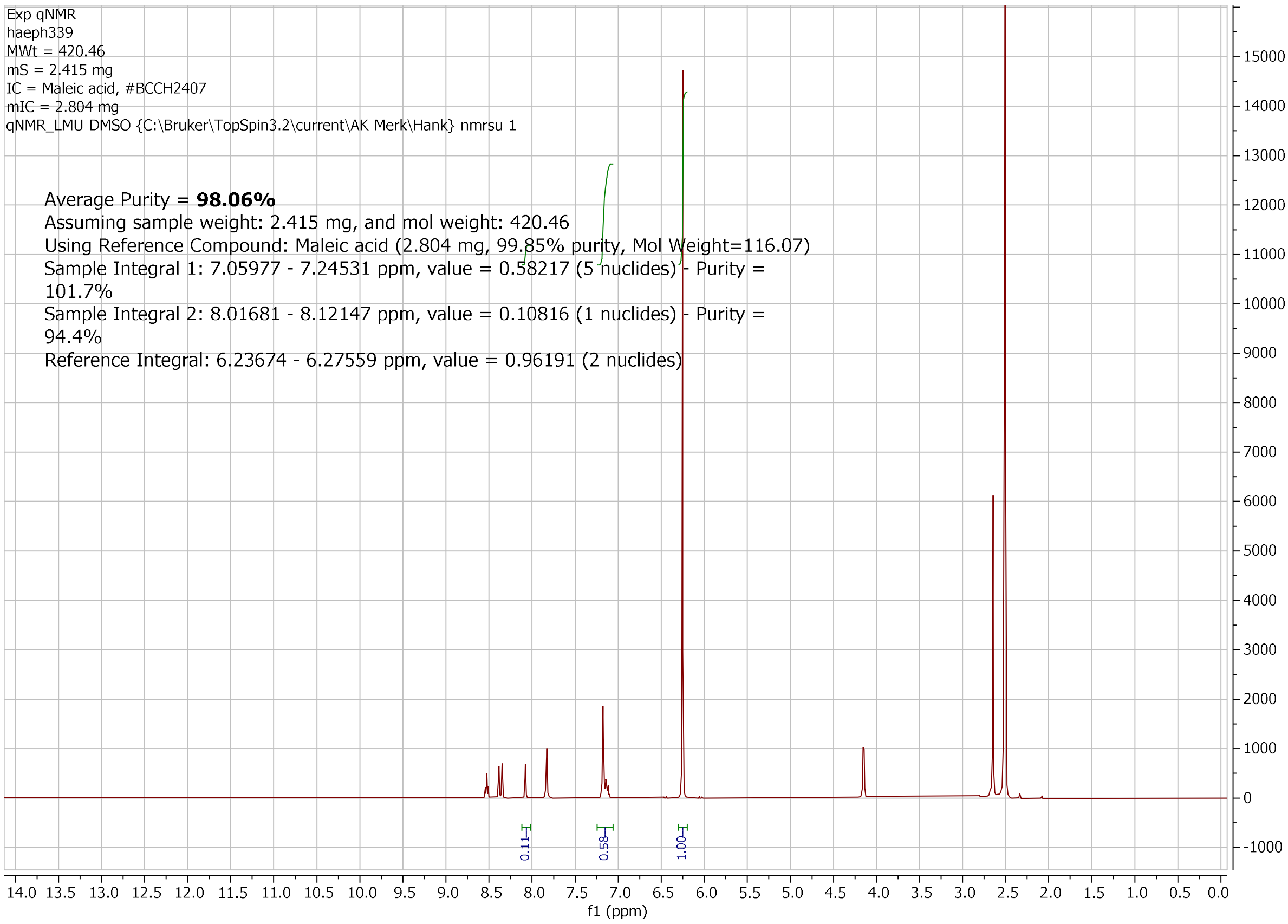
Exp qNMR
haeph339
MWt = 420.46
mS = 2.415 mg
IC = Maleic acid, #BCCH2407
mIC = 2.804 mg
qNMR_LMU DMSO {C:\Bruker\TopSpin3.2\current\AK Merk\Hank} nmrsu 1
Average Purity = **98.06%**
Assuming sample weight: 2.415 mg, and mol weight: 420.46
Using Reference Compound: Maleic acid (2.804 mg, 99.85% purity, Mol Weight=116.07)
Sample Integral 1: 7.05977 - 7.24531 ppm, value = 0.58217 (5 nuclides) - Purity = 101.7%
Sample Integral 2: 8.01681 - 8.12147 ppm, value = 0.10816 (1 nuclides) - Purity = 94.4%
Reference Integral: 6.23674 - 6.27559 ppm, value = 0.96191 (2 nuclides)
0.11
0.58
1.00
f1 (ppm)

Compound **37**:

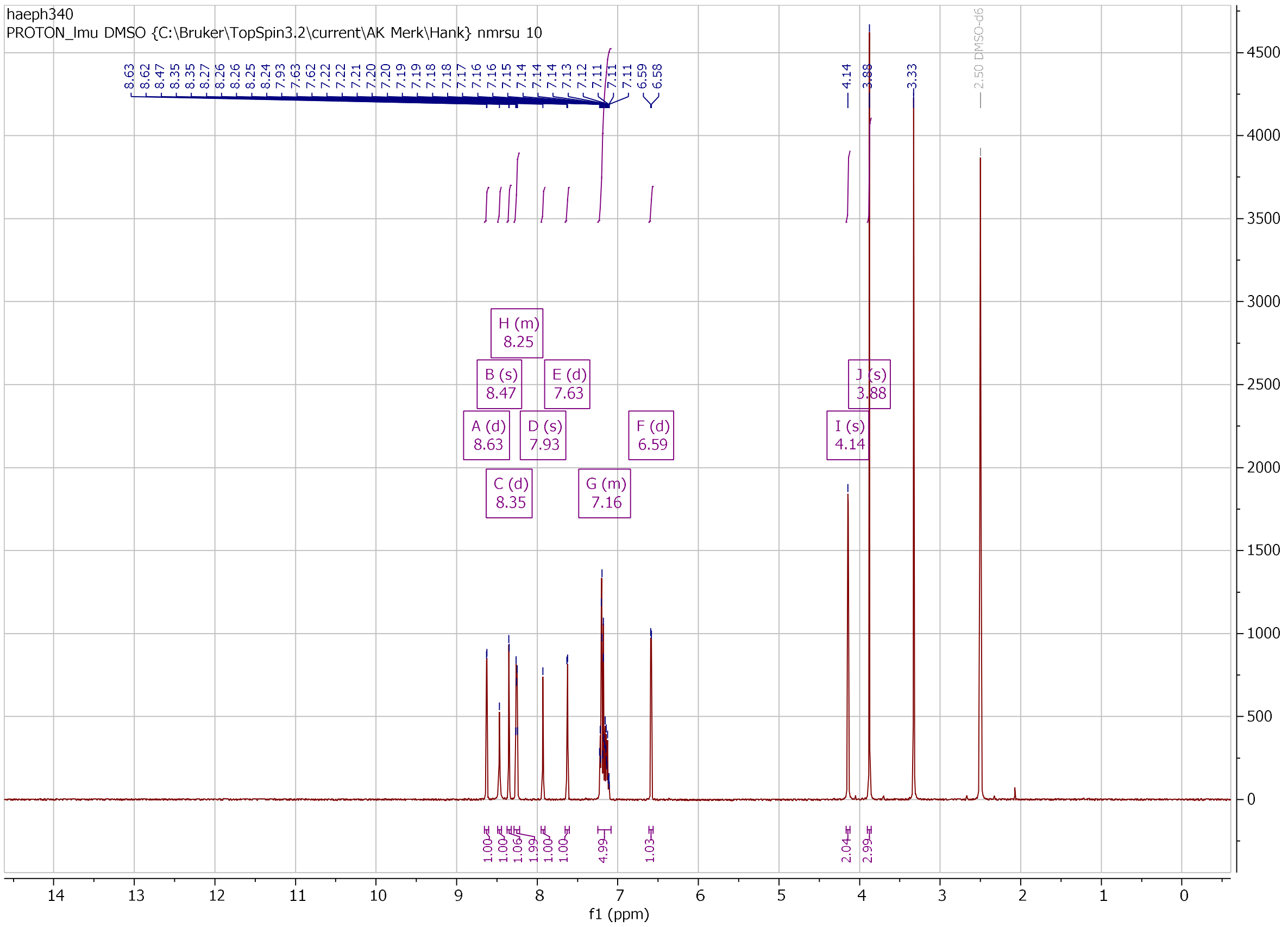
haeph340
PROTON_lmu DMSO {C:\Bruker\TopSpin3.2\current\AK Merk\Hank} nmrsu 10
H (m) 8.25
B (s) 8.47
E (d) 7.63
J (s) 3.88
A (d) 8.63
D (s) 7.93
F (d) 6.59
I (s) 4.14
C (d) 8.35
G (m) 7.16
2.50 DMSO-d6
f1 (ppm)

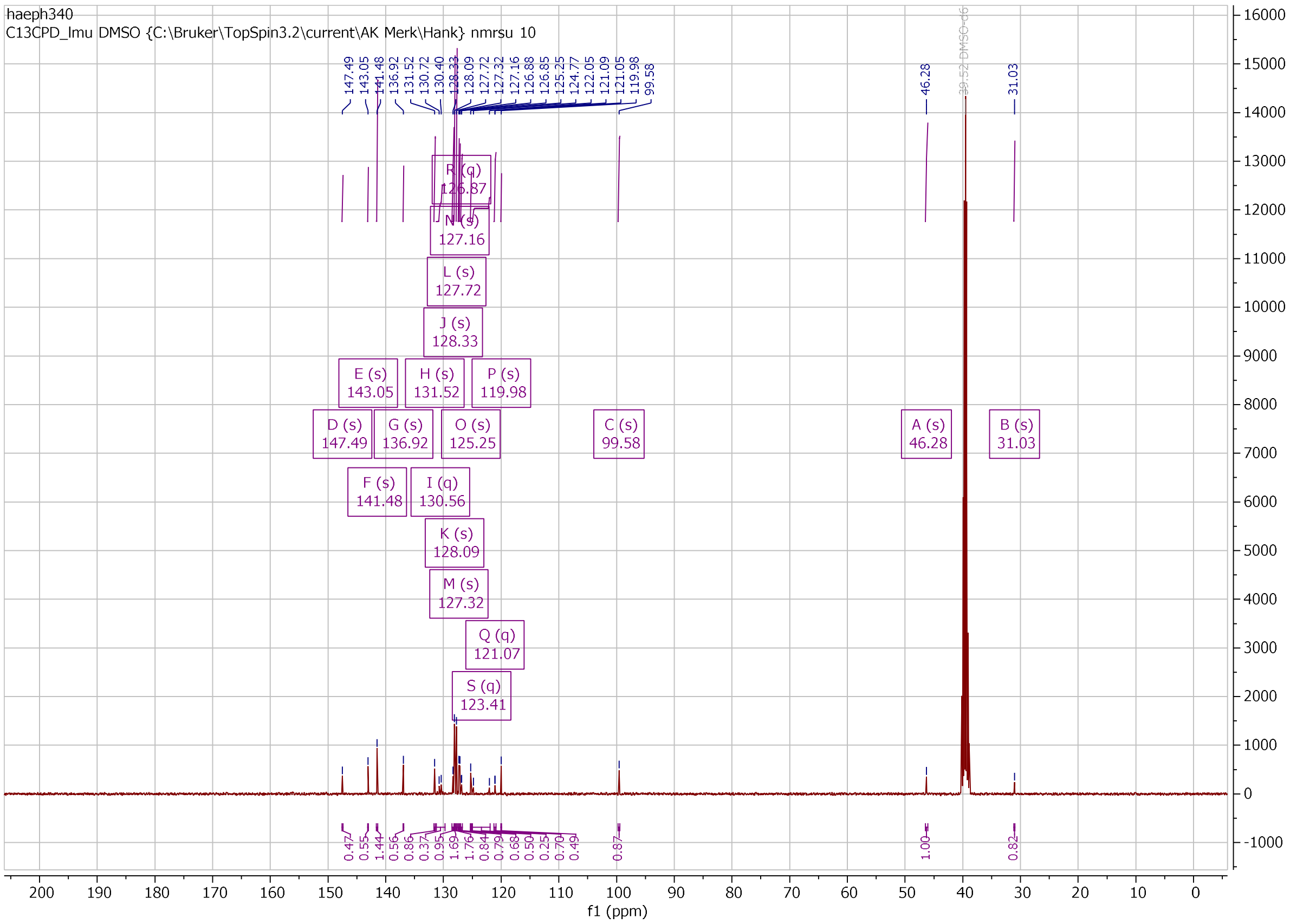
haeph340
C13CPD_lmu DMSO {C:\Bruker\TopSpin3.2\current\AK Merk\Hank} nmrsu 10
147.49
143.05
141.48
136.92
131.52
130.72
130.40
128.33
128.09
127.72
127.32
127.16
126.88
126.85
125.25
124.77
122.05
121.09
121.05
119.98
99.58
46.28
31.03
D (s) 147.49
E (s) 143.05
F (s) 141.48
G (s) 136.92
H (s) 131.52
I (q) 130.56
J (s) 128.33
K (s) 128.09
L (s) 127.72
M (s) 127.32
N (s) 127.16
O (s) 125.25
P (s) 119.98
Q (q) 121.07
R (q) 126.87
S (q) 123.41
C (s) 99.58
A (s) 46.28
B (s) 31.03
f1 (ppm)

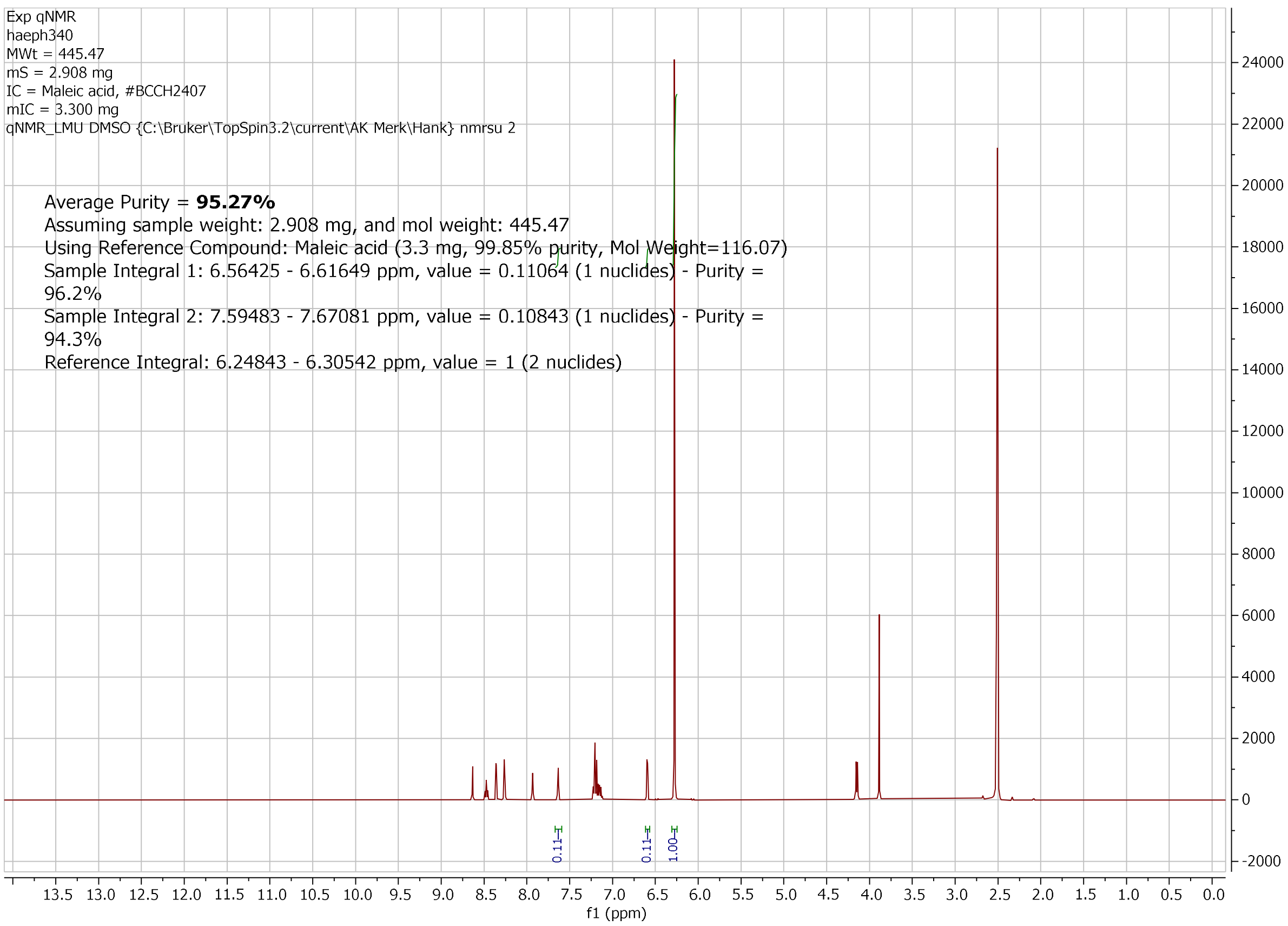
Exp qNMR
haeph340
MWt = 445.47
mS = 2.908 mg
IC = Maleic acid, #BCCH2407
mIC = 3.300 mg
qNMR_LMU DMSO {C:\Bruker\TopSpin3.2\current\AK Merk\Hank} nmrsu 2
Average Purity = 95.27%
Assuming sample weight: 2.908 mg, and mol weight: 445.47
Using Reference Compound: Maleic acid (3.3 mg, 99.85% purity, Mol Weight=116.07)
Sample Integral 1: 6.56425 - 6.61649 ppm, value = 0.11064 (1 nuclides) - Purity = 96.2%
Sample Integral 2: 7.59483 - 7.67081 ppm, value = 0.10843 (1 nuclides) - Purity = 94.3%
Reference Integral: 6.24843 - 6.30542 ppm, value = 1 (2 nuclides)
0.11
0.11
1.00
f1 (ppm)

Compound **38**:

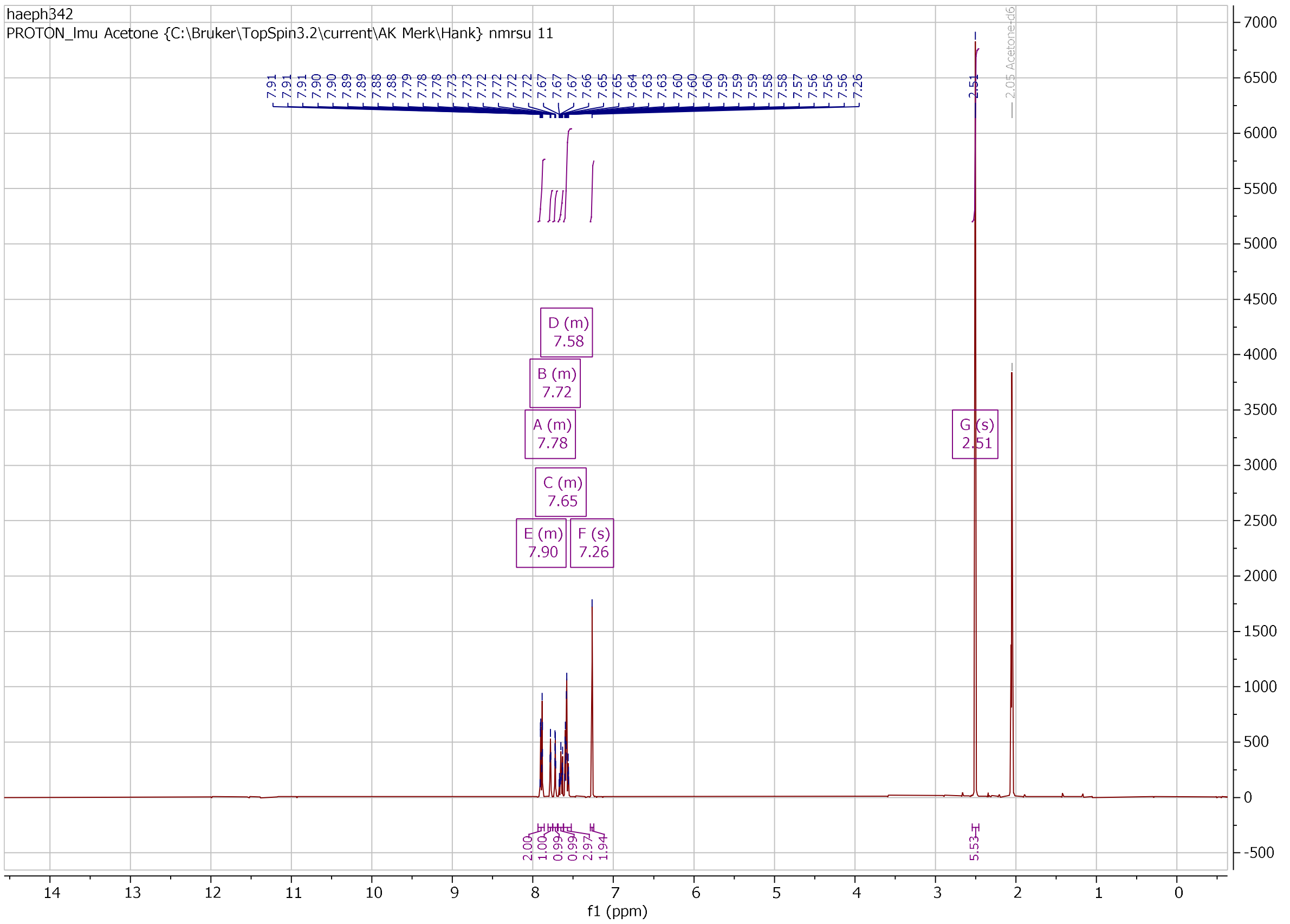
haeph342
PROTON_lmu Acetone {C:\Bruker\TopSpin3.2\current\AK Merk\Hank} nmrsu 11
f1 (ppm)

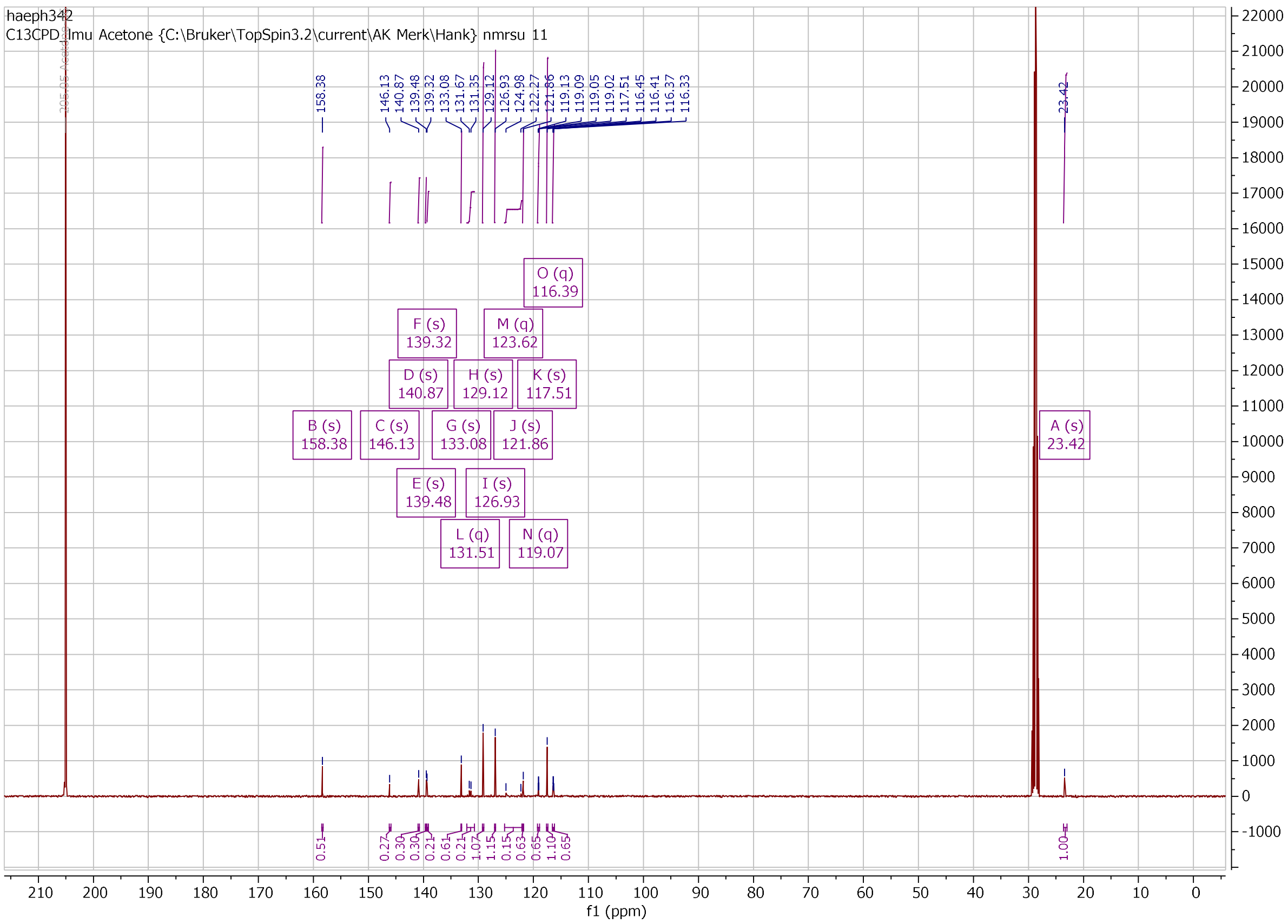
haeph342
C13CPD_lmu Acetone {C:\Bruker\TopSpin3.2\current\AK Merk\Hank} nmrsu 11
f1 (ppm)

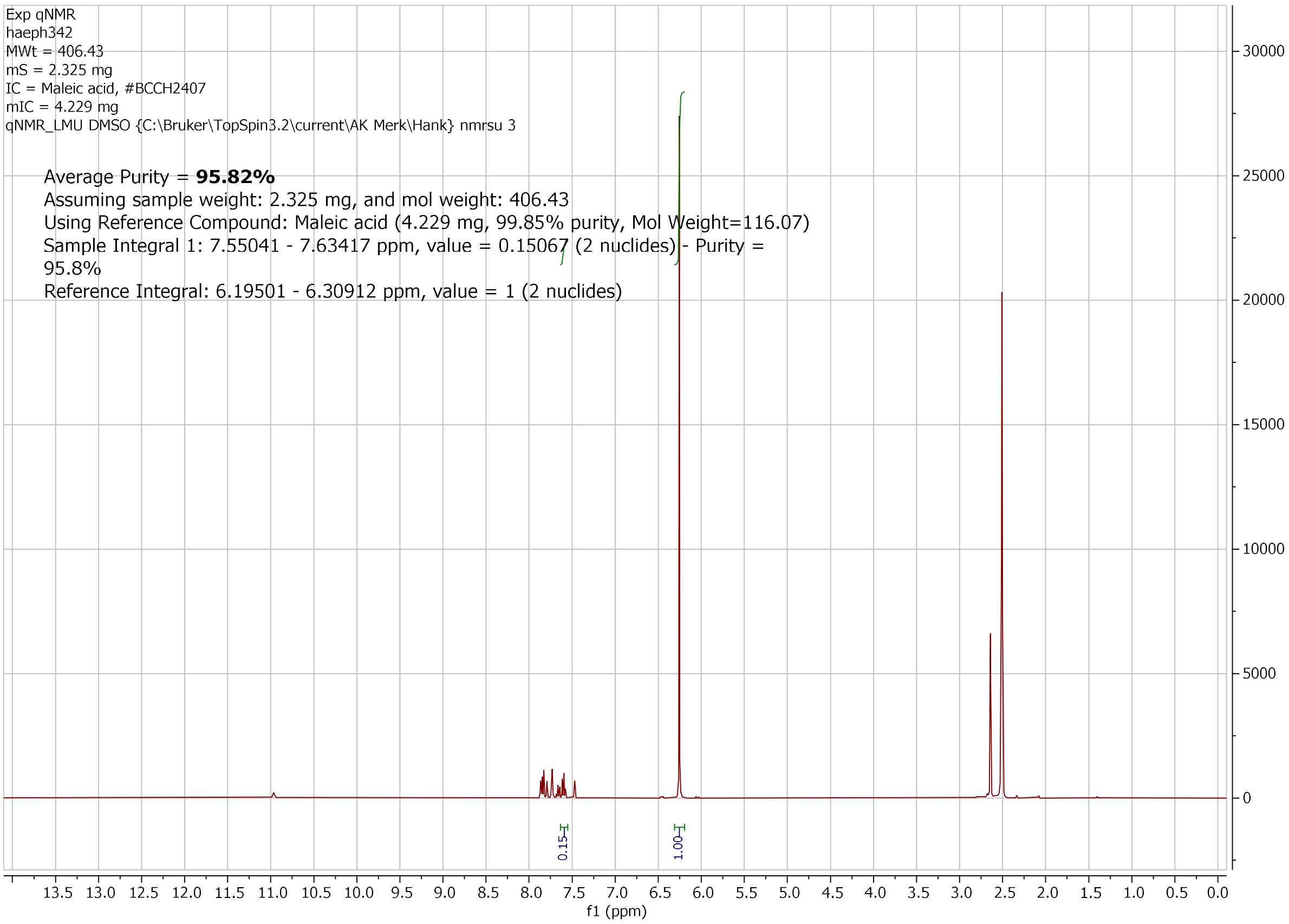
Exp qNMR
haeph342
MWt = 406.43
mS = 2.325 mg
IC = Maleic acid, #BCCH2407
mIC = 4.229 mg
qNMR_LMU DMSO {C:\Bruker\TopSpin3.2\current\AK Merk\Hank} nmrsu 3
Average Purity = **95.82%**
Assuming sample weight: 2.325 mg, and mol weight: 406.43
Using Reference Compound: Maleic acid (4.229 mg, 99.85% purity, Mol Weight=116.07)
Sample Integral 1: 7.55041 - 7.63417 ppm, value = 0.15067 (2 nuclides) - Purity = 95.8%
Reference Integral: 6.19501 - 6.30912 ppm, value = 1 (2 nuclides)
0.15
1.00
f1 (ppm)

Compound **39**:

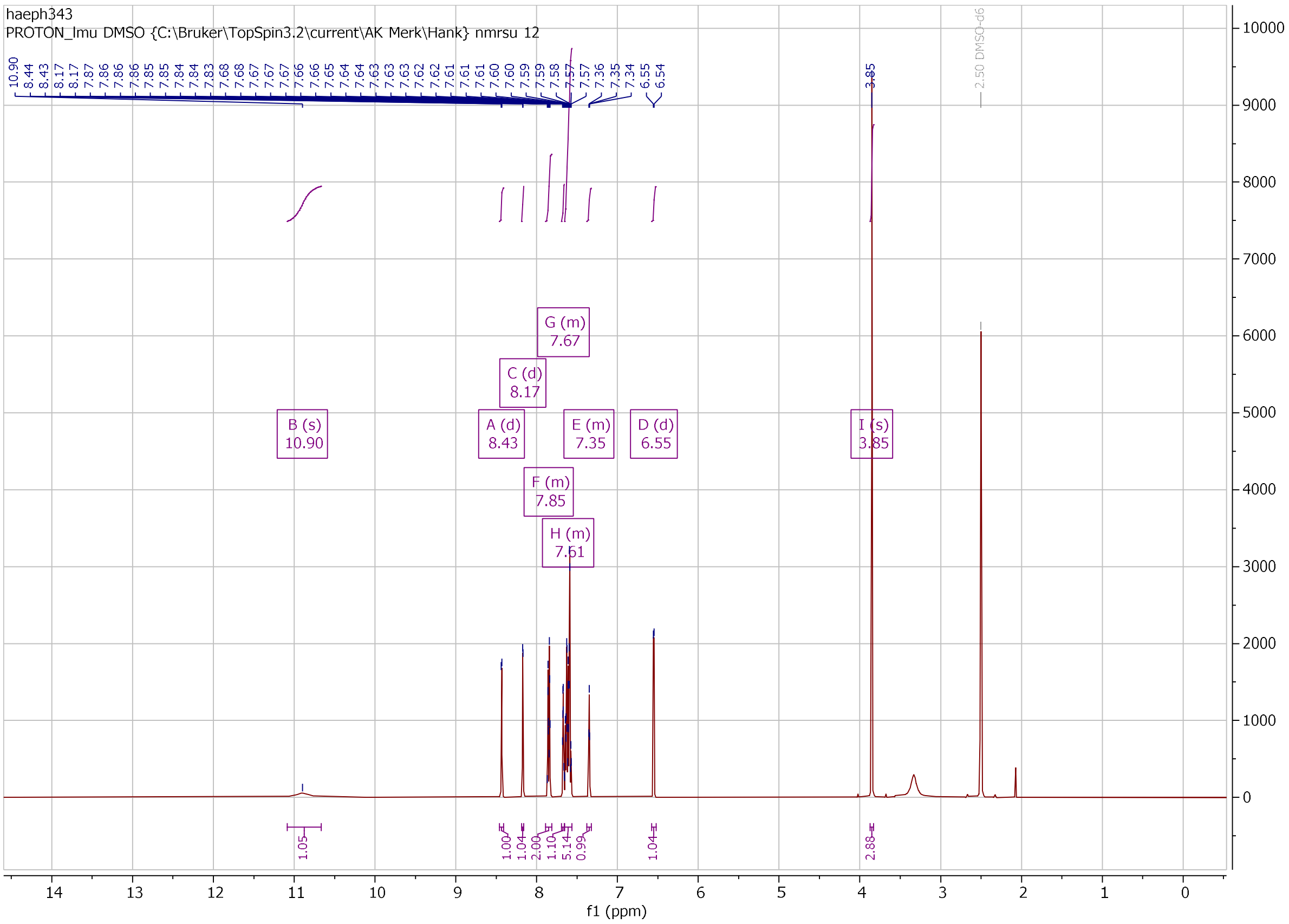
haeph343
PROTON_lmu DMSO {C:\Bruker\TopSpin3.2\current\AK Merk\Hank} nmrsu 12
2.50 DMSO-d6
G (m) 7.67
C (d) 8.17
B (s) 10.90
A (d) 8.43
E (m) 7.35
D (d) 6.55
I (s) 3.85
F (m) 7.85
H (m) 7.61
1.05
1.00
1.04
2.00
1.10
5.14
0.99
1.04
2.88
f1 (ppm)

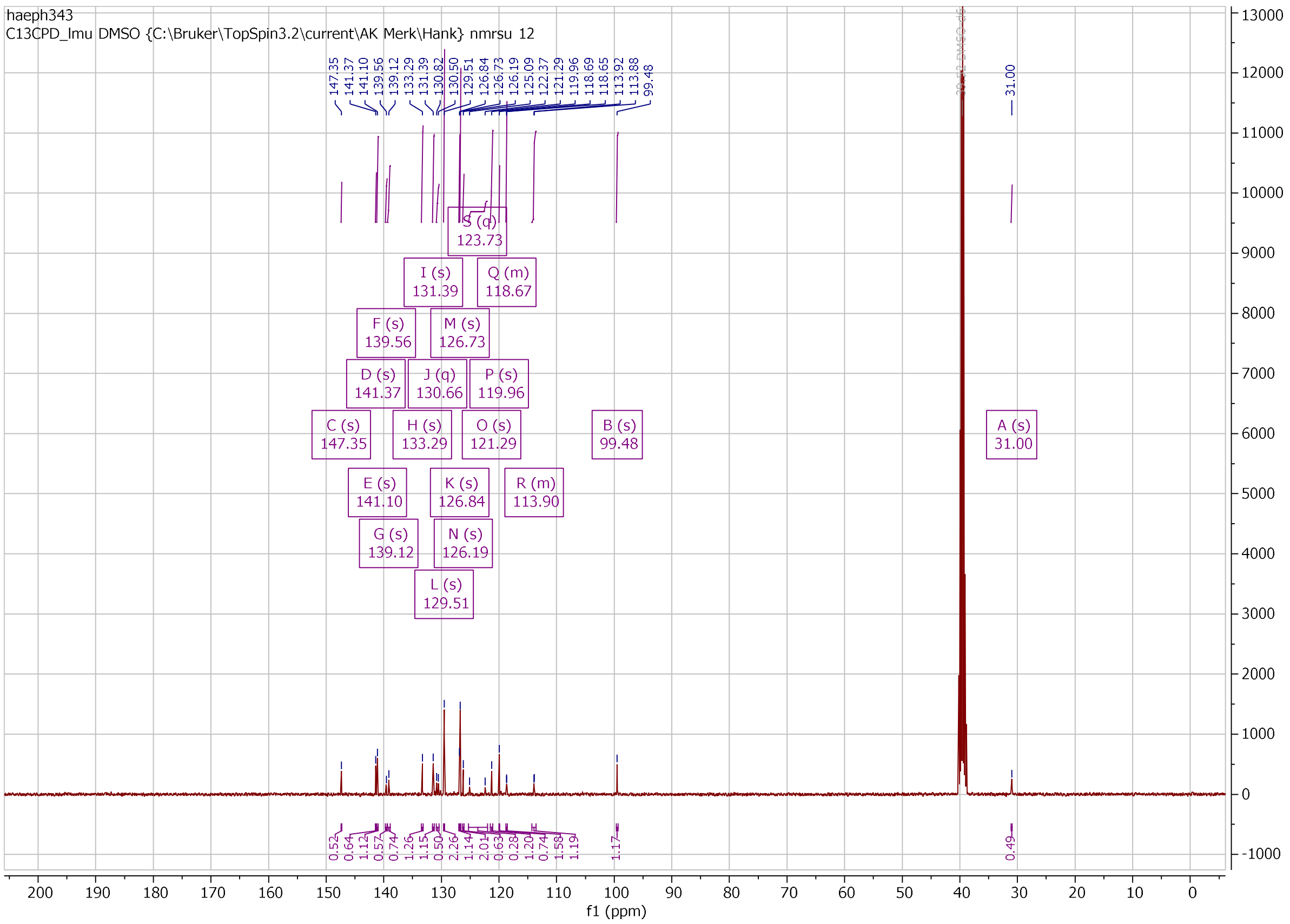
haeph343
C13CPD_lmu DMSO {C:\Bruker\TopSpin3.2\current\AK Merk\Hank} nmrsu 12
147.35
141.37
141.10
139.56
139.12
133.29
131.39
130.82
130.50
129.51
126.84
126.73
126.19
125.09
122.37
121.29
119.96
118.69
118.65
113.92
113.88
99.48
31.00
f1 (ppm)

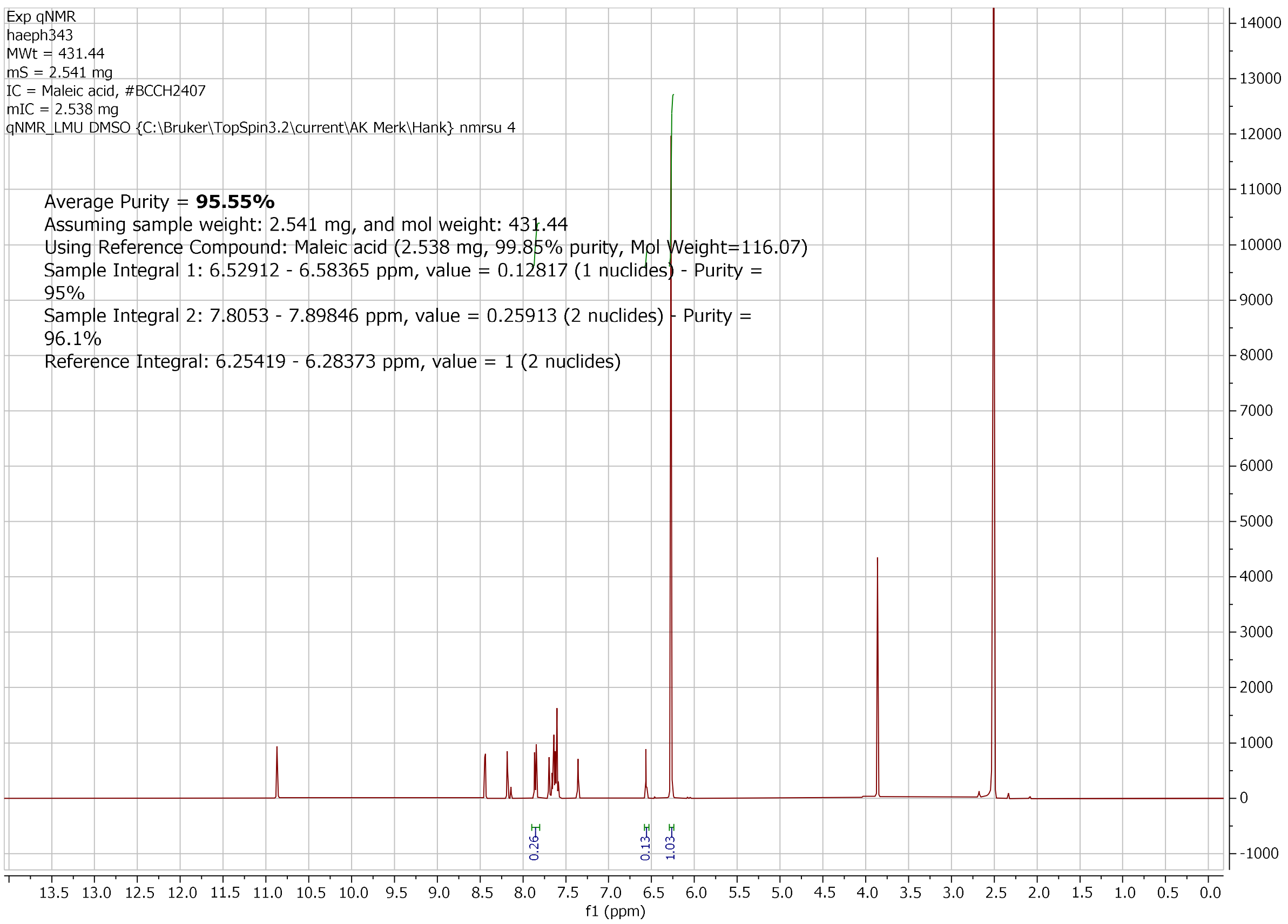
Exp qNMR
haeph343
MWt = 431.44
mS = 2.541 mg
IC = Maleic acid, #BCCH2407
mIC = 2.538 mg
qNMR_LMU DMSO {C:\Bruker\TopSpin3.2\current\AK Merk\Hank} nmrsu 4
Average Purity = 95.55%
Assuming sample weight: 2.541 mg, and mol weight: 431.44
Using Reference Compound: Maleic acid (2.538 mg, 99.85% purity, Mol Weight=116.07)
Sample Integral 1: 6.52912 - 6.58365 ppm, value = 0.12817 (1 nuclides) - Purity = 95%
Sample Integral 2: 7.8053 - 7.89846 ppm, value = 0.25913 (2 nuclides) - Purity = 96.1%
Reference Integral: 6.25419 - 6.28373 ppm, value = 1 (2 nuclides)
f1 (ppm)

Compound **40**:

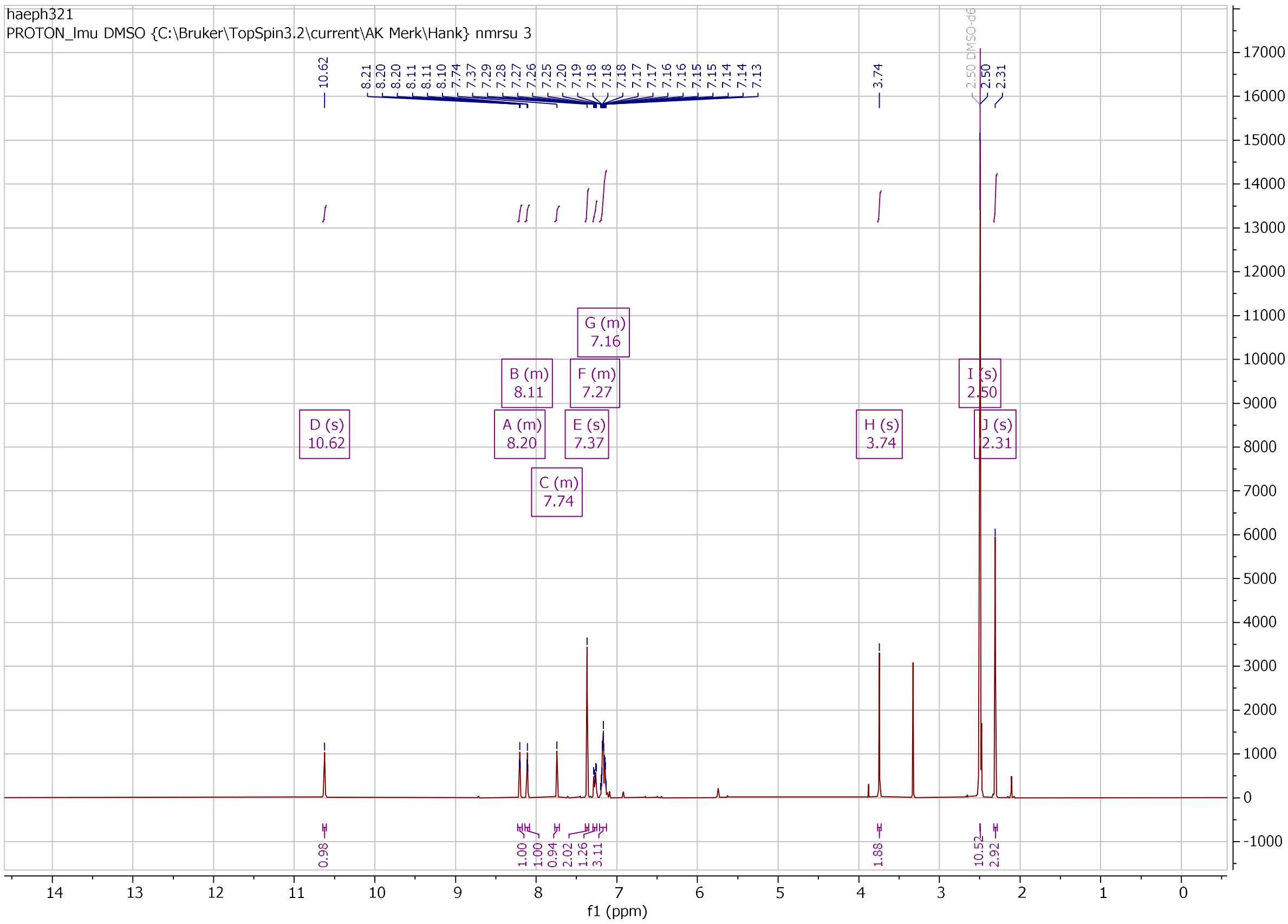
haeph321
PROTON_Imu DMSO {C:\Bruker\TopSpin3.2\current\AK Merk\Hank} nmrsu 3
D (s) 10.62
A (m) 8.20
B (m) 8.11
C (m) 7.74
E (s) 7.37
F (m) 7.27
G (m) 7.16
H (s) 3.74
I (s) 2.50
J (s) 2.31
f1 (ppm)

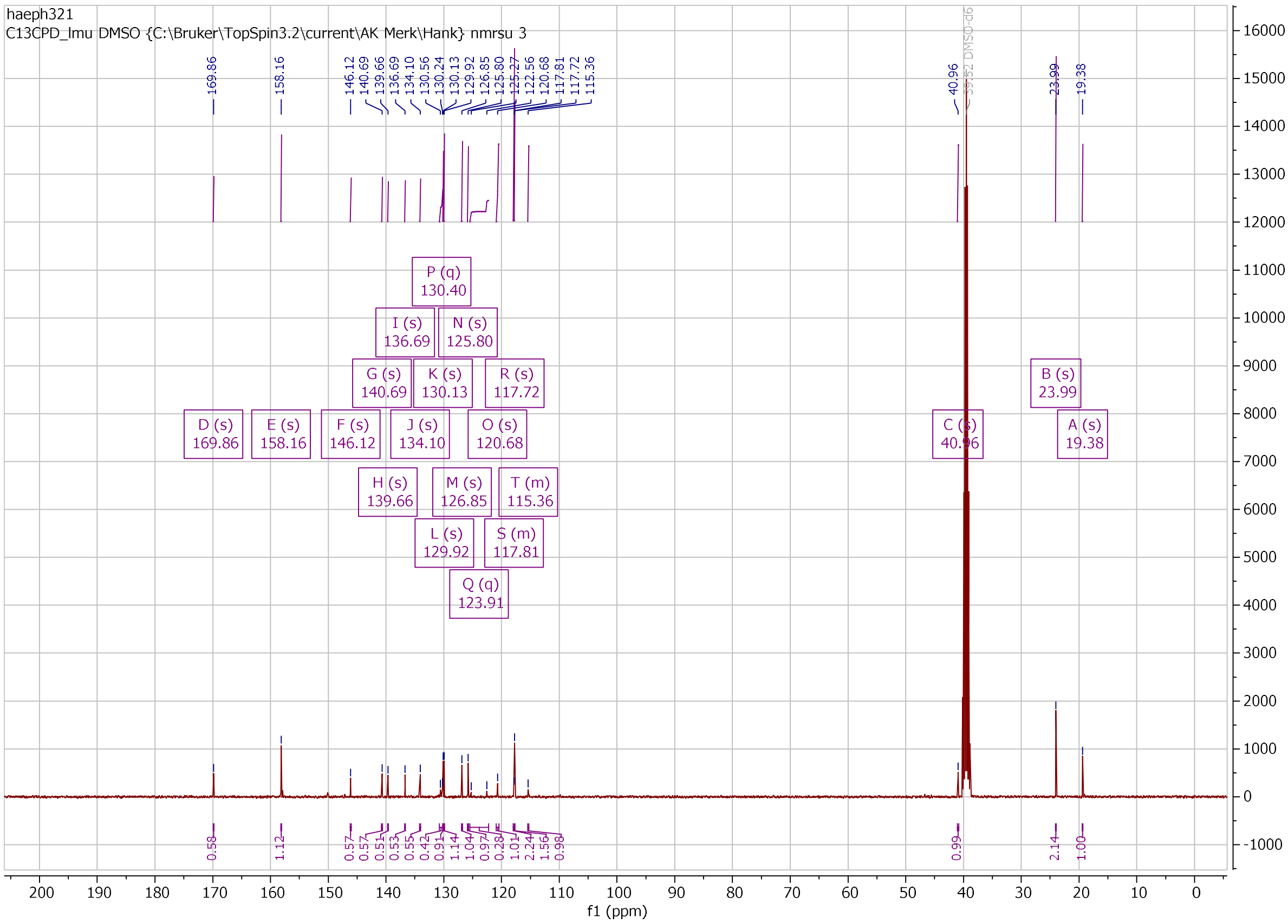
haeph321
C13CPD_Imu DMSO {C:\Bruker\TopSpin3.2\current\AK Merk\Hank} nmrsu 3
D (s) 169.86
E (s) 158.16
F (s) 146.12
G (s) 140.69
H (s) 139.66
I (s) 136.69
J (s) 134.10
P (q) 130.40
K (s) 130.13
L (s) 129.92
M (s) 126.85
N (s) 125.80
Q (q) 123.91
O (s) 120.68
S (m) 117.81
R (s) 117.72
T (m) 115.36
C (s) 40.96
B (s) 23.99
A (s) 19.38
f1 (ppm)

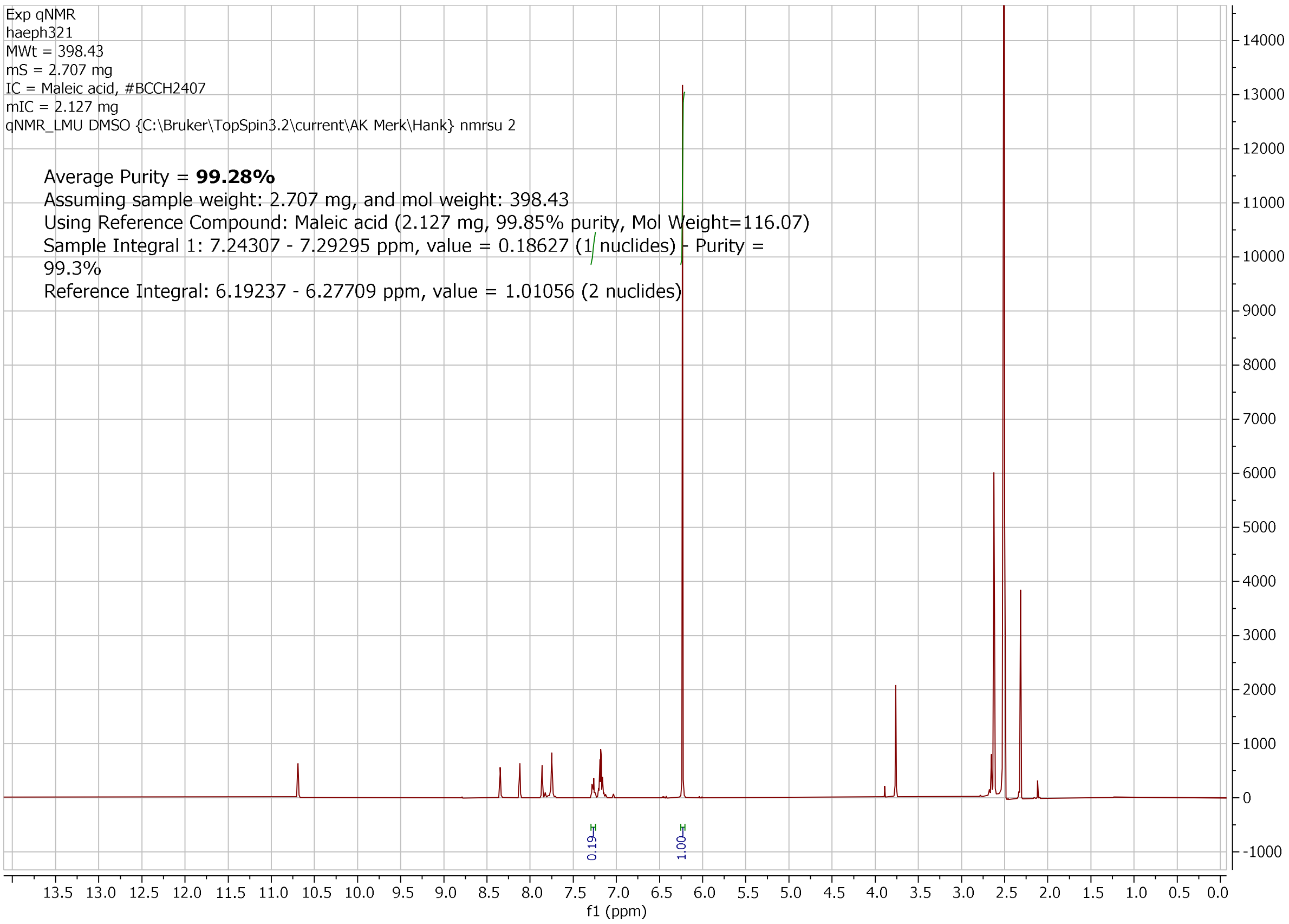
Exp qNMR
haeph321
MWt = 398.43
mS = 2.707 mg
IC = Maleic acid, #BCCH2407
mIC = 2.127 mg
qNMR_LMU DMSO {C:\Bruker\TopSpin3.2\current\AK Merk\Hank} nmrsu 2
Average Purity = 99.28%
Assuming sample weight: 2.707 mg, and mol weight: 398.43
Using Reference Compound: Maleic acid (2.127 mg, 99.85% purity, Mol Weight=116.07)
Sample Integral 1: 7.24307 - 7.29295 ppm, value = 0.18627 (1 nuclides) - Purity = 99.3%
Reference Integral: 6.19237 - 6.27709 ppm, value = 1.01056 (2 nuclides)
f1 (ppm)

Compound **41**:

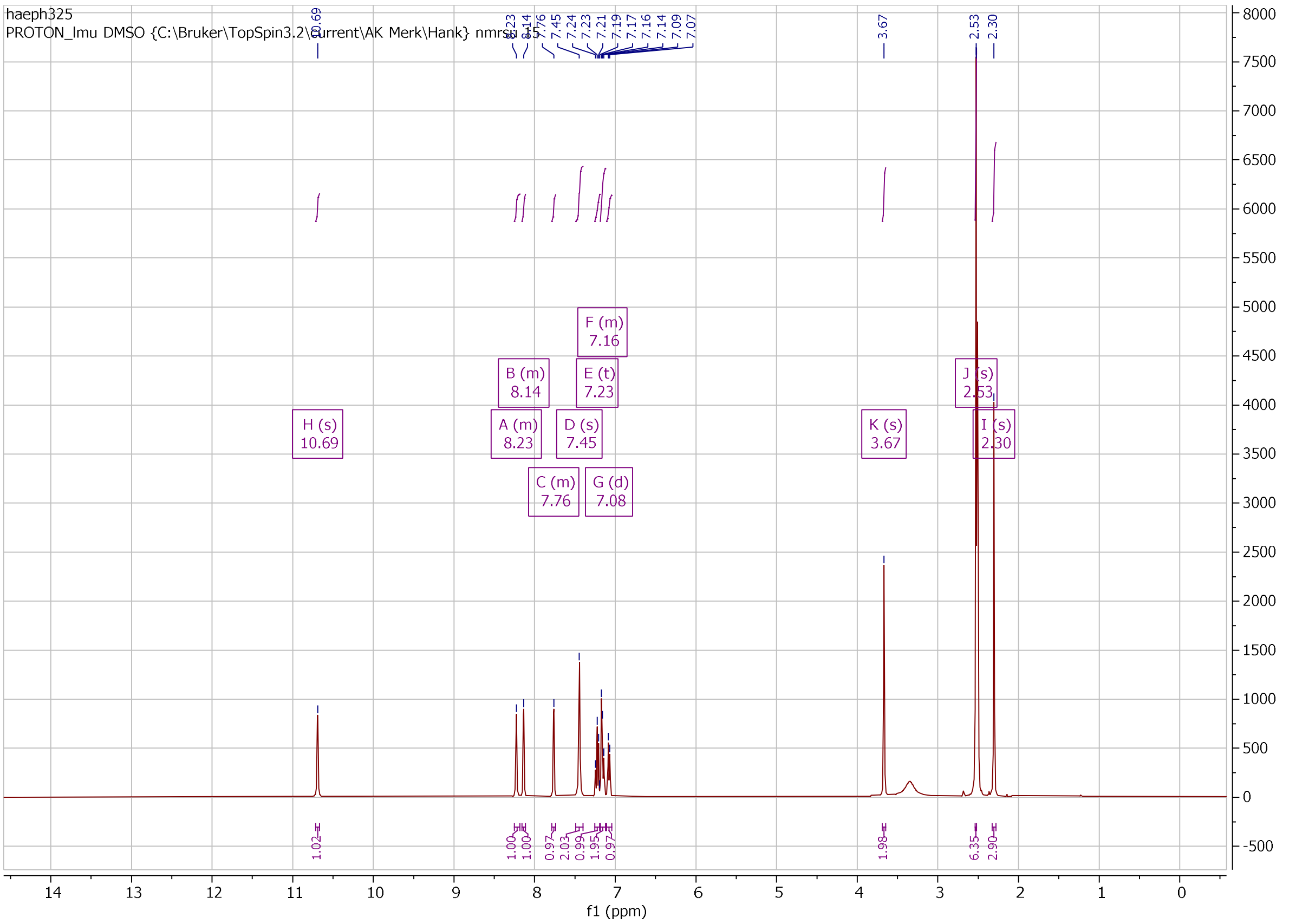
haeph325
PROTON_lmu DMSO {C:\Bruker\TopSpin3.2\current\AK Merk\Hank} nmrsu 35
H (s) 10.69
A (m) 8.23
B (m) 8.14
C (m) 7.76
D (s) 7.45
E (t) 7.23
F (m) 7.16
G (d) 7.08
K (s) 3.67
J (s) 2.53
I (s) 2.30
f1 (ppm)

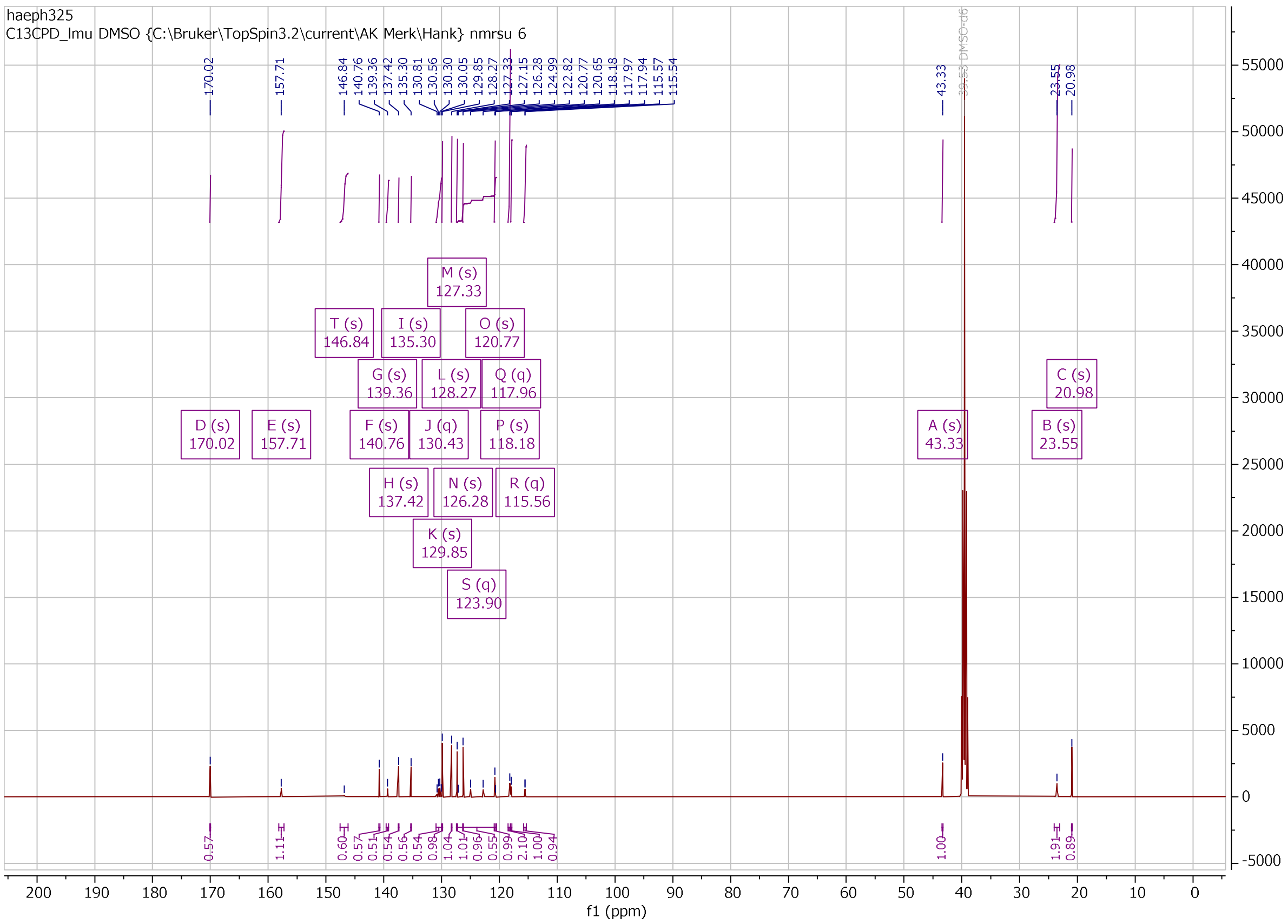
haeph325
C13CPD_lmu DMSO {C:\Bruker\TopSpin3.2\current\AK Merk\Hank} nmrsu 6
f1 (ppm)

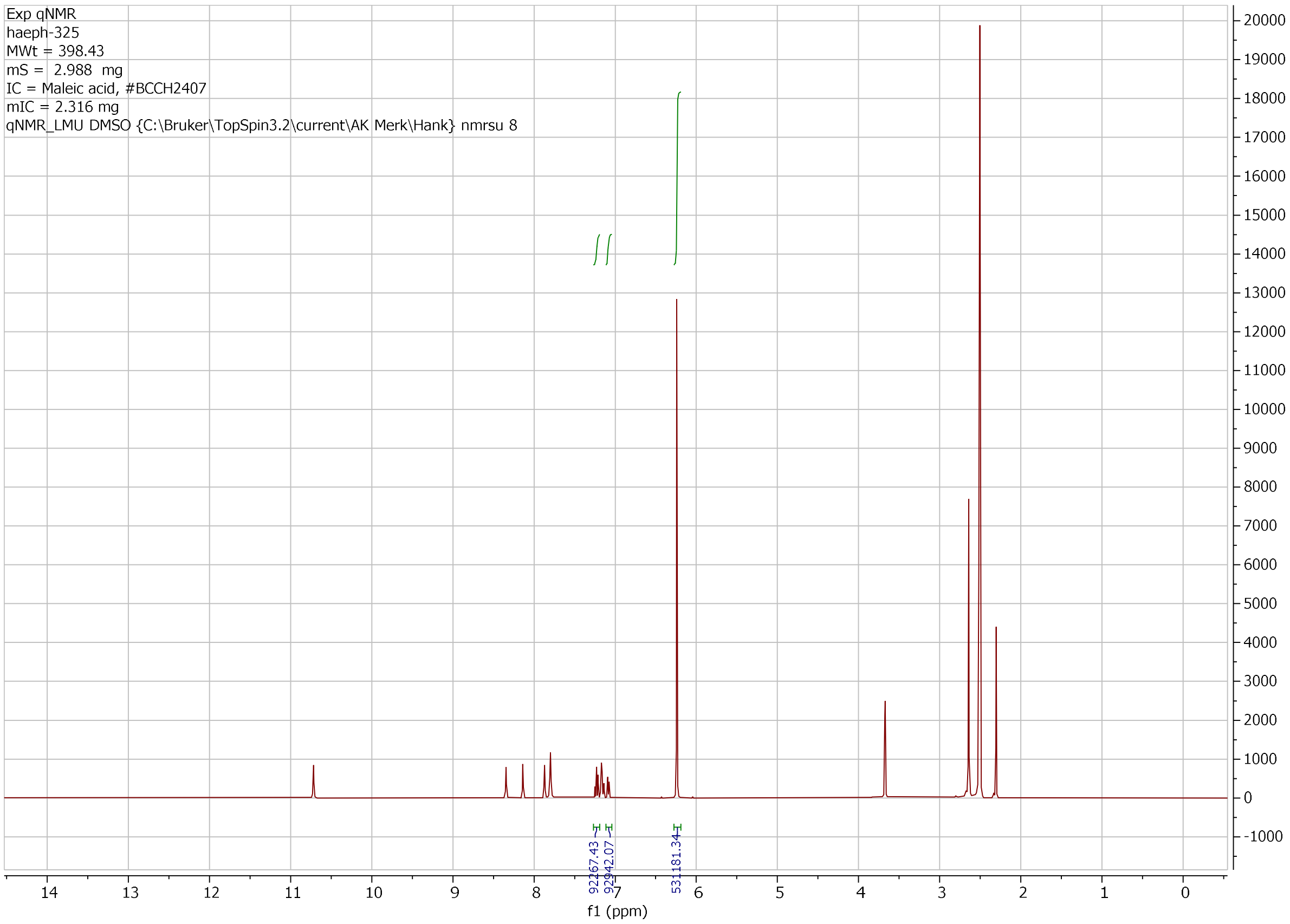
Exp qNMR
haeph-325
MWt = 398.43
mS = 2.988 mg
IC = Maleic acid, #BCCH2407
mIC = 2.316 mg
qNMR_LMU DMSO {C:\Bruker\TopSpin3.2\current\AK Merk\Hank} nmrsu 8
f1 (ppm)

Compound **42**:

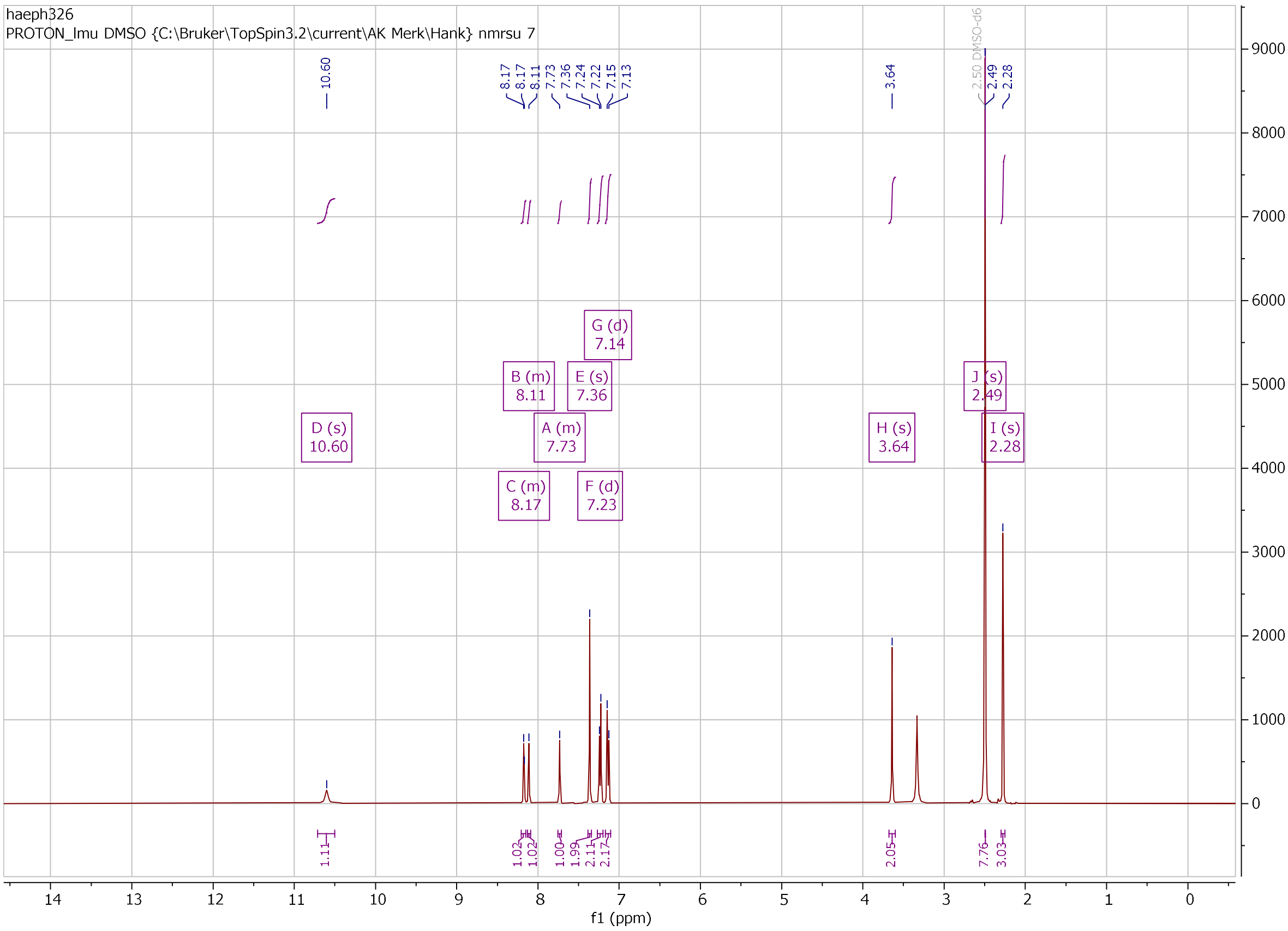
haeph326
PROTON_Imu DMSO {C:\Bruker\TopSpin3.2\current\AK Merk\Hank} nmrsu 7
10.60
8.17 8.17 8.11 8.11 7.73 7.36 7.24 7.22 7.15 7.13
3.64
2.50 DMSO-d6 2.49 2.28
G (d) 7.14
B (m) 8.11
E (s) 7.36
J (s) 2.49
D (s) 10.60
A (m) 7.73
H (s) 3.64
I (s) 2.28
C (m) 8.17
F (d) 7.23
1.11 1.03 1.02 1.00 1.99 2.11 2.17 2.05 7.76 3.03
f1 (ppm)

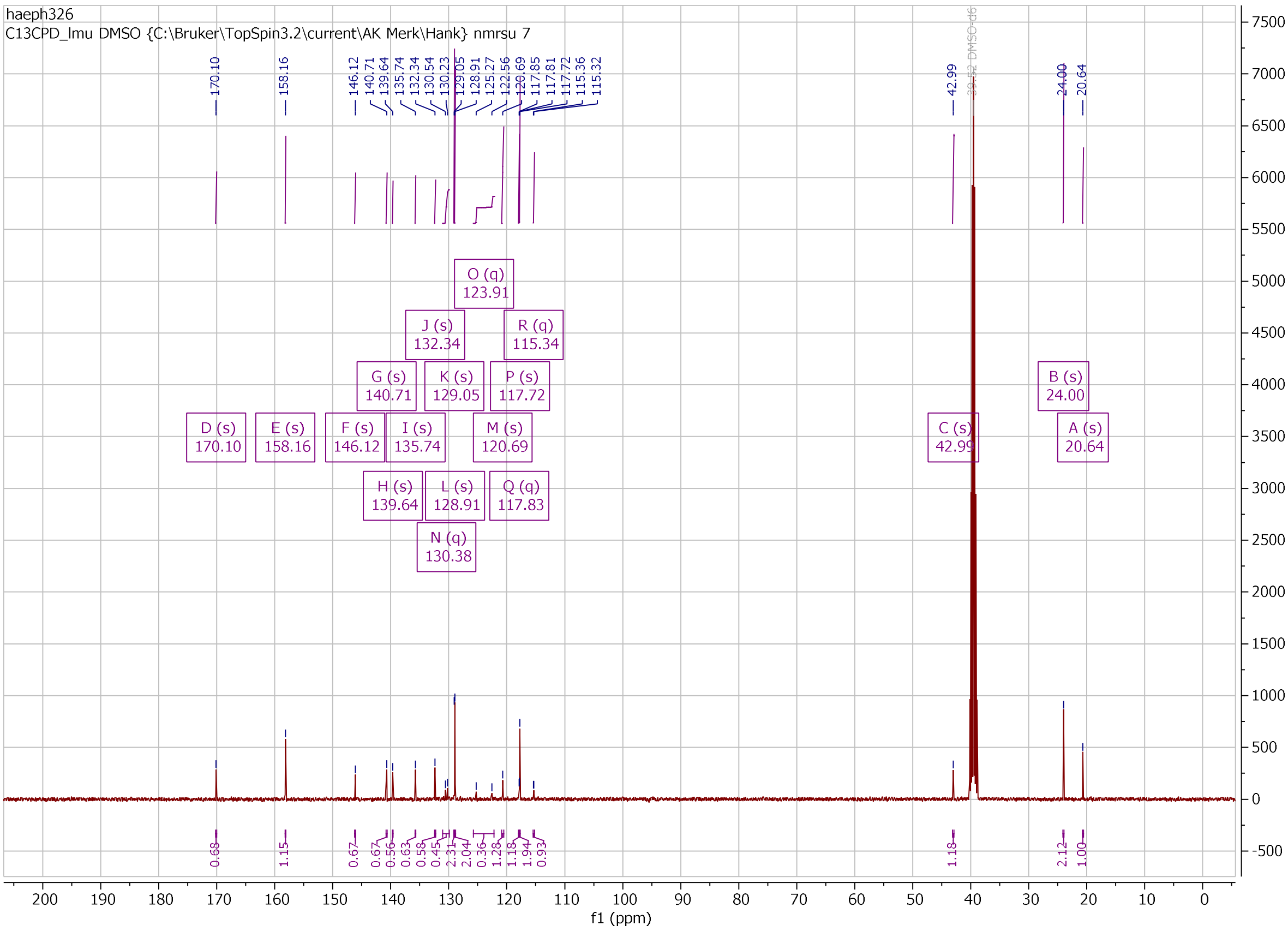
haeph326
C13CPD_Imu DMSO {C:\Bruker\TopSpin3.2\current\AK Merk\Hank} nmrsu 7
170.10 158.16 146.12 140.71 139.64 135.74 132.34 130.54 130.23 129.05 128.91 125.27 122.56 120.69 117.85 117.81 117.72 115.36 115.32
42.99 39.52 DMSO-d6 24.00 20.64
O (q) 123.91
J (s) 132.34
R (q) 115.34
G (s) 140.71
K (s) 129.05
P (s) 117.72
B (s) 24.00
D (s) 170.10
E (s) 158.16
F (s) 146.12
I (s) 135.74
M (s) 120.69
C (s) 42.99
A (s) 20.64
H (s) 139.64
L (s) 128.91
Q (q) 117.83
N (q) 130.38
f1 (ppm)

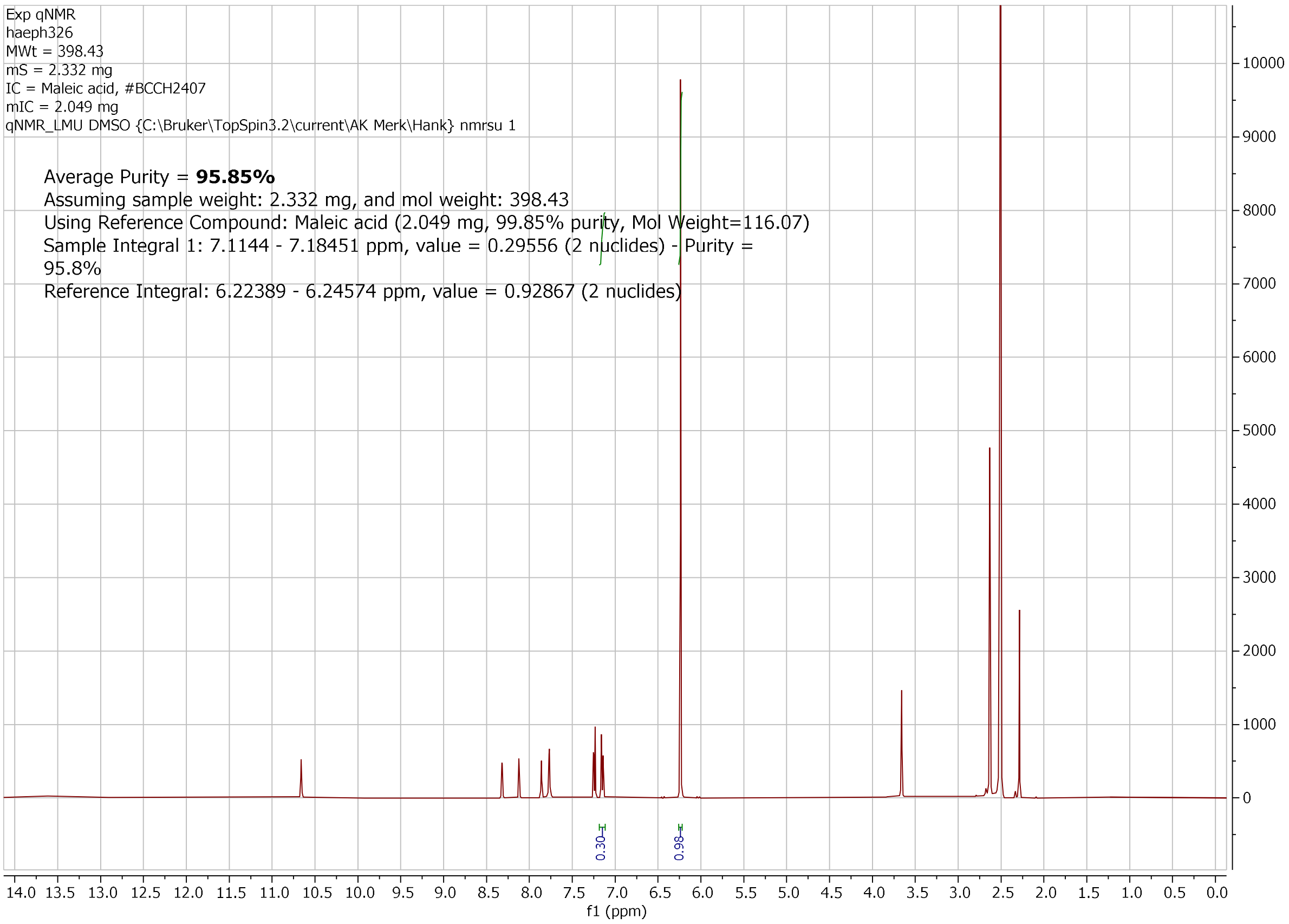
Exp qNMR
haeph326
MWt = 398.43
mS = 2.332 mg
IC = Maleic acid, #BCCH2407
mIC = 2.049 mg
qNMR_LMU DMSO {C:\Bruker\TopSpin3.2\current\AK Merk\Hank} nmrsu 1
Average Purity = **95.85%**
Assuming sample weight: 2.332 mg, and mol weight: 398.43
Using Reference Compound: Maleic acid (2.049 mg, 99.85% purity, Mol Weight=116.07)
Sample Integral 1: 7.1144 - 7.18451 ppm, value = 0.29556 (2 nuclides) - Purity = 95.8%
Reference Integral: 6.22389 - 6.24574 ppm, value = 0.92867 (2 nuclides)
f1 (ppm)

Compound **43**:

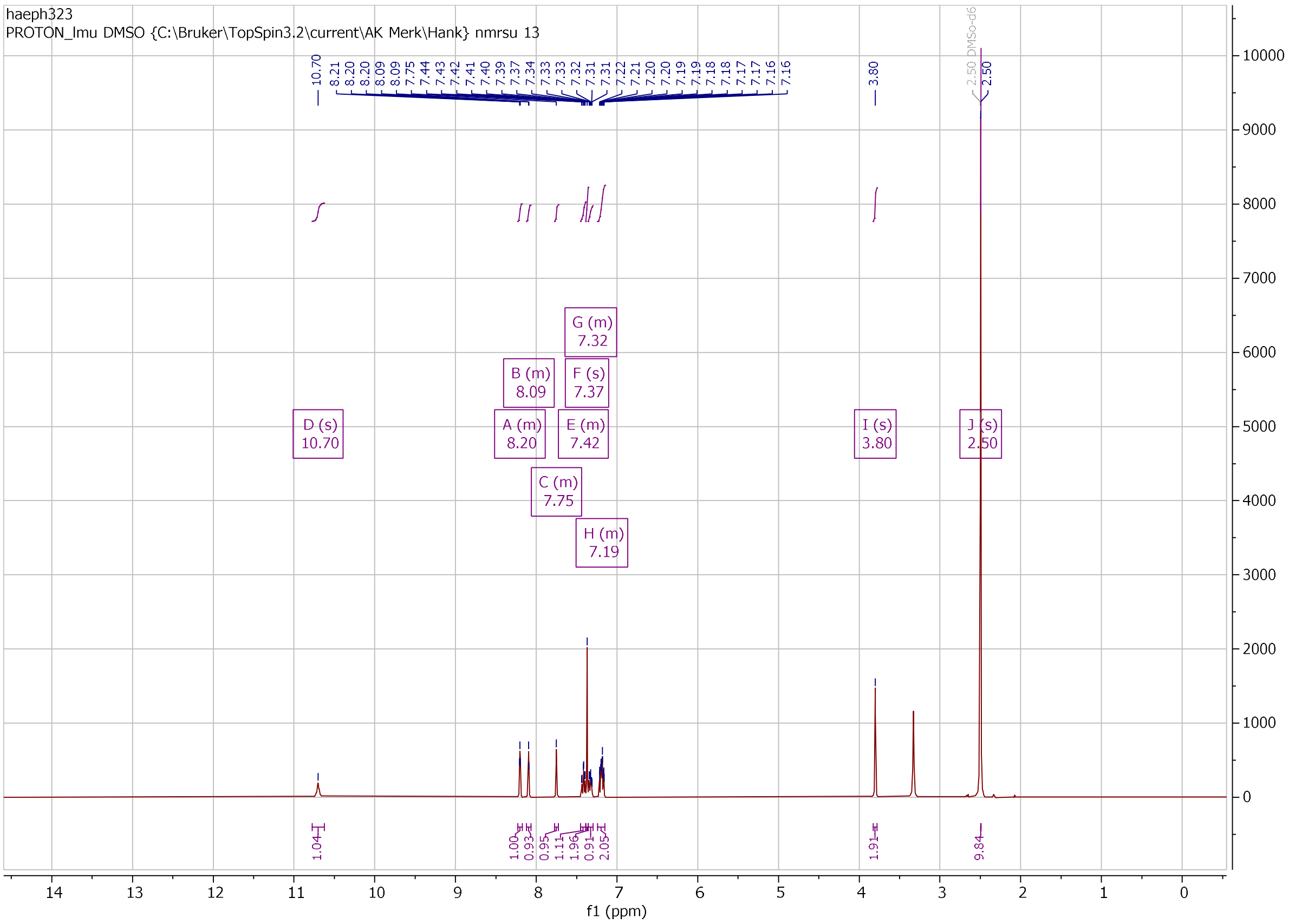
haeph323
PROTON_lmu DMSO {C:\Bruker\TopSpin3.2\current\AK Merk\Hank} nmrsu 13
D (s) 10.70
A (m) 8.20
B (m) 8.09
C (m) 7.75
E (m) 7.42
F (s) 7.37
G (m) 7.32
H (m) 7.19
I (s) 3.80
J (s) 2.50
f1 (ppm)

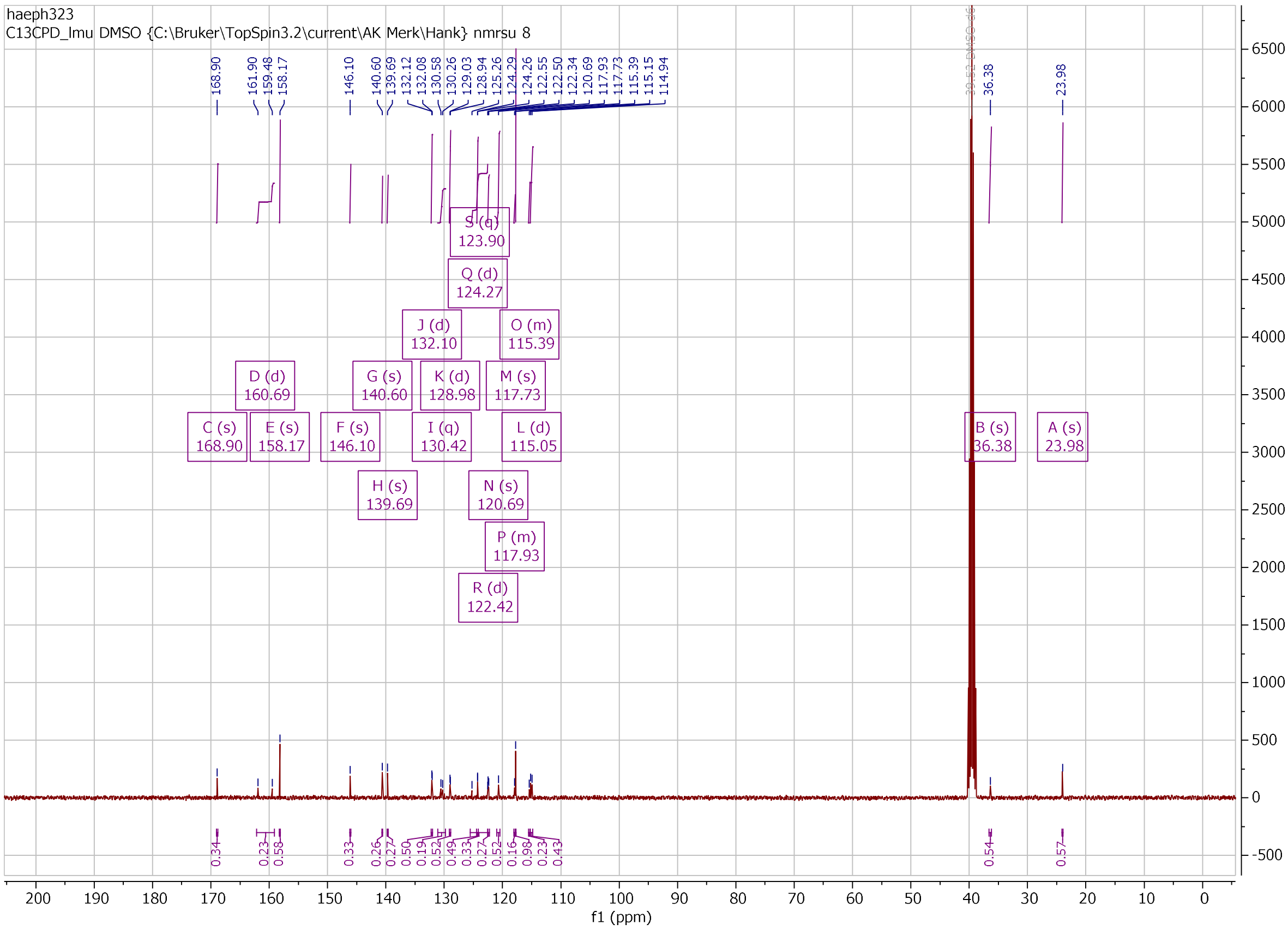
haeph323
C13CPD_lmu DMSO {C:\Bruker\TopSpin3.2\current\AK Merk\Hank} nmrsu 8
168.90
161.90
159.48
158.17
146.10
140.60
139.69
132.12
132.08
130.58
130.26
129.03
128.94
125.26
124.29
124.26
122.55
122.50
122.34
120.69
117.93
117.73
115.39
115.15
114.94
36.38
23.98
S (q) 123.90
Q (d) 124.27
J (d) 132.10
O (m) 115.39
D (d) 160.69
G (s) 140.60
K (d) 128.98
M (s) 117.73
C (s) 168.90
E (s) 158.17
F (s) 146.10
I (q) 130.42
L (d) 115.05
B (s) 36.38
A (s) 23.98
H (s) 139.69
N (s) 120.69
P (m) 117.93
R (d) 122.42
f1 (ppm)

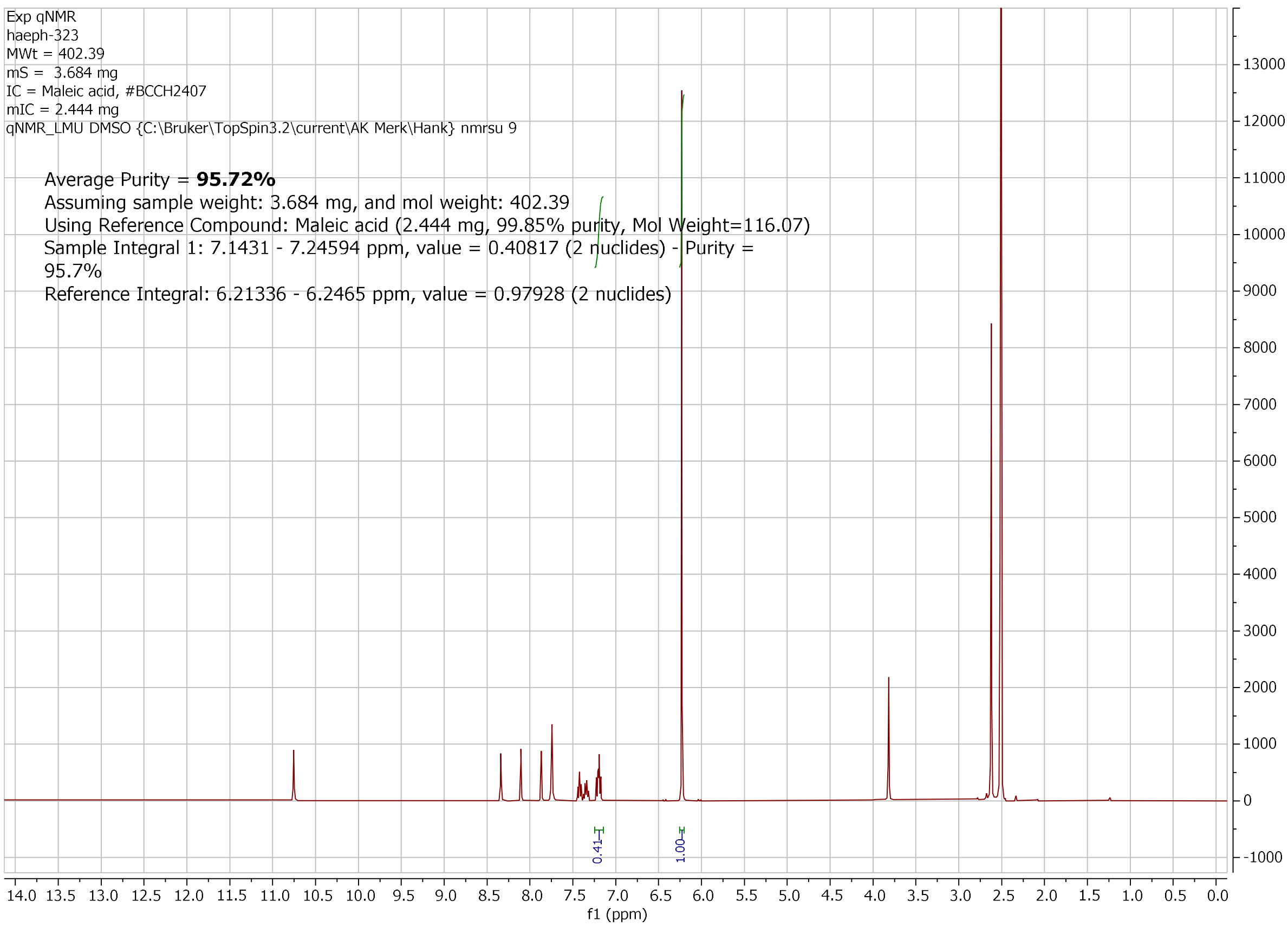
Exp qNMR
haeph-323
MWt = 402.39
mS = 3.684 mg
IC = Maleic acid, #BCCH2407
mIC = 2.444 mg
qNMR_LMU DMSO {C:\Bruker\TopSpin3.2\current\AK Merk\Hank} nmrsu 9
Average Purity = 95.72%
Assuming sample weight: 3.684 mg, and mol weight: 402.39
Using Reference Compound: Maleic acid (2.444 mg, 99.85% purity, Mol Weight=116.07)
Sample Integral 1: 7.1431 - 7.24594 ppm, value = 0.40817 (2 nuclides) - Purity = 95.7%
Reference Integral: 6.21336 - 6.2465 ppm, value = 0.97928 (2 nuclides)
0.41
1.00
f1 (ppm)

Compound **44**:

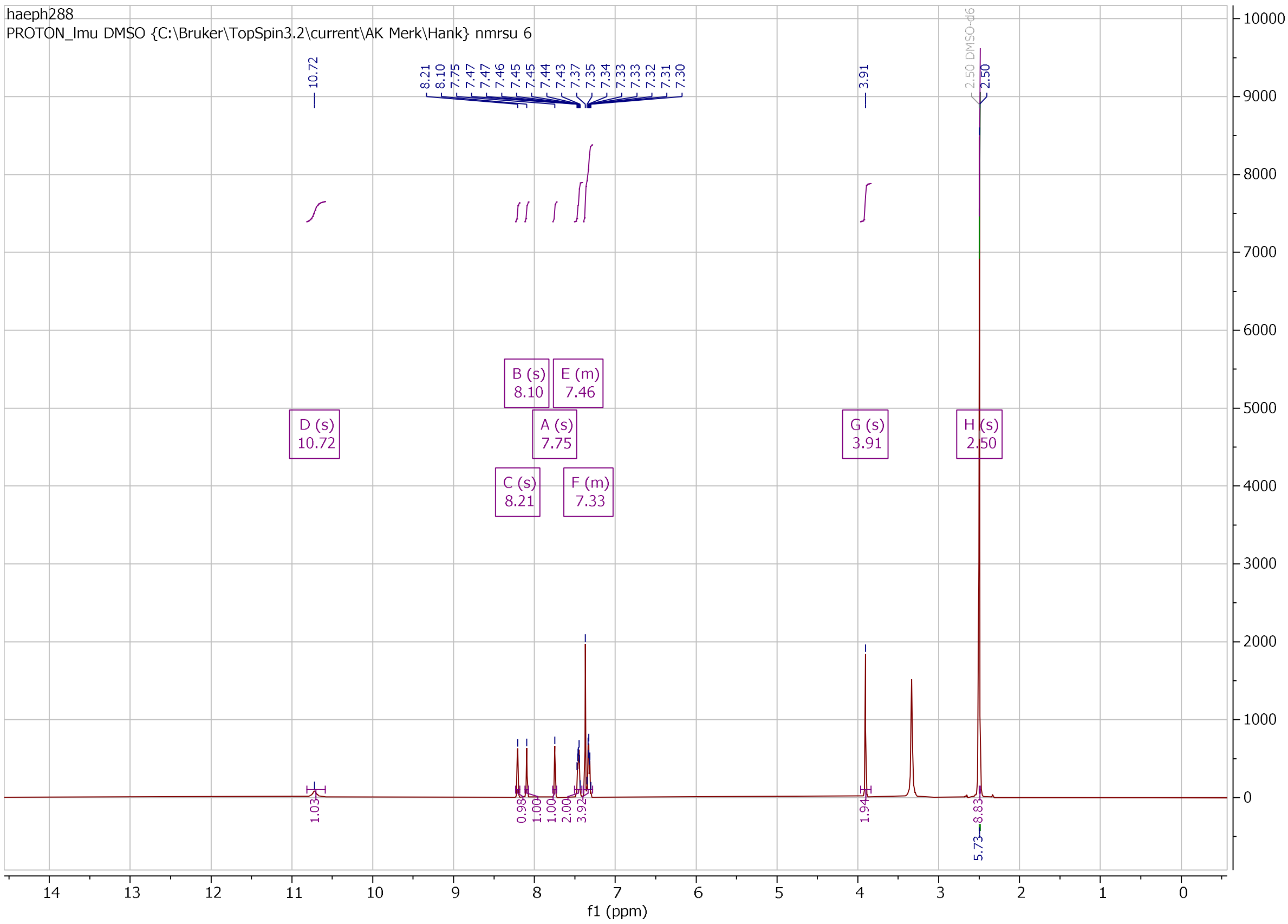

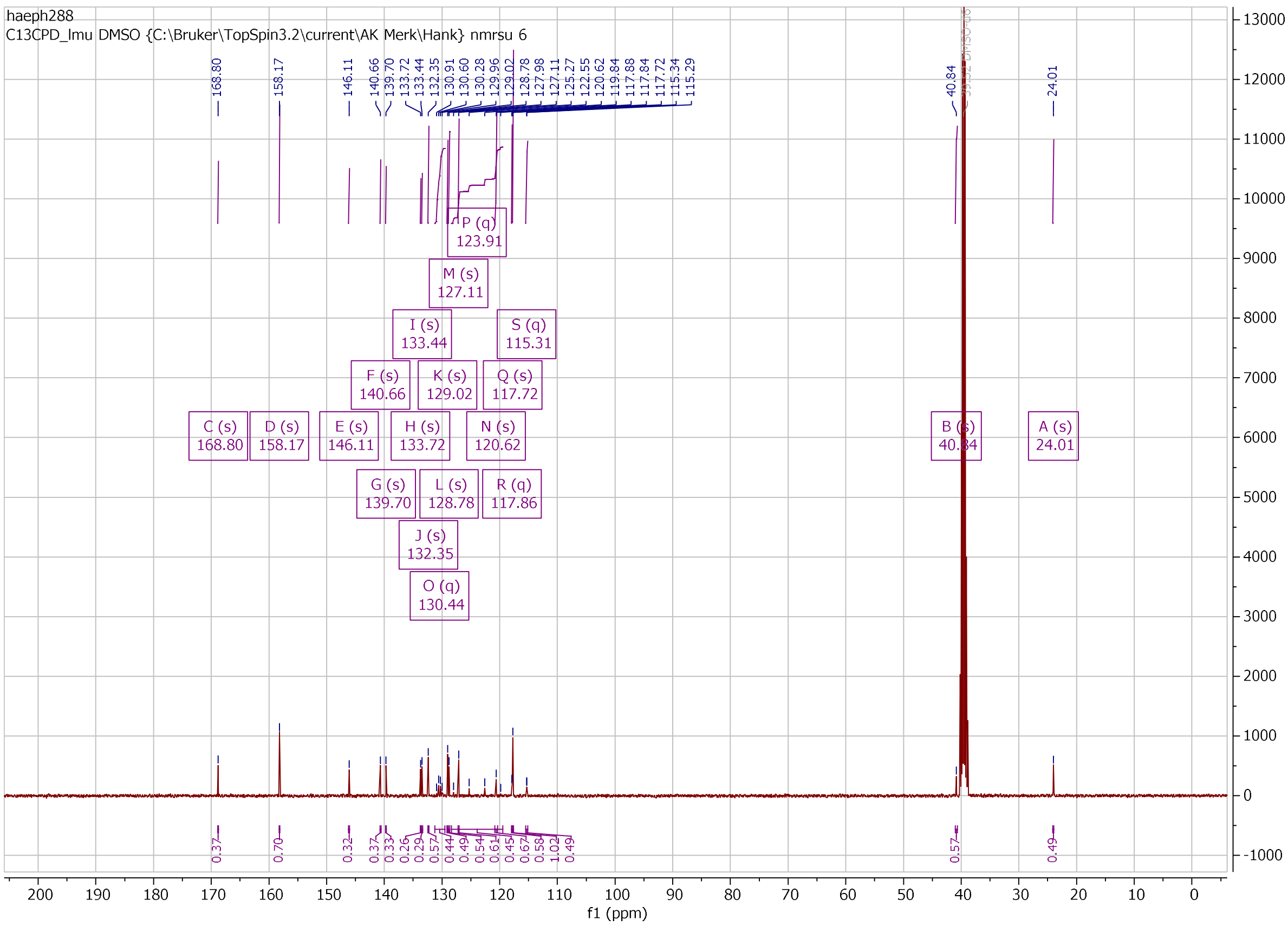

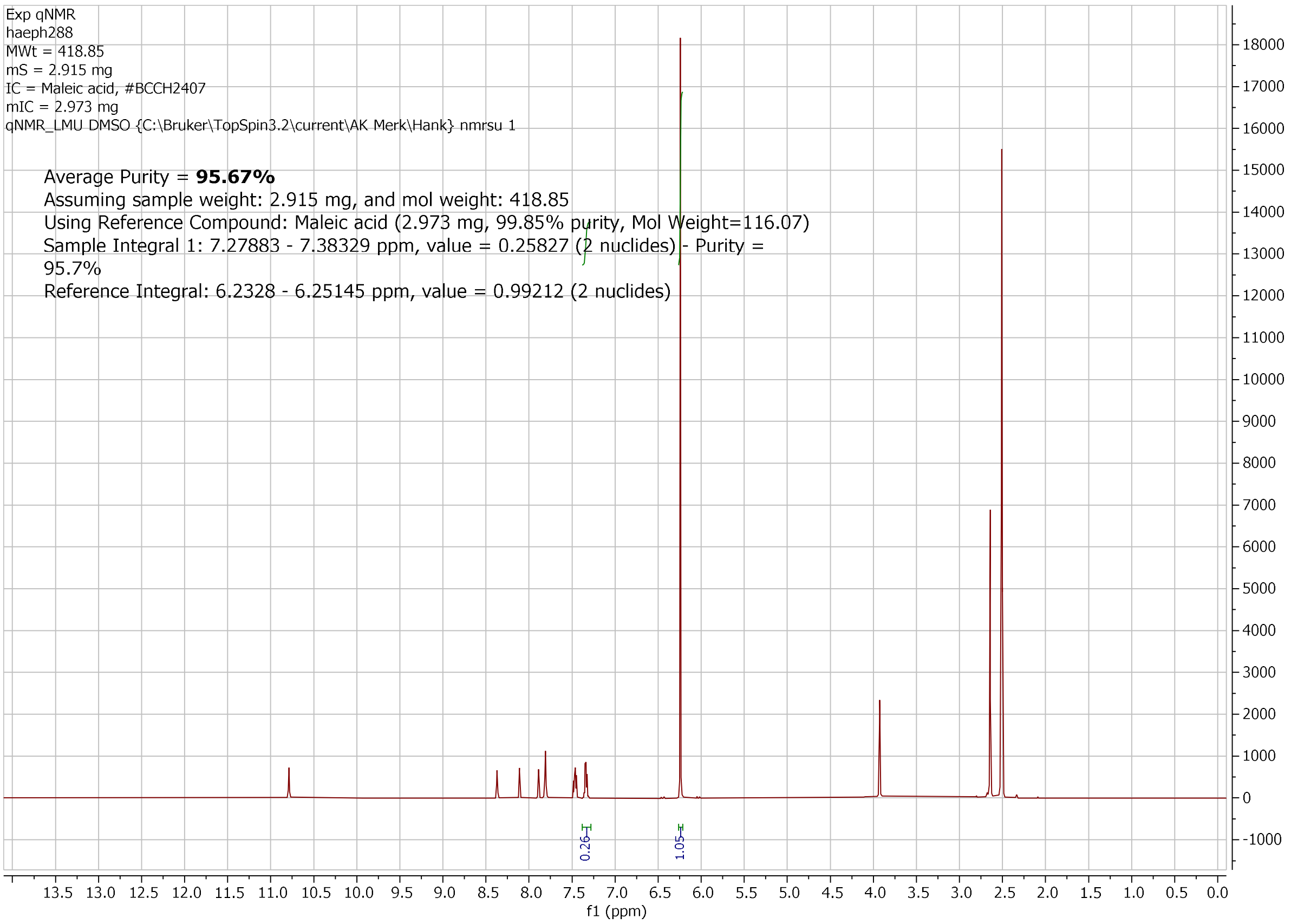
Exp qNMR
haeph288
MWt = 418.85
mS = 2.915 mg
IC = Maleic acid, #BCCH2407
mIC = 2.973 mg
qNMR_LMU DMSO {C:\Bruker\TopSpin3.2\current\AK Merk\Hank} nmrsu 1
Average Purity = **95.67%**
Assuming sample weight: 2.915 mg, and mol weight: 418.85
Using Reference Compound: Maleic acid (2.973 mg, 99.85% purity, Mol Weight=116.07)
Sample Integral 1: 7.27883 - 7.38329 ppm, value = 0.25827 (2 nuclides) - Purity = 95.7%
Reference Integral: 6.2328 - 6.25145 ppm, value = 0.99212 (2 nuclides)
0.26
1.05
f1 (ppm)

Compound **45**:

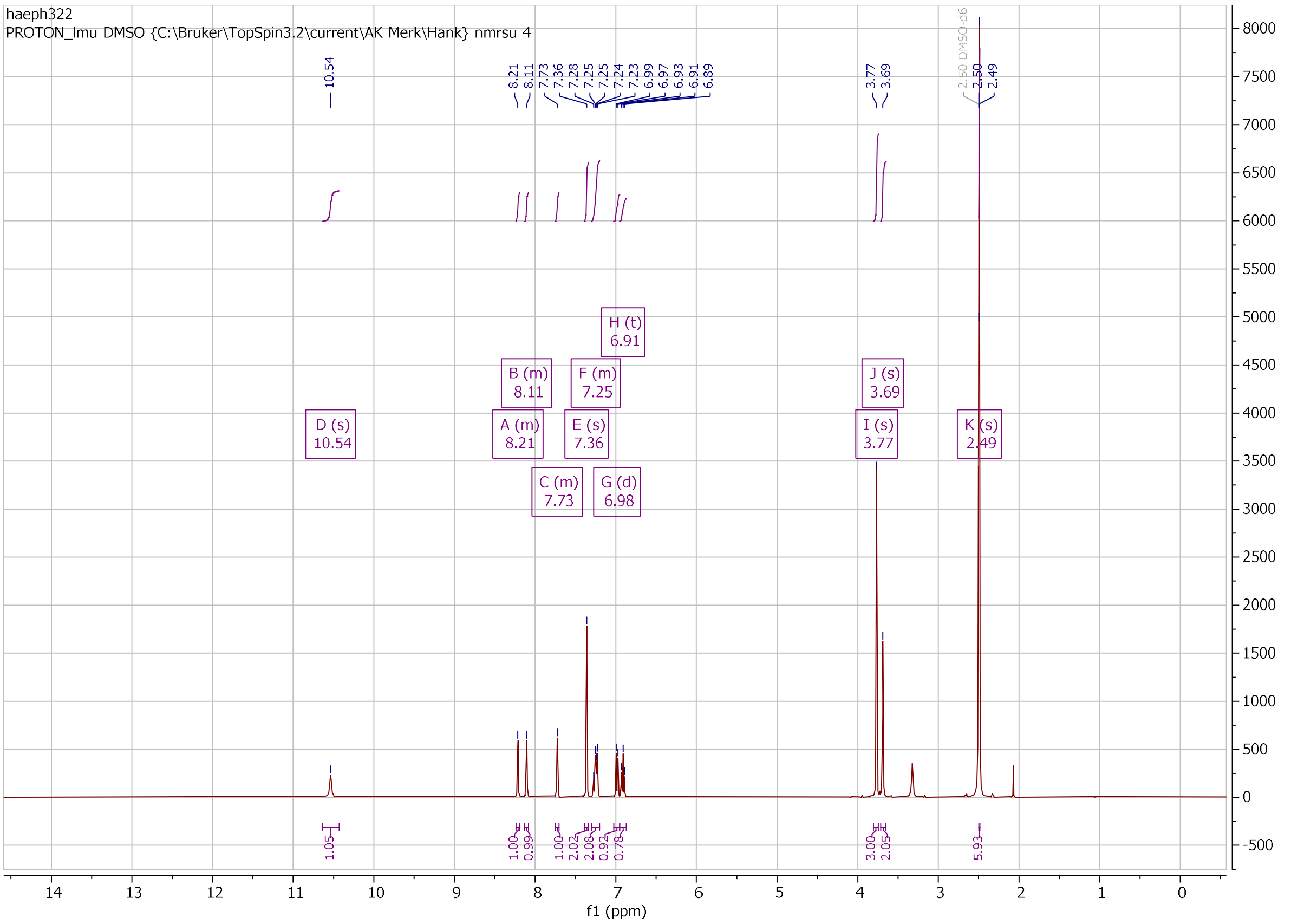
haeph322
PROTON_lmu DMSO {C:\Bruker\TopSpin3.2\current\AK Merk\Hank} nmrsu 4
10.54
8.21
8.11
7.73
7.36
7.28
7.25
7.25
7.24
7.23
6.99
6.97
6.93
6.91
6.89
3.77
3.69
2.50 DMSO-d6
2.50
2.49
H (t) 6.91
B (m) 8.11
F (m) 7.25
J (s) 3.69
D (s) 10.54
A (m) 8.21
E (s) 7.36
I (s) 3.77
K (s) 2.49
C (m) 7.73
G (d) 6.98
1.05
1.00
0.99
1.00
2.02
2.08
0.92
0.78
3.00
2.05
5.93
f1 (ppm)

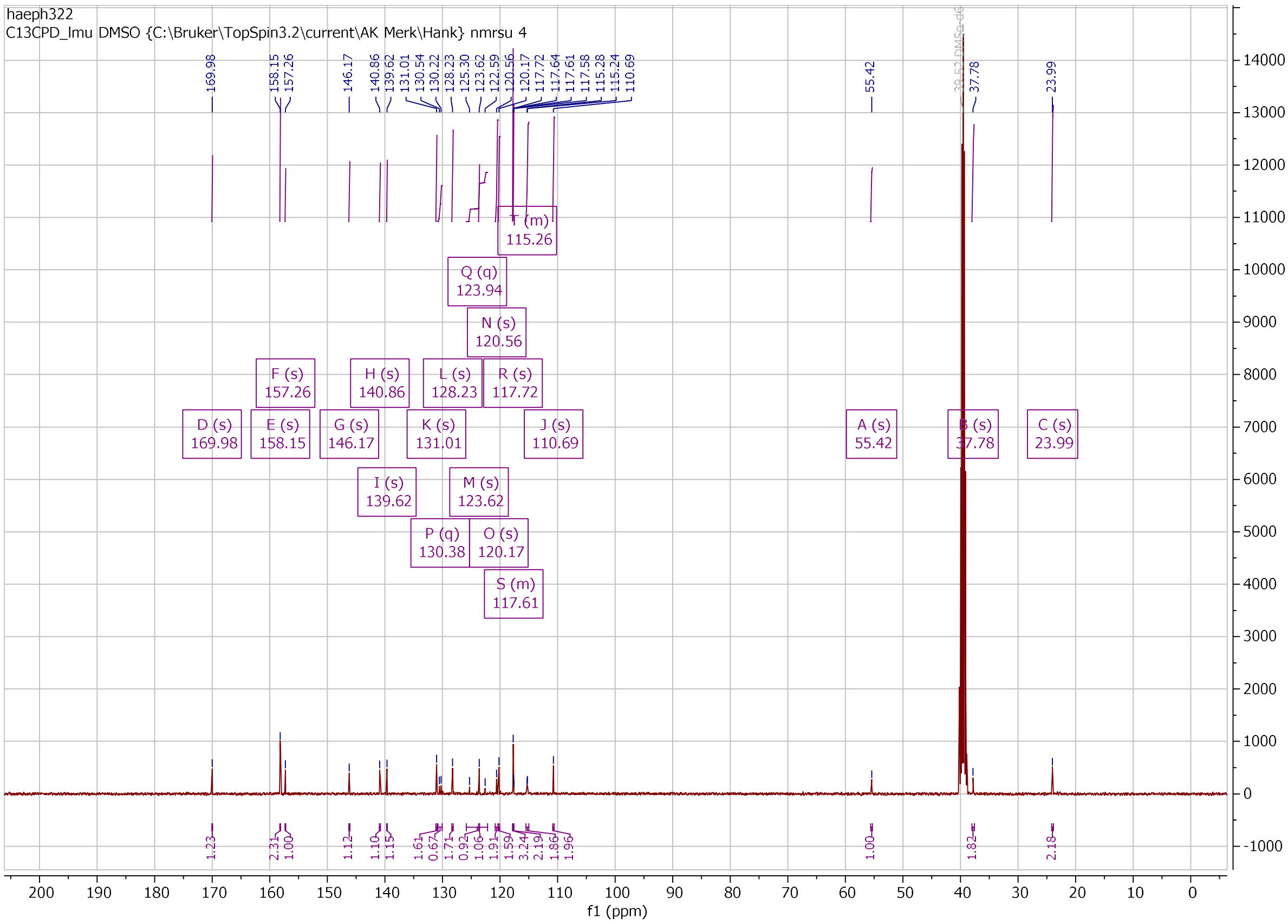

haeph322
C13CPD_lmu DMSO {C:\Bruker\TopSpin3.2\current\AK Merk\Hank} nmrsu 4
169.98
158.15
157.26
146.17
140.86
139.62
131.01
130.54
130.22
128.23
125.30
123.62
122.59
120.56
120.17
117.72
117.64
117.61
117.58
115.28
115.24
110.69
55.42
37.78
23.99
D (s) 169.98
E (s) 158.15
F (s) 157.26
G (s) 146.17
H (s) 140.86
I (s) 139.62
K (s) 131.01
L (s) 128.23
M (s) 123.62
N (s) 120.56
O (s) 120.17
P (q) 130.38
Q (q) 123.94
R (s) 117.72
S (m) 117.61
T (m) 115.26
J (s) 110.69
A (s) 55.42
B (s) 37.78
C (s) 23.99
1.23
2.31
1.00
1.12
1.10
1.15
1.61
0.67
1.71
0.93
1.06
1.91
1.59
3.24
2.19
1.86
1.96
1.00
1.82
2.18
f1 (ppm)

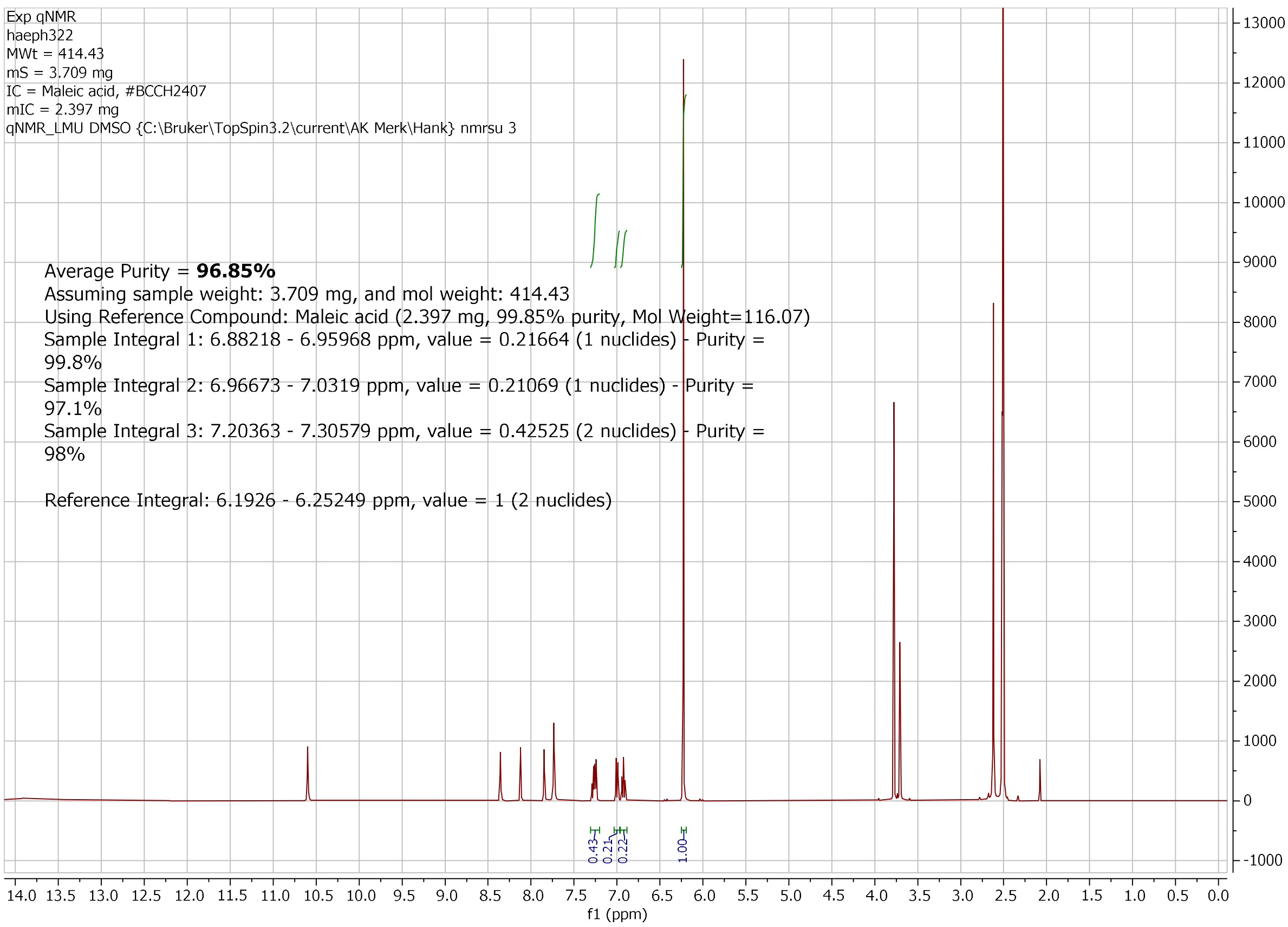

Exp qNMR
haeph322
MWt = 414.43
mS = 3.709 mg
IC = Maleic acid, #BCCH2407
mIC = 2.397 mg
qNMR_LMU DMSO {C:\Bruker\TopSpin3.2\current\AK Merk\Hank} nmrsu 3
Average Purity = **96.85%**
Assuming sample weight: 3.709 mg, and mol weight: 414.43
Using Reference Compound: Maleic acid (2.397 mg, 99.85% purity, Mol Weight=116.07)
Sample Integral 1: 6.88218 - 6.95968 ppm, value = 0.21664 (1 nuclides) - Purity = 99.8%
Sample Integral 2: 6.96673 - 7.0319 ppm, value = 0.21069 (1 nuclides) - Purity = 97.1%
Sample Integral 3: 7.20363 - 7.30579 ppm, value = 0.42525 (2 nuclides) - Purity = 98%
Reference Integral: 6.1926 - 6.25249 ppm, value = 1 (2 nuclides)
0.43
0.21
0.22
1.00
f1 (ppm)

Compound **46**:

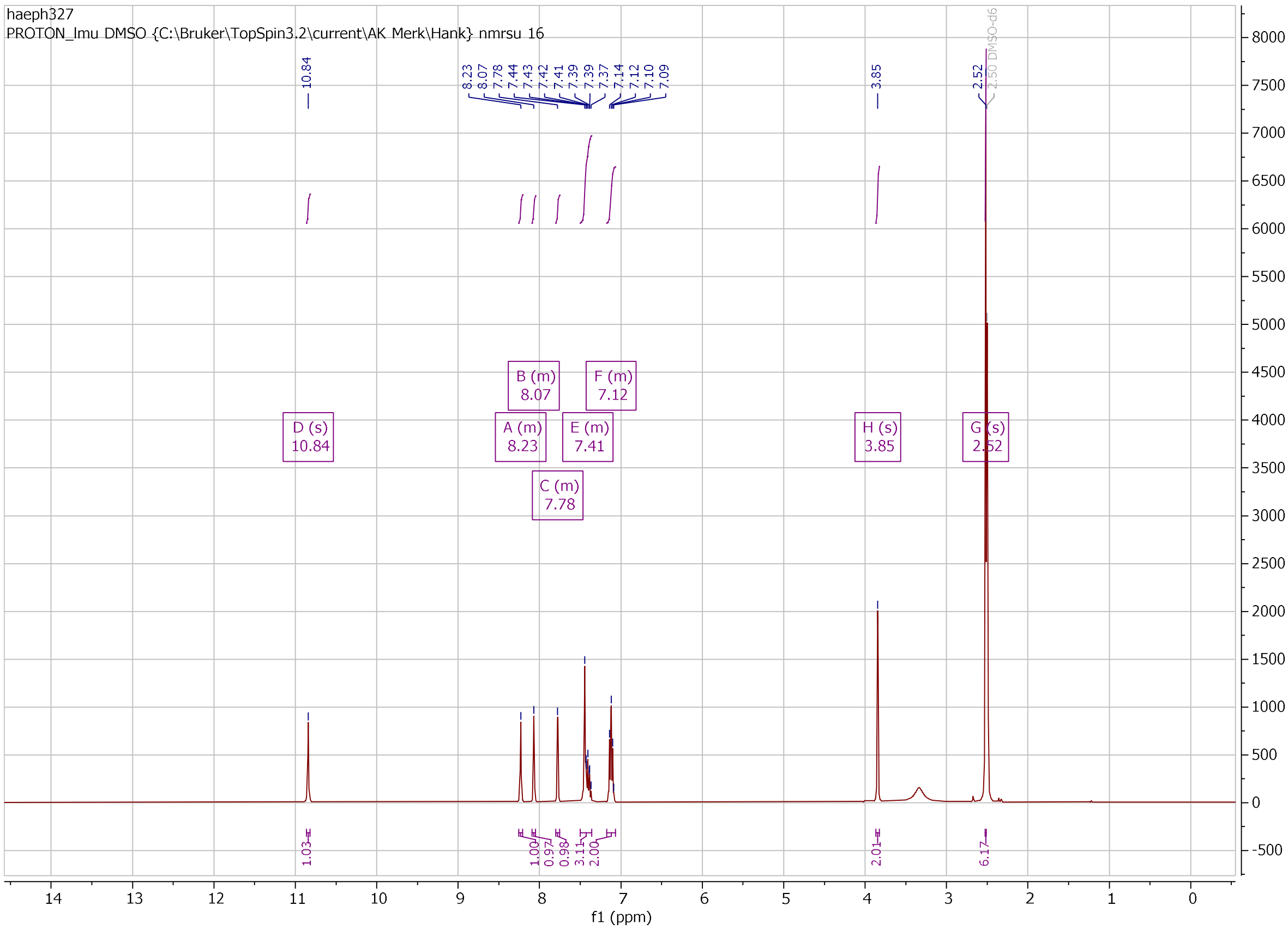

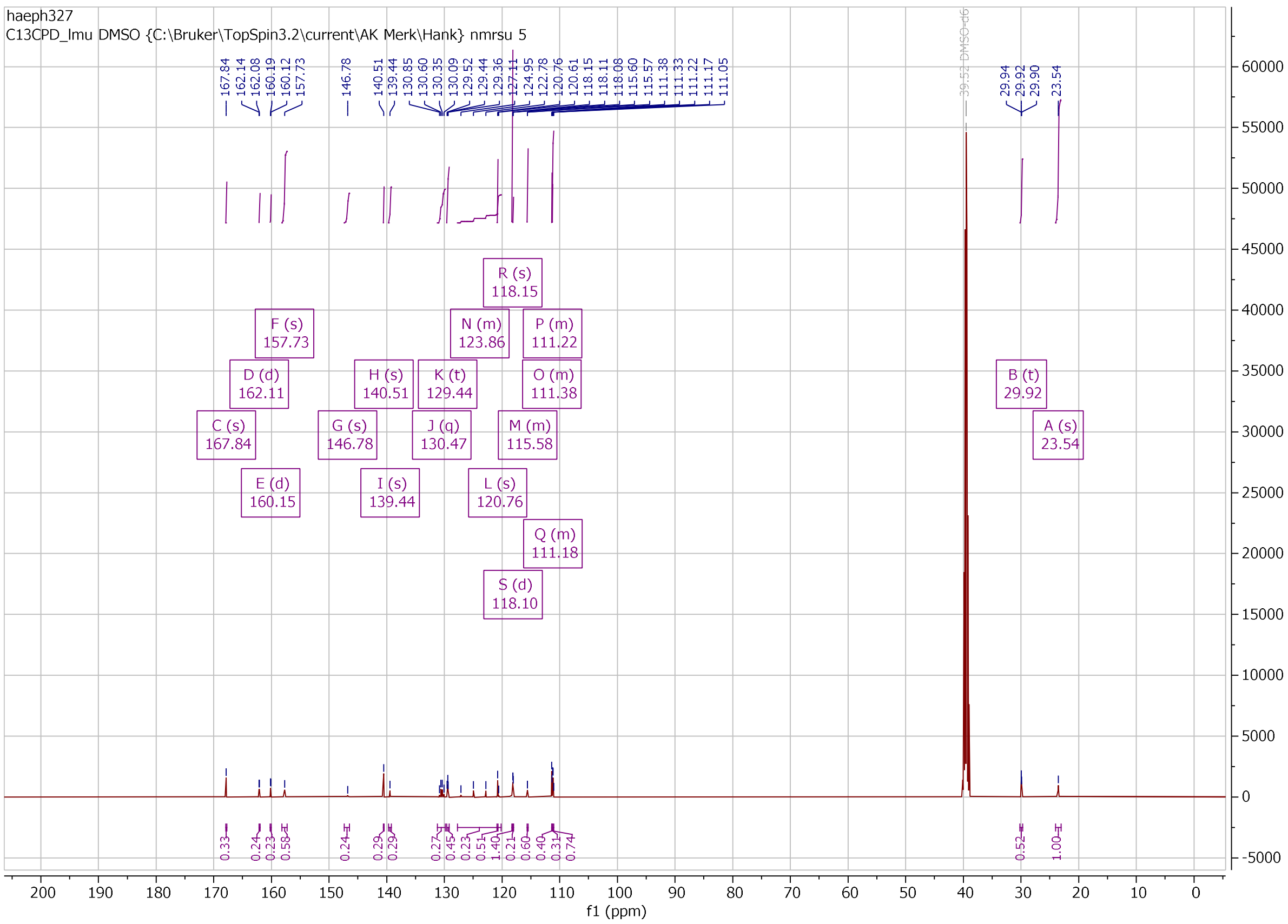

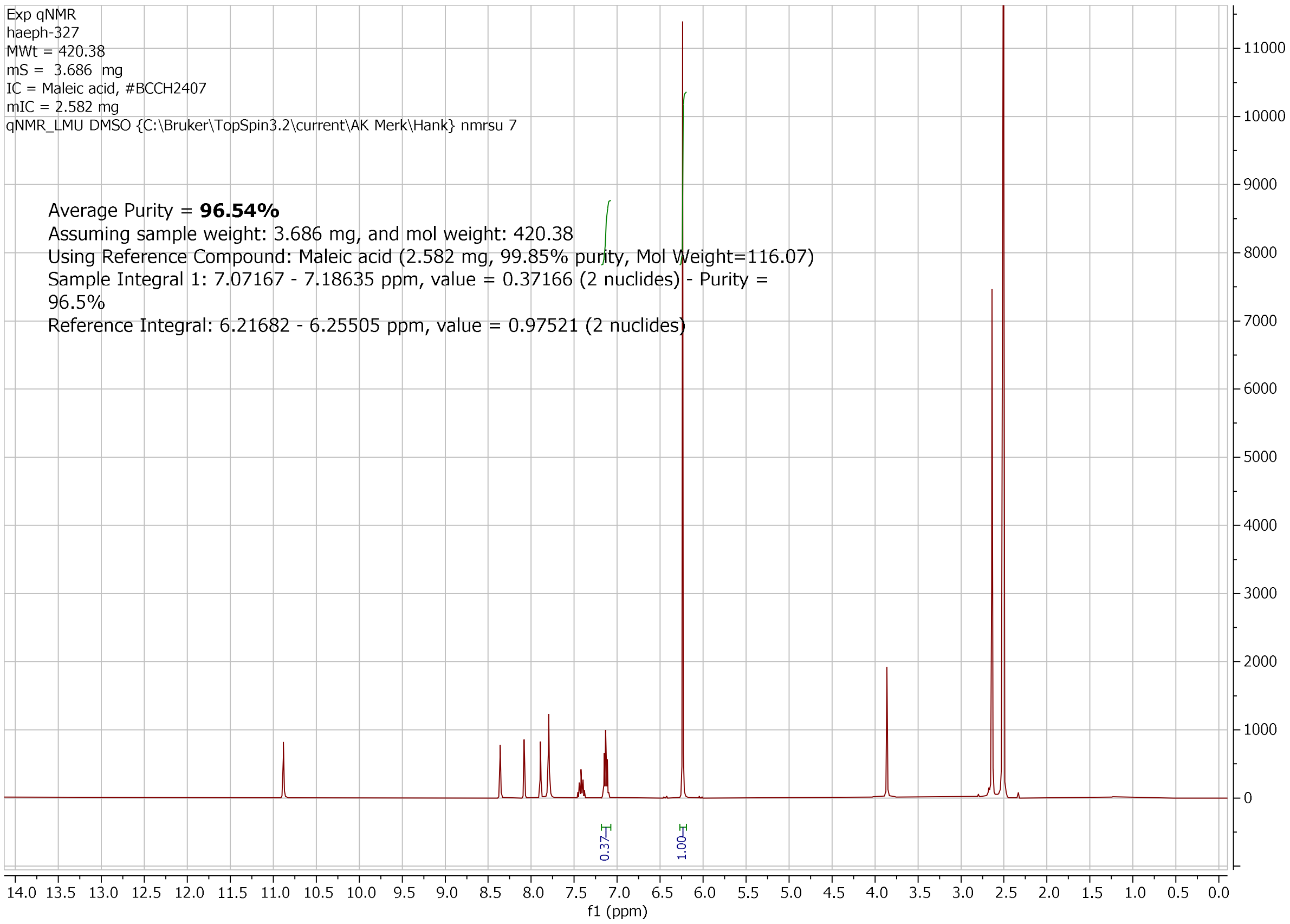
Exp qNMR
haeph-327
MWt = 420.38
mS = 3.686 mg
IC = Maleic acid, #BCCH2407
mIC = 2.582 mg
qNMR_LMU DMSO {C:\Bruker\TopSpin3.2\current\AK Merk\Hank} nmrsu 7
Average Purity = 96.54%
Assuming sample weight: 3.686 mg, and mol weight: 420.38
Using Reference Compound: Maleic acid (2.582 mg, 99.85% purity, Mol Weight=116.07)
Sample Integral 1: 7.07167 - 7.18635 ppm, value = 0.37166 (2 nuclides) - Purity = 96.5%
Reference Integral: 6.21682 - 6.25505 ppm, value = 0.97521 (2 nuclides)
0.37
1.00
f1 (ppm)

Compound **47**:

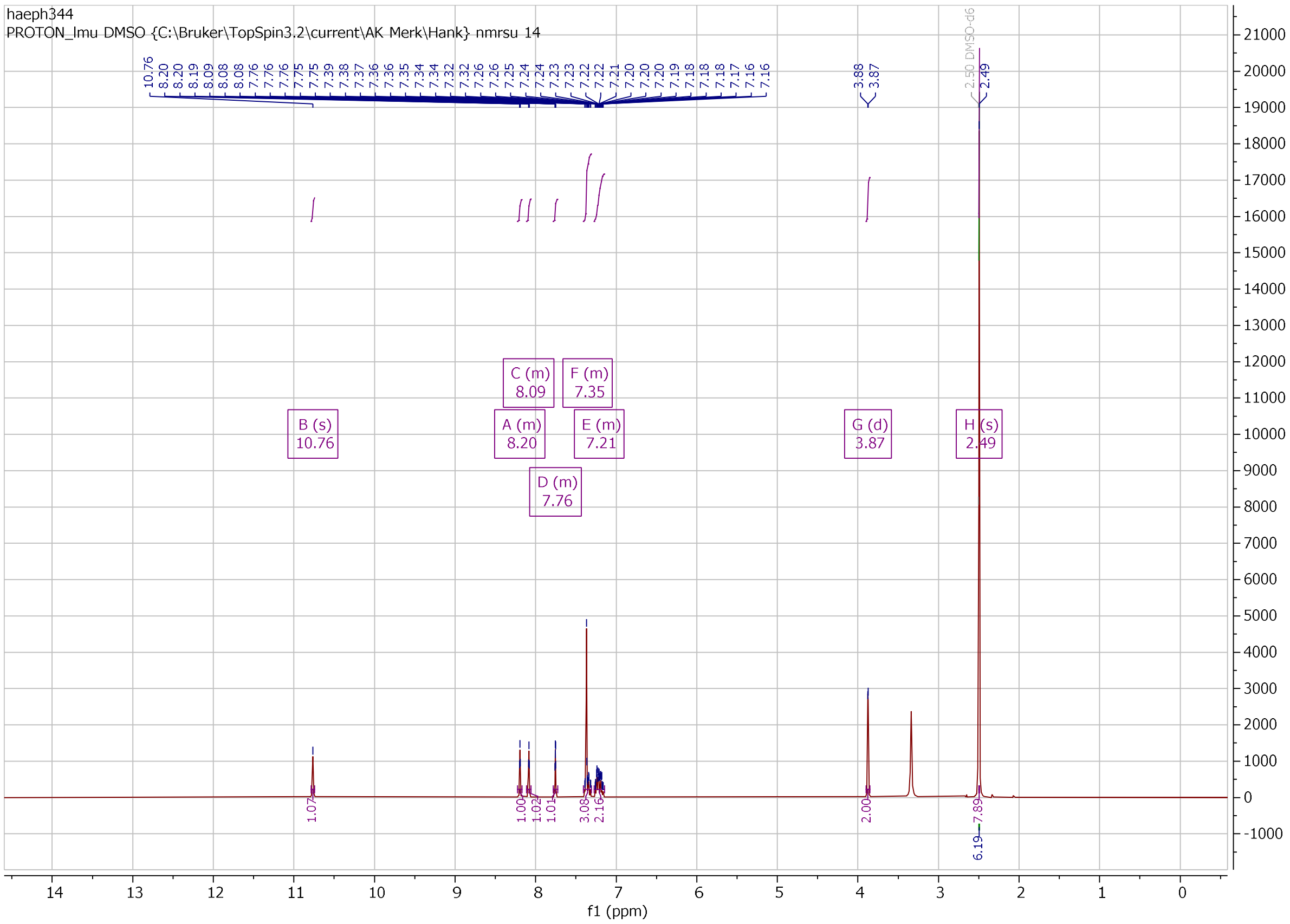
haeph344
PROTON_lmu DMSO {C:\Bruker\TopSpin3.2\current\AK Merk\Hank} nmrsu 14
B (s) 10.76
C (m) 8.09
A (m) 8.20
D (m) 7.76
F (m) 7.35
E (m) 7.21
G (d) 3.87
H (s) 2.49
2.50 DMSO-d6
f1 (ppm)

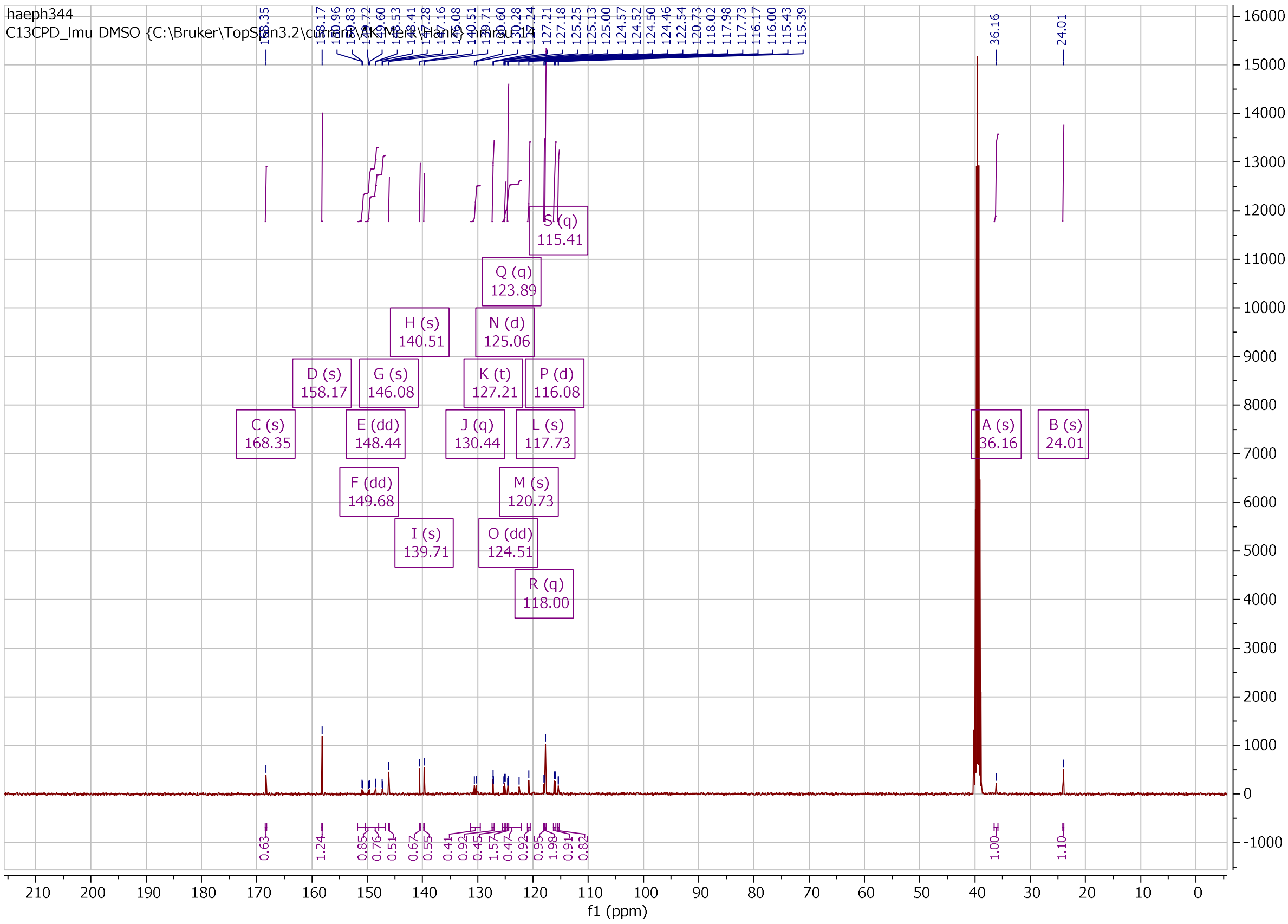
haeph344
C13CPD_lmu DMSO {C:\Bruker\TopSpin3.2\current\AK Merk\Hank} nmrsu 14
C (s) 168.35
D (s) 158.17
E (dd) 148.44
F (dd) 149.68
G (s) 146.08
H (s) 140.51
I (s) 139.71
J (q) 130.44
K (t) 127.21
L (s) 117.73
M (s) 120.73
N (d) 125.06
O (dd) 124.51
P (d) 116.08
Q (q) 123.89
R (q) 118.00
S (q) 115.41
A (s) 36.16
B (s) 24.01
f1 (ppm)

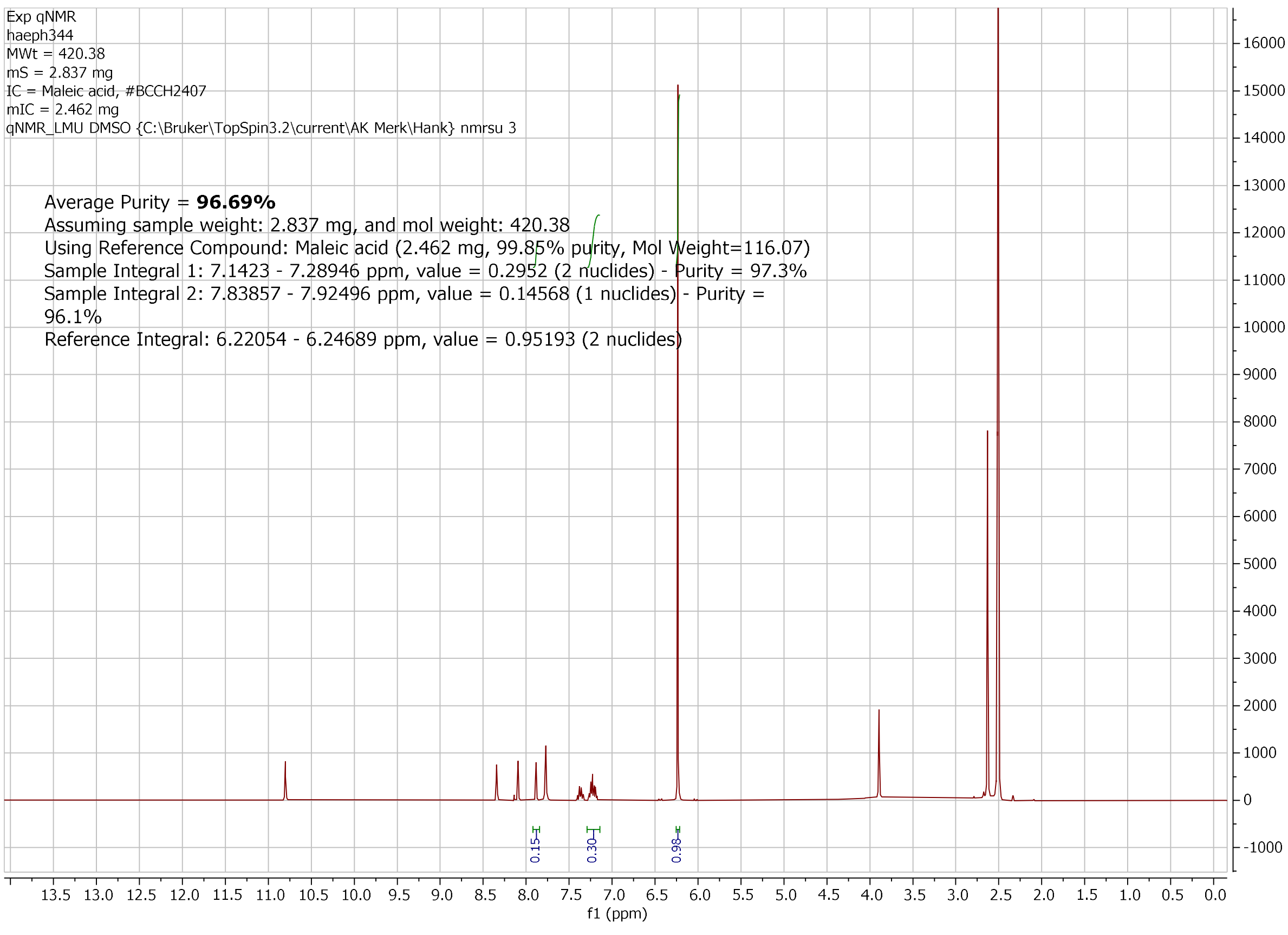
Exp qNMR
haeph344
MWt = 420.38
mS = 2.837 mg
IC = Maleic acid, #BCCH2407
mIC = 2.462 mg
qNMR_LMU DMSO {C:\Bruker\TopSpin3.2\current\AK Merk\Hank} nmrsu 3
Average Purity = 96.69%
Assuming sample weight: 2.837 mg, and mol weight: 420.38
Using Reference Compound: Maleic acid (2.462 mg, 99.85% purity, Mol Weight=116.07)
Sample Integral 1: 7.1423 - 7.28946 ppm, value = 0.2952 (2 nuclides) - Purity = 97.3%
Sample Integral 2: 7.83857 - 7.92496 ppm, value = 0.14568 (1 nuclides) - Purity = 96.1%
Reference Integral: 6.22054 - 6.24689 ppm, value = 0.95193 (2 nuclides)
f1 (ppm)

Compound **48**:

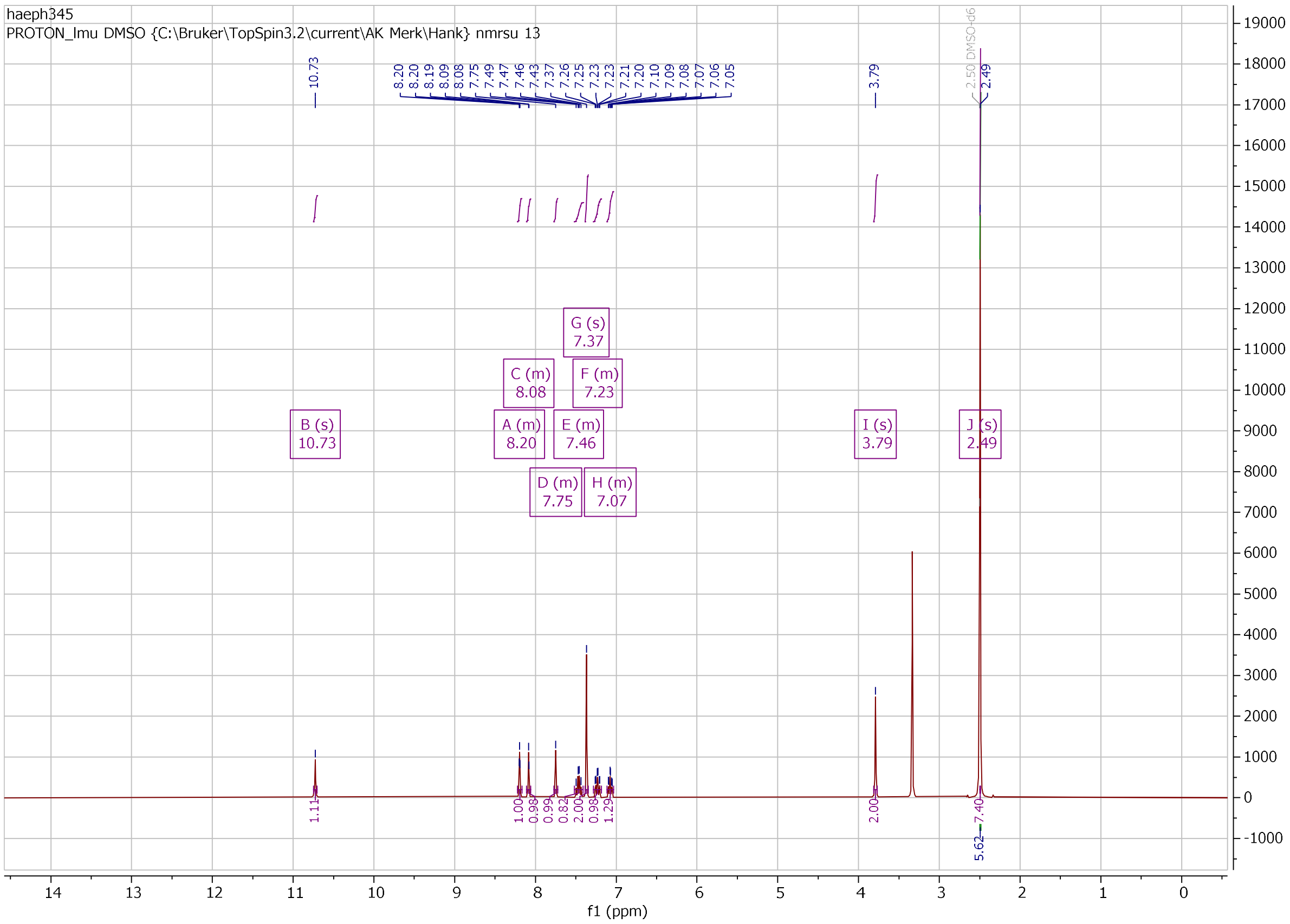

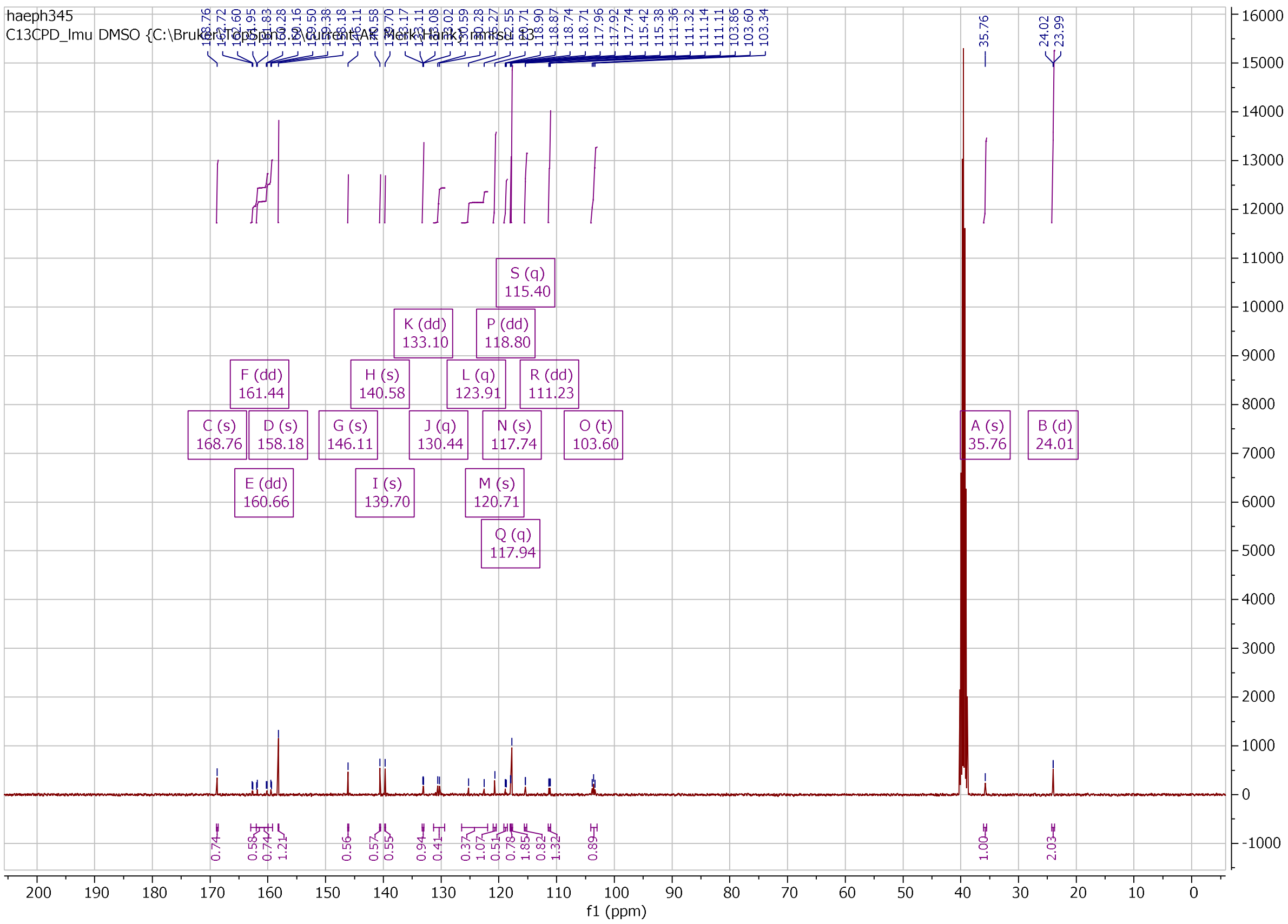

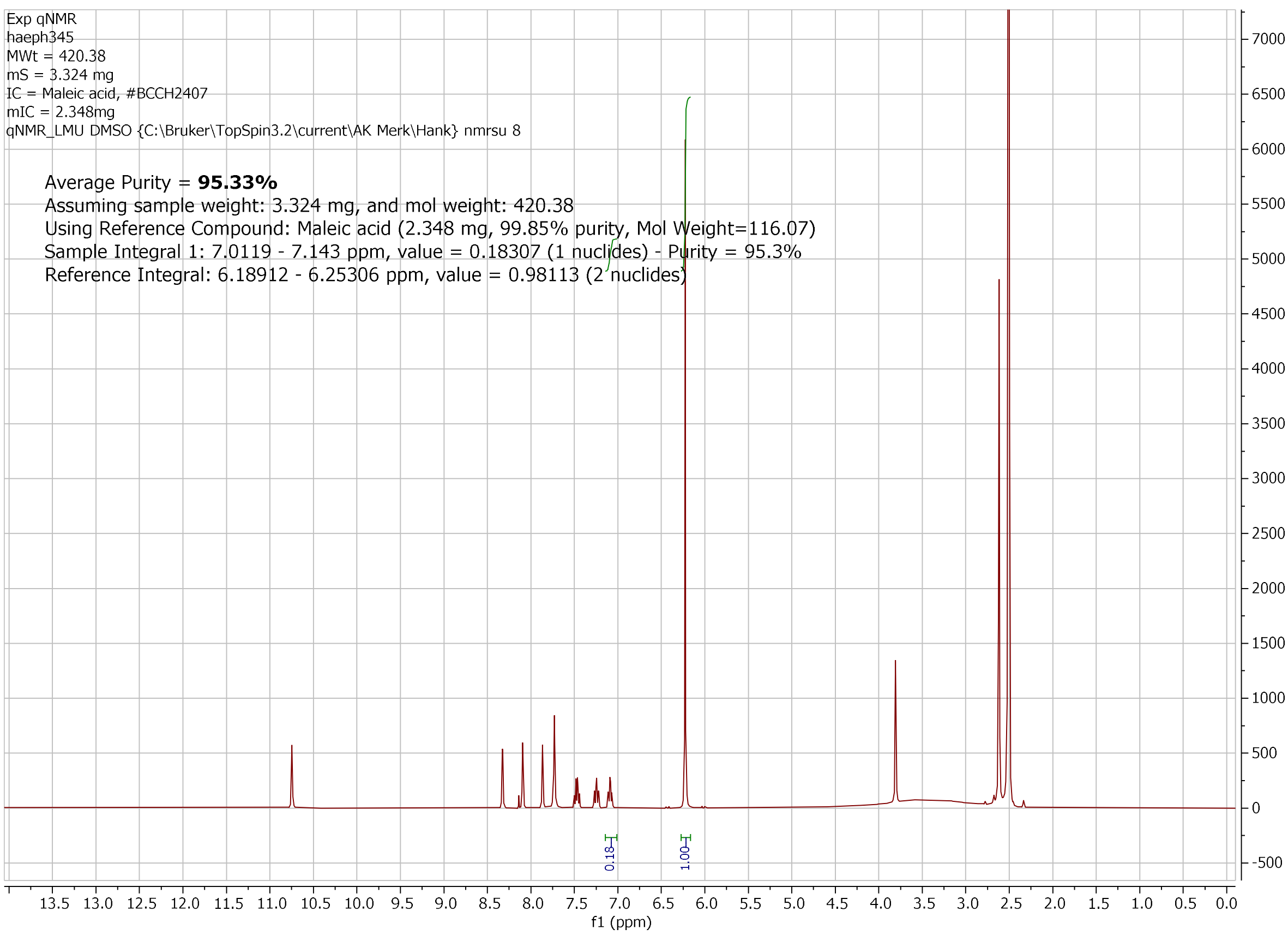
Exp qNMR
haeph345
MWt = 420.38
mS = 3.324 mg
IC = Maleic acid, #BCCH2407
mIC = 2.348mg
qNMR_LMU DMSO {C:\Bruker\TopSpin3.2\current\AK Merk\Hank} nmrsu 8
Average Purity = **95.33%**
Assuming sample weight: 3.324 mg, and mol weight: 420.38
Using Reference Compound: Maleic acid (2.348 mg, 99.85% purity, Mol Weight=116.07)
Sample Integral 1: 7.0119 - 7.143 ppm, value = 0.18307 (1 nuclides) - Purity = 95.3%
Reference Integral: 6.18912 - 6.25306 ppm, value = 0.98113 (2 nuclides)
f1 (ppm)

Compound **49**:

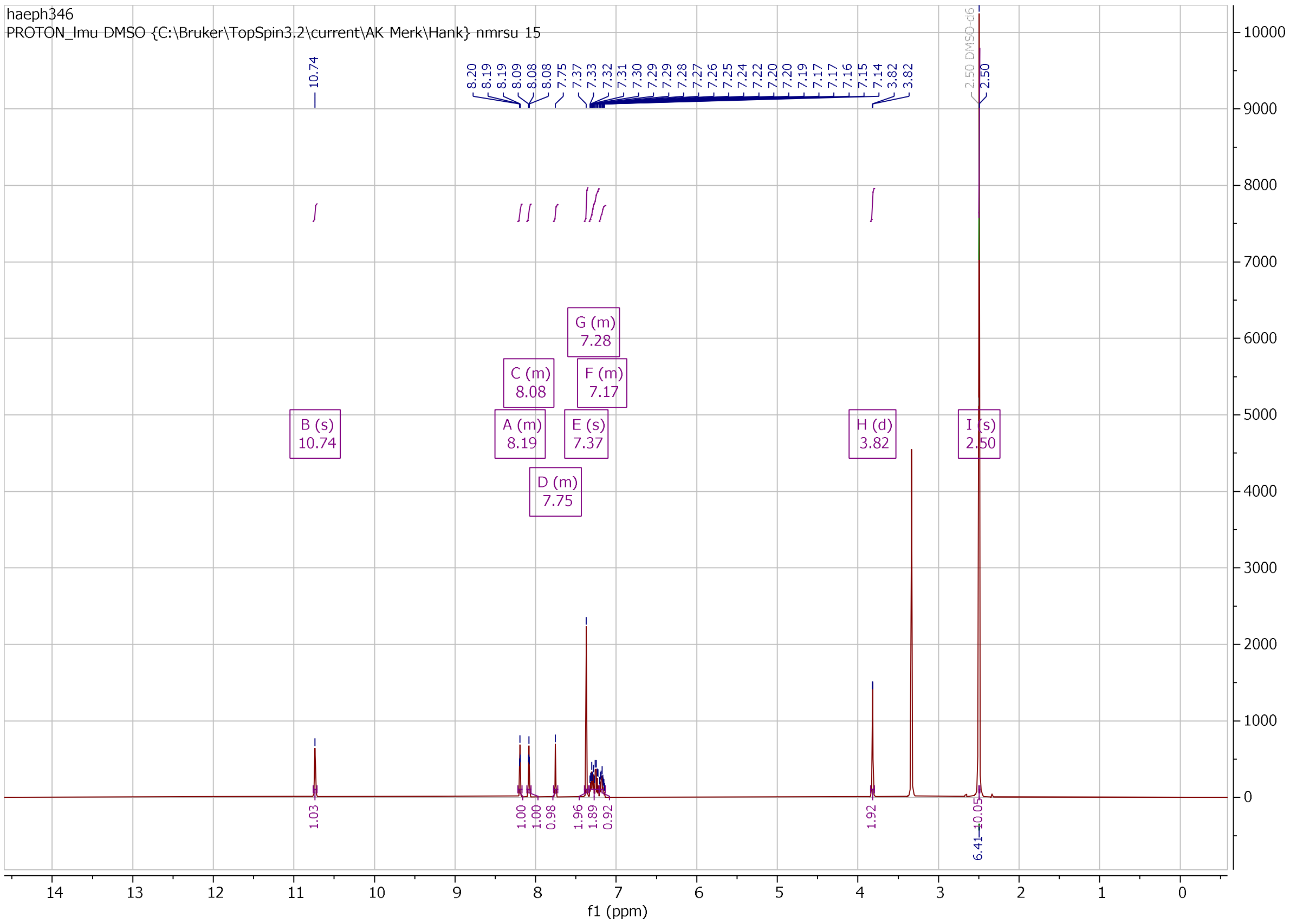
haeph346
PROTON_lmu DMSO {C:\Bruker\TopSpin3.2\current\AK Merk\Hank} nmrsu 15
B (s) 10.74
A (m) 8.19
C (m) 8.08
D (m) 7.75
E (s) 7.37
G (m) 7.28
F (m) 7.17
H (d) 3.82
I (s) 2.50
f1 (ppm)

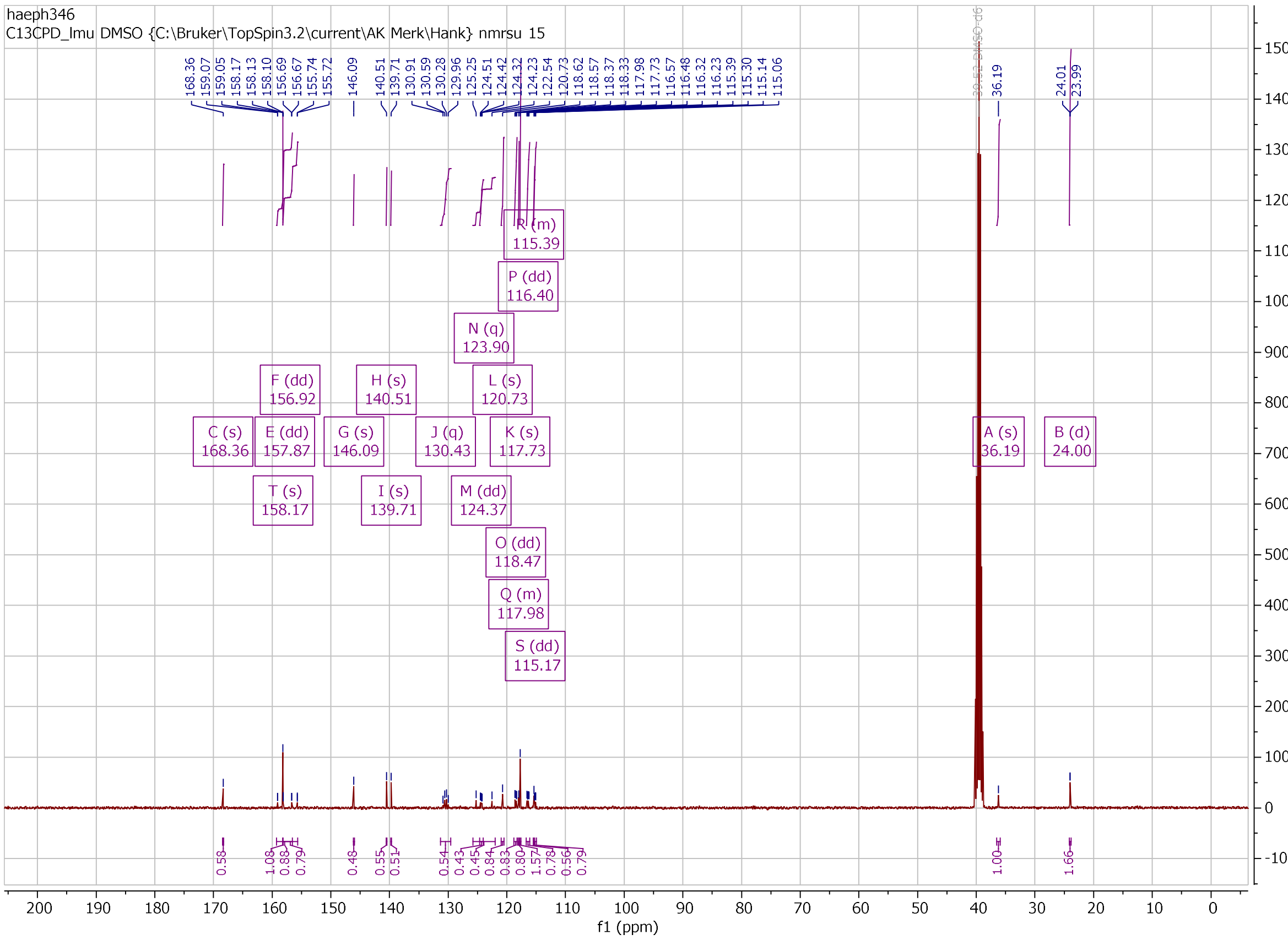
haeph346
C13CPD_lmu DMSO {C:\Bruker\TopSpin3.2\current\AK Merk\Hank} nmrsu 15
168.36 159.07 159.05 158.17 158.13 158.10 156.69 156.67 155.74 155.72 146.09 140.51 139.71 130.91 130.59 130.28 129.96 125.25 124.51 124.42 124.32 124.23 122.54 120.73 118.62 118.57 118.37 118.33 117.98 117.73 116.57 116.48 116.32 116.23 115.39 115.30 115.14 115.06
36.19
24.01 23.99
R (m) 115.39
P (dd) 116.40
N (q) 123.90
F (dd) 156.92
H (s) 140.51
L (s) 120.73
C (s) 168.36
E (dd) 157.87
G (s) 146.09
J (q) 130.43
K (s) 117.73
A (s) 36.19
B (d) 24.00
T (s) 158.17
I (s) 139.71
M (dd) 124.37
O (dd) 118.47
Q (m) 117.98
S (dd) 115.17
f1 (ppm)

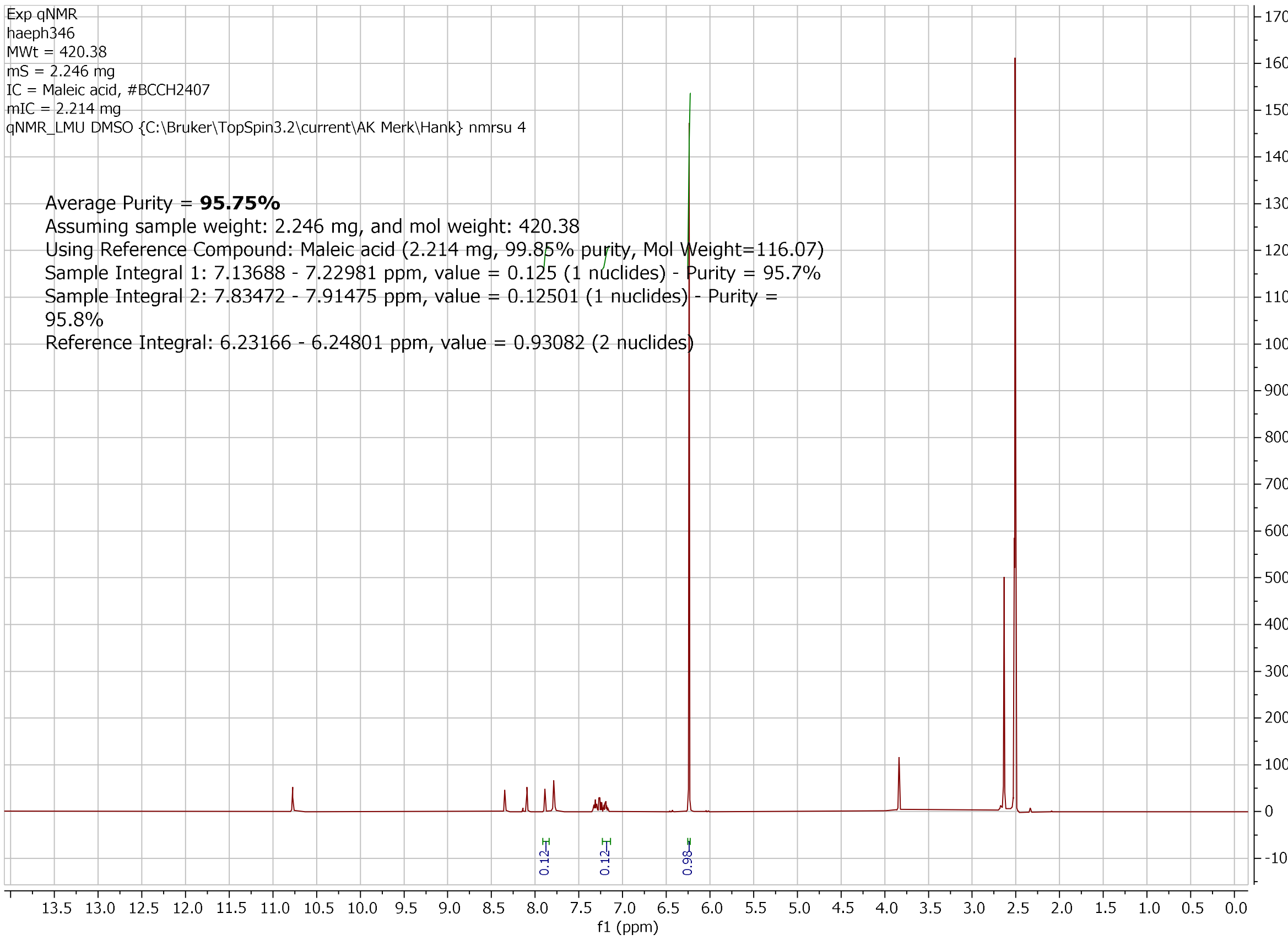
Exp qNMR
haeph346
MWt = 420.38
mS = 2.246 mg
IC = Maleic acid, #BCCH2407
mIC = 2.214 mg
qNMR_LMU DMSO {C:\Bruker\TopSpin3.2\current\AK Merk\Hank} nmrsu 4
Average Purity = **95.75%**
Assuming sample weight: 2.246 mg, and mol weight: 420.38
Using Reference Compound: Maleic acid (2.214 mg, 99.85% purity, Mol Weight=116.07)
Sample Integral 1: 7.13688 - 7.22981 ppm, value = 0.125 (1 nuclides) - Purity = 95.7%
Sample Integral 2: 7.83472 - 7.91475 ppm, value = 0.12501 (1 nuclides) - Purity = 95.8%
Reference Integral: 6.23166 - 6.24801 ppm, value = 0.93082 (2 nuclides)
f1 (ppm)

Compound **50**:

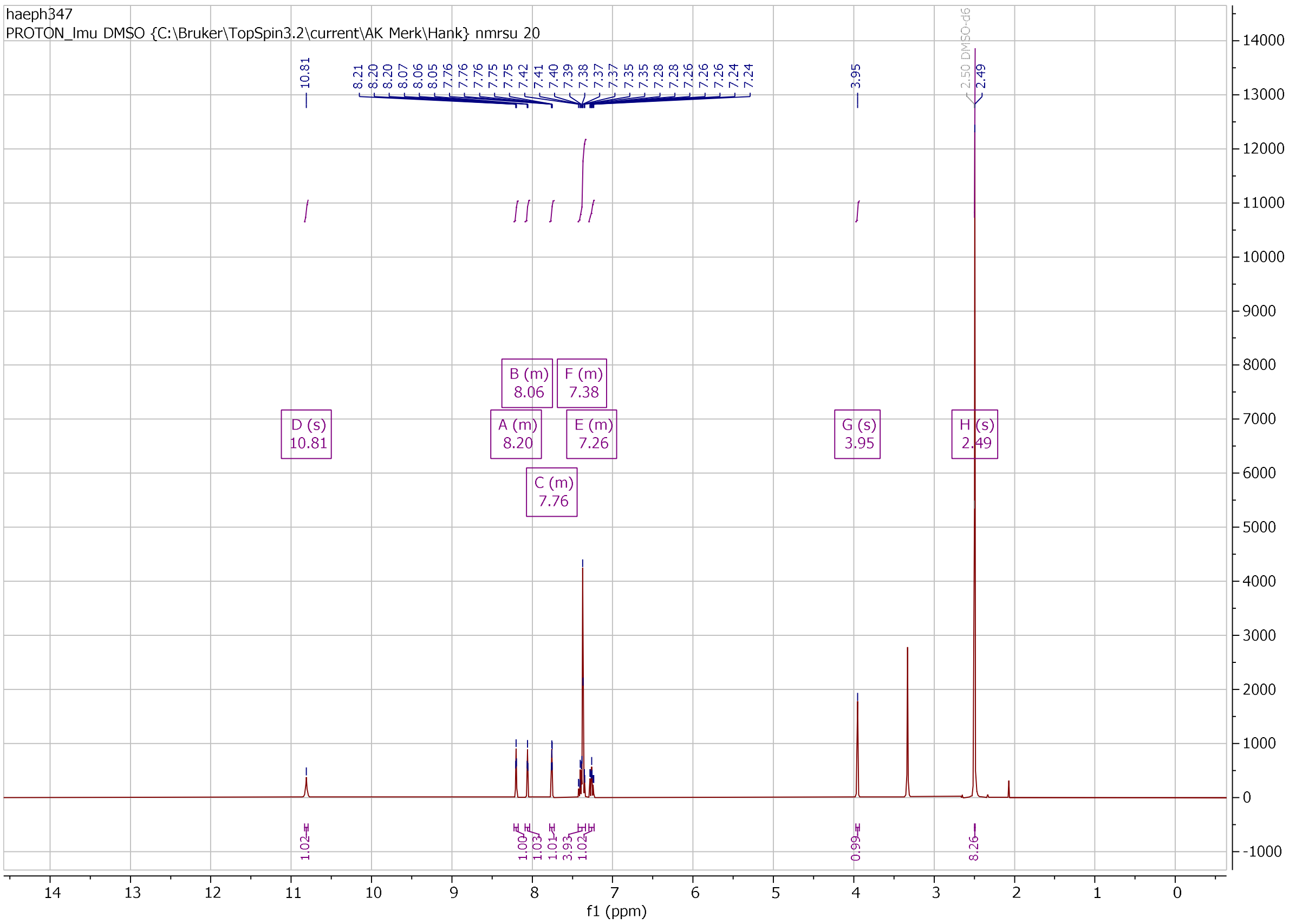
haeph347
PROTON_Imu DMSO {C:\Bruker\TopSpin3.2\current\AK Merk\Hank} nmrsu 20
D (s) 10.81
A (m) 8.20
B (m) 8.06
C (m) 7.76
F (m) 7.38
E (m) 7.26
G (s) 3.95
H (s) 2.49
f1 (ppm)

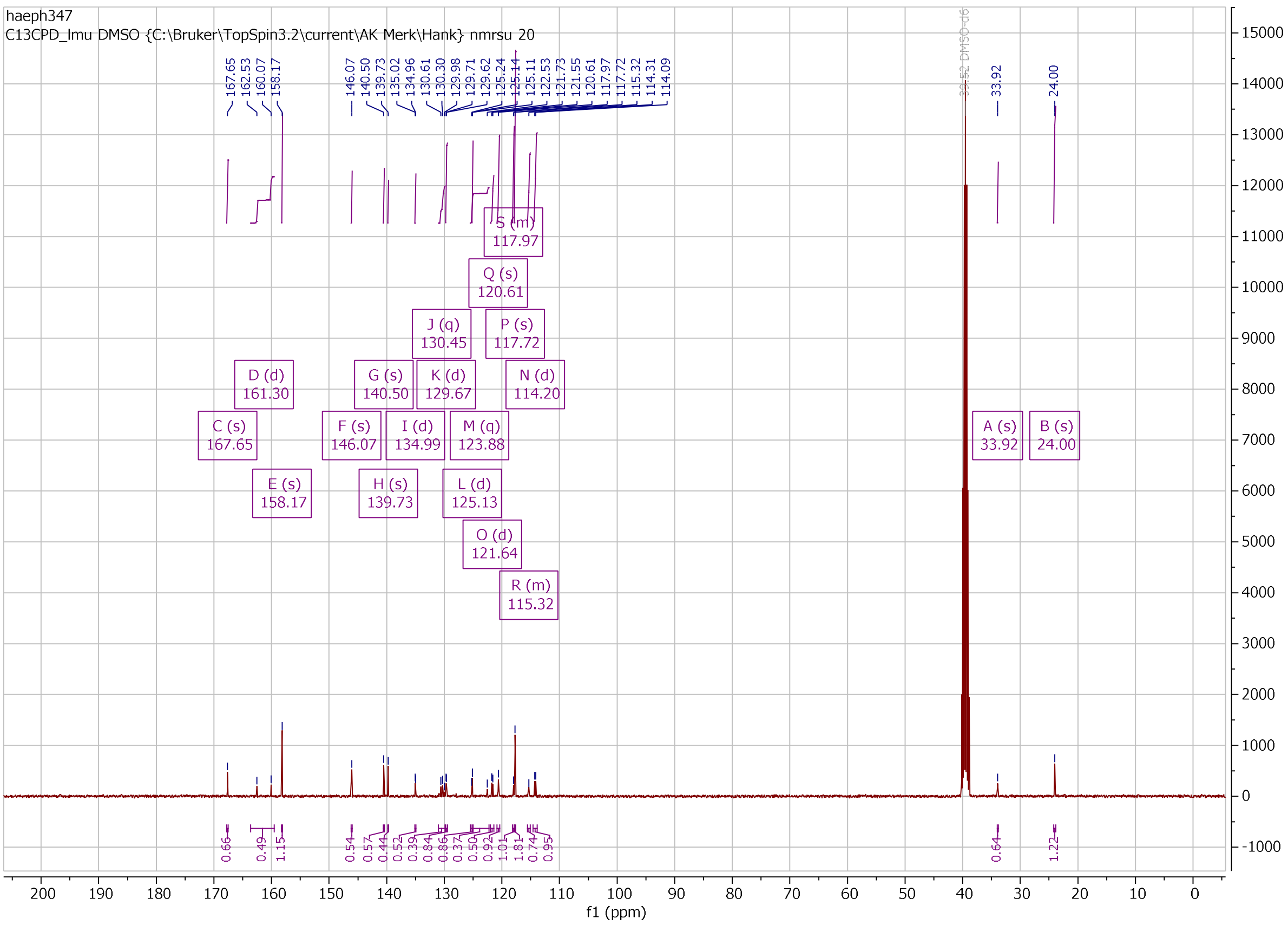
haeph347
C13CPD_Imu DMSO {C:\Bruker\TopSpin3.2\current\AK Merk\Hank} nmrsu 20
C (s) 167.65
D (d) 161.30
E (s) 158.17
F (s) 146.07
G (s) 140.50
H (s) 139.73
I (d) 134.99
J (q) 130.45
K (d) 129.67
L (d) 125.13
M (q) 123.88
O (d) 121.64
Q (s) 120.61
S (m) 117.97
P (s) 117.72
R (m) 115.32
N (d) 114.20
A (s) 33.92
B (s) 24.00
f1 (ppm)

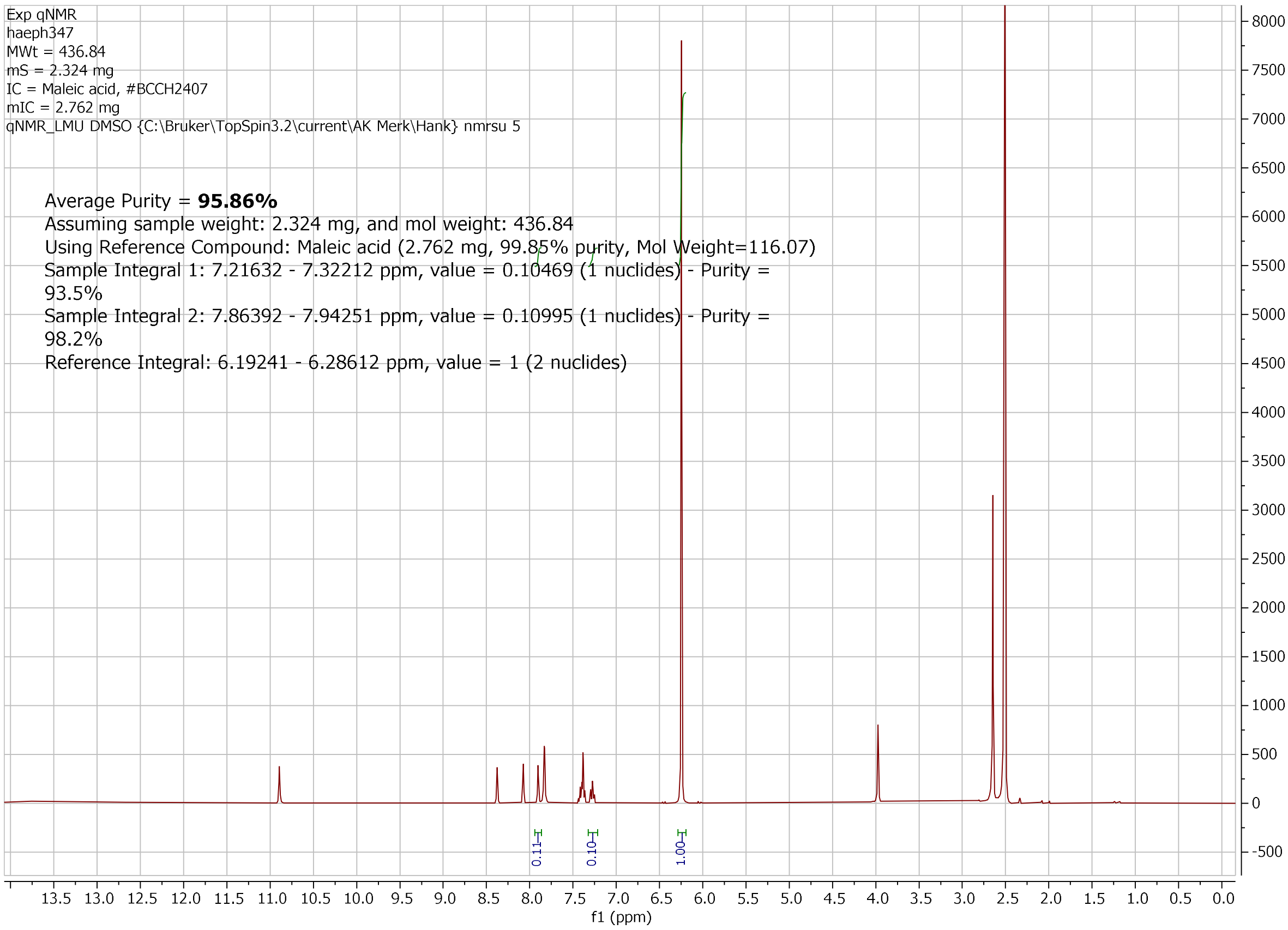
Exp qNMR
haeph347
MWt = 436.84
mS = 2.324 mg
IC = Maleic acid, #BCCH2407
mIC = 2.762 mg
qNMR_LMU DMSO {C:\Bruker\TopSpin3.2\current\AK Merk\Hank} nmrsu 5
Average Purity = **95.86%**
Assuming sample weight: 2.324 mg, and mol weight: 436.84
Using Reference Compound: Maleic acid (2.762 mg, 99.85% purity, Mol Weight=116.07)
Sample Integral 1: 7.21632 - 7.32212 ppm, value = 0.10469 (1 nuclides) - Purity = 93.5%
Sample Integral 2: 7.86392 - 7.94251 ppm, value = 0.10995 (1 nuclides) - Purity = 98.2%
Reference Integral: 6.19241 - 6.28612 ppm, value = 1 (2 nuclides)
f1 (ppm)

Compound **51**:

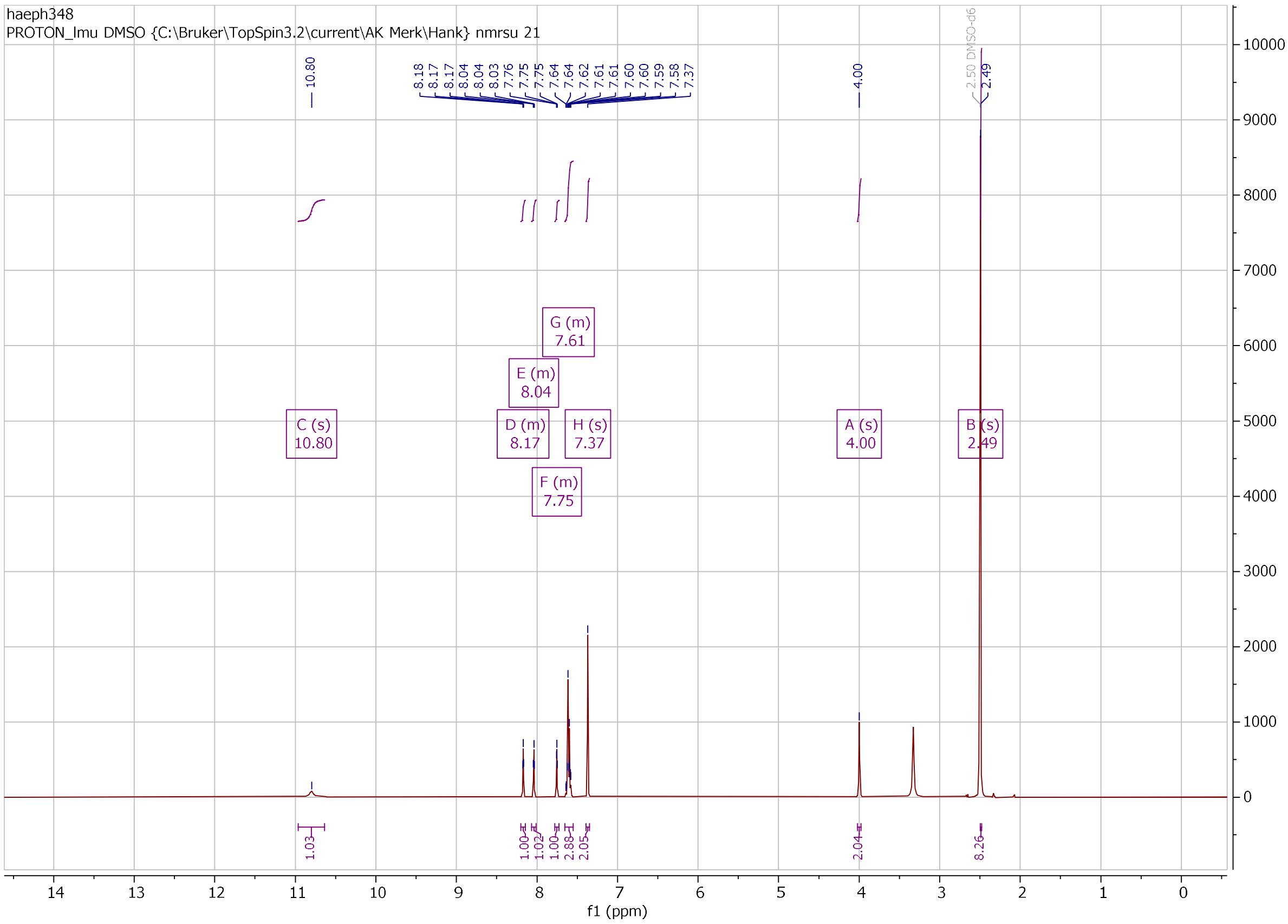
haeph348
PROTON_lmu DMSO {C:\Bruker\TopSpin3.2\current\AK Merk\Hank} nmrsu 21
G (m) 7.61
E (m) 8.04
C (s) 10.80
D (m) 8.17
H (s) 7.37
A (s) 4.00
B (s) 2.49
F (m) 7.75
f1 (ppm)

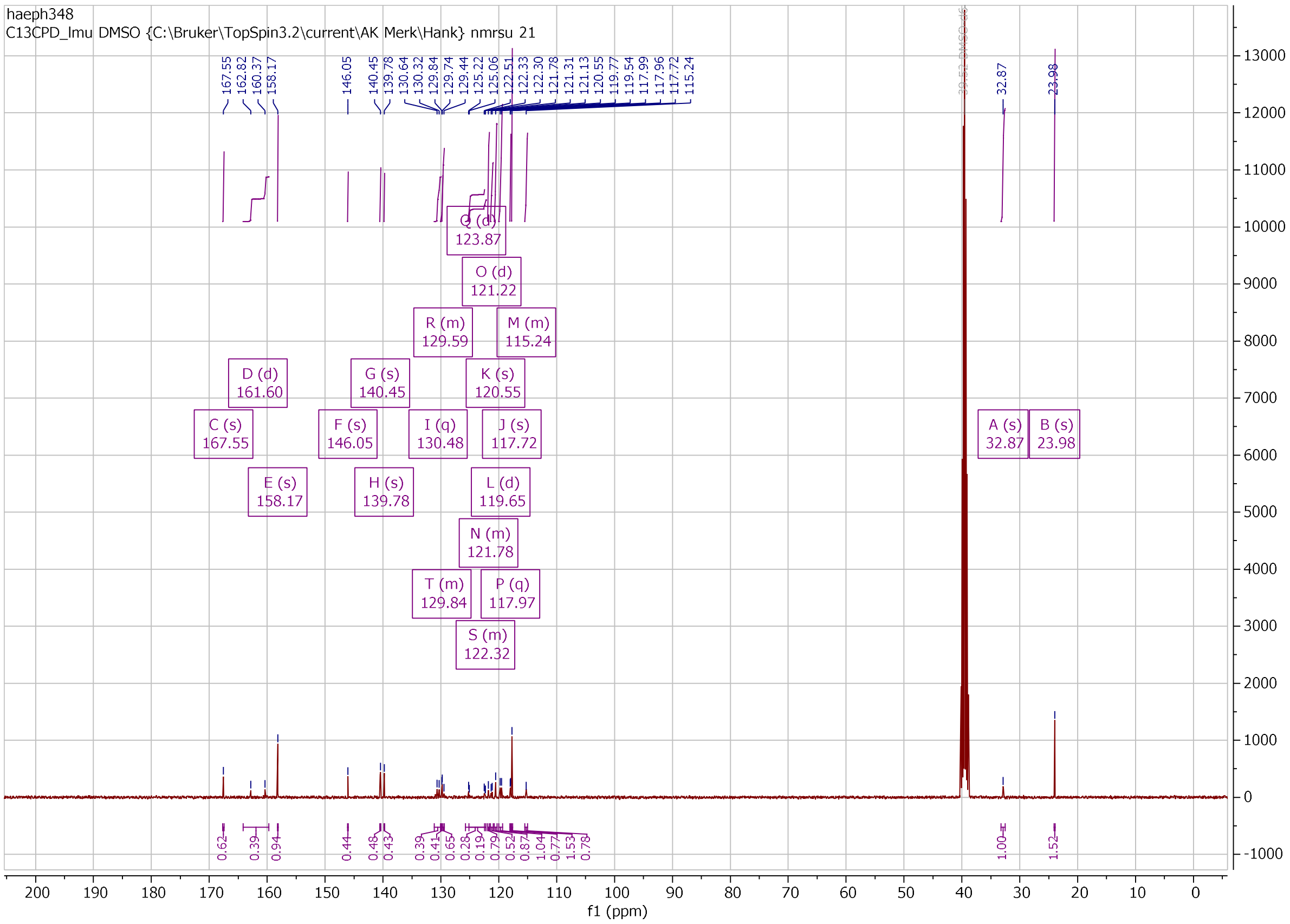
haeph348
C13CPD_lmu DMSO {C:\Bruker\TopSpin3.2\current\AK Merk\Hank} nmrsu 21
167.55
162.82
160.37
158.17
146.05
140.45
139.78
130.64
130.32
129.84
129.74
129.44
125.22
125.06
122.51
122.33
122.30
121.78
121.31
121.13
120.55
119.77
119.54
117.99
117.96
117.72
115.24
32.87
23.98
Q (d) 123.87
O (d) 121.22
R (m) 129.59
M (m) 115.24
D (d) 161.60
G (s) 140.45
K (s) 120.55
C (s) 167.55
F (s) 146.05
I (q) 130.48
J (s) 117.72
A (s) 32.87
B (s) 23.98
E (s) 158.17
H (s) 139.78
L (d) 119.65
N (m) 121.78
T (m) 129.84
P (q) 117.97
S (m) 122.32
f1 (ppm)

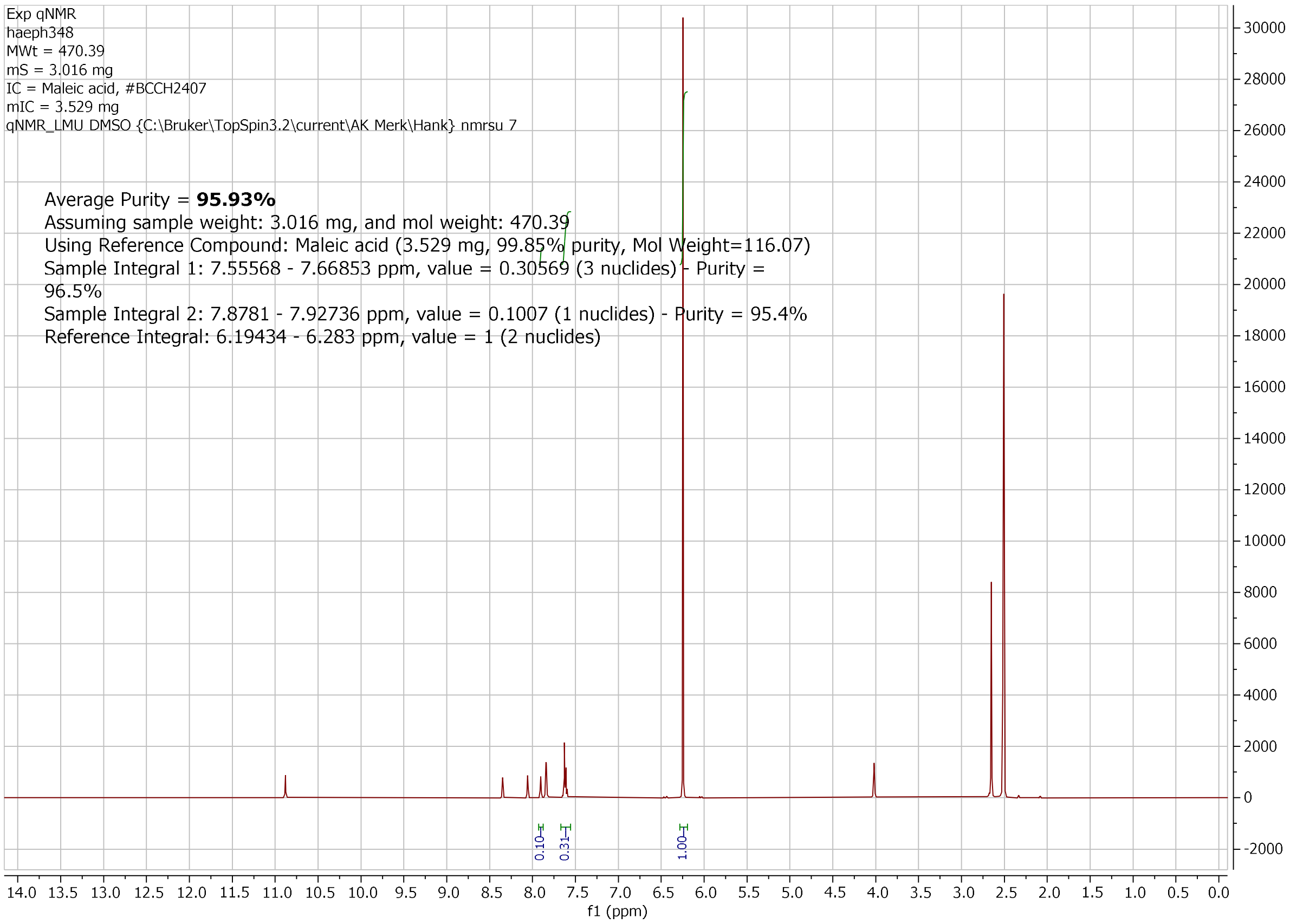
Exp qNMR
haeph348
MWt = 470.39
mS = 3.016 mg
IC = Maleic acid, #BCCH2407
mIC = 3.529 mg
qNMR_LMU DMSO {C:\Bruker\TopSpin3.2\current\AK Merk\Hank} nmrsu 7
Average Purity = **95.93%**
Assuming sample weight: 3.016 mg, and mol weight: 470.39
Using Reference Compound: Maleic acid (3.529 mg, 99.85% purity, Mol Weight=116.07)
Sample Integral 1: 7.55568 - 7.66853 ppm, value = 0.30569 (3 nuclides) - Purity = 96.5%
Sample Integral 2: 7.8781 - 7.92736 ppm, value = 0.1007 (1 nuclides) - Purity = 95.4%
Reference Integral: 6.19434 - 6.283 ppm, value = 1 (2 nuclides)
0.10
0.31
1.00
f1 (ppm)

Compound **52**:

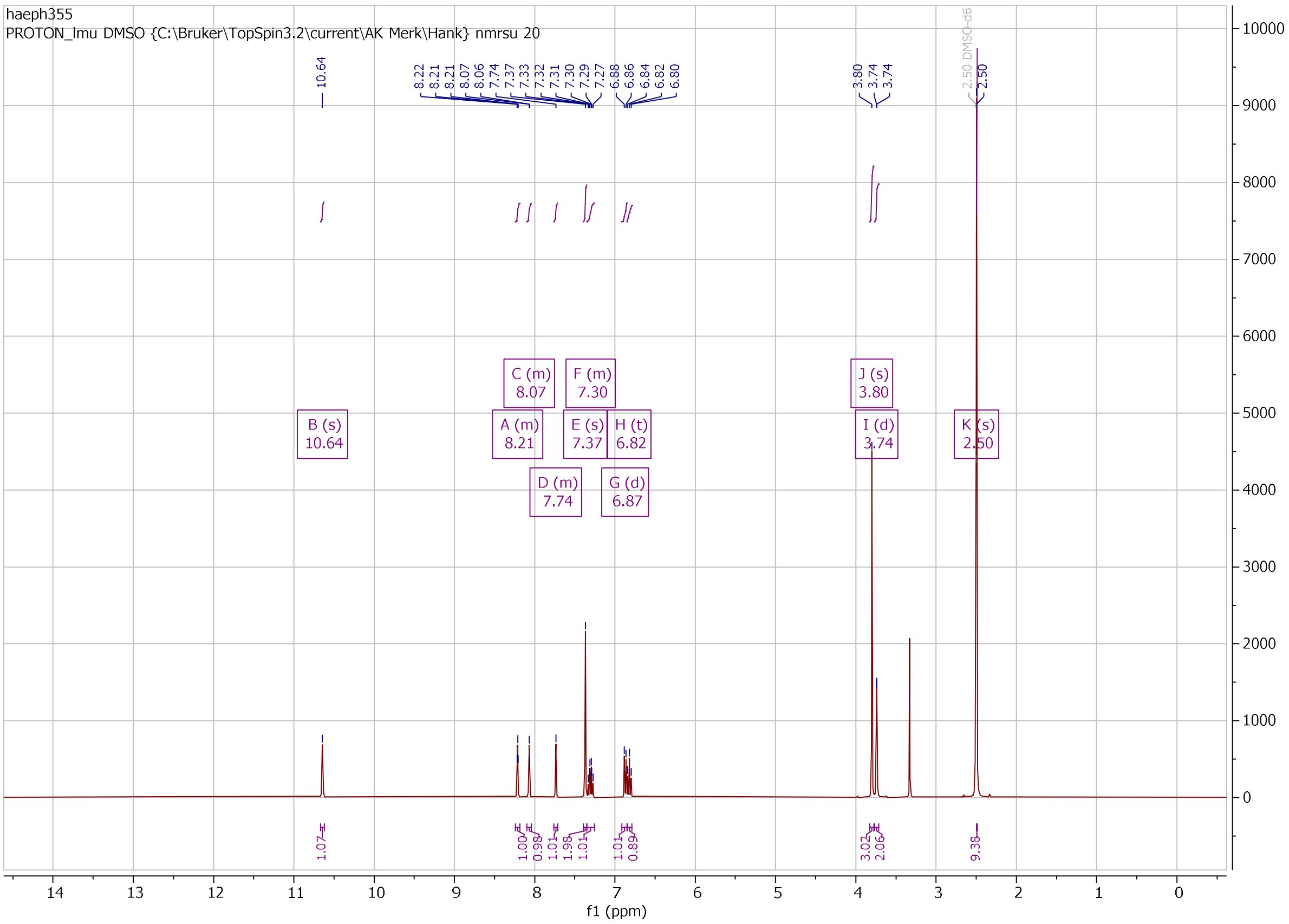

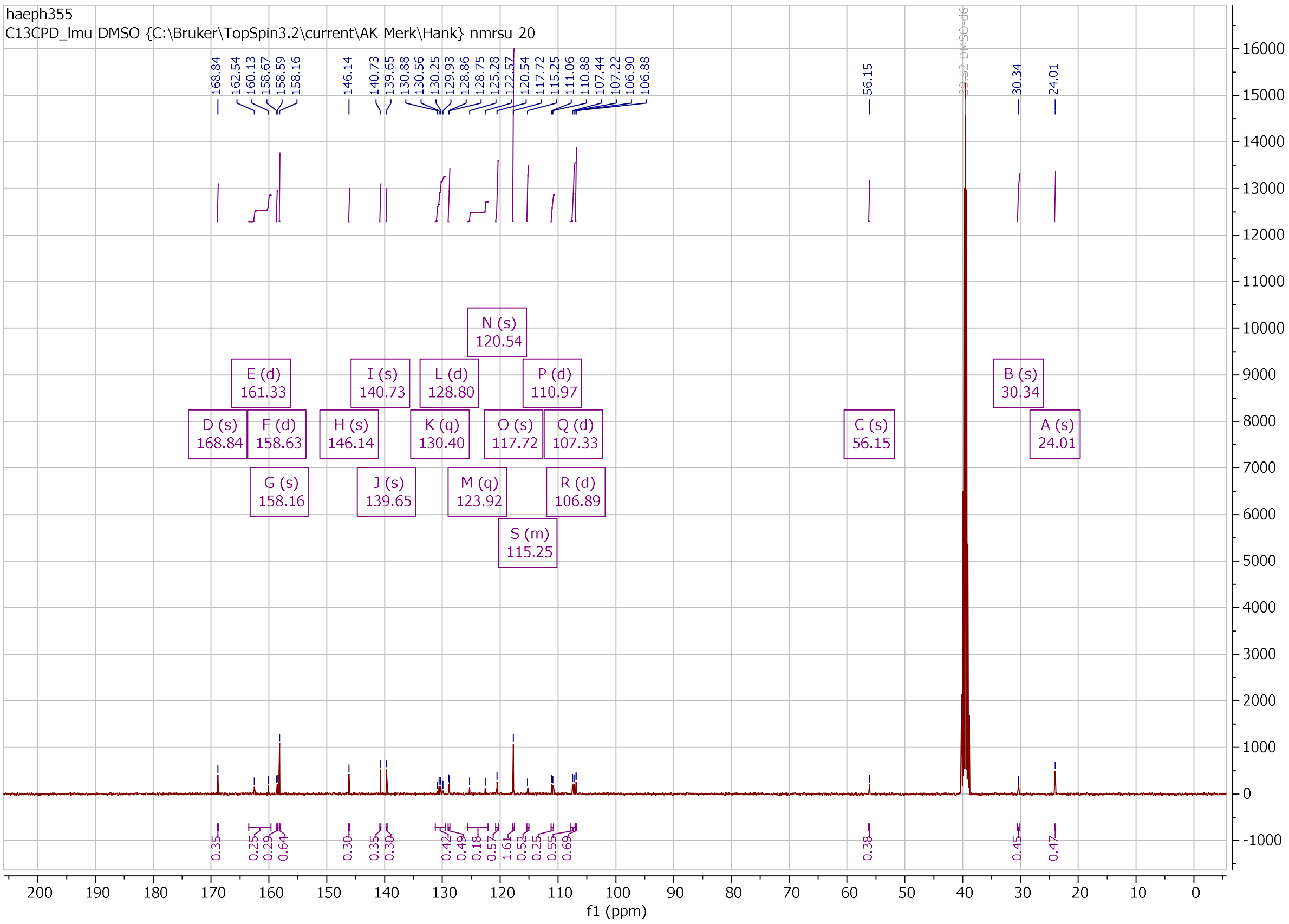

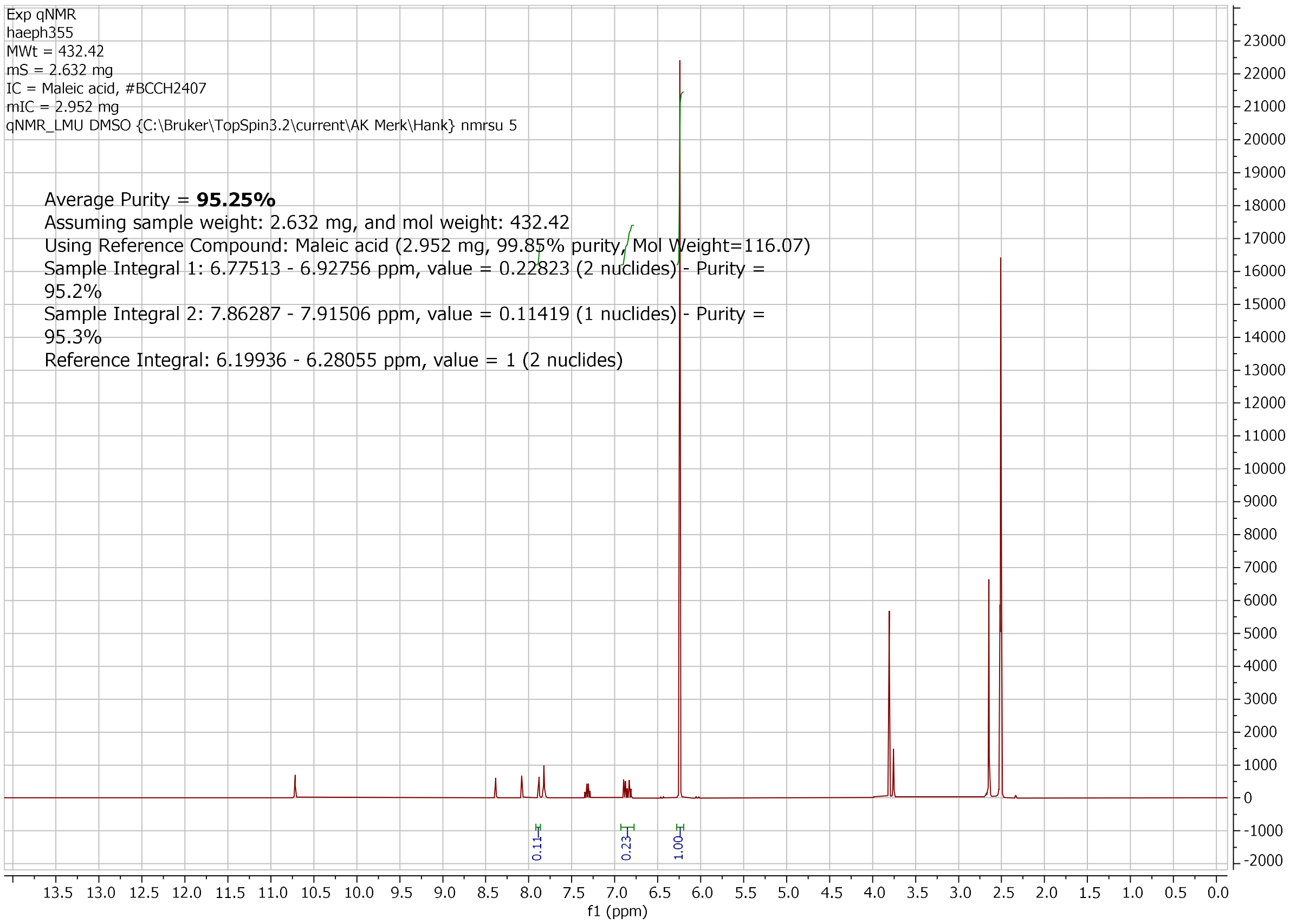
Exp qNMR
haeph355
MWt = 432.42
mS = 2.632 mg
IC = Maleic acid, #BCCH2407
mIC = 2.952 mg
qNMR_LMU DMSO {C:\Bruker\TopSpin3.2\current\AK Merk\Hank} nmrsu 5
Average Purity = 95.25%
Assuming sample weight: 2.632 mg, and mol weight: 432.42
Using Reference Compound: Maleic acid (2.952 mg, 99.85% purity, Mol Weight=116.07)
Sample Integral 1: 6.77513 - 6.92756 ppm, value = 0.22823 (2 nuclides) - Purity = 95.2%
Sample Integral 2: 7.86287 - 7.91506 ppm, value = 0.11419 (1 nuclides) - Purity = 95.3%
Reference Integral: 6.19936 - 6.28055 ppm, value = 1 (2 nuclides)
f1 (ppm)

Compound **53**:

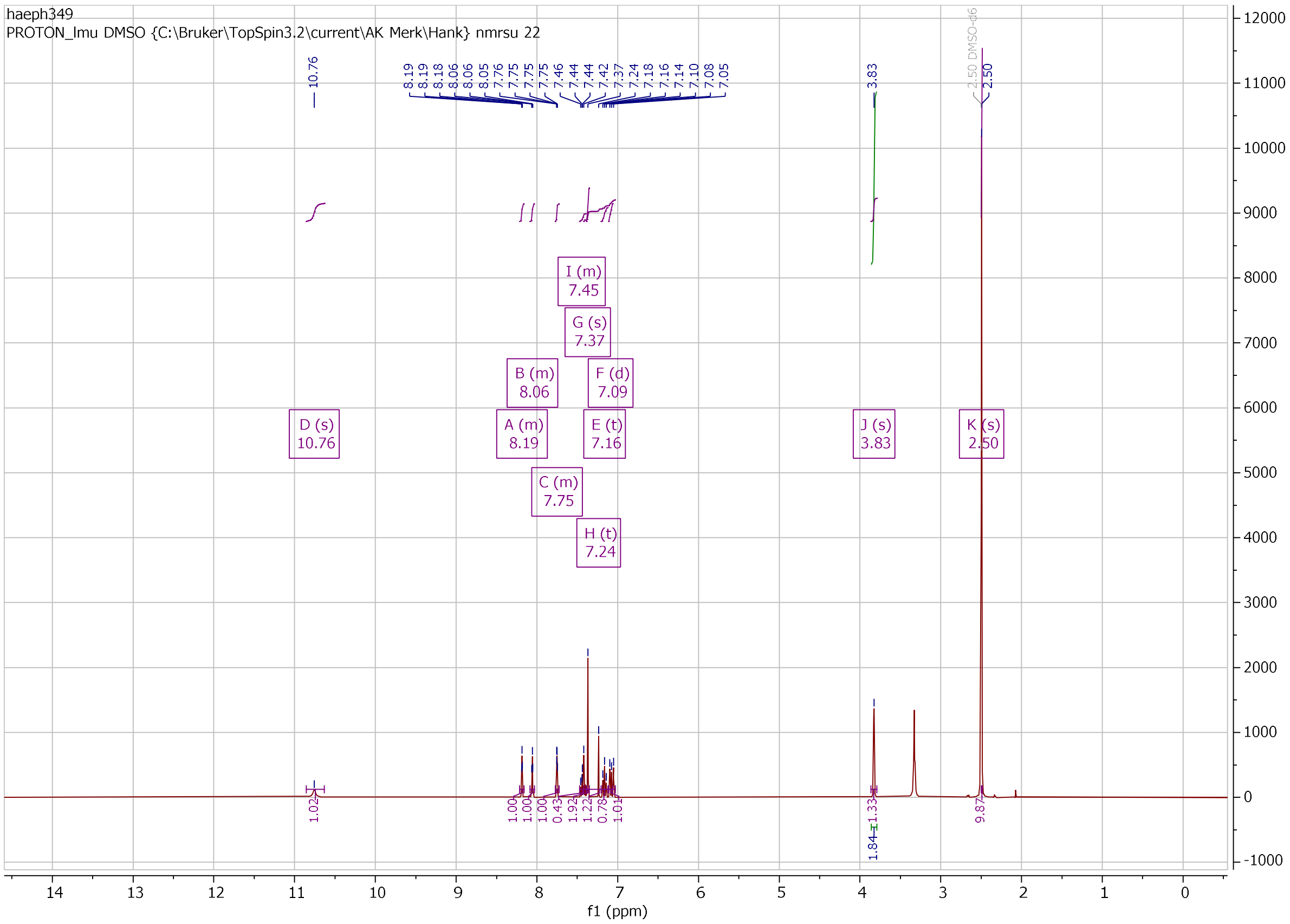
haeph349
PROTON_lmu DMSO {C:\Bruker\TopSpin3.2\current\AK Merk\Hank} nmrsu 22
D (s) 10.76
A (m) 8.19
B (m) 8.06
C (m) 7.75
I (m) 7.45
G (s) 7.37
F (d) 7.09
E (t) 7.16
H (t) 7.24
J (s) 3.83
K (s) 2.50
f1 (ppm)

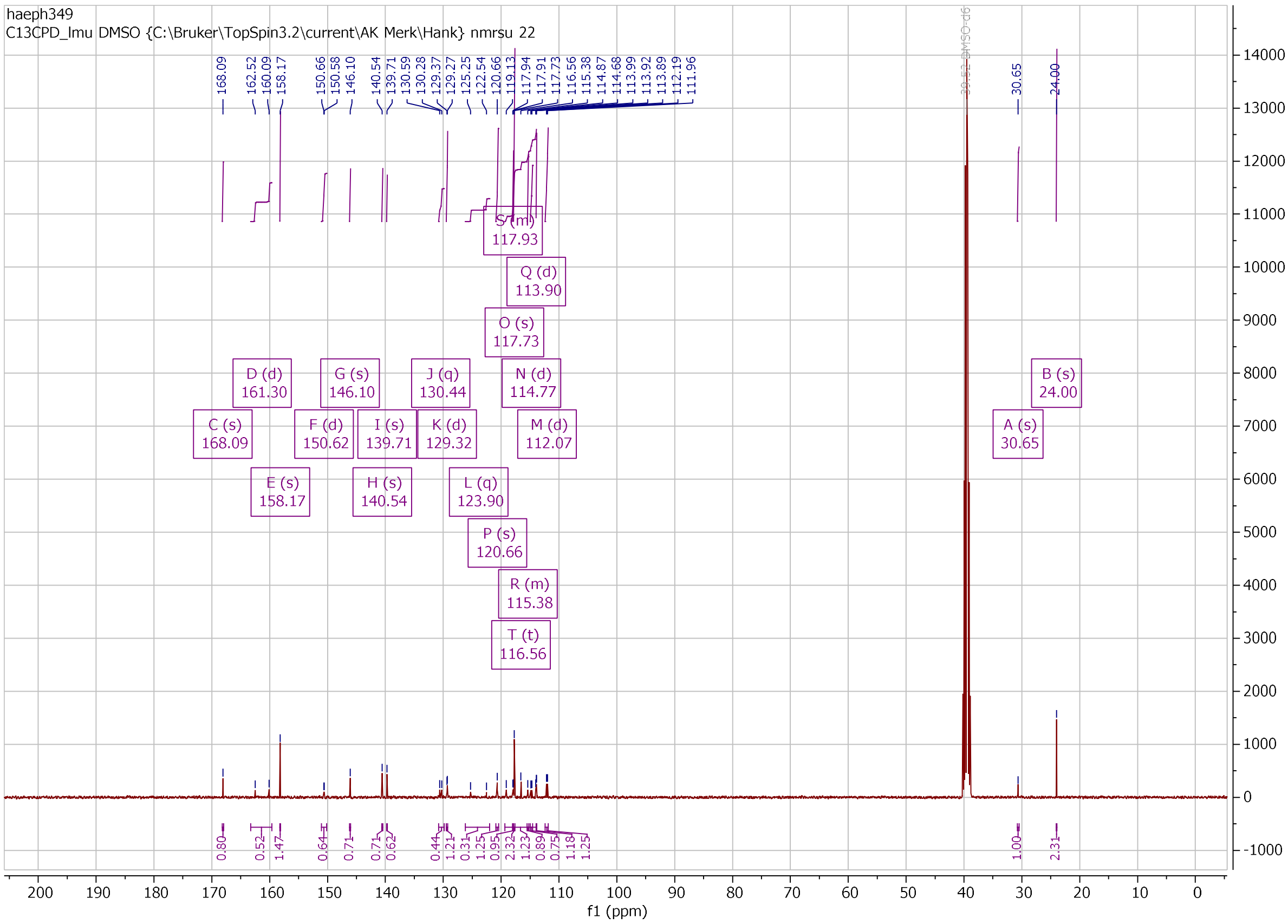
haeph349
C13CPD_lmu DMSO {C:\Bruker\TopSpin3.2\current\AK Merk\Hank} nmrsu 22
f1 (ppm)

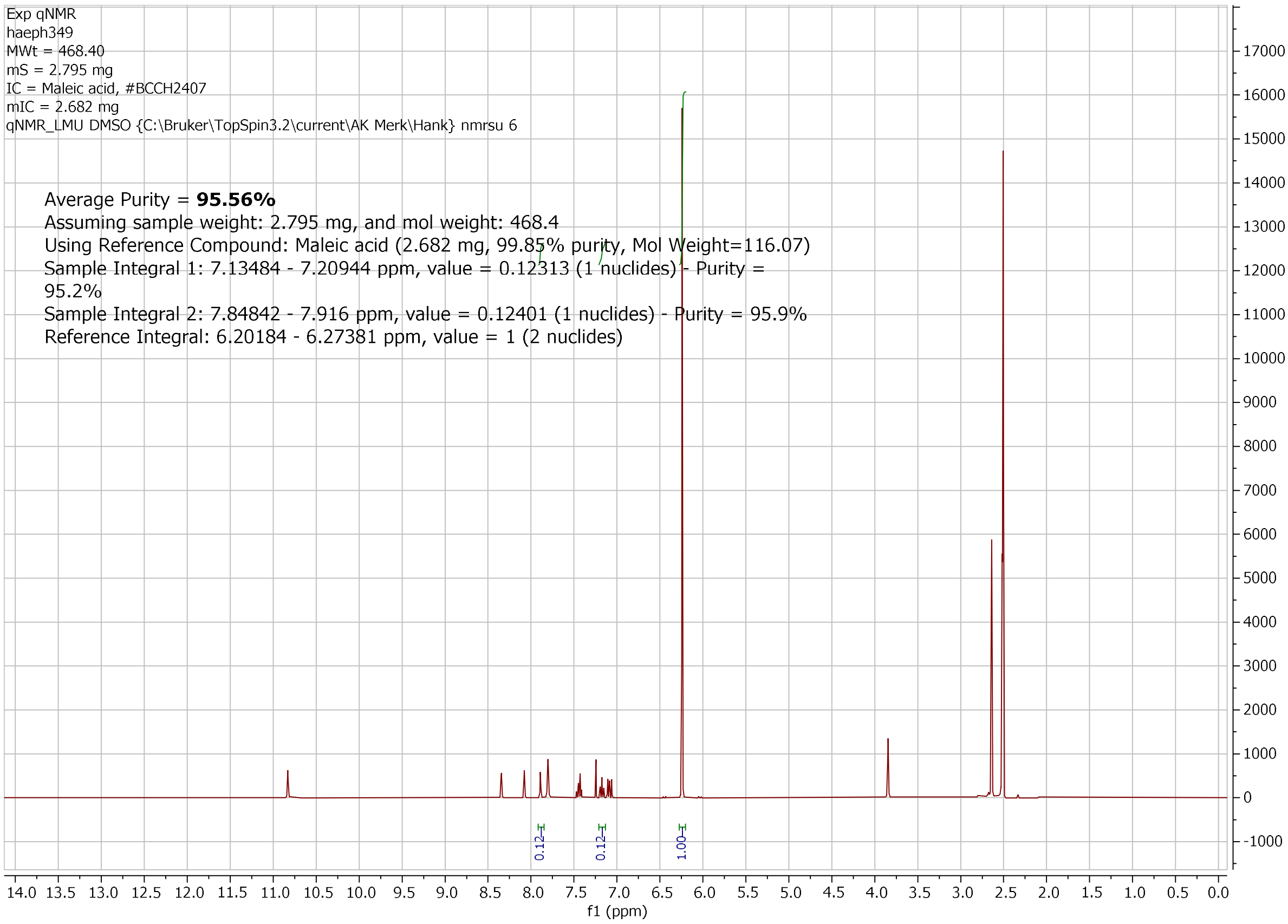
Exp qNMR
haeph349
MWt = 468.40
mS = 2.795 mg
IC = Maleic acid, #BCCH2407
mIC = 2.682 mg
qNMR_LMU DMSO {C:\Bruker\TopSpin3.2\current\AK Merk\Hank} nmrsu 6
Average Purity = 95.56%
Assuming sample weight: 2.795 mg, and mol weight: 468.4
Using Reference Compound: Maleic acid (2.682 mg, 99.85% purity, Mol Weight=116.07)
Sample Integral 1: 7.13484 - 7.20944 ppm, value = 0.12313 (1 nuclides) - Purity = 95.2%
Sample Integral 2: 7.84842 - 7.916 ppm, value = 0.12401 (1 nuclides) - Purity = 95.9%
Reference Integral: 6.20184 - 6.27381 ppm, value = 1 (2 nuclides)
f1 (ppm)

Compound **54**:

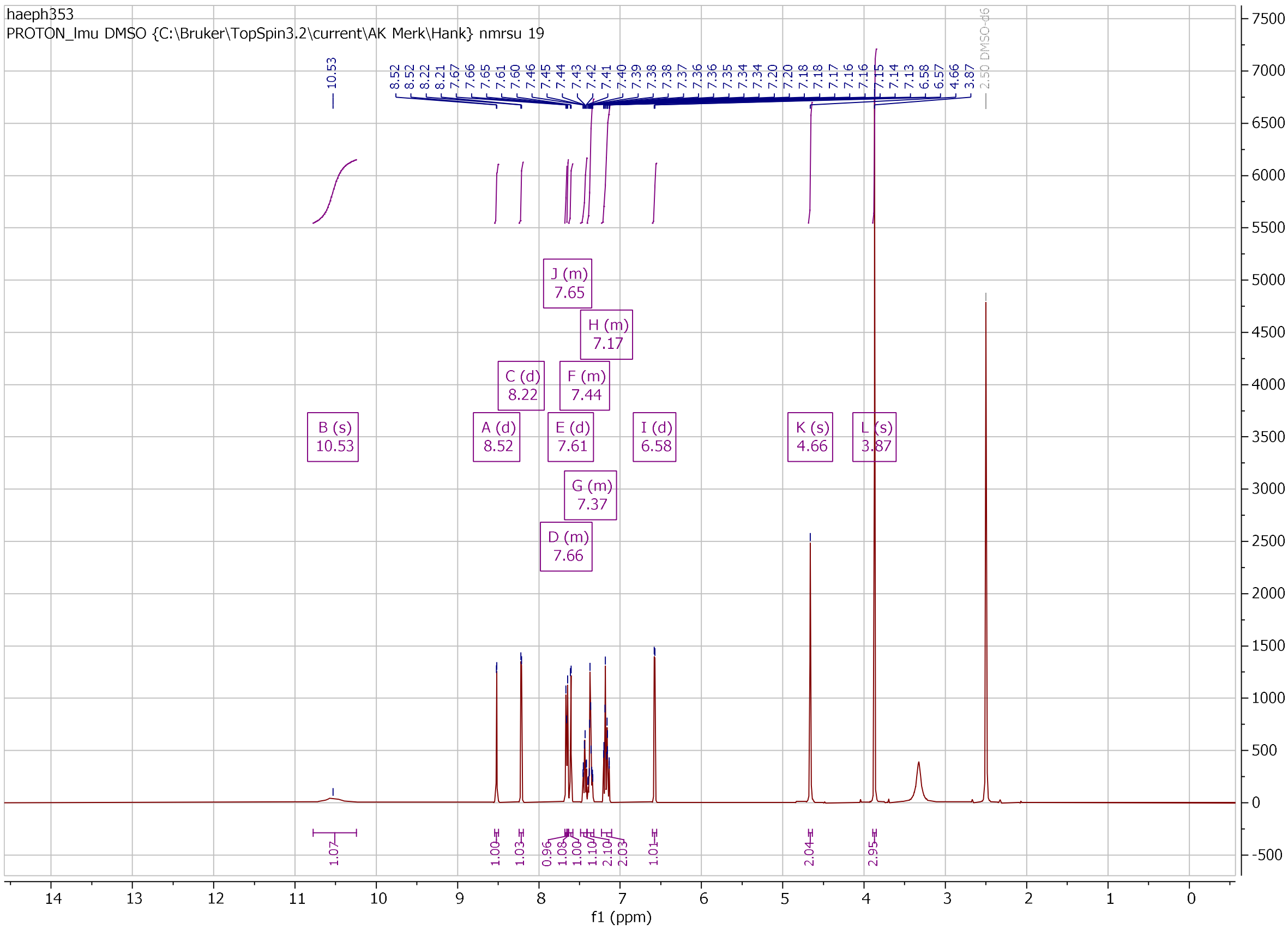
haeph353
PROTON_Imu DMSO {C:\Bruker\TopSpin3.2\current\AK Merk\Hank} nmrsu 19
J (m) 7.65
H (m) 7.17
C (d) 8.22
F (m) 7.44
B (s) 10.53
A (d) 8.52
E (d) 7.61
I (d) 6.58
K (s) 4.66
L (s) 3.87
G (m) 7.37
D (m) 7.66
f1 (ppm)

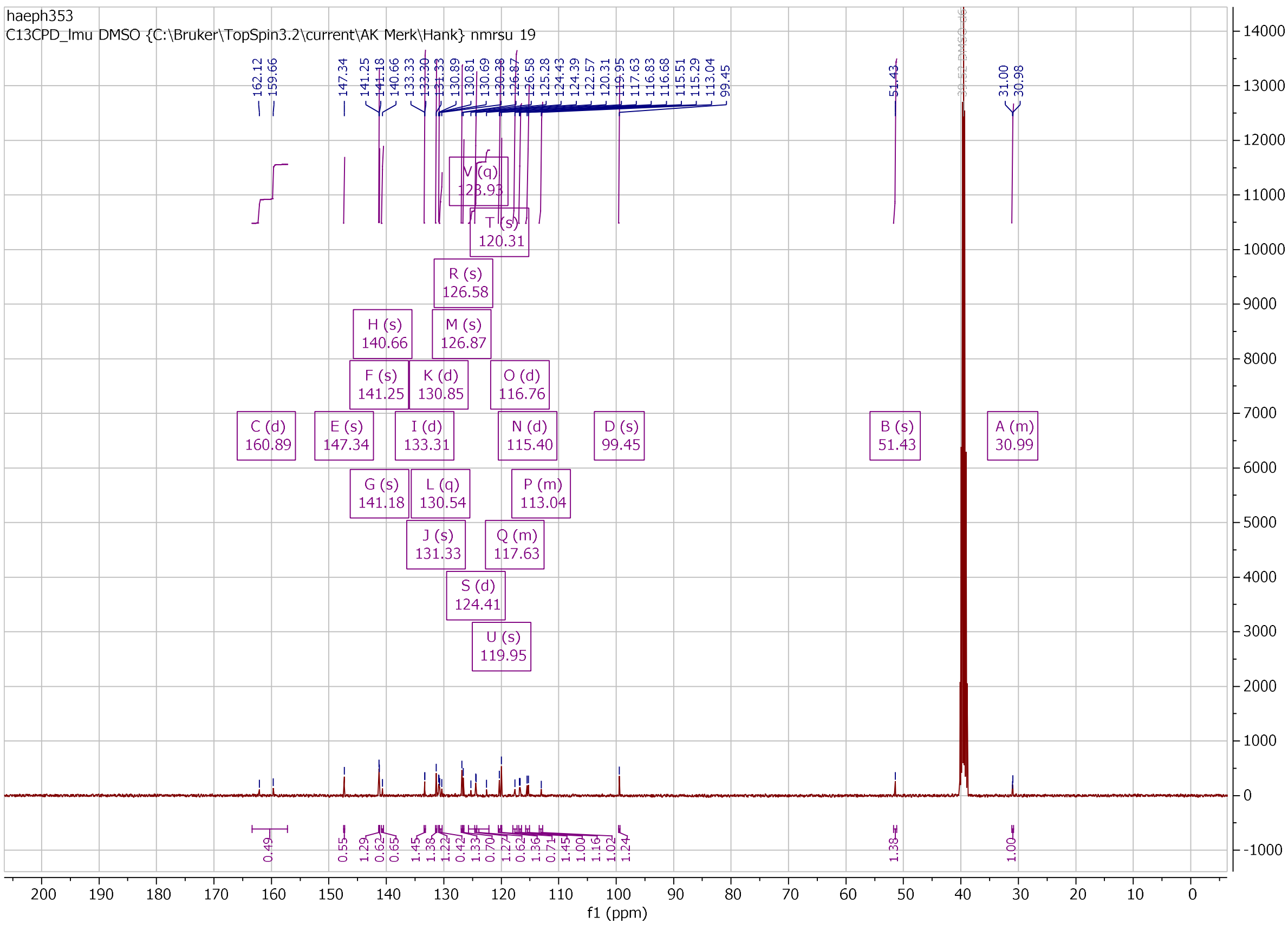
haeph353
C13CPD_Imu DMSO {C:\Bruker\TopSpin3.2\current\AK Merk\Hank} nmrsu 19
V (q) 128.93
T (s) 120.31
R (s) 126.58
H (s) 140.66
M (s) 126.87
F (s) 141.25
K (d) 130.85
O (d) 116.76
C (d) 160.89
E (s) 147.34
I (d) 133.31
N (d) 115.40
D (s) 99.45
B (s) 51.43
A (m) 30.99
G (s) 141.18
L (q) 130.54
P (m) 113.04
J (s) 131.33
Q (m) 117.63
S (d) 124.41
U (s) 119.95
f1 (ppm)

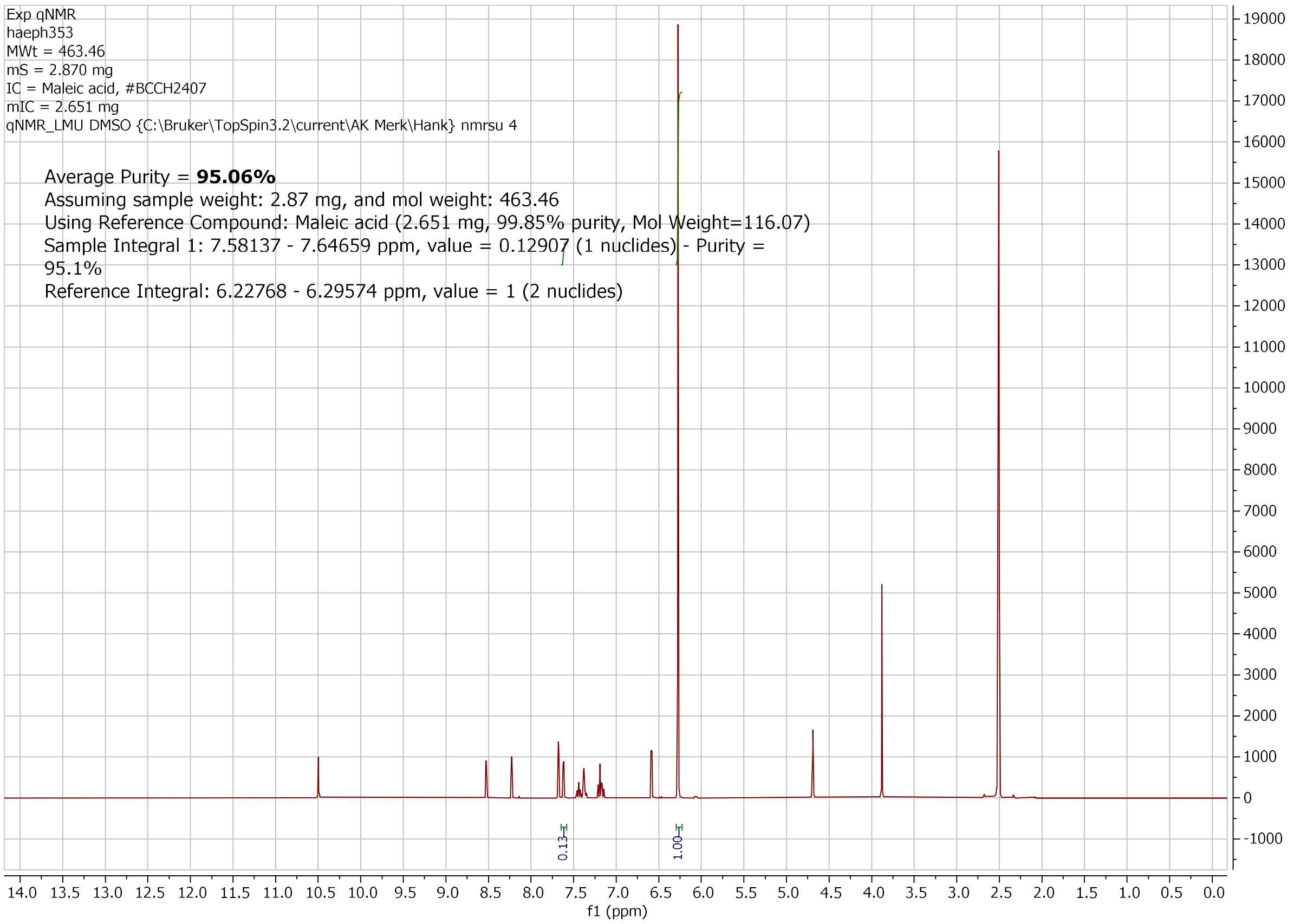
Exp qNMR
haeph353
MWt = 463.46
mS = 2.870 mg
IC = Maleic acid, #BCCH2407
mIC = 2.651 mg
qNMR_LMU DMSO {C:\Bruker\TopSpin3.2\current\AK Merk\Hank} nmrsu 4
Average Purity = **95.06%**
Assuming sample weight: 2.87 mg, and mol weight: 463.46
Using Reference Compound: Maleic acid (2.651 mg, 99.85% purity, Mol Weight=116.07)
Sample Integral 1: 7.58137 - 7.64659 ppm, value = 0.12907 (1 nuclides) - Purity = 95.1%
Reference Integral: 6.22768 - 6.29574 ppm, value = 1 (2 nuclides)
0.13
1.00
f1 (ppm)

Compound **55**:

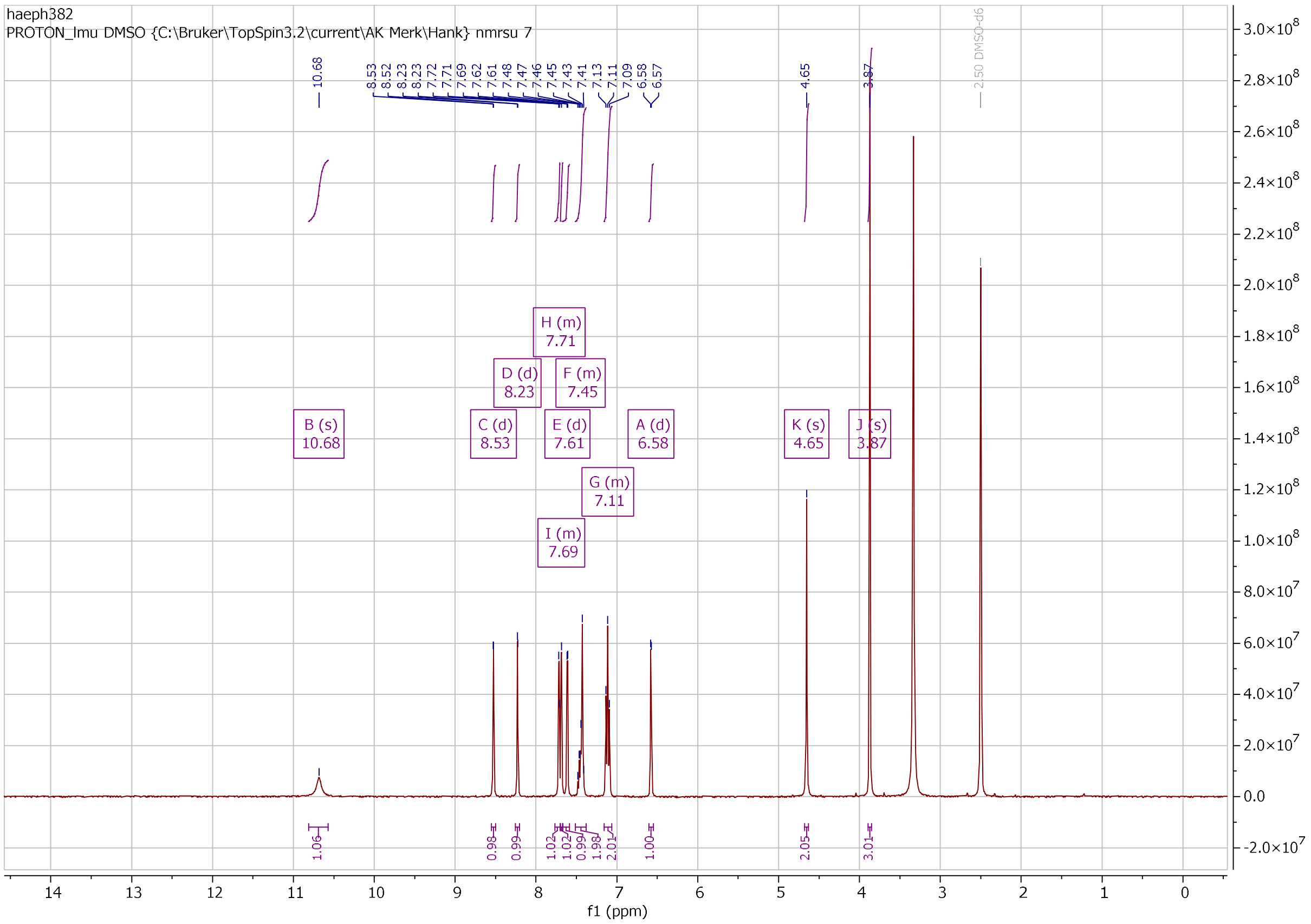
haeph382
PROTON_lmu DMSO {C:\Bruker\TopSpin3.2\current\AK Merk\Hank} nmrsu 7
10.68
8.53 8.52 8.23 8.23 7.72 7.71 7.69 7.62 7.61 7.48 7.47 7.46 7.45 7.43 7.41 7.13 7.11 7.09 6.58 6.57
4.65
3.87
2.50 DMSO-d6
H (m) 7.71
D (d) 8.23
F (m) 7.45
B (s) 10.68
C (d) 8.53
E (d) 7.61
A (d) 6.58
K (s) 4.65
J (s) 3.87
G (m) 7.11
I (m) 7.69
1.06
0.98
0.99
1.02
1.02
0.99
1.98
2.01
1.00
2.05
3.01
f1 (ppm)

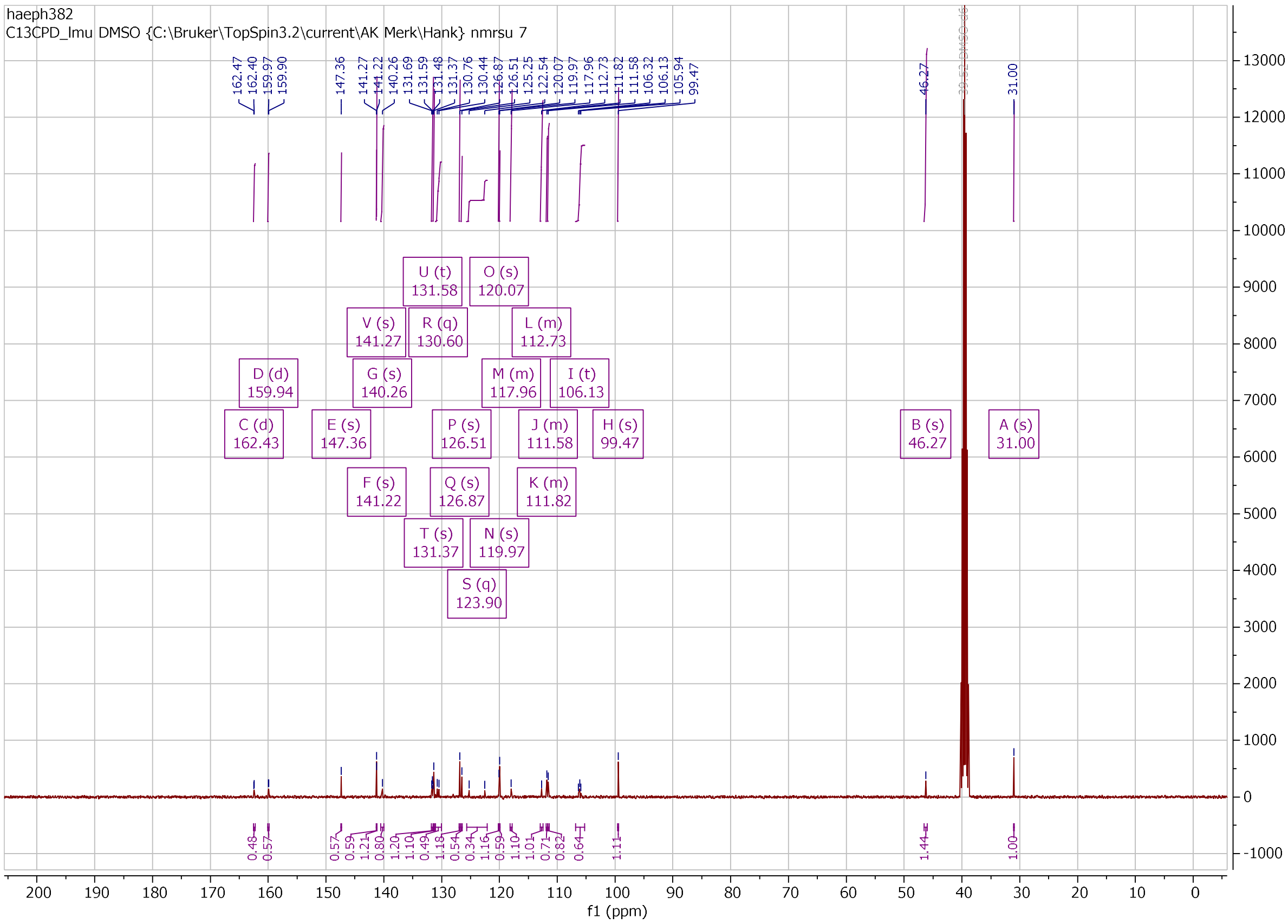
haeph382
C13CPD_lmu DMSO {C:\Bruker\TopSpin3.2\current\AK Merk\Hank} nmrsu 7
U (t) 131.58
O (s) 120.07
V (s) 141.27
R (q) 130.60
L (m) 112.73
D (d) 159.94
G (s) 140.26
M (m) 117.96
I (t) 106.13
C (d) 162.43
E (s) 147.36
P (s) 126.51
J (m) 111.58
H (s) 99.47
B (s) 46.27
A (s) 31.00
F (s) 141.22
Q (s) 126.87
K (m) 111.82
T (s) 131.37
N (s) 119.97
S (q) 123.90
f1 (ppm)

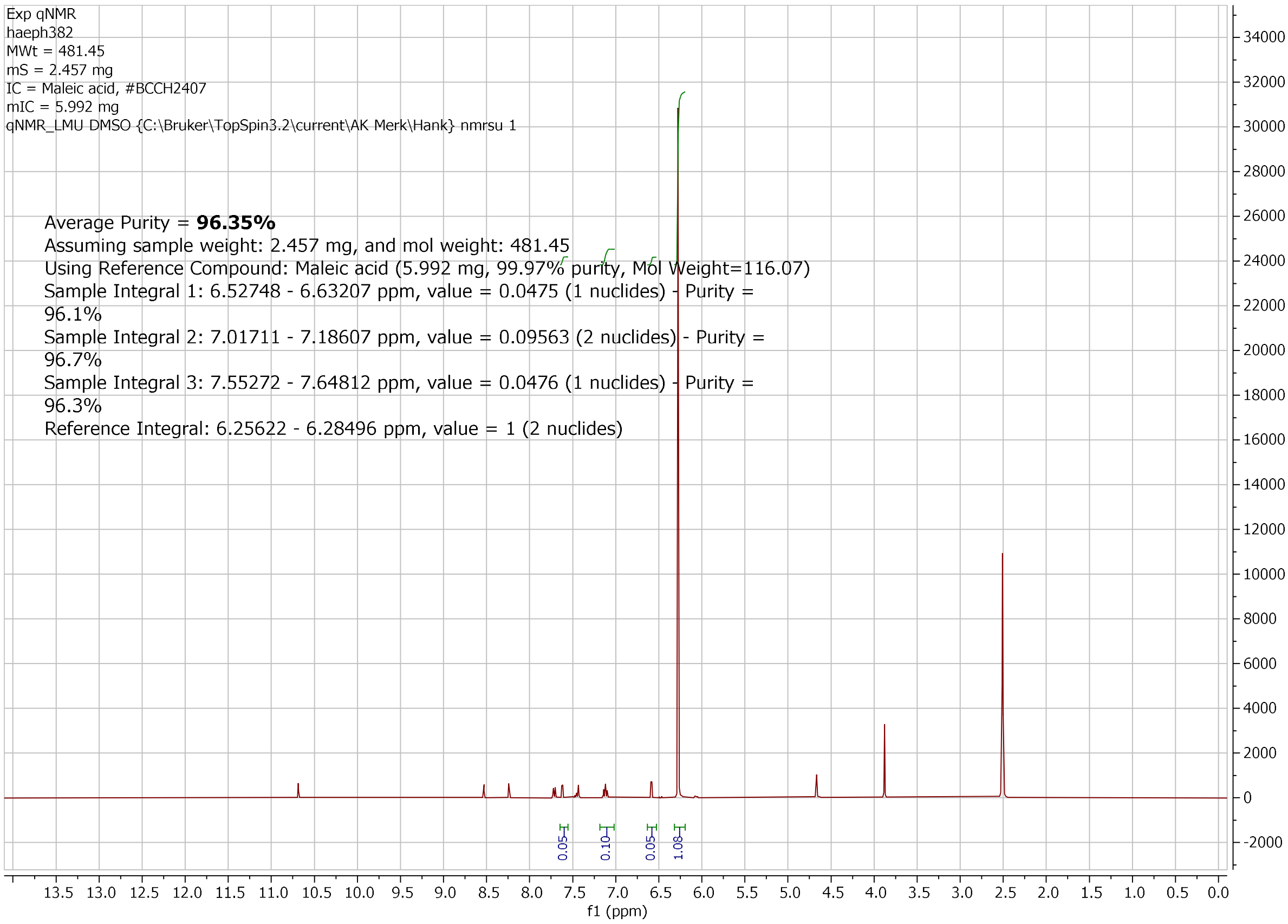
Exp qNMR
haeph382
MWt = 481.45
mS = 2.457 mg
IC = Maleic acid, #BCCH2407
mIC = 5.992 mg
qNMR_LMU DMSO {C:\Bruker\TopSpin3.2\current\AK Merk\Hank} nmrsu 1
Average Purity = 96.35%
Assuming sample weight: 2.457 mg, and mol weight: 481.45
Using Reference Compound: Maleic acid (5.992 mg, 99.97% purity, Mol Weight=116.07)
Sample Integral 1: 6.52748 - 6.63207 ppm, value = 0.0475 (1 nuclides) - Purity = 96.1%
Sample Integral 2: 7.01711 - 7.18607 ppm, value = 0.09563 (2 nuclides) - Purity = 96.7%
Sample Integral 3: 7.55272 - 7.64812 ppm, value = 0.0476 (1 nuclides) - Purity = 96.3%
Reference Integral: 6.25622 - 6.28496 ppm, value = 1 (2 nuclides)
f1 (ppm)

Compound **56**:

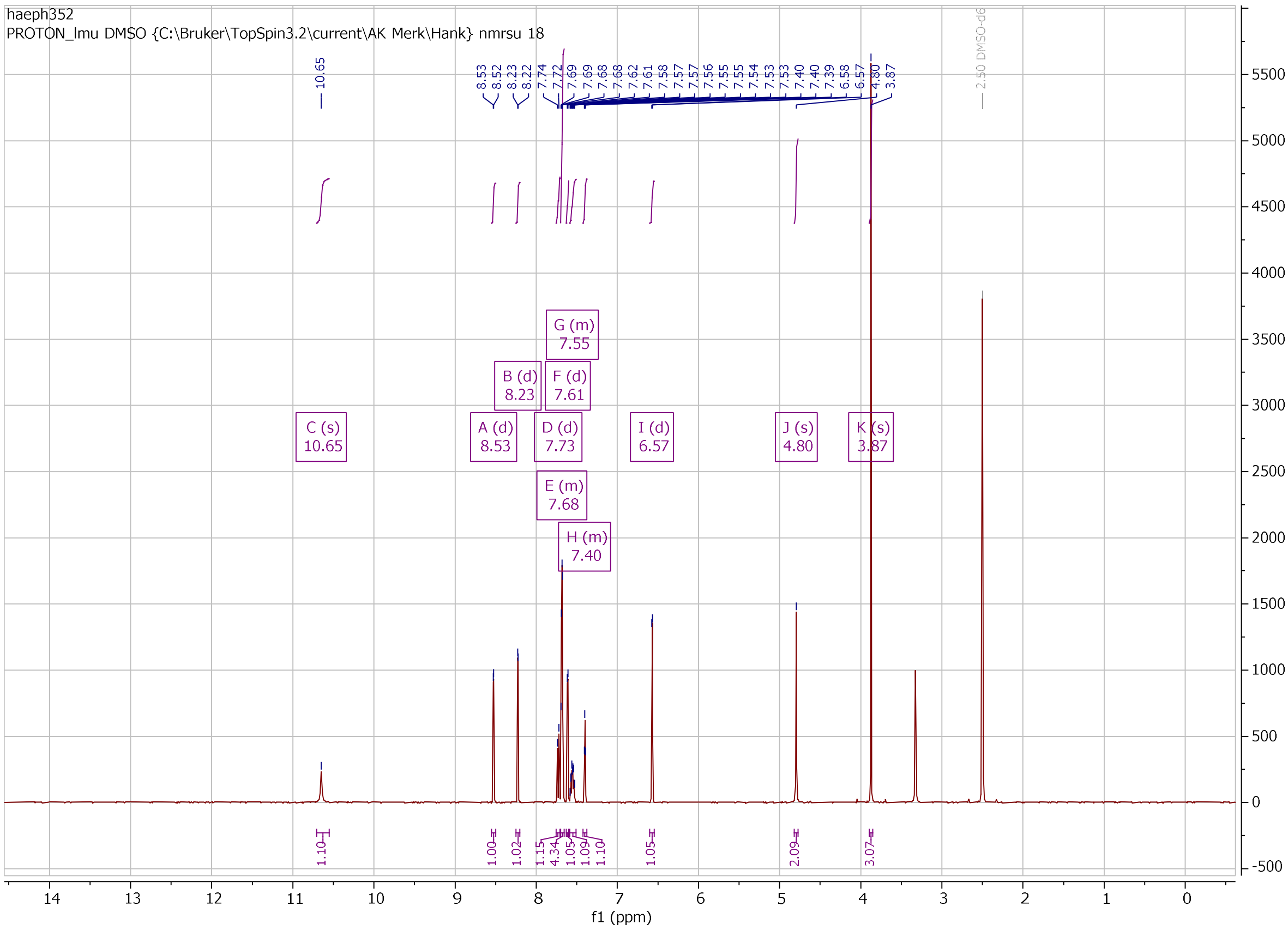
haeph352
PROTON_lmu DMSO {C:\Bruker\TopSpin3.2\current\AK Merk\Hank} nmrsu 18
C (s) 10.65
A (d) 8.53
B (d) 8.23
D (d) 7.73
E (m) 7.68
F (d) 7.61
G (m) 7.55
H (m) 7.40
I (d) 6.57
J (s) 4.80
K (s) 3.87
2.50 DMSO-d6
f1 (ppm)

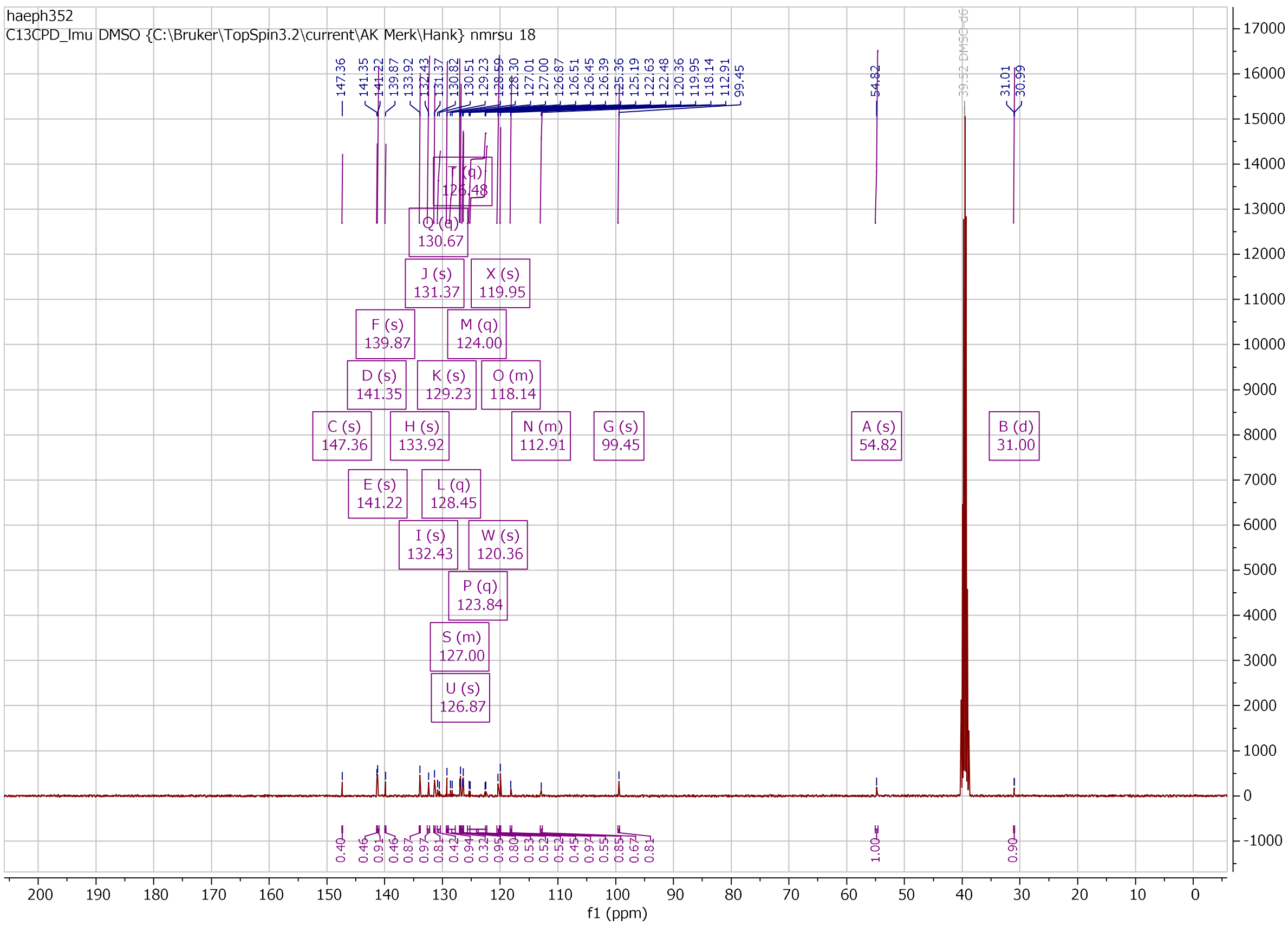
haeph352
C13CPD_lmu DMSO {C:\Bruker\TopSpin3.2\current\AK Merk\Hank} nmrsu 18
C (s) 147.36
D (s) 141.35
E (s) 141.22
F (s) 139.87
H (s) 133.92
I (s) 132.43
J (s) 131.37
Q (q) 130.67
K (s) 129.23
L (q) 128.45
S (m) 127.00
U (s) 126.87
T (q) 125.48
M (q) 124.00
P (q) 123.84
W (s) 120.36
X (s) 119.95
O (m) 118.14
N (m) 112.91
G (s) 99.45
A (s) 54.82
B (d) 31.00
39.52 DMSO-d6
f1 (ppm)

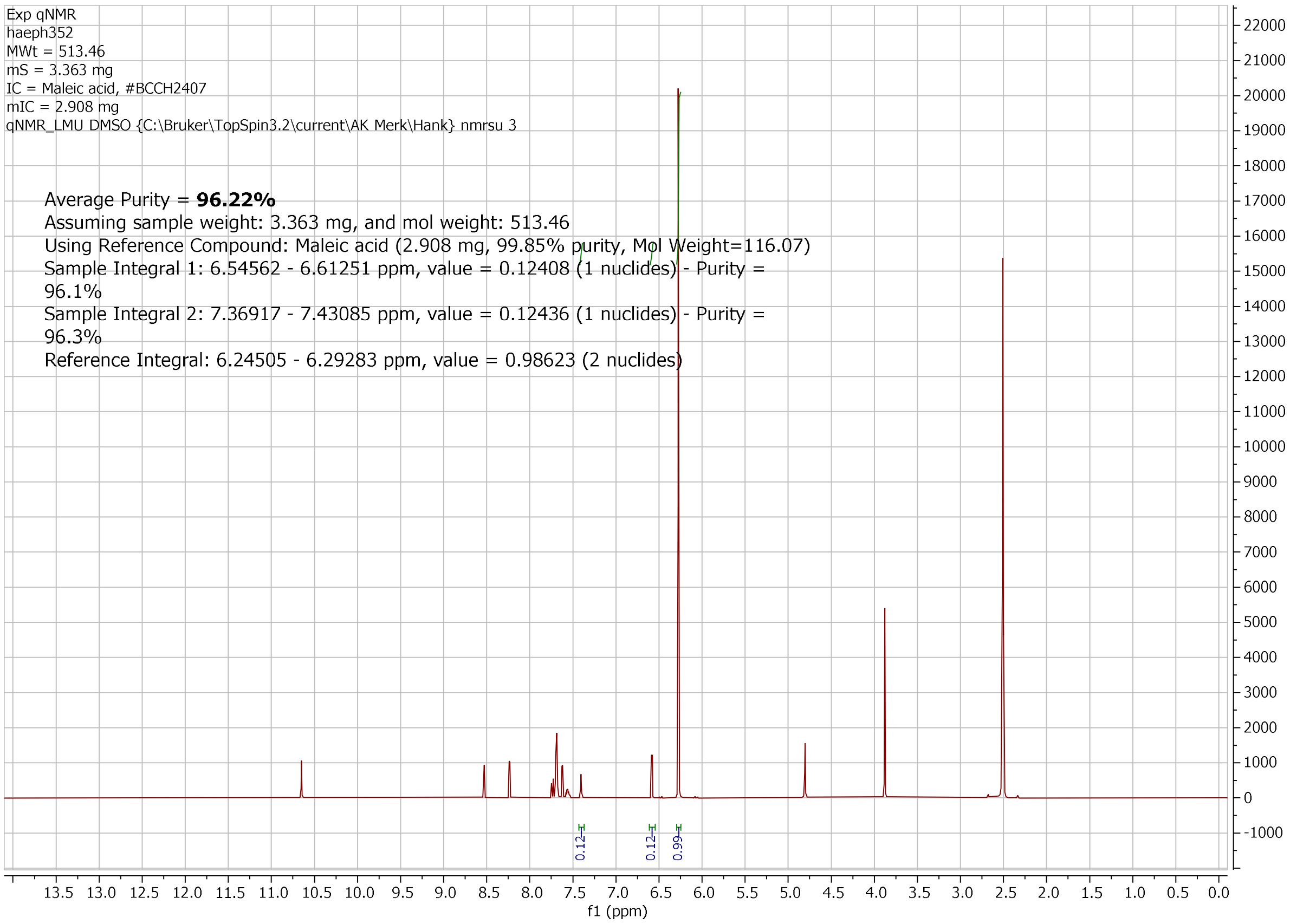
Exp qNMR
haeph352
MWt = 513.46
mS = 3.363 mg
IC = Maleic acid, #BCCH2407
mIC = 2.908 mg
qNMR_LMU DMSO {C:\Bruker\TopSpin3.2\current\AK Merk\Hank} nmrsu 3
Average Purity = **96.22%**
Assuming sample weight: 3.363 mg, and mol weight: 513.46
Using Reference Compound: Maleic acid (2.908 mg, 99.85% purity, Mol Weight=116.07)
Sample Integral 1: 6.54562 - 6.61251 ppm, value = 0.12408 (1 nuclides) - Purity = 96.1%
Sample Integral 2: 7.36917 - 7.43085 ppm, value = 0.12436 (1 nuclides) - Purity = 96.3%
Reference Integral: 6.24505 - 6.29283 ppm, value = 0.98623 (2 nuclides)
f1 (ppm)

Compound **58**:

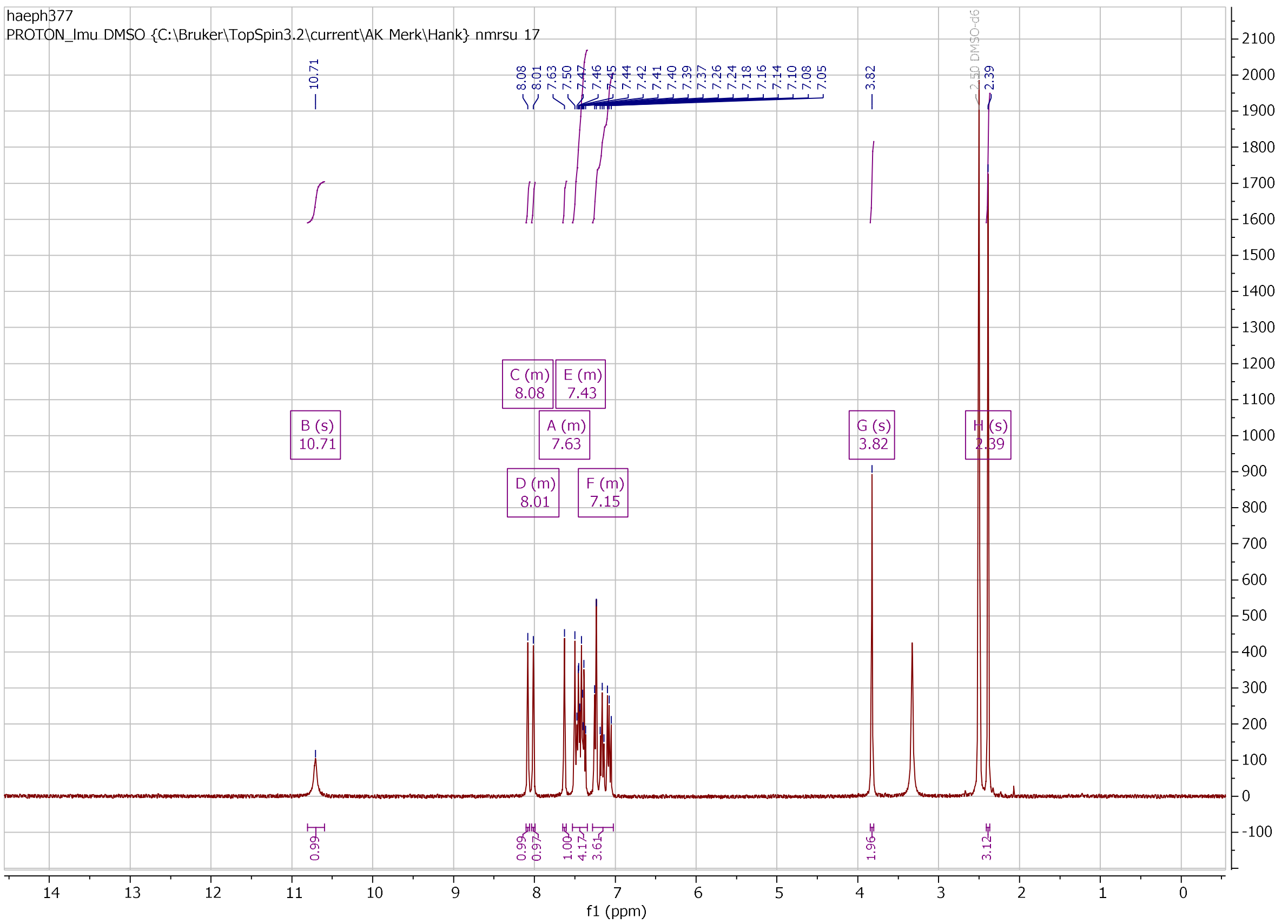
haeph377
PROTON_lmu DMSO {C:\Bruker\TopSpin3.2\current\AK Merk\Hank} nmrsu 17
B (s) 10.71
C (m) 8.08
D (m) 8.01
A (m) 7.63
E (m) 7.43
F (m) 7.15
G (s) 3.82
H (s) 2.39
f1 (ppm)

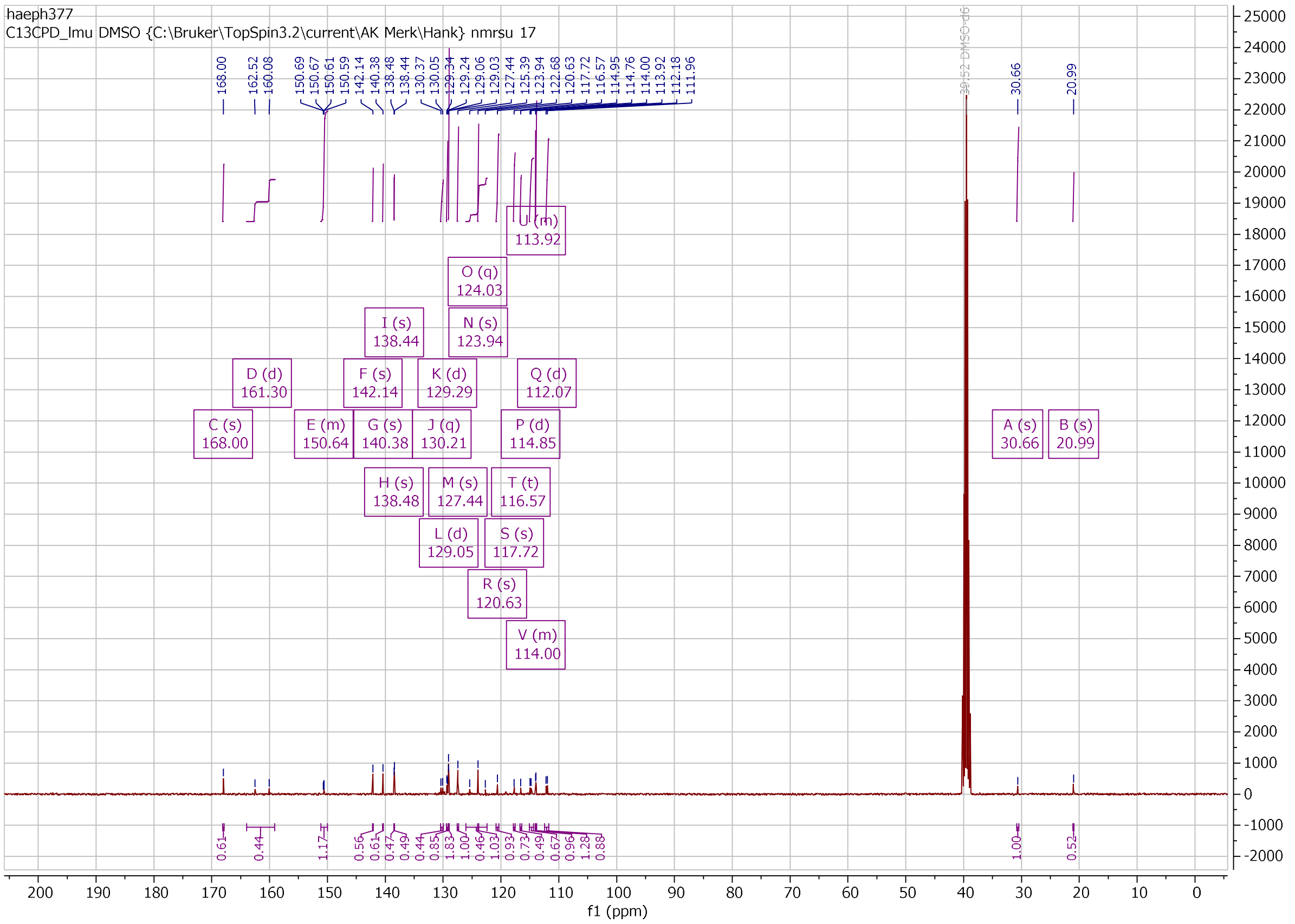
haeph377
C13CPD_lmu DMSO {C:\Bruker\TopSpin3.2\current\AK Merk\Hank} nmrsu 17
f1 (ppm)

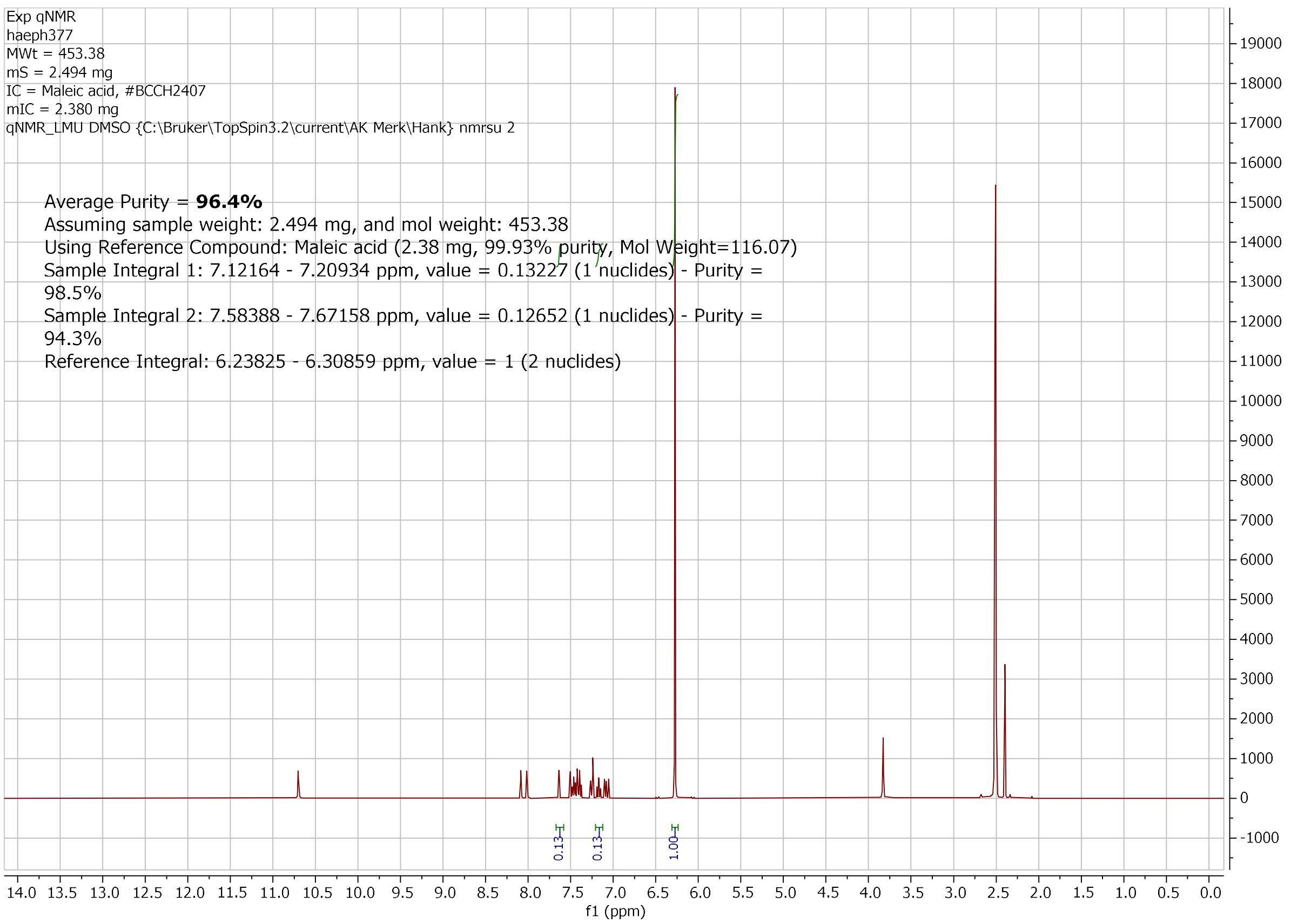
Exp qNMR
haeph377
MWt = 453.38
mS = 2.494 mg
IC = Maleic acid, #BCCH2407
mIC = 2.380 mg
qNMR_LMU DMSO {C:\Bruker\TopSpin3.2\current\AK Merk\Hank} nmrsu 2
Average Purity = **96.4%**
Assuming sample weight: 2.494 mg, and mol weight: 453.38
Using Reference Compound: Maleic acid (2.38 mg, 99.93% purity, Mol Weight=116.07)
Sample Integral 1: 7.12164 - 7.20934 ppm, value = 0.13227 (1 nuclides) - Purity = 98.5%
Sample Integral 2: 7.58388 - 7.67158 ppm, value = 0.12652 (1 nuclides) - Purity = 94.3%
Reference Integral: 6.23825 - 6.30859 ppm, value = 1 (2 nuclides)
f1 (ppm)

Compound **59**:

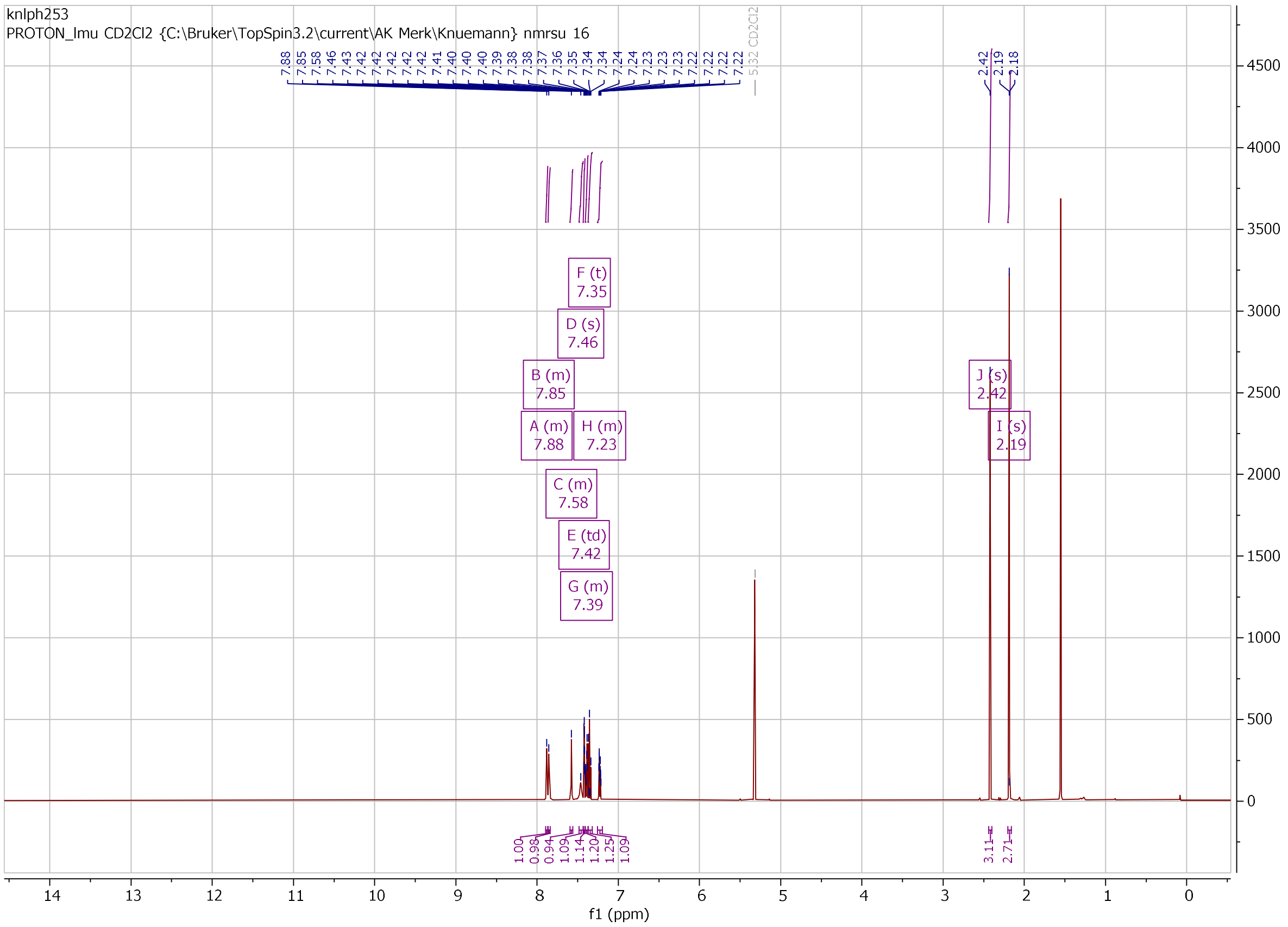
knlph253
PROTON_Imu CD2Cl2 {C:\Bruker\TopSpin3.2\current\AK Merk\Knuemann} nmrsu 16
f1 (ppm)

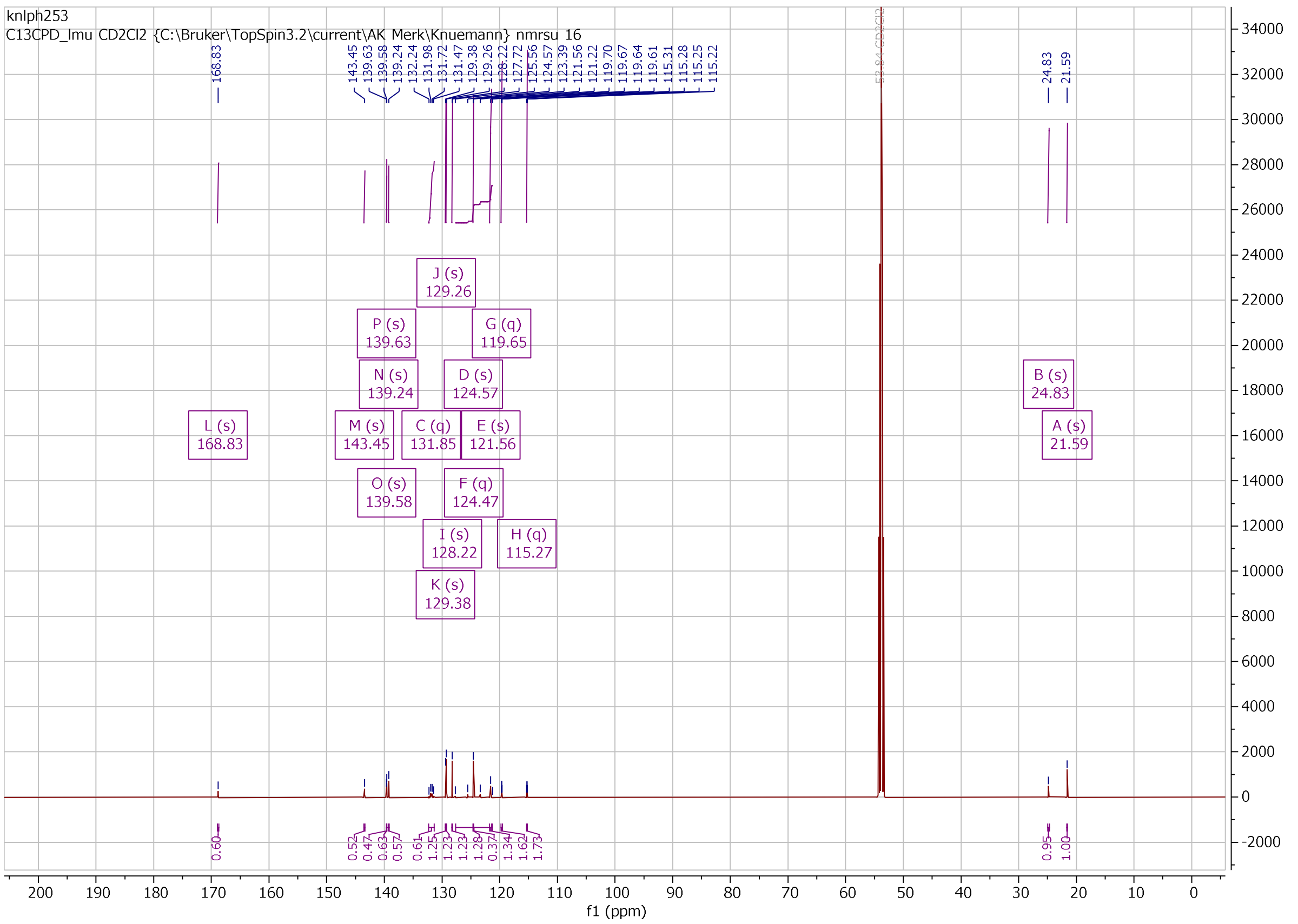
knlph253
C13CPD_Imu CD2Cl2 {C:\Bruker\TopSpin3.2\current\AK Merk\Knuemann} nmrsu 16
f1 (ppm)

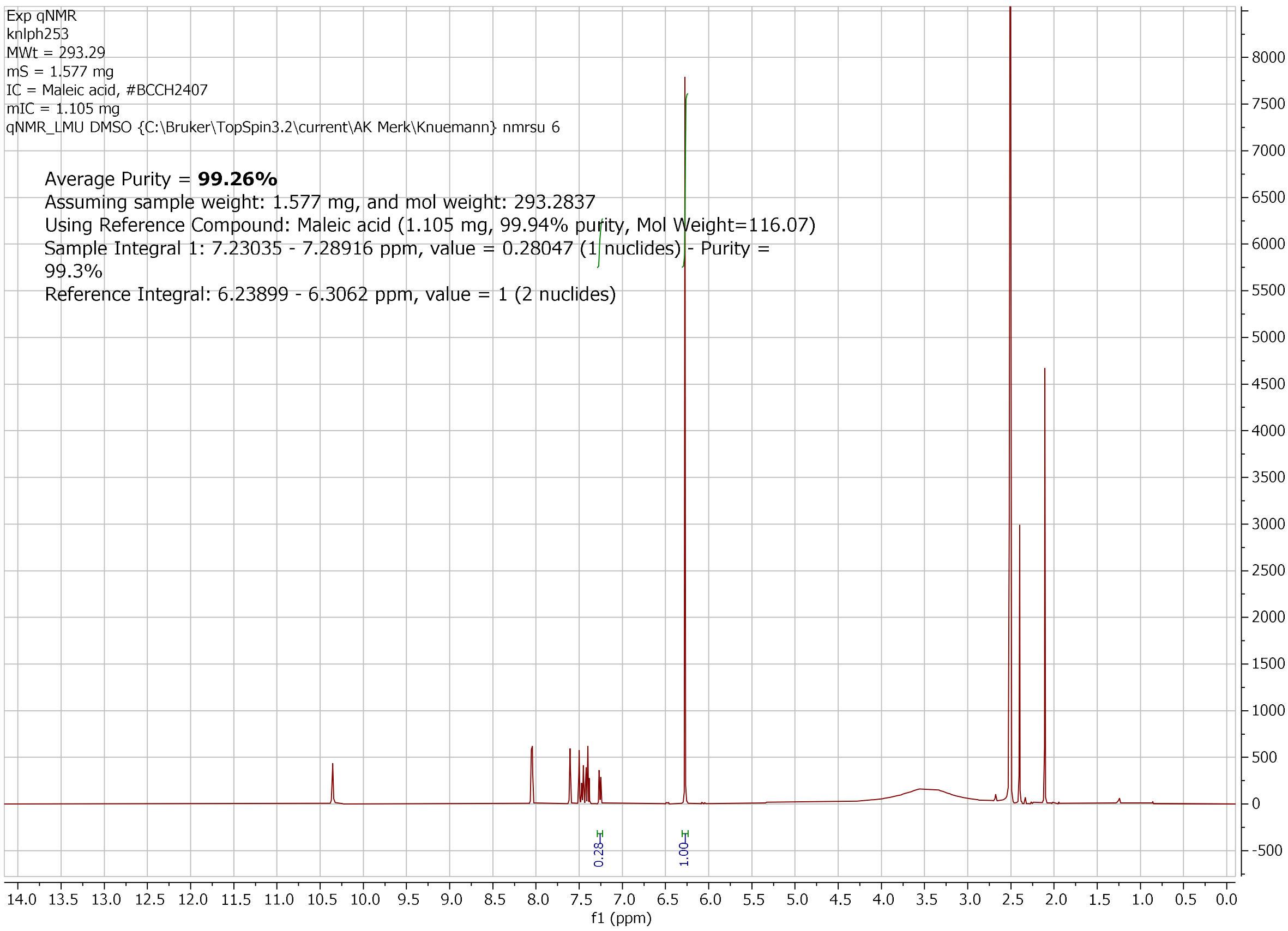

Exp qNMR
knlph253
MWt = 293.29
mS = 1.577 mg
IC = Maleic acid, #BCCH2407
mIC = 1.105 mg
qNMR_LMU DMSO {C:\Bruker\TopSpin3.2\current\AK Merk\Knuemann} nmrsu 6
Average Purity = **99.26%**
Assuming sample weight: 1.577 mg, and mol weight: 293.2837
Using Reference Compound: Maleic acid (1.105 mg, 99.94% purity, Mol Weight=116.07)
Sample Integral 1: 7.23035 - 7.28916 ppm, value = 0.28047 (1 nuclides) - Purity = 99.3%
Reference Integral: 6.23899 - 6.3062 ppm, value = 1 (2 nuclides)
0.28
1.00
f1 (ppm)

Supplementary References

(1) Kanehisa, M.; Furumichi, M.; Sato, Y.; Kawashima, M.; Ishiguro-Watanabe, M. KEGG for Taxonomy-Based Analysis of Pathways and Genomes. *Nucleic Acids Res.* **2023**, *51* (D1), D587–D592.

(2) Vietor, J.; Gege, C.; Stiller, T.; Busch, R.; Schallmayer, E.; Kohlhof, H.; Höfner, G.; Pabel, J.; Marschner, J. A.; Merk, D. Development of a Potent Nurr1 Agonist Tool for in Vivo Applications. *J. Med. Chem.* **2023**, *66* (9), 6391–6402.
